# Losartan and prednisolone for post-COVID syndrome and cardiac inflammation: a randomized, double-blind, placebo-controlled trial

**DOI:** 10.1038/s41467-026-75991-w

**Published:** 2026-07-30

**Authors:** Valentina O. Puntmann, Eike Nagel, Dietrich Beitzke, Andreas Kammerlander, Inga Voges, Marcus Doerr, Bishwas Chamling, Biykem Bozkurt, Juan Carlos Kaski, Erica Spatz, Eva Herrmann, Gernot Rohde, Philipp DeLeuw, Christine Windemuth-Kieselbach, Sebastian Eckhardt, Peter C. Taylor, Colin Berry

**Affiliations:** 1 DZHK Centre for Cardiovascular Imaging, Institute for Experimental and Translational Cardiovascular Imaging, German Centre for Cardiovascular Research – Partner Site Rhein-Main, Goethe University Frankfurt, Frankfurt am Main, Germany; 2 Cardio-Pulmonary Institute, Rhein-Main, Site Frankfurt-am-Main, Germany; 3 Department of Cardiology, University Hospital Frankfurt, Frankfurt am Main, Germany; 4 Division of Cardiovascular and Interventional Radiology, Department of Biomedical Imaging and Image-guided Therapy, University Hospital Vienna, Vienna, Austria; 5 Department of Cardiology, University Hospital Vienna, Vienna, Austria; 6 Department of Cardiology, University Hospital Schleswig-Holstein, Campus Kiel, German Centre for Cardiovascular Research - Partner Site Hamburg/Kiel/Lübeck, Kiel, Germany; 7 Cardiology, Angiology, and Pulmonology, Department of Internal Medicine B, University Hospital Greifswald, Germany, German Centre for Cardiovascular Research – Partner Site Greifswald, Greifswald, Germany; 8 Winters Center for Heart Failure Research; Cardiovascular Research Institute, DeBakey VA Medical Center, Baylor College of Medicine, Houston, TX USA; 9 Cardiovascular Sciences, Molecular and Clinical Sciences, St George’s, University of London, London, UK; 10 Yale Center for Outcomes Research and Evaluation, Section of Cardiovascular Medicine, Yale School of Medicine, New Haven, CT USA; 11 Institute of Biostatistics and Mathematical Modelling, German Centre for Cardiovascular Research – Partner Site Rhein-Main; Goethe University Frankfurt, Frankfurt am Main, Germany; 12 Department of Respiratory Medicine, University Hospital, Frankfurt am Main, Germany; 13 Infektologikum Frankfurt, Frankfurt am Main, Germany; 14 Alcedis GmbH (CRO), Giessen, Germany; 15 Nuffield Department of Orthopaedics, Rheumatology and Musculoskeletal Sciences, University of Oxford, Oxford, UK; 16 BHF Glasgow Cardiovascular Research Centre (GCRC), School of Cardiovascular & Metabolic Health, University of Glasgow, Glasgow, UK

**Keywords:** Randomized controlled trials, Arterial stiffening

## Abstract

Persistent cardiac symptoms are common in post-COVID syndrome, even without structural heart disease. Evidence implicates immune dysregulation, endothelial dysfunction and low-grade cardiovascular inflammation. Yet no targeted treatment exists. Myoflame-19 is a multicenter, double-blind clinical trial of 279 participants with inflammatory cardiac involvement defined by cardiovascular magnetic resonance, randomized 1:1 to losartan plus prednisolone (*n = 139*) or matching placebos (*n = 140*) for 16 weeks. The modified intention-to-treat population comprised 124 and 122 participants. The primary endpoint, change in left ventricular (LV) ejection fraction, was neutral: between-group difference 0.74 percentage points (pp), 95%CI −0.14 to 1.62, p = 0.10, unpaired t-test; supportive baseline-adjusted ANCOVA 0.99, 95%CI 0.15-1.83, p = 0.021. Among prespecified secondary endpoints, several symptom and imaging measures showed numerical differences favoring intervention, including Average Symptom Score components (modified Canadian Chest Pain Scale −4.8 pp, 95%CI −17.3 to 7.6; NYHA class −8.1 pp, −20.6 to 4.3; Long COVID symptom burden −7.7 pp, −18.9 to 3.6), native T1 and T2 values (native T1 −2.46 ms, −8.35 to 3.42; native T2 −0.31 ms, −1.16 to 0.53), and LV end-diastolic volume ( + 1.45 ml/m², −0.09 to 3.00); however, confidence intervals included the null value and these findings should be regarded as hypothesis-generating.Treatment was safe and well-tolerated. These findings indicate a neutral treatment effect on the primary endpoint. They inform targeted immunomodulation and design of future trials in post-COVID syndrome and inflammatory cardiac involvement. Trial registration: EudraCT 2022-001682-12; NCT05619653.

## Abstract (web summary)

Persistent cardiac symptoms are common in post-COVID syndrome. In the Myoflame-19 trial, losartan plus prednisolone yielded neutral results for the primary outcome, change in left ventricular ejection fraction, in 279 participants with inflammatory cardiac involvement assessed by cardiovascular magnetic resonance.

## Introduction

Persistent cardiac symptoms, effort intolerance and exertion-induced fatigue are recognized consequences of COVID-19 infection, even among previously healthy individuals with mild acute illness^1–4^. The emerging post-COVID syndrome is associated with reduced quality of life (QoL) and substantial functional limitation, affecting a large segment of the working-age population. A body of evidence demonstrates a complex and dynamic inflammatory pathophysiology, evolving from infection-triggered immune dysregulation to autoimmune and autoinflammatory end-organ injury^5–11^. Some of the proposed mechanisms include angiotensin-II induced endothelial dysfunction, microvascular injury and sustained myocardial inflammation^8,12–15^. The resulting tissue hypoperfusion, metabolic derangements and mitochondrial dysfunction^16–18^, offer, at least in part, a mechanistic explanation for the heterogeneous multisystem symptoms observed in post-COVID syndrome^1,19^. Despite this expanding mechanistic insight, no evidence-based therapy has yet been shown to modify inflammatory cardiac involvement in this setting^5^.

Cardiovascular magnetic resonance (CMR) studies have consistently demonstrated a pattern of low-grade inflammatory cardiac involvement with elevated T1 and T2 mapping values, non-ischemic perimyocardial late gadolinium enhancement and mild systolic dysfunction within a preserved low-normal range, which typically persists over time and occurs in the absence of structural heart disease^3,19–21^.

Losartan and low-dose prednisolone offer a biologically plausible therapeutic strategy to target angiotensin-II-driven endothelial dysfunction^22^ and attenuate low-grade myocardial inflammation. Both agents have well-characterized cardioprotective and immunomodulatory effects across conditions involving inflammatory myocardial remodeling^23^, including hypertension^24^, diabetes^25^, cardio-oncology^26^, and postviral syndromes^27^. A combined cardioprotective and immunomodulatory therapy may mitigate inflammation-induced endothelial dysfunction and improve effort tolerance^28^. The Myoflame-19 trial was designed to test the hypothesis that targeted cardioprotection and immunomodulation with losartan and low-dose prednisolone improve cardiac function in individuals with symptomatic post-COVID syndrome and CMR-defined cardiac inflammation. Using imaging-based selection and a quantitative CMR-derived primary endpoint, the trial evaluated whether a 16-week intervention could lead to a measurable improvement in left ventricular ejection fraction (LVEF) compared with placebo in this inflammatory cardiac endotype.

## Results

### Baseline

**Fig. 1:**
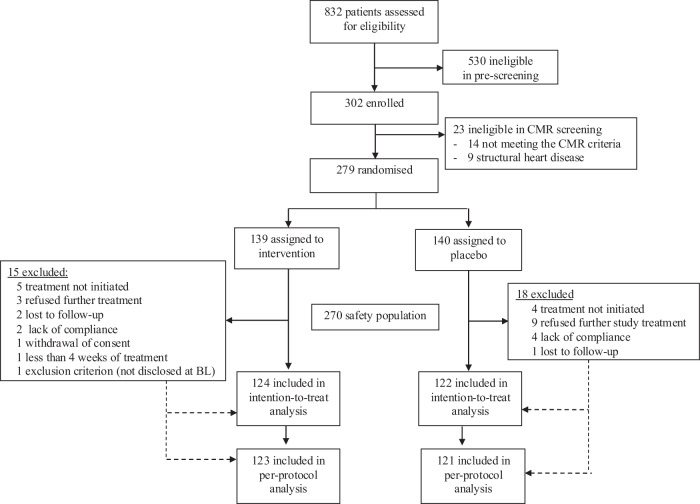
CONSORT flow diagram of the study population. CONSORT diagram summarizing participant screening, randomization, treatment allocation and follow-up. The safety population included all participants who received ≥1 dose of study medication (*n* = 270). Modified intention-to-treat population consisted of a total of 246 participants who received ≥4 weeks of treatment and completed the 16-week follow-up CMR; the per-protocol population comprised 244 participants taking study medication for 75% of the study time, excluding two participants who received ≥4 weeks of treatment but discontinued study medication before reaching the prespecified adherence threshold required for per-protocol inclusion.

The Myoflame-19 participant flow and population derivation are detailed in the CONSORT diagram (Fig. 1). Between December 2022 and March 2025, four centers (Frankfurt, Kiel, Greifswald, and Vienna) screened candidates across five European countries (Germany, Austria, Switzerland, Luxembourg, and Poland). Eligible individuals underwent comprehensive baseline clinical, laboratory and imaging assessments, with standardized CMR used to exclude pre-existing structural heart disease and to confirm the presence of CMR-defined cardiac inflammation required for randomization. Of the 279 randomized participants, paired CMR data were available in 246 (88%) of randomized participants, who were included in the modified intention-to-treat efficacy analysis, representing the full analysis set for efficacy analyses of the primary endpoint, necessitating the participants to complete both baseline and week 16 (W16) imaging. The per-protocol population further required ≥4 weeks of treatment exposure and no major protocol deviations. The safety population included all participants receiving ≥1 dose of study medication (*n* = 270). The reasons for non-completion included treatment non-initiation (*n* = 9), refusal of study therapy (*n* = 12), lack of compliance (*n* = 6), loss to follow-up (*n* = 3), and others (*n* = 3), with similar distribution across treatment groups (*n* = 33, 12%; intervention vs. placebo: 12.2 vs. 11.4%; CONSORT diagram (Fig. 1)).

Baseline demographic and clinical characteristics of the modified intention-to-treat population were well balanced between groups and are presented in Table 1 (the complete dataset, 279 randomized participants and per protocol population is presented in Supplementary Tables S2 and S3). Median age was 39 years, 73% were women, most had ≥2 prior COVID-19 infections, and 97% were vaccinated (60% with three doses). Symptom duration was similar between groups. Symptom burden was high at baseline: 85% reported ≥5 persistent symptoms, most commonly fatigue, dyspnea and chest tightness, with substantial functional limitation. QoL scores showed marked physical and mental health impairment in both groups. Prior medication use (75% overall) included analgesics (13%), antihistamines (12%), antithrombotics (11%), low-dose naltrexone (10%), antidepressants (9%) and antacids (8%). Hypothyroidism was present in 14%.

**Table 1:** Baseline demographic, clinical, and CMR characteristics by treatment group (intention-to-treat population)

Parameter	Intervention, *N* = 124, median [Q1, Q3], *n*, %	Placebo, *N* = 122, median [Q1, Q3], *n*, %
Demographics		
Age (years)	38.5 (32.0, 46.0)	39.0 (30.0, 46.0)
Females, *n*, %	96 (77.4)	83 (68.0)
Ethnic origin (Caucasian), *n*, %	116 (93.5)	115 (94.3)
BMI (kg/m^2^)	24.1 (21.3, 27.0)	23.7 (21.1, 28.1)
BP systolic (mmHg)	124.0 (112.0, 133.0)	124.0 (115.0, 134.0)
BP diastolic (mmHg)	81.0 (74.0, 88.0)	83.0 (74.0, 91.0)
Heart rate (bpm)	75.0 (67.0, 83.0)	77.0 (69.0, 85.0)
More than 1 COVID-19 infection, *n*, %	68 (54.8)	78 (63.9)
COVID-19 reinfection during the study, *n*, %	8 (6.5)	10 (8.2)
Number of COVID-19 vaccinations taken	3.0 (3.0, 3.0)	3.0 (3.0, 3.0)
More than 2 COVID-19 vaccinations, *n*, %	95 (76.6)	104 (85.2)
Duration of post-COVID condition (days)	461.5 (173.0, 700.0)	398.5 (162.0, 625.0)
Symptoms		
>5 symptoms, *n*, %	105 (84.7)	104 (85.2)
Fatigue, *n*, %	120 (96.8)	116 (95.1)
Headache, *n*, %	81 (65.3)	83 (68.0)
Shortness of breath, *n*, %	117 (94.4)	117 (95.9)
Loss of smell, *n*, %	19 (15.3)	8 (6.6)
Persistent cough, *n*, %	49 (39.5)	38 (31.1)
Sore throat, *n*, %	57 (46.0)	53 (43.4)
Fever, *n*, %	25 (20.2)	31 (25.4)
Muscle pains, *n*, %	96 (77.4)	102 (83.6)
Skipped meals, *n*, %	31 (25.0)	36 (29.5)
Chest tightness, *n*, %	104 (83.9)	105 (86.1)
Diarrhea, *n*, %	30 (24.2)	31 (25.4)
Hoarse voice, *n*, %	34 (27.4)	32 (26.2)
Abdominal pain, *n*, %	43 (34.7)	45 (36.9)
Delirium, *n*, %	89 (71.8)	85 (69.7)
Loss of consciousness, *n*, %	31 (25.0)	26 (21.3)
Palpitations (excessive tachycardia), *n*, %	81 (65.3)	76 (62.3)
Canadian Chest Pain Scale ≥II, *n*, %	94 (75.8)	93 (76.2)
NYHA ≥III, *n*, %	89 (71.8)	88 (72.1)
QoL- SF-36 - PCS	28.3 (23.9, 34.8)	30.1 (24.8, 35.2)
QoL- SF-36 - MCS	39.7 (28.8, 45.8)	38.1 (31.4, 45.3)
Blood values		
Hemoglobin (g/L)	136.5 (130.0, 145.5)	138.0 (130.0, 148.2)
Hematocrit (%)	39.8 (37.8, 42.4)	40.2 (38.1, 42.8)
Mean corpuscular volume (fL)	86.9 (84.8, 89.8)	87.8 (85.7, 90.0)
Leukocytes (abs) (pt/nl)	6.4 (5.6, 7.8)	6.5 (5.3, 7.5)
Neutrophils (abs.) (pt/nl)	4.0 (3.2, 5.2)	4.0 (3.0, 4.9)
Lymphocytes (total) (pt/nl)	1.8 (1.5, 2.2)	1.7 (1.4, 2.1)
Eosinophils (abs.) (pt/nl)	0.1 (0.1, 0.2)	0.1 (0.1, 0.2)
eGFR (MDRD; ml/min/1.73 m^2^)	103.5 (94.5, 115.8)	105.6 (97.6, 115.6)
Sodium (mmol/l)	139.0 (138.0, 140.0)	139.0 (138.0, 140.0)
Potassium (mmol/l)	4.2 (4.0, 4.4)	4.2 (4.0, 4.5)
Total cholesterol (mg/dl)	190.0 (169.0, 218.0)	187.0 (162.2, 207.0)
LDL (mg/dl)	116.2 (91.8, 138.1)	112.0 (90.0, 134.8)
HDL (mg/dl)	56.0 (46.9, 68.2)	58.3 (49.2, 72.0)
HbA1c (%)	5.1 (4.9, 5.4)	5.2 (5.0, 5.4)
D-dimer (ng/ml) (log transformed)	5.3 (5.3, 5.6)	5.3 (5.3, 5.6)
Fibrinogen (g/l) (log transformed)	1.1 (1.0, 1.2)	1.0 (0.9, 1.2)
Troponin T (ng/ml) (log transformed)	−4.6 (−5.7, 1.3)	−4.7 (−5.7, 1.4)
NT-proBNP (pg/ml) (log transformed)	3.8 (3.5, 4.2)	3.7 (3.2, 4.3)
CRP (mg/dl) (log transformed)	−2.4 (−3.2, −1.6)	−2.5 (−3.2, −1.9)
Thyroid-stimulating hormone (mU/l)	1.5 (1.1, 2.0)	1.6 (1.2, 2.1)
CMR imaging		
LVEF (%)	56.0 (54.0, 59.0)	55.5 (53.0, 57.0)
LV-EDVI (ml/m^2^)	80.7 (73.3, 86.6)	82.3 (74.7, 91.1)
LV-ESVI (ml/m^2^)	35.4 (30.5, 39.1)	36.4 (31.9, 41.9)
LVMI (g/m^2^)	40.4 (37.2, 44.9)	42.0 (38.2, 49.6)
GLS (%)	20.0 (18.9, 21.0)	19.7 (18.3, 20.9)
RVEF (%)	56.0 (53.0, 60.0)	55.0 (52.0, 59.0)
RV-EDVI (ml/m^2^)	81.8 (73.6, 90.4)	84.5 (75.4, 94.8)
RV-ESVI (ml/m^2^)	36.6 (29.8, 42.3)	36.8 (31.1, 44.3)
LA area (cm^2^)	23.0 (21.0, 25.9)	24.0 (21.0, 25.0)
RA area (cm^2^)	20.0 (17.0, 22.0)	20.9 (18.0, 22.8)
Native T1 (ms)	1140.0 (1131.0, 1152.5)	1141.0 (1131.0, 1153.0)
Native T2 (ms)	40.1 (39.0, 41.6)	39.8 (39.0, 41.3)
Perimyocardial enhancement (non-ischemic), *n*, %	82 (66.1)	72 (59.0)
Pericardial effusion, *n*, %	83 (66.9)	72 (59.0)
>1 cm, *n*, %	3 (2.4)	2 (1.6)

Pericardial effusion >1 cm defines the anatomical measurement threshold of pathophysiological relevance.

*BMI* body mass index, *BP* blood pressure, *COVID-19* coronavirus disease 2019, *eGFR* estimated glomerular filtration rate, calculated using the modification of diet in renal disease equation, *NT-proBNP* N-terminal pro-B-type natriuretic peptide, *NYHA* New York Health Association, *QoL* quality of life, physical component summary (PCS) and mental component summary (MCS) scores, *LDL* low-density lipoprotein cholesterol, *HDL* high-density lipoprotein cholesterol, *HbA1c* glycated hemoglobin A. *CRP* C-reactive protein, *LV* left ventricular, *RV* right ventricular, *LVEF* LV ejection fraction, *LV-EDVI* LV enddiastolic volume index, *LV-ESVI* LV endsystolic volume index, *GLS* global longitudinal strain, *LA* left atrium, *RA* right atrium.

Participants were normotensive adults with similar body mass index and vital signs across groups. Baseline laboratory profiles showed preserved hematologic, metabolic, and renal function. Cardiac biomarkers were normal, with maxima of troponin T of 9.2 ng/ml (intervention) and 10.0 ng/ml (placebo). Baseline CMR showed preserved biventricular systolic function and no structural heart disease, consistent with the inclusion criteria. Elevated native T1 and T2 values reflect diffuse inflammatory myocardial involvement, with non-ischemic (non-necrotic) perimyocardial contrast enhancement and small pericardial effusion (<1 cm) notable in most participants.

### Primary endpoint: change in LVEF

At W16, the mean increase in LVEF was 2.17% in the intervention group and 1.43% in the placebo group. The prespecified unpaired t-test was neutral with the between-group difference of 0.74 percentage points (95% CI, −0.14 to 1.62; *p* = 0.10). Prespecified supportive baseline-adjusted ANCOVA estimated a treatment-associated functional change (0.99 percentage points, 95% CI, 0.15–1.83; *p* = 0.021), corresponding to a relative increase of 1.69% (95% CI, 0.17–3.21; *p* = 0.030). Effect estimates were directionally consistent across analytical approaches. Exploratory subgroup analyses indicated a greater rise in LVEF in participants with baseline NT-proBNP <125 ng/L, heart rate >75 bpm, and absence of COVID-19 reinfection during follow-up. No associations were observed with age, sex, symptom duration, vaccination status, or antidepressant use.

### Secondary clinical and biomarker endpoints

**Fig. 2:**
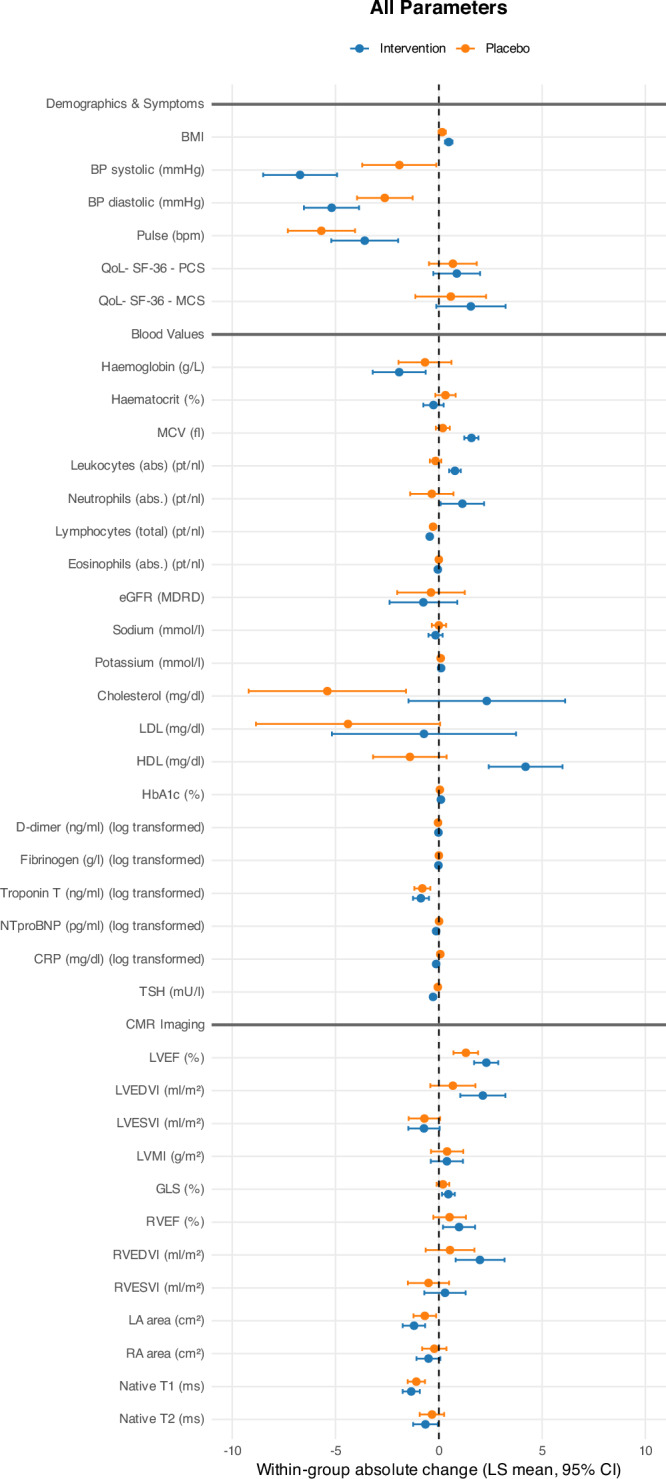
Least-squares mean changes in clinical, laboratory, and imaging parameters. Forest plot displaying least-squares mean absolute changes (95% confidence intervals) from baseline to week 16 for vital signs, quality-of-life measures, and blood biomarkers and cardiovascular magnetic resonance (CMR) parameters, comparing intervention and placebo groups. Each row reports a parameter; the two points per row represent the within-group least-squares mean absolute change for the intervention group (blue) and the placebo group (orange), with horizontal bars indicating two-sided 95% confidence intervals. Estimates are derived from the modified intention-to-treat population (*n* = 246 participants with paired baseline and week-16 assessments; intervention *n* = 124, placebo *n* = 122).

The proportion of participants reporting >5 symptoms decreased by 7.7 percentage points (95% CI −18.9 to 3.6), and fatigue decreased by 6.3 percentage points (95% CI −14.0 to 1.3) in the intervention group compared with placebo (Supplementary Tables S5 and S6). There was no change in QoL with intervention. Systolic and diastolic blood pressure decreased more in the intervention group. A small increase in body mass index accompanied these changes. Lymphocyte and eosinophil counts, C-reactive protein and thyroid-stimulating hormone were lower with intervention, while mean corpuscular volume, total cholesterol, high-density lipoprotein and glycated hemoglobin A1 were higher. Both groups showed decreases in D-dimer and troponin T and increases in serum potassium. Renal function remained stable. Native T1 values decreased in both groups. Additional changes with intervention included increased left and right end-diastolic ventricular volumes and global longitudinal strain, and decreased native T2, left atrial size, and proportion of perimyocardial contrast enhancement. The between-group changes in categorical symptom outcomes and continuous clinical, biomarker and imaging parameters are summarized in Tables 2 and 3, with the per-protocol analysis of continuous variables in Supplementary Table S4, and presented in Fig. 2.

**Table 2:** Between-group differences and changes from baseline to week 16 in demographic, clinical, and cardiovascular magnetic resonance (CMR) characteristics (intention-to-treat population)

Parameter	Intervention *n*(%)	Placebo *n*(%)	Absolute difference *n*(%)
Symptoms			
>5 symptoms	84 (67.7)	92 (75.4)	−8 (−7.7)
Fatigue	107 (86.3)	113 (92.6)	−6 (−6.3%)
Headache	74 (59.7)	73 (59.8)	+1 (−0.1)
Shortness of breath	103 (83.1)	101 (82.8)	+2 (+0.3)
Loss of smell	15 (12.1)	9 (7.4)	+6 (+4.7)
Persistent cough	38 (30.6)	30 (24.6)	+8 (+6.0)
Sore throat	48 (38.7)	60 (49.2)	−12 (−10.5)
Fever	18 (14.5)	21 (17.2)	−3 (−2.7)
Muscle pains	80 (64.5)	89 (73.0)	−9 (−8.5)
Skipped meals	25 (20.2)	30 (24.6)	−5 (−4.4)
Chest tightness	79 (63.7)	78 (63.9)	+1 (−0.2)
Diarrhea	27 (21.8)	27 (22.1)	0 (−0.3)
Hoarse voice	35 (28.2)	31 (25.4)	+4 (+2.8)
Abdominal pain	34 (27.4)	48 (39.3)	−14 (−11.9)
Delirium	91 (73.4)	86 (70.5)	+5 (+2.9)
Loss of consciousness	7 (5.6)	8 (6.6)	−1 (−1.0)
Palpitations (excessive tachycardia)	61 (49.2)	54 (44.3)	+7 (+4.9)
Canadian Chest Pain Scale ≥II	54 (43.5)	59 (48.4)	−5 (−4.9)
NYHA≥III	57 (46.0)	66 (54.1)	−9 (−8.1)
Perimyocardial enhancement (non-ischemic)	62 (50.0)	65 (53.3)	−3 (−3.3)
Pericardial effusion	86 (69.4)	81 (66.4)	+5 (+3.0)
>1 cm	4 (3.2)	1 (0.8)	+3 (+2.4)

Abbreviations as in Table 1.

**Table 3:** Between-group differences and least-squares mean changes from baseline to week 16 in demographic, clinical, and cardiovascular magnetic resonance (CMR) characteristics—continuous variables (intention-to-treat population)

	W16 absolute change			W16 percentage change		
Parameter	Intervention LS means (95% CI)	Placebo LS means (95% CI),	Difference LS means (95% CI)	Intervention LS means (95% CI)	Placebo LS means (95% CI)	Difference, LS means (95% CI)
Vital signs						
BMI (kg/m^2^)	0.48 (0.30–0.66)	0.16 (−0.01 to 0.34)	0.32 (0.07–0.57)	1.84 (1.14–2.54)	0.70 (−0.00 to 1.40)	1.14 (0.15–2.13)
BP systolic (mmHg)	−6.72 (−8.51 to −4.93)	−1.91 (−3.71 to −0.12)	−4.81 (−7.35 to −2.27)	−4.95 (−6.39 to −3.51)	−1.14 (−2.59 to 0.31)	−3.81 (−5.86 to −1.76)
BP diastolic (mmHg)	−5.19 (−6.53 to −3.86)	−2.62 (−3.96 to −1.27)	−2.58 (−4.47 to −0.68)	−5.71 (−7.41 to −4.00)	−2.66 (−4.37 to −0.94)	−3.05 (−5.47 to −0.63)
Heart rate (bpm)	−3.59 (−5.21 to −1.97)	−5.69 (−7.31 to −4.06)	2.10 (−0.21 to 4.40)	−3.10 (−5.31 to −0.89)	−6.29 (−8.50 to −4.07)	3.19 (0.05–6.32)
QoL- SF-36 - PCS	0.87 (−0.27 to 2.00)	0.68 (−0.48 to 1.84)	0.19 (−1.44 to 1.81)	3.91 (0.07–7.75)	4.24 (0.30–8.18)	−0.33 (−5.83 to 5.17)
QoL- SF-36 - MCS	1.55 (−0.12 to 3.23)	0.58 (−1.14 to 2.29)	0.97 (−1.42 to 3.37)	9.95 (3.34–16.55)	10.22 (3.45–17.00)	−0.28 (−9.74 to 9.19)
Blood values						
Hemoglobin (g/L)	−1.92 (−3.20 to −0.64)	−0.67 (−1.95 to 0.61)	−1.25 (−3.06 to 0.57)	−1.24 (−2.15 to −0.33)	−0.36 (−1.27 to 0.56)	−0.88 (−2.18 to 0.41)
Hematocrit (%)	−0.26 (−0.75 to 0.23)	0.32 (−0.17 to 0.81)	−0.57 (−1.27 to 0.12)	−6.62 (−98.67 to 85.44)	90.41 (−1.65 to 182.46)	−97.02 (−227.21 to 33.17)
Mean corpuscular volume (fL)	1.58 (1.23–1.92)	0.19 (−0.15 to 0.53)	1.39 (0.90–1.87)	1.85 (1.45–2.24)	0.24 (−0.16 to 0.64)	1.61 (1.05–2.18)
Leukocytes (abs) (pt/nl)	0.78 (0.50–1.06)	−0.16 (−0.44 to 0.12)	0.94 (0.55–1.33)	13.47 (9.32–17.63)	0.06 (−4.10 to 4.21)	13.42 (7.54–19.29)
Neutrophils (abs.) (pt/nl)	1.14 (0.09–2.19)	−0.34 (−1.39 to 0.71)	1.48 (−0.00 to 2.97)	42.21 (14.50–69.92)	12.03 (−15.56 to 39.63)	30.18 (−8.99 to 69.34)
Lymphocytes (total) (pt/nl)	−0.44 (−0.54 to −0.34)	−0.28 (−0.38 to −0.18)	−0.16 (−0.31 to −0.02)	−9.55 (−14.03 to −5.07)	2.12 (−2.3 6 to 6.60)	−11.67 (−18.01 to −5.32)
Eosinophils (abs.) (pt/nl)	−0.05 (−0.06 to −0.03)	0.00 (−0.01 to 0.02)	−0.05 (−0.08 to −0.03)	−11.51 (−37.16 to 14.15)	45.86 (20.10–71.63)	−57.37 (−93.73 to −21.01)
eGFR (ml/min/1.73)	−0.75 (−2.39 to 0.89)	−0.38 (−2.02 to 1.26)	−0.37 (−2.69 to 1.95)	−0.22 (−1.85 to 1.41)	−0.32 (−1.95 to 1.32)	0.09 (−2.22 to 2.41)
Sodium (mmol/l)	−0.16 (−0.50 to 0.18)	0.00 (−0.34 to 0.35)	−0.16 (−0.65 to 0.32)	−0.10 (−0.35 to 0.15)	0.02 (−0.23 to 0.27)	−0.12 (−0.47 to 0.23)
Potassium (mmol/l)	0.11 (0.04–0.17)	0.09 (0.03–0.16)	0.02 (−0.08 to 0.11)	2.89 (1.37–4.40)	2.49 (0.95–4.03)	0.40 (−1.76 to 2.56)
Cholesterol (mg/dl)	2.32 (−1.47 to 6.12)	−5.40 (−9.21 to −1.59)	7.72 (2.34–13.11)	1.87 (−0.13 to 3.87)	−2.04 (−4.05 to −0.04)	3.91 (1.07–6.75)
LDL (mg/dl)	−0.72 (−5.18 to 3.74)	−4.40 (−8.85 to 0.06)	3.68 (−2.64 to 9.99)	65.28 (−67.92 to 198.48)	103.42 (−29.78 to 236.62)	−38.13 (−226.72 to 150.45)
HDL (mg/dl)	4.20 (2.42–5.99)	−1.40 (−3.19 to 0.38)	5.61 (3.08–8.14)	8.30 (5.74–10.85)	−0.77 (−3.33 to 1.78)	9.07 (5.45-12.69)
HbA1c (%)	0.10 (0.07–0.14)	0.05 (0.01–0.08)	0.06 (0.01–0.10)	2.06 (1.42–2.71)	0.97 (0.31–1.63)	1.09 (0.17–2.01)
D-dimer (ng/ml) (log transformed)	−0.02 (−0.07 to 0.03)	−0.04 (−0.09 to 0.02)	0.02 (−0.06 to 0.09)	−0.96 (−5.16 to 3.23)	−4.44 (−8.69 to −0.19)	3.48 (−2.50 to 9.45)
Fibrinogen (g/l) (log transformed)	−0.02 (−0.05 to 0.00)	0.00 (−0.03 to 0.03)	−0.02 (−0.06 to 0.02)	−1.36 (−4.20 to 1.47)	1.43 (−1.44 to 4.31)	−2.80 (−6.84 to 1.24)
Troponin T (ng/ml) (log transformed)	−0.87 (−1.25 to −0.48)	−0.80 (−1.19 to −0.41)	−0.07 (−0.62 to 0.48)	−70.58 (−100.81 to −40.35)	−62.47 (−92.70 to −32.24)	−8.12 (−50.87 to 34.63)
NT-proBNP (pg/ml) (log transformed)	−0.13 (−0.25 to −0.01)	0.01 (−0.11 to 0.13)	−0.14 (−0.31 to 0.03)	−2.28 (−6.24 to 1.69)	2.74 (−1.24 to 6.73)	−5.02 (−10.64 to 0.60)
CRP (mg/dl) (log transformed)	−0.13 (−0.26 to 0.01)	0.07 (−0.07 to 0.20)	−0.20 (−0.39 to −0.01)	27.50 (−10.93 to 65.93)	−2.91 (−41.67 to 35.84)	30.41 (−24.19 to 85.02)
Thyroid- stimulating hormone (mU/l)	−0.28 (−0.40 to −0.16)	−0.05 (−0.17 to 0.07)	−0.22 (−0.39 to −0.06)	−17.89 (−131.36 to 95.58)	91.78 (−22.17 to 205.72)	−109.66 (−270.73 to 51.41)
CMR imaging						
LVEF (%)	2.30 (1.71–2.88)	1.31 (0.71–1.90)	0.99 (0.15–1.83)	4.22 (3.15–5.28)	2.53 (1.45–3.60)	1.69 (0.17–3.21)
LVEDVI (ml/m²)	2.13 (1.04–3.22)	0.68 (−0.41 to 1.77)	1.45 (−0.09 to 3.00)	2.84 (1.41–4.27)	1.37 (−0.05 to 2.80)	1.47 (−0.56 to 3.49)
LVESVI (ml/m^2^)	−0.72 (−1.48 to 0.04)	−0.70 (−1.46 to 0.06)	−0.02 (−1.10 to 1.06)	−1.68 (−3.85 to 0.48)	−0.92 (−3.09 to 1.25)	−0.77 (−3.85 to 2.31)
LV mass index (g/m^2^)	0.39 (−0.39 to 1.17)	0.40 (−0.38 to 1.18)	−0.02 (−1.13 to 1.10)	4.33 (−3.66 to 12.33)	3.37 (−4.59 to 11.33)	0.96 (−10.40 to 12.32)
GLS (%)	0.46 (0.15–0.77)	0.20 (−0.11 to 0.51)	0.26 (−0.18 to 0.71)	2.66 (1.04–4.28)	1.67 (0.04–3.31)	0.99 (−1.32 to 3.30)
RVEF (%)	0.98 (0.20–1.76)	0.52 (−0.27 to 1.31)	0.46 (−0.65 to 1.57)	2.11 (0.69–3.52)	1.37 (−0.06 to 2.79)	0.74 (−1.27 to 2.75)
RVEDVI (ml/m^2^)	1.99 (0.81–3.18)	0.54 (−0.64 to 1.72)	1.46 (−0.22 to 3.13)	2.83 (1.38–4.28)	1.30 (−0.16 to 2.75)	1.53 (−0.52 to 3.59)
RVESVI (ml/m^2^)	0.32 (−0.67 to 1.31)	−0.52 (−1.51 to 0.47)	0.84 (−0.56 to 2.25)	7.36 (−3.45 to 18.16)	1.58 (−9.22 to 12.38)	5.78 (−9.56 to 21.12)
LA area (cm^2^)	−1.20 (−1.75 to −0.66)	−0.68 (−1.23 to −0.13)	−0.53 (−1.30 to 0.25)	−3.15 (−5.45 to −0.86)	−1.55 (−3.88 to 0.79)	−1.61 (−4.88 to 1.67)
RA area (cm^2^)	−0.50 (−1.08 to 0.07)	−0.22 (−0.81 to 0.37)	−0.29 (−1.11 to 0.54)	0.94 (−1.38 to 3.27)	−0.19 (−2.57 to 2.19)	1.13 (−2.20 to 4.46)
Native T1 (ms)	−13.37 (−17.51 to −9.23)	−10.91 (−15.09 to −6.73)	−2.46 (−8.35 to 3.42)	−1.15 (−1.51 to −0.79)	−0.93 (−1.29 to −0.56)	−0.22 (−0.73 to 0.29)
Native T2 (ms)	−0.65 (−1.24 to −0.05)	−0.33 (−0.93 to 0.26)	−0.31 (−1.16 to 0.53)	−4.09 (−14.55 to 6.37)	−12.22 (−22.77 to −1.67)	8.13 (−6.74 to 23.00)

Continuous variables are presented as least-squares mean changes from baseline, estimated using analysis of covariance (ANCOVA) models including treatment group as a fixed factor and the baseline value of the respective parameter as a covariate. Between-group differences reflect adjusted treatment effects. Absolute change values reflect the prespecified primary outcome metric, whereas percentage change values are presented descriptively to facilitate cross-parameter comparison. Abbreviations as in Table 1.

### Safety, compliance, and tolerability

A total of 185 adverse events (AEs) were recorded, including 9 serious AEs (see Supplementary Tables S7–S10). The AEs incidence was similar between groups, and no excess of serious AEs occurred in the intervention group. Among the 9 serious AEs, 4 required hospitalization and 5 were classified as important medical events; none involved prespecified safety concerns, such as syncope, septic shock, biomarker elevation, renal impairment or hemodynamic instability. All participants with serious AEs recovered without sequelae. Treatment compliance was high in both groups. Overall, 236 participants (96%) reached the target losartan dose of 50 mg, with no significant difference between groups. Treatment adjustments due to AEs were similar between the groups.

## Discussion

In this randomized, double-blind, placebo-controlled clinical trial of individuals with post-COVID syndrome and cardiac inflammation confirmed by CMR and without structural heart disease, 16 weeks of losartan plus low-dose prednisolone, the prespecified confirmatory unpaired t-test did not meet the significance threshold. The supportive prespecified ANCOVA estimated a modest treatment-associated functional difference by accounting for baseline variability and reducing residual variance. As the confirmatory primary analysis was neutral, all mechanistic interpretation is exploratory and contextual. The magnitude of improvement was consistent with the biological hypothesis and with the anticipated small-to-moderate effect size within a short interventional period. Secondary endpoints showed greater reductions in blood pressure and inflammatory biomarkers with intervention, and a higher proportion of participants reported improvement in overall symptom burden and fatigue. Treatment was well-tolerated, with similar AE rates across groups and high adherence. These findings provide physiological and analytical insight into targeted cardioprotective immunomodulation in a defined post-COVID inflammatory cardiac endotype, highlight the utility of standardized CMR for phenotypic enrichment and objective assessment of treatment response, and establish a framework to inform the design of future confirmatory trials.

The combined use of unadjusted confirmatory and baseline-adjusted supportive analyses reflects the exploratory therapeutic context of a heterogeneous post-COVID population without validated clinical endpoints. The confirmatory primary analysis was neutral and therefore does not, on its own, support a mechanistic interpretation. Functional findings are instead considered within the broader physiological and imaging context. Within this analytical framework, supportive baseline-adjusted ANCOVA analyses estimated a modest treatment-associated functional difference with biological plausibility. The observed increase in LVEF over 16 weeks is directionally consistent with the mechanistic hypothesis that attenuation of low-grade cardiac inflammation may translate into functional improvement in post-COVID condition. These functional changes are interpreted alongside concurrent improvements in myocardial tissue characteristics and inflammatory biomarker findings, supporting a targeted cardioprotective immunomodulatory strategy, with losartan enhancing endothelial function and prednisolone reducing low-grade myocardial inflammation, in a condition without validated clinical endpoints or established therapies^29,30^. Both agents were repurposed for their anti-inflammatory and cardioprotective properties, and well-characterized efficacy and safety profiles. The use of CMR-derived LVEF as the primary endpoint provided a sensitive, quantitative, and reproducible surrogate marker, enabling assessment of myocardial functional change within this endotype-defined population.

Participants were selected for objective evidence of persistent inflammatory cardiac involvement, including elevated native T1 or T2 mapping, or non-ischemic perimyocardial contrast enhancement or LVEF 45–50%, while carefully excluding those with known antecedent cardiac disease, in whom the effects of losartan are already established^23,24,26,31^. The impact of prednisolone on systolic function is less well defined^27,32^. Overall, participants exhibited preserved systolic function and did not meet criteria for heart failure; therefore, no evidence base exists to guide therapy in individuals with LVEF >45%. Prior observational data suggest no spontaneous improvement in LVEF over time in this post-COVID endotype, supporting its role as a disease-defining functional marker rather than a manifestation of structural heart failure^3,19^. Within this context, observed changes in LVEF may reflect functional modulation of inflammatory myocardial dysfunction rather than modification of an established heart failure substrate. Subgroup analyses suggested a greater benefit in participants with normal NT-proBNP, higher baseline heart rate and absence of reinfection, patterns that may align with mechanisms involving improved vascular compliance, reduced sympathoadrenal activation and attenuation of post-infectious inflammatory effects.

Left ventricular end-diastolic volume increased only modestly with intervention, with similar directional changes across the groups. This observation likely reflects the combined pharmacologic profile of the investigational regimen; whereas losartan may promote modest intravascular volume contraction, concomitant low-dose prednisolone can exert countervailing effects through sodium and fluid retention. The observed increase in LVEF is unlikely to be attributable solely to preload effects, as ventricular volumes remained descriptively stable. Although blood pressure reduction may have modified afterload, the overall pattern suggests that loading conditions alone do not fully account for the LVEF signal. The lower total cholesterol in the placebo group appeared partly driven by an increase in HDL in the intervention arm, while LDL remained largely unchanged. Although exploratory, this directionality is consistent with reported pleiotropic metabolic effects of losartan^33^; therefore, this observation may be validated by future studies.

A central feature of the trial design was rigorous phenotypic selection, whereby the effects of the cardioprotective immunomodulatory intervention on post-COVID-19 cardiac inflammation could be selectively and specifically assessed. The prespecified Myoflame-19 CMR criteria were based on validated tissue mapping cutoffs and non-ischemic perimyocardial contrast enhancement patterns derived from prior studies, using identical acquisition and analysis methodology in post-COVID and other contexts of postviral syndromes and autoimmune inflammatory cardiomyopathy^34^. Contrast-enhanced imaging by CMR facilitated the visualization of non-necrotic perimyocardial inflammatory changes, a recognized feature of immune-mediated cardiac involvement^34,35^. The sensitivity of tissue parametric mapping for diffuse inflammatory cardiomyopathies was enhanced by standardized acquisition platforms and predefined quantitative thresholds, ensuring that each marker contributed independent mechanistic information. Within this endotype-defined framework, the intervention was associated with directional improvements in myocardial tissue markers and functional indices, consistent with the cardioprotective immunomodulatory hypothesis in post-COVID pathophysiology. Yet the persistence of inflammatory CMR abnormalities was paralleled by ongoing effort intolerance, consistent with a sustained inflammatory cardiac phenotype, which is unlikely to resolve spontaneously or with short-term interventions, potentially requiring targeted, long-term therapy.

The heterogeneous symptom profile of post-COVID syndrome remains a clinical challenge. Although all participants had CMR evidence of cardiac inflammation, most also exhibited multisystem symptoms, prolonged illness duration and substantial functional limitation typical of contemporary post-COVID populations. Improvements in fatigue and overall symptom burden were greater in the direction of the intervention. Structured pacing guidance in line with standard supportive care recommendations^4^ to avoid relapses of effort intolerance with activity in both arms may have contributed to the improvement in both groups. Adherence was not formally quantified; symptom endpoints were exploratory without multiplicity adjustment. Despite symptom improvements, parallel QoL gains were not observed, likely reflecting the short 16-week timeframe, the multidimensional determinants of QoL beyond cardiac physiology, as well as highlighting the gap between partial physiological recovery and patient expectations, relative to the pre-illness baseline. As symptom trajectories may not fully reflect underlying physiological recovery, objective measures, such as LVEF, may be valuable for determining the direction of early improvement in cardiac function and inflammatory markers, independent of symptom duration. Several clinical trials in post-COVID syndrome have evaluated antivirals, statins, anticoagulation, sodium-glucose transporter 2 inhibitors, and angiotensin-II and endothelin receptor antagonists^30^, with limited or no consistent evidence of clinical benefit to date. Key distinguishing features of the present trial include CMR-based phenotypic enrichment, exclusion of participants with antecedent cardiac disease, and a specifically designed cardioprotective immunomodulatory strategy targeting inflammation-induced myocardial dysfunction. The use of CMR-derived LVEF as a prespecified primary efficacy endpoint enabled quantitative assessment of treatment response within a moderate sample size. Collectively, these design elements emphasize the value of imaging-guided selection in reducing pathophysiological heterogeneity and identifying treatment-responsive subgroups.

A few limitations apply. The cohort was predominantly Caucasian, and validation in more diverse populations is required. The trial was powered for a physiological endpoint rather than patient-reported outcomes. Paired CMR completion was 88%, corresponding to a 12 % non-evaluable rate for the primary endpoint, higher than the 8% allowance used in sample-size planning. A subset of participants did not proceed with therapeutic participation following eligibility confirmation. Excluding these cases, attrition approximated the prespecified estimate. The primary endpoint was analyzed using a complete-case approach restricted to participants with paired baseline and follow-up CMR. Attrition was balanced between treatment groups and therefore unlikely to influence between-group comparisons. Although the confirmatory analysis was neutral, supportive baseline-adjusted modeling provided contextual insight relevant to analytical planning for future confirmatory studies. Randomized trials in post-COVID condition remain challenging owing to population heterogeneity, evolving pathophysiology, and the absence of validated endpoints; within this landscape, the present trial contributes methodological and mechanistic foundations for subsequent investigations. The modest absolute LVEF effect size should be interpreted within the preserved baseline systolic function of the cohort, which inherently constrains the dynamic range for measurable improvement over a short intervention period. Targeting inflammatory myocardial dysfunction in individuals without structural heart disease represents a pathophysiologically ambitious strategy in a population with no established therapeutic framework. Advanced CMR imaging may limit generalizability to settings without access to standardized tissue mapping and core laboratory analysis. Secondary endpoints were prespecified as exploratory. Durability of functional and tissue responses beyond 16 weeks was not assessed and requires future evaluation.

In conclusion, 16 weeks of combined losartan and low-dose prednisolone was associated with a modest treatment-associated difference in LVEF compared with placebo in individuals with post-COVID syndrome and CMR-defined cardiac inflammation. These findings provide pathophysiological and analytical insight into cardioprotective immunomodulation as a therapeutic strategy. They establish a framework to inform the design and powering of future confirmatory studies.

## Methods

### Study design and participants

We conducted an international, multicenter, randomized, double-blind, placebo-controlled trial investigating combined cardioprotective and immunomodulatory therapy in individuals with post-COVID inflammatory cardiac involvement^1^. The investigator-initiated study was sponsored by Goethe University Frankfurt, Germany, with financial support from Bayer AG and the German Centre for Cardiovascular Research (DZHK). Trial operations, data management, monitoring and statistical analyses were performed by an independent clinical research organization. Oversight was provided by independent steering and data monitoring committees. The trial is reported in accordance with the CONSORT 2025 guidelines (Supplementary Note 3). The first participant was enrolled on 14 December 2022, and the last on 30 March 2025.

The protocol was approved by the Ethics Committee of University Hospital Frankfurt, local ethics committees and by the competent authorities (BfArM and BASG). The trial is registered with the European Clinical Trial Information System (CTIS, EudraCT 2022-001682-12) and ClinicalTrials.gov (NCT05619653). Two authors had full access to the data and verified the accuracy and completeness. The full study protocol and statistical analysis plan have been published previously^36^ and are provided in the Supplementary Information (Supplementary Note 1, study protocol; Supplementary Note 2, statistical analysis plan).

### Participants

Candidates registered through a study website and were screened against predefined inclusion and exclusion criteria. Eligible participants were ≥18 years of age, had documented COVID-19 infection ≥28 days before enrolment and reported persistent or new-onset cardiac symptoms, not present before infection^1^. All participants were required to meet the prespecified CMR criteria for inflammatory cardiac involvement, defined as abnormal native T1 or T2 mapping, non-ischemic perimyocardial contrast enhancement, or LVEF ≤50%.

Exclusion criteria included known structural heart disease, baseline LVEF ≤44%, conditions requiring guideline-directed cardiac therapy, major comorbidities, prior immunosuppression and contraindications to study drugs or contrast-enhanced CMR. Symptomatic hypotension (systolic blood pressure <90 mm Hg) unresponsive to oral hydration was an exclusion criterion, as was any prior or current use of any guideline-directed cardiac therapy (renin-angiotensin-II aldosterone system blockers^23^, neprilysin inhibitors, vericiguat, sodium-glucose transport 2 inhibitors, diuretics, or antihypertensive treatment). Use of oral prednisolone or other immunosuppressive/biological agents within 10 weeks prior to enrollment was an exclusion criterion. Further exclusions included alcohol, drug, or chemical abuse; participation in another investigational study; or inability to provide written informed consent. Female-specific exclusions included pregnancy, breastfeeding, or childbearing potential without highly effective contraception. All participants provided written informed consent.

### Trial procedures

Demographics, vital signs, symptom burden, concomitant medications, and study medication use were recorded at baseline and follow-up visits. Participants underwent standardized CMR imaging at baseline and week 16 using clinical 3.0-Tesla scanners (Siemens Healthcare, Syngo MR XA60A) with harmonized software versions. The imaging protocol included standardized short-axis and long-axis cine imaging, prespecified sequences with harmonized parameters for T1 and T2 mapping and contrast enhancement imaging approximately 10 min after administration of gadobutrol (Gadovist, 0.1 mmol/kg, Bayer Healthcare). Myocardial perfusion imaging with the vasodilator regadenoson (Rapiscan, GE Healthcare; 400 μg/5 ml) was performed to exclude significant coronary artery disease.

All imaging procedures were prespecified in standard operating procedures, supported by structured training and ongoing quality assurance. De-identified CMR datasets were uploaded into the electronic case report form and analyzed centrally, blinded to clinical data and treatment allocation, using semi-automated software (suiteHEART, NeoSoft LLC, USA).

### Randomization and masking

Participants were randomly assigned in a 1:1 ratio to receive losartan plus prednisolone or matching placebos for 16 weeks. Losartan was initiated at 12.5 mg once daily, taken in the evening, and uptitrated to 50 mg as tolerated. Prednisolone was started at 20 mg once daily in the morning and tapered to a maintenance daily dose of 5 mg. Participants received standardized guidance on lifestyle management, including structured pacing, in line with standard guidelines^36,37^, and hydration strategies to minimize symptoms of hypotension.

### Safety and compliance

Safety and compliance were assessed during scheduled remote and in-person visits. Each participant was provided with a certified BP monitor and an electronic patient diary to record daily BP and HR. Remote follow-up reviews of symptoms, BP diary, safety, tolerance, and compliance were conducted at weeks 2, 6, and 12. Week 6 assessment included an on-site visit (or with local doctors) for a 12-channel ECG and routine blood tests.

### Outcomes

The primary efficacy endpoint was the absolute change in LVEF from baseline to 16 weeks, measured by CMR. Secondary endpoints included in the present analysis were changes in symptom scores, functional class, blood biomarkers, CMR parameters and safety outcomes. The latter included AEs, serious AEs, treatment adherence and tolerance. The safety population comprised all participants who received ≥1 dose of study medication.

### Statistical analysis

Statistical analyses were performed using SAS 9.4 (SAS Institute, Cary, NC, USA) by independent statisticians with full access to the data. Normality of data distribution was assessed using the Shapiro–Wilk test. Baseline characteristics are presented as medians with interquartile ranges (IQRs) for continuous variables and counts (%) for categorical variables.

The prespecified confirmatory primary analysis compared absolute change in LVEF between treatment groups using an unpaired t-test; a prespecified baseline-adjusted ANCOVA incorporating baseline LVEF as a covariate was performed as a supportive analysis to account for baseline variability and reduce residual variance in estimating treatment-associated functional change. A two-sided *α* level of 0.05 was applied to the primary analysis.

Absolute change from baseline to week 16 was prespecified as the primary efficacy metric for LVEF, reflecting the direct physiological difference in ventricular function over time. Percentage change was additionally calculated for selected secondary parameters to facilitate comparative interpretation across variables with differing baseline scales and units. Percentage change metrics were used descriptively and were not employed for confirmatory hypothesis testing.

Secondary endpoints were analyzed descriptively with summary statistics by treatment group and visit. Continuous secondary outcomes were analyzed using baseline-adjusted ANCOVA models. Categorical secondary outcomes are presented descriptively as counts and percentages at each time point, with absolute between-group differences where appropriate; no formal hypothesis testing was undertaken for these exploratory endpoints, and no adjustments for multiple comparisons were applied. Secondary endpoints included changes from baseline in symptom scores (Long COVID Questionnaire, modified Canadian Chest Pain Scale, New York Heart Association functional class and SF-36 RAND quality-of-life questionnaire), blood biomarkers and CMR measures of cardiac structure and function (T1 and T2 mapping, LV volumes and mass, myocardial strain and perimyocardial contrast enhancement). The additional prespecified secondary outcomes (contrast enhancement extent, aortic imaging, and exercise tolerance testing) will be reported in future dedicated analyses.

Safety endpoints included AEs, compliance and treatment tolerance. Tolerance was assessed by the frequency of dose reductions or treatment discontinuations due to side effects. The cumulative prednisolone dose was calculated. Treatment response by “restitutio ad integrum” was defined as a normal CMR result, including sequence-specific T1 and T2 values within normal ranges, sex- and age-adjusted normal LVEF, non-dilated LV and absence of contrast enhancement.

Primary efficacy analyses were conducted in participants with paired baseline and week-16 CMR data (complete-case analysis). Individuals without evaluable follow-up imaging were not included in efficacy analyses, and no imputation procedures were applied. Sample size calculations are provided in the Supplementary Information. All primary and secondary efficacy analyses were conducted in both intention-to-treat and per-protocol populations.

### Reporting summary

Further information on research design is available in the Nature Portfolio Reporting Summary linked to this article.

## Supplementary information

Supplementary Information

Reporting Summary

Transparent Peer Review File

## Data Availability

MYOFLAME-19 was conducted in accordance with ICH E6 Good Clinical Practice, including the applicable ICH E6(R2) requirements and, where applicable, current ICH E6(R3) principles, EU Regulation 536/2014, and the General Data Protection Regulation (EU) 2016/679. Trial data are held by the independent clinical research organisation responsible for data management; two authors (C.W.K. and S.E.) had full access to the complete dataset and verified the accuracy and fidelity of the analyses. Under the existing regulatory approvals and participant consent, the authors are permitted to share summary-level and aggregate data but are not permitted to release individual-participant data. In addition, trial documentation identifies a residual risk of re-identification for participant-level data from this limited cohort, including pseudonymised or partially de-identified datasets; raw cardiovascular magnetic resonance (CMR) images (DICOM) are particularly susceptible to re-identification. Summary-level data supporting the primary and secondary findings are available in the published Article and Supplementary Information. The full trial protocol and statistical analysis plan are provided as Supplementary Notes. Availability differs by data type: summary-level and aggregate data are available as described above; individual-participant data, participant-level derived datasets, and raw cardiovascular magnetic resonance (CMR) images are not available under the current regulatory and ethics approvals. Requests concerning individual-participant data may be directed to the corresponding author (puntmann(at)med.uni-frankfurt.de) or trial sponsor (research-support(at)uni-frankfurt.de), as applicable. Any future data access would require the establishment of an appropriate regulatory, ethics, and data-protection framework, including renewed approvals where required.

## Code availability

No custom code was used for data processing or analysis. All statistical analyses were performed using standard procedures implemented in SAS version 9.4.
