# Supplementary material for: Losartan and prednisolone for post-COVID syndrome and cardiac inflammation: a randomized, double-blind, placebo-controlled trial
Source: Nat Commun. 2026 Jul 30;17:7599. doi: 10.1038/s41467-026-75991-w (PMC13424660; doi:10.1038/s41467-026-75991-w)
Supplement: Supplementary file 1 — Supplementary Information [file 41467_2026_75991_MOESM1_ESM.pdf]

Myoflame-19 Trial - Supplementary Appendix

Title Page

Full Title: Losartan and Prednisolone for Post-COVID Syndrome and Cardiac Inflammation: A Randomized, Double-Blind, Placebo-Controlled Trial

1 Supplementary Methods

All trial procedures and endpoint definitions were prespecified in the protocol (Version 3.1) and SAP (Version 1.0).

1.1 Lifestyle Guidance

All participants received standardized written and verbal guidance on pacing strategies and hydration practices at study initiation, reflecting contemporary supportive management for post-COVID syndromes. Guidance included energy conservation, graded activity pacing, avoidance of post-exertional symptom exacerbation, and maintenance of adequate fluid intake. Counselling was harmonized across sites through site-initiation visit supported by the CRO. Adherence to pacing or hydration recommendations was not formally quantified.

1.2 Sample size

We estimated a treatment difference in mean LVEF change of 2–4% with a standard deviation of 9% and a 1:1 allocation ratio, corresponding to an effect size (Cohen's d) of 0.22–0.44, i.e., a small to moderate effect. Assuming a Cohen's d of 0.35, a two-sided α of 5%, and 80% power, 130 patients per group were required. Allowing for an 8% dropout rate, a total of 280 patients needed to be randomized. A screening failure rate of approximately 50% was anticipated due to preexisting cardiovascular disease or comorbidities, or prior medication. LVEF, a validated surrogate outcome measure of efficacy and a standard measure of cardiac performance across imaging modalities, was selected as the primary endpoint; CMR was used as the gold-standard technique owing to the accuracy of volumetric analyses and the ability to derive highly standardized, objective measures.

1.3 Population definitions

The intention-to-treat population corresponded to a modified ITT (full analysis set) and included all randomized participants who underwent baseline and week-16 CMR assessments and received at least 4 weeks of study treatment. The per-protocol population included participants who completed baseline and week-16 CMR assessments, received ≥75% study-drug compliance, and had no major protocol deviations as prespecified in the statistical analysis plan."

1.4 Safety analyses

Safety was assessed in the safety set (SAF), defined as all participants who received at least one dose of IMPs. Separate safety analyses were performed for contrast agent exposure in all participants who received at least one dose of gadobutrol. The AEs, categorized as "special situations", were reported separately. The AEs were coded using MedDRA preferred term to system organ class (SOC) independently by the CRO's pharmacovigilance team using standardized procedures.

1.5 List of Abbreviations

AE — Adverse event

ANCOVA — Analysis of covariance

BfArM — Bundesinstitut für Arzneimittel und Medizinprodukte (Federal Institute for Drugs and Medical Devices, Germany)

BL — baseline

BMI — Body mass index

BP — Blood pressure

BASG — Austrian Federal Office for Safety in Health Care

CMR — Cardiovascular magnetic resonance

CI — Confidence interval

CRP — C-reactive protein

DZHK — Deutsches Zentrum für Herz-Kreislauf-Forschung (German Centre for Cardiovascular Research)

ECG — Electrocardiogram

EF — Ejection fraction

eGFR — Estimated glomerular filtration rate

FAS — Full analysis set

HbA1c — Hemoglobin A1c

HDL — High-density lipoprotein

HR — Heart rate

IMP — Investigational medicinal product

IQR — Interquartile range

ITT — Intention-to-treat

LA — Left atrium / Left atrial

LV — Left ventricle / Left ventricular

LVEDVI — Left ventricular end-diastolic volume index

LVEF — Left ventricular ejection fraction

LVESVI — Left ventricular end-systolic volume index

LVMI — Left ventricular mass index

MedDRA — Medical Dictionary for Regulatory Activities

MCV — Mean corpuscular volume

MCS — Mental component summary (from SF-36)

NT-proBNP — N-terminal pro–B-type natriuretic peptide

NYHA — New York Heart Association

PCS — Physical component summary (from SF-36)

PT – preferred term

QoL — Quality of life

RA — Right atrium / Right atrial

RCT — Randomized controlled trial

RVEF — Right ventricular ejection fraction

RR — Relative risk

SAE — Serious adverse event

SAF — Safety analysis set

SGLT2 — Sodium–glucose cotransporter 2

SF-36 — Short Form-36 health survey

SOB — Shortness of breath

SOC — System Organ Class

T1/T2 — Relaxation time parameters in magnetic resonance imaging

1.6 Myoflame-19 protocol Amendment History

Protocol Version 1.1 (Approved 21 Sept 2022)

Initial approved protocol.

Changes in Version 1.2

Substantive methodological updates:

- Added pregnancy testing at week 16 and for missed periods.
- Added Data Safety Monitoring Committee.
- Added atrial fibrillation and clinically significant arrhythmias as exclusion criteria; clarified exclusion of suspected structural heart disease.
- Added restriction on dietary supplements (vitamins B, C, D, CoQ10) during treatment period.
- Added an on-site week-6 visit (ECG, labs) aligned with transition to maintenance dosing.

Clarifications (not affecting study design):

- Minor updates to disease context, outcome descriptions, roles/responsibilities.

Changes in Version 2.0 (Approved 24 July 2023)

Eligibility & recruitment:

- Removal of 6-month inclusion window to improve recruitment.
- Updated exclusion for prior immunosuppression to within 10 weeks (from 6 months).
- Allowed antigen tests (not only PCR) for confirmation of prior COVID-19 infection.

Safety & medication:

- Expanded definition of adverse events of special interest (including misuse, overdose, drug interactions).
- Clarified that vaccination is not permitted during study treatment.
- Pregnancy during treatment classified as an AE.

Procedural updates:

- Deleted stratified randomisation by site.
- Added optional oral administration of ivabradine before cardiac MRI.
- Minor corrections to study medication formulation and laboratory listings.

Administrative corrections (not affecting design, endpoints, or analyses) were made to screening documentation, contraceptive wording, and visit logistics.

Changes in Version 3.0

Study timeline:

- Updated study timeline:
- End of recruitment: Q4 2024
- Primary endpoint: Q2 2025
- End of study: Q2 2026

Procedures:

- Adjusted visit windows (baseline–treatment ≤6 weeks; treatment visits ±10 days; week-16 visit ±14 days).
- Allowed week-16 visit to be shortened to primary endpoint assessment in severe fatigue.
- Removed lymphocyte subset analysis and VLDL from laboratory tests.
- Updated RAND-36 questionnaire terminology (“Brain fog”; addition of excessive tachycardia/POTS).

- Allowed Paxlovid for intercurrent COVID-19 and ivabradine 15 mg orally before MRI.

Operational updates:

- Permitted shipment of replacement study medication to participants when needed.
- Administrative updates to contact details and anonymisation procedures.

Changes in Version 3.1 (Approved 1 July 2024)

- Minor correction to study title (grammatical adjustment only).

Changes in Version 3.1 (Approved 17 Dec 2024)

- Non-substantial amendment extending overall trial duration by <10%; no changes to design, endpoints, eligibility, interventions or analysis.

For primary endpoint and supportive analyses as per SAP:

- last patient in: 30th March 2025;
- last patient out: 12th August 2025;
- hard database lock 07th September 2025.

2 Supplementary Results

2.1 Extended Results and Safety Analyses

There was excellent compliance with the study medication in both groups, defined as documented treatment adherence at weeks 2, 6 and 12 (>75% compliance) with minimal missing doses (average compliance: 0.98 (0.96-0.99) across both groups; 1- taken every day, 0.5 taken every other day). A total of 236 (96%) individuals achieved the target dose of losartan 50 mg, with similar distribution between the groups. Change of losartan treatment due to side-effects was similar between the groups (intervention: n=16 (12.9%), placebo n=17 (13.9%)). The total cumulative dose of prednisolone per individual within the study period was 970 mg (950, 980), averaging about 8 mg a day, similar between the groups. There was a similar rate of off-the-schedule prednisolone reduction within the first 4 weeks (intervention, n=14 (11.3%); placebo: n= 8 (6.6%)). Change of prednisolone treatment due to side effects was similar between the groups (intervention: n=24 (19.4%), placebo n=22 (18.0%)).

The SAF for study medication comprised a total of 270 individuals, receiving at least one dose of medication (**Supplementary tables**). During the study, 185 AEs were reported, of which 9 were classified as SAEs. The incidence of AEs was similar in both groups, with no excess of SAEs in the intervention group. Counted on MedDRA SOC-PT level, common AEs (>10%) included: infections (n=82, 30%), general (n=59, 22%); neurological (n=44, 16%), cardiovascular (n=38, 14%), gastrointestinal system (n=29, 11%). Among 9 SAEs, 4 included hospitalization, and 5 important medical events. Treatment with IMP was temporarily paused in three patients who experienced SAEs. None of the SAEs included prespecified serious complications, including syncope, septic shock, rise in cardiac biomarkers, drop in eGFR, hypotensive or hypertensive crisis. All participants with SAEs recovered without sequelae. There were no between-group differences in worsening of cardiovascular symptoms, defined as an increase in NYHA class, onset of clinical heart failure (intervention: n= 51 (38%), placebo: n=59 (43%)). In total, 7 AEs were reported as special situations for the IMP.

The SAF set for gadobutrol comprised a total of 302 individuals. Participants received on average 7.4 ml and 7.2 ml of gadobutrol at baseline and follow-up CMR assessment. There were 3 mild AEs (nausea after administration) likely causally related to the contrast agent. There was no case of a contrast-agent induced allergic reaction. Fourteen patients received 0.2 mmol/kg instead of 0.1 mmol/kg in error, with no symptoms or sequelae.

3 Supplementary Tables

Table S1. Synopsis. Summary of the trial design, objectives, primary and secondary endpoints, eligibility criteria, sample size and key safety parameters, extracted from the trial protocol.

**Key Inclusion Criteria:**	• Participants ≥ 18 years<br>• Participants with documented recent COVID19 infection (>4 weeks)<br>• PASC Syndrome, defined by persistence or new symptoms, not present prior to the infection.<br>• CMR evidence of inflammatory cardiac involvement at BL by any of the following criteria:<br>• Increased native T1≥ 1130 ms at 3.0 Tesla (or 1030 ms at 1.5 Tesla) and/or;<br>• Increased native T2 ≥39.5 ms at 3.0 Tesla (or 49.5 at 1.5 Tesla) and/or<br>• present non-ischaemic myopericardial LGE and/or;<br>• LVEF ≥45 - ≤50%.<br>• Willingness to comply with the study procedures and study protocol
**Key Exclusion Criteria**	• Severe acute COVID illness requiring hospitalisation<br>• Known allergy to or intolerance of the study medications<br>• Symptomatic hypotension (systolic blood pressure less than 90 mm Hg), not reversible with oral hydration<br>• Any previous or current use of ACE inhibitors, AR Blockers<br>• Any previous oral prednisolone, or any other immunosuppressive or biological treatment (within prior 10 weeks)<br>• History or CMR evidence of pre-existing significant heart disease, including:<br>a. Known cardiac impairment with LVEF ≤44%<br>b. Congestive heart failure (NYHA III-IV)<br>c. Active heart failure treatment<br>d. Established ischaemic heart disease, peripheral arterial disease and/or cerebrovascular disease;<br>e. Persistent or permanent atrial fibrillation or significant heart rhythm abnormalities<br>f. Congenital or clinically relevant valvular heart disease (moderate or severe)<br>g. Specific cardiomyopathy (hypertrophic, hypertensive heart disease, amyloidosis, previous myocarditis, non-ischaemic dilated cardiomyopathy, arrhythmogenic right ventricular cardiomyopathy, non-compaction cardiomyopathy, etc).<br>• Known significant concomitant diseases that are likely to interfere with the evaluation of the participant's safety and of the study outcome (e.g. diabetes, lung or hepatic disease, epilepsy, psychiatric disorders, renal disease with a current estimated GFR <30 mL/min/1.73 m^2 using MDRD formula, chronic systemic infection or immunocompromise)<br>• Exceeding scanner bore and table-holding capacity: Weight >125 kg, BMI > 35 kg/m<br>• Contraindications to contrast-enhanced CMR imaging, e.g.<br>a. MR-unsafe implantable device<br>b. known allergy to gadolinium-based contrast agent (CBGA)<br>• For female participants:<br>a. Pregnant or breastfeeding women<br>b. Women of childbearing potential not willing to use highly effective contraception (as defined in 18.2.13)<br>• Known alcohol, drug or chemical abuse<br>• Participants currently participating in an investigational study or for whom participation is planned.<br>• Unable to provide written informed consent.<br>• Participants with CMR evidence of structural heart disease or incidental heart rhythm abnormalities will be advised to see their own doctor for further investigation.

**Objectives & Endpoints:**	
Primary efficacy objective:	To determine efficacy of a combined immunosuppressive and antiremodelling therapy in COVID-19 related postacute inflammatory cardiovascular involvement determined by CMR to reduce inflammatory myocardial injury compared to placebo
Primary efficacy endpoint:	Absolute LVEF change to baseline at W16, measured by CMR, compared between the verum and placebo group by absolute treatment difference
Secondary efficacy objectives:	To determine the efficacy of a combined immunosuppressive and antiremodelling therapy for 16W in COVID-19 related postacute inflammatory cardiovascular involvement determined by CMR compared to placebo at all available time points compared to BL, by improvement in other clinical parameters.<br>• Scar burden by late gadolinium enhancement (LGE)<br>• Cardiopulmonary exercise testing (CPET)<br>• Myocardial T1 and T2 mapping measures<br>• Cardiac structure (LV volume and mass)<br>• Myocardial deformation/strain<br>• Aortic wall imaging (LGE) and stiffness (PWV)<br>• Symptom Score (Modified Canadian Chest pain scale, NYHA, MRC Dyspnoea scale, Long COVID Questionnaire(2))<br>• QoL (RAND 36-Item Health Survey Version 2.0)<br>• Compliance/Tolerance of therapy<br>• Assessment of treatment response<br>• Progression to HF, MACE and death, compared to placebo after 1- and years' time.
Secondary efficacy endpoints:	Secondary endpoints will be analysed at all available visits for both treatment groups. For all continuous endpoints, "changes" refer to the difference between the visit measurement and baseline (BL, absolute and in %):<br><br>**Reported in present manuscript:**<br>• Mean T1 and T2 values (ms) and change thereof compared to BL<br>• Mean LV volume (ml/m^2) and mass (g/m^2) and change thereof compared to BL<br>• Mean Myocardial strain (%) and change thereof compared to BL<br>• Average Symptom Score and change thereof compared to BL;<br>• Compliance: Frequency of prescribed medication consumed, participant diary and drug adherence, total cumulative steroid dose;<br>• Tolerance: number of participants who required dose reduction or treatment cessation due to side effects, especially due to<br>o Hypotension<br>o Unblinding due to safety issues<br>• Number of Responders by achieving:<br>o partial response: a normal CMR result is defined as normal T1 and T2, normal gender-age predicted LVEF, non-dilated LV<br>o total response: in addition to the above absence of LGE<br>**To be reported in future manuscripts following dedicated analyses.**<br>• Mean LGE extent (%) and change thereof compared to BL<br>• CPET (achieved Work Rate, VO_2max, VCO_2 max, RER, AT and slope) and change thereof compared to BL<br>• Aortic wall thickness (LGE, mm) and change thereof compared to BL;<br>• Mean Pulse wave velocity (m/s) and change thereof compared to BL;<br>• Proportion of participant with HF or MACE after 1 years<br>• 1- and year Event-free survival

**Key safety parameters:**	Frequency, severity and number of adverse events (AE):<br>• Proportion of participants with infectious complications (a combination of at least two of the following:<br>o fever ≥38.5°C,<br>o rise on hsCRP,<br>o neutrophilia,<br>o lymphocytosis,<br>o need for antiviral or antibiotic treatment)<br>• Proportion of participants with symptomatic hypotension (blackouts and systolic BP<90 mmHg) accompanied by a syncope<br>• Proportion of participants with symptomatic tachycardia with heartrate >110/min accompanied by a syncope;<br>• Proportion of participants with a significant rise in cardiac biomarkers (hsTNT, NTproBNP, >3-times the BL)<br>• Proportion of participants with onset of clinical hear failure.<br>• Absolute changes in lipid profile, HbA1c, thyroid function tests compared to BL<br>• Proportion of participants with a significant drop in eGFR compared to BL (>25%)<br>• Proportion of participants with worsening of cardiovascular symptoms (increase in CCS, NYHA class, clinical heart failure)<br>• Proportion of participants with acute psychotic episode<br>• Proportion of participants with hypertensive crisis (with systolic BP>180mmHg and diastolic >120 mmHg)
**Additional Assessments:**	Blood samples (whole blood, serum and plasma) will be retained for measurement for future measurements, as indicated in the section 9.2.6.
**Study design:**	**Multicentre, randomised double-blind, placebo controlled clinical trial 1:1 randomisation**
**Planned sample size:**	n=280 (including 8% drop-out), 140 in each the combined verum and combined placebo arm
**Total number of centres:**	4 centres (in Germany and Austria)
**Study medication:**	Prednisolone and Losartan
**Study groups:**	1. **Verum arm:** Prednisolone + Losartan<br>2. **Placebo arm:** Placebo 1+ 2 (corresponds to standard-of-care)
**Randomisation**	Central stratified randomisation with 1:1 of subgroups in the verum and corresponding placebo arms
**Study design and methodology:**	Two-hundred and eighty consecutive participants with PASC-CVS syndrome and evidence of inflammatory cardiac involvement on baseline CMR, and no previous history of cardiac conditions or hospitalisation during the acute illness, will be randomized 1:1 into the verum or the placebo arm.<br>The resulting treatment groups are<br>In the **<u>Verum</u>** arm (n=140 participants),<br>**(A)** Anti-remodelling therapy with <u>Losartan</u> and Immunosuppressive therapy with <u>Prednisolone</u><br>In the **<u>Placebo</u>**-arm (n=140 participants)<br>**(B)** Placebo 1 of antiremodelling therapy and Placebo 2 of immunosuppressive therapy<br>Assessments will include clinical assessment with symptoms questionnaires, blood testing, CMR and CPET will be performed at BL and W16; pregnancy test will be performed in all women at baseline and W16. In addition, women of childbearing potential (WOCBP) will be instructed to contact their study physician immediately in the absence of menstruation or in case of other clinical evidence of pregnancy for further<br>clarification.

	Participants will receive study treatment, as appropriate for the randomised arm. Participants will undertake titration of Losartan (starting dose 12.5 mg orally at night,

	with increase in dose as tolerated every 1-2 weeks, to maximally tolerated dose: maximal daily dose 50 mg). Uptitration will be dependent participants' tolerance of Losartan. Prednisolone will be commenced at 20 mg and tapered over 6 weeks to maintenance dose of 5 mg to be taken 6-16 weeks.<br>Additional visits will be performed at W2, W6 (onsite or remotely with test conducted at the general physician) and W12 for<br>• Medication review<br>• Optimisation of the therapy<br>• Safety visit (video-call).<br>Primary efficacy endpoint assessment will be conducted at W16 after commencement of treatment.<br>Participants will be followed up over a 1- year period for outcome endpoints (efficacy endpoint).
**Concomitant Treatments**	The following concomitant medication will be permitted:<br>• Paracetamol, ibuprofen on per needed basis<br>• Rate-control: betablockers or ivabradine<br>• Contraception<br>• Prior COVID vaccination<br>• Paxlovid in case of acute COVID infection<br>Colchicine, immunosuppressive therapies or interventions, and any vaccinations will not be permitted for the duration of the treatment period. Multivitamins and other supplements will be discouraged for the duration of treatment period (16 weeks) of this study.

3.1 Table S2. Baseline Demographic, Clinical, and CMR Characteristics by Treatment Group (Randomized Population).

Parameter	Intervention, N=139,<br>Median [Q1, Q3], n, %	Placebo, N=140,<br>Median [Q1, Q3], n, %
**Demographics**		
Age (years)	38.0 (32.0, 47.0)	39.0 (29.5, 46.0)
Females, n,%	107 (77.0)	97 (69.3)
Ethnic origin (Caucasian), n,%	129 (92.8)	132 (94.3)
BMI (kg/m2)	23.8 (21.1, 26.7)	23.5 (21.1, 27.9)
BP systolic (mmHg)	123.0 (112.0, 132.0)	124.0 (115.0, 134.0)
BP diastolic (mmHg)	81.0 (74.0, 88.0)	82.5 (75.0, 90.0)
Heart rate (bpm)	75.5 (68.0, 84.0)	78.0 (69.0, 85.0)
More than 1 COVID infection, n,%	76 (54.7)	90 (64.3)
COVID Reinfection during the study, n,%	11 (7.9)	11 (7.9)

Parameter	Intervention, N=139, Median [Q1, Q3], n, %	Placebo, N=140, Median [Q1, Q3], n, %
Number of COVID vaccinations taken	3.0 (3.0, 3.0)	3.0 (3.0, 3.0)
More than 2 COVID vaccinations, n,%	105 (75.5)	116 (82.9)
Duration of PostCOVID illness (days)	444.0 (172.0, 702.0)	399.5 (165.5, 623.0)
**Symptoms**		
>5 symptoms, n,%	118 (84.9)	120 (85.7)
Fatigue, n,%	135 (97.1)	133 (95.0)
Headache, n,%	91 (65.5)	96 (68.6)
Shortness of breath, n,%	131 (94.2)	135 (96.4)
Loss of smell, n,%	24 (17.3)	10 (7.1)
Persistent cough, n,%	51 (36.7)	43 (30.7)
Sore throat, n,%	61 (43.9)	63 (45.0)
Fever, n,%	28 (20.1)	33 (23.6)
Muscle pains, n,%	107 (77.0)	117 (83.6)
Skipped meals, n,%	35 (25.2)	41 (29.3)
Chest Pain, n,%	117 (84.2)	121 (86.4)
Diarrhea, n,%	32 (23.0)	37 (26.4)
Hoarse Voice, n,%	37 (26.6)	37 (26.4)
Abdominal Pain, n,%	48 (34.5)	53 (37.9)
Delirium, n,%	95 (68.3)	99 (70.7)
Loss of Consciousness, n,%	37 (26.6)	33 (23.6)
Palpitations, n,%	87 (62.6)	86 (61.4)
Chest pain Scale ≥II, n,%	104 (74.8)	107 (76.4)

Parameter	Intervention, N=139, Median [Q1, Q3], n, %	Placebo, N=140, Median [Q1, Q3], n, %
NYHA ≥III, n,%	100 (71.9)	102 (72.9)
QoL- SF-36 - PCS	28.3 (23.9, 34.8)	30.1 (24.8, 35.2)
QoL- SF-36 - MCS	39.7 (28.8, 45.8)	38.1 (31.4, 45.3)
**Blood values**		
Hb (mg/ml)	136.5 (130.0, 145.0)	137.0 (129.5, 148.1)
Hematocrit (%)	39.7 (37.7, 42.2)	40.2 (38.0, 42.8)
MCV (fL)	87.2 (84.8, 89.9)	87.6 (85.4, 90.4)
Leukocytes (abs) (pt/nl)	6.4 (5.5, 7.7)	6.4 (5.3, 7.6)
Neutrophils (abs.) (pt/nl)	4.0 (3.2, 5.2)	4.0 (3.0, 5.0)
Lymphocytes (total) (pt/nl)	1.8 (1.5, 2.1)	1.8 (1.4, 2.2)
Eosinophils (abs.) (pt/nl)	0.1 (0.1, 0.2)	0.1 (0.1, 0.2)
eGFR (ml/min/1.73m^2)	105.0 (95.4, 116.1)	105.9 (97.0, 116.0)
Sodium (mmol/l)	139.0 (138.0, 140.0)	139.0 (138.0, 140.0)
Potassium (mmol/l)	4.2 (4.0, 4.4)	4.2 (4.0, 4.5)
Total cholesterol (mg/dl)	190.0 (169.0, 219.0)	186.5 (163.1, 208.5)
LDL (mg/dl)	116.2 (92.0, 138.3)	110.4 (89.9, 133.6)
HDL (mg/dl)	56.2 (47.5, 68.5)	57.9 (48.9, 72.0)
HbA1c (%)	5.1 (4.9, 5.4)	5.2 (5.0, 5.4)
D-dimer (ng/ml) (log transformed)	5.3 (5.3, 5.6)	5.3 (5.3, 5.6)
Fibrinogen (g/l) (log transformed)	1.1 (1.0, 1.2)	1.0 (0.9, 1.2)
Troponin T (ng/ml) (log transformed)	1.1 (-5.7, 1.3)	-1.5 (-5.5, 1.4)
NTproBNP (pg/ml) (log transformed)	3.8 (3.5, 4.2)	3.8 (3.2, 4.3)

Parameter	Intervention, N=139, Median [Q1, Q3], n, %	Placebo, N=140, Median [Q1, Q3], n, %
CRP (mg/dl) (log transformed)	-2.5 (-3.2, -1.7)	-2.5 (-3.2, -1.9)
Thyroid stimulating hormone (mU/l)	1.5 (1.1, 2.0)	1.6 (1.2, 2.1)
**CMR Imaging**		
LVEF (%)	56.0 (54.0, 59.0)	55.0 (53.0, 57.0)
LVEDVI (ml/m²)	80.7 (73.6, 86.6)	81.7 (73.8, 88.7)
LVESVI (ml/m²)	35.8 (30.5, 39.1)	35.9 (32.0, 41.5)
LV mass index (g/m²)	40.2 (37.2, 45.0)	41.6 (38.0, 48.8)
GLS (%)	20.0 (18.9, 21.0)	19.6 (18.3, 20.9)
RVEF (%)	56.0 (53.0, 60.0)	56.0 (52.0, 59.0)
RVEDVI (ml/m²)	81.9 (73.9, 90.4)	82.5 (74.6, 93.3)
RVESVI (ml/m²)	36.7 (30.6, 42.3)	35.7 (30.9, 43.5)
LA area (cm²)	23.0 (21.0, 25.0)	23.0 (21.0, 25.0)
RA area (cm²)	20.0 (17.0, 22.0)	20.0 (18.0, 22.0)
Native T1 (ms)	1138.0 (1131.0, 1152.0)	1140.5 (1131.0, 1153.0)
Native T2 (ms)	40.1 (38.9, 41.7)	39.8 (39.0, 41.3)
Perimyocardial enhancement, n,%	94 (67.6)	83 (59.3)
Pericardial effusion, n,%	93 (66.9)	86 (61.4)
Pericardial effusion >1cm, n,%	3 (2.2)	3 (2.1)

Abbreviations: body mass index - BMI, blood pressure - BP, coronavirus disease 2019 - COVID-19; estimated glomerular filtration rate – eGFR, calculated using the Modification of Diet in Renal Disease equation), N-terminal pro–B-type natriuretic peptide - NT-proBNP, New York Health Association -NYHA; Quality of life -QoL physical component summary (PCS) and mental component summary (MCS) scores; low-density lipoprotein cholesterol – LDL; high-density lipoprotein cholesterol – HDL; glycated hemoglobin A – HbA1c. C-reactive protein – CRP; left ventricular – LV; right ventricular – RV; LV ejection fraction – LVEF; LV enddiastolic volume index - LV-EDVI; LV endsystolic volume index – LV-ESVI; global longitudinal strain – GLS, left atrium - LA; right atrium – RA; Pericardial effusion >1cm defines the anatomical measurement threshold of pathophysiological relevance

3.2 **Table S3. Baseline Demographic, Clinical, and CMR Characteristics by Treatment Group (Per-Protocol Population).**

	Baseline	
Parameter	*Intervention N=123 Median [Q1, Q3] , n, %*	*Placebo N=121 Median [Q1, Q3] , n, %*
Demographics		
Age (years)	39.0 (32.0, 46.0)	39.0 (30.0, 46.0)
Females, n,%	96 (78.0)	82 (67.8)
Ethnic origin (Caucasian), n,%	115 (93.5)	114 (94.2)
BMI (kg/m^2)	24.1 (21.3, 27.0)	23.7 (21.1, 28.1)
BP systolic (mmHg)	124.0 (112.0, 133.0)	124.0 (115.0, 134.0)
BP diastolic (mmHg)	81.0 (74.0, 88.0)	83.0 (74.0, 91.0)
Heart rate (bpm)	75.0 (67.0, 83.0)	77.0 (69.0, 85.0)
More than 1 COVID-19 infection, n,%	67 (54.5)	77 (63.6)
COVID-19 Reinfection during the study, n,%	8 (6.5)	10 (8.3)
Number of COVID-19 vaccinations taken	470.0 (173.0, 702.0)	399.0 (169.0, 625.0)
More than 2 COVID-19 vaccinations, n,%	3.0 (3.0, 3.0)	3.0 (3.0, 3.0)
Duration of post-COVID condition (days)	95 (77.2)	103 (85.1)
**Symptoms**		
>5 symptoms, n,%	104 (84.6)	103 (85.1)
Fatigue, n,%	119 (96.7)	115 (95.0)
Headache, n,%	80 (65.0)	83 (68.6)
Shortness of breath, n,%	117 (95.1)	116 (95.9)

Parameter	*Baseline* Intervention N=123 Median [Q1, Q3], n, %	Placebo N=121 Median [Q1, Q3], n, %
Loss of smell, n,%	19 (15.4)	8 (6.6)
Persistent cough, n,%	49 (39.8)	38 (31.4)
Sore throat, n,%	56 (45.5)	52 (43.0)
Fever, n,%	25 (20.3)	31 (25.6)
Unusual muscle pains, n,%	95 (77.2)	101 (83.5)
Skipped meals, n,%	30 (24.4)	36 (29.8)
Chest tightness, n,%	103 (83.7)	104 (86.0)
Diarrhea, n,%	30 (24.4)	31 (25.6)
Hoarse Voice, n,%	34 (27.6)	31 (25.6)
Abdominal Pain, n,%	42 (34.1)	45 (37.2)
Delirium, n,%	88 (71.5)	85 (70.2)
Loss of Consciousness, n,%	30 (24.4)	26 (21.5)
Palpitations (excessive tachycardia), n,%	81 (65.9)	76 (62.8)
Canadian Chest pain Scale ≥II, n,%	93 (75.6)	92 (76.0)
NYHA ≥III, n,%	88 (71.5)	87 (71.9)
QoL- SF-36 - PCS	28.5 (23.6, 34.9)	30.1 (25.0, 35.3)
QoL- SF-36 - MCS	39.7 (28.7, 45.9)	38.4 (31.9, 45.6)
**Blood values**		
Hemoglobin (g/L)	136.0 (130.0, 145.0)	138.0 (130.0, 148.2)
Hematocrit (%)	39.8 (37.8, 42.2)	40.2 (38.1, 42.8)
Mean corpuscular volume (fL)	86.9 (84.8, 89.9)	87.9 (85.7, 90.0)
Leukocytes (abs) (pt/nl)	6.4 (5.6, 7.9)	6.5 (5.3, 7.5)

Parameter	*Baseline* *Intervention* *N=123* *Median [Q1, Q3] , n, %*	*Placebo* *N=121* *Median [Q1, Q3] , n, %*
Neutrophils (abs.) (pt/nl)	4.0 (3.3, 5.2)	4.0 (3.0, 4.9)
Lymphocytes (total) (pt/nl)	1.8 (1.5, 2.2)	1.8 (1.4, 2.1)
Eosinophils (abs.) (pt/nl)	0.1 (0.1, 0.2)	0.1 (0.1, 0.2)
eGFR (MDRD; ml/min/1.73m^2	102.6 (94.0, 115.6)	105.5 (97.6, 115.6)
Sodium (mmol/l)	139.0 (138.0, 140.0)	139.0 (138.0, 140.0)
Potassium (mmol/l)	4.2 (4.0, 4.4)	4.2 (4.0, 4.5)
Total cholesterol (mg/dl)	191.0 (169.0, 218.0)	186.0 (162.2, 207.0)
LDL (mg/dl)	116.9 (91.8, 138.1)	111.2 (90.0, 132.3)
HDL (mg/dl)	56.1 (46.9, 68.2)	58.2 (49.2, 72.0)
HbA1c (%)	5.1 (4.9, 5.4)	5.2 (5.0, 5.4)
D-dimer (ng/ml) (log transformed)	5.3 (5.3, 5.6)	5.3 (5.3, 5.6)
Fibrinogen (g/l) (log transformed)	1.1 (1.0, 1.2)	1.0 (0.9, 1.2)
Troponin T (ng/ml) (log transformed)	-4.6 (-5.7, 1.2)	-5.0 (-5.7, 1.4)
NT-proBNP (pg/ml) (log transformed)	3.8 (3.5, 4.2)	3.7 (3.2, 4.3)
CRP (mg/dl) (log transformed)	-2.4 (-3.2, -1.6)	-2.5 (-3.2, -1.9)

Parameter	*Baseline*<br>*Intervention*<br>*N=123*<br>*Median [Q1, Q3], n, %*	*Placebo*<br>*N=121*<br>*Median [Q1, Q3], n, %*
TSH (mU/l)	1.5 (1.1, 2.0)	1.6 (1.2, 2.1)
**CMR Imaging**		
LVEF (%)	56.0 (54.0, 59.0)	56.0 (53.0, 57.0)
LV-EDVI (ml/m²)	80.6 (73.3, 86.6)	82.3 (74.7, 91.1)
LV-ESVI (ml/m²)	35.4 (30.5, 39.1)	36.4 (31.9, 41.9)
LVMI (g/m²)	40.3 (37.2, 44.9)	42.1 (38.2, 49.6)
GLS (%)	20.0 (18.9, 21.0)	19.7 (18.3, 20.9)
RVEF (%)	56.0 (53.0, 60.0)	55.0 (52.0, 59.0)
RV-EDVI (ml/m²)	81.7 (73.6, 90.3)	84.5 (75.4, 94.8)
RV-ESVI (ml/m²)	36.5 (29.8, 42.2)	36.7 (31.1, 44.3)
LA area (cm²)	23.0 (21.0, 25.7)	23.7 (21.0, 25.5)
RA area (cm²)	20.0 (17.0, 22.0)	21.0 (18.0, 23.0)
Native T1 (ms)	1140.0 (1131.0, 1153.0)	1141.0 (1131.0, 1153.0)
Native T2 (ms)	40.1 (39.1, 41.6)	39.8 (39.0, 41.3)
Perimyocardial enhancement (non-ischemic), n,%	81 (65.9)	71 (58.7)
Pericardial effusion, n,%	82 (66.7)	71 (58.7)
>1cm, n,%	3 (2.4)	2 (1.7)

Abbreviations: quartile -Q; body mass index - BMI, blood pressure - BP, corona-virus disease 2019 - COVID-19; estimated glomerular filtration rate – eGFR, calculated using the Modification of Diet in Renal Disease – MDRD, equation), N-terminal pro–B-type natriuretic peptide - NT-proBNP, New York Health Association -NYHA; Quality of life -QoL, was assessed using the 36-Item Short Form Health Survey (SF-36) physical component summary (PCS) and mental component summary (MCS) scores; low-density lipoprotein cholesterol – LDL; high-density lipoprotein cholesterol – HDL; glycated hemoglobin A – HbA1c. C-reactive protein – CRP; thyroid stimulating hormone - TSH; left ventricular – LV; right ventricular – RV; LV ejection fraction – LVEF; LV enddiastolic volume index - LV-EDVI; LV endsystolic volume index – LV-ESVI; LV mass index – LVMI; global longitudinal strain - GLS left atrium - LA; right atrium – RA;

3.3 **Table S4. Between-Group Differences and Least-Squares Mean Changes from Baseline to Week 16 in Demographic, Clinical, and CMR Characteristics (Per-Protocol Population).**

	W16 absolute change			*W16 percentage change*		
Parameter	*Intervention LS means (95% CI), n, %*	*Placebo LS means (95% CI), n, %*	*Difference LS means (95% CI)*	*Intervention LS means (95% CI)*	*Placebo LS means (95% CI)*	*Difference LS means (95% CI)*
**Vital signs**						
BMI (kg/m^2)	0.49 (0.31-0.66)	0.17 (-0.01-0.35)	0.32 (0.07-0.57)	1.87 (1.17-2.58)	0.73 (0.03-1.44)	1.14 (0.14-2.14)
BP systolic (mmHg)	-6.93 (-8.71--5.14)	-1.86 (-3.65--0.07)	-5.07 (-7.60--2.54)	-5.10 (-6.54--3.66)	-1.09 (-2.54-0.35)	-4.01 (-6.05--1.97)
BP diastolic (mmHg)	-5.35 (-6.68--4.02)	-2.63 (-3.97--1.29)	-2.72 (-4.61--0.82)	-5.94 (-7.63--4.25)	-2.67 (-4.37--0.97)	-3.27 (-5.67--0.87)
Heart rate (bpm)	-3.64 (-5.27--2.01)	-5.70 (-7.34--4.06)	2.06 (-0.25-4.38)	-3.15 (-5.38--0.93)	-6.30 (-8.53--4.07)	3.15 (-0.01-6.30)
**Symptoms**						
>5 symptoms, n,%	83 (67.5)	91 (75.2)				
Fatigue, n,%	106 (86.2)	112 (92.6)				
Headache, n,%	73 (59.3)	73 (60.3)				
Shortness of breath, n, %	103 (83.7)	101 (83.5)				
Loss of smell, n,%	15 (12.2)	9 (7.4)				
Persistent cough, n,%	37 (30.1)	29 (24.0)				
Sore throat, n,%	47 (38.2)	59 (48.8)				
Fever (FV), n,%	18 (14.6)	21 (17.4)				
Unusual muscle pains, n,%	79 (64.2)	89 (73.6)				
Skipped meals, n,%	24 (19.5)	30 (24.8)				
Chest tightness, n,%	78 (63.4)	77 (63.6)				
Diarrhea, n,%	27 (22.0)	27 (22.3)				

Parameter	W16 absolute change<br>Intervention LS means (95% CI), n, %	Placebo LS means (95% CI), n, %	Difference LS means (95% CI)	W16 percentage change<br>Intervention LS means (95% CI)	Placebo LS means (95% CI)	Difference LS means (95% CI)
Hoarse Voice, n,%	35 (28.5)	30 (24.8)				
Abdominal Pain, n,%	33 (26.8)	47 (38.8)				
Delirium, n,%	90 (73.2)	86 (71.1)				
Loss of Consciousness, n,%	7 (5.7)	8 (6.6)				
Palpitations (excessive tachycardia), n,%	60 (48.8)	54 (44.6)				
Canadian Chest pain Scale ≥II, n,%	53 (43.1)	59 (48.8)				
NYHA ≥III, n,%	56 (45.5)	65 (53.7)				
QoL- SF-36 - PCS	0.84 (-0.29-1.98)	0.62 (-0.55-1.79)	0.22 (-1.41-1.86)	3.84 (-0.02-7.70)	3.99 (0.03-7.95)	-0.15 (-5.69-5.38)
QoL- SF-36 - MCS	1.61 (-0.07-3.29)	0.61 (-1.12-2.33)	1.00 (-1.41-3.41)	10.19 (3.56-16.82)	10.39 (3.58-17.20)	-0.20 (-9.71-9.30)
Blood values						
Hemoglobin (g/L)	-1.96 (-3.25--0.67)	-0.70 (-1.99-0.60)	-1.26 (-3.09-0.57)	-1.27 (-2.19--0.34)	-0.38 (-1.30-0.54)	-0.89 (-2.19-0.42)
Hematocrit (%)	-0.27 (-0.76-0.23)	0.33 (-0.16-0.83)	-0.60 (-1.30-0.10)	-10.18 (-102.71-82.34)	94.68 (2.15-187.20)	-104.86 (-235.73-26.01)
Mean corpuscular volume (fL)	1.60 (1.25-1.94)	0.17 (-0.17-0.52)	1.42 (0.93-1.91)	1.87 (1.47-2.27)	0.22 (-0.18-0.62)	1.65 (1.09-2.22)
Leukocytes (abs) (pt/nl)	0.78 (0.50-1.06)	-0.16 (-0.44-0.12)	0.94 (0.54-1.34)	13.44 (9.26-17.63)	0.06 (-4.13-4.24)	13.39 (7.46-19.31)
Neutrophils (abs.) (pt/nl)	1.14 (0.08-2.20)	-0.34 (-1.39-0.72)	1.48 (-0.02-2.97)	42.32 (14.37-70.27)	12.19 (-15.64-40.02)	30.13 (-9.38-69.63)
Lymphocytes (total) (pt/nl)	-0.45 (-0.55--0.34)	-0.28 (-0.39--0.18)	-0.16 (-0.31--0.02)	-9.56 (-14.08--5.04)	2.05 (-2.47-6.56)	-11.61 (-18.01--5.21)

Parameter	W16 absolute change<br>Intervention LS means (95% CI), n, %	Placebo LS means (95% CI), n, %	Difference LS means (95% CI)	W16 percentage change<br>Intervention LS means (95% CI)	Placebo LS means (95% CI)	Difference LS means (95% CI)
Eosinophils (abs.) (pt/nl)	-0.05 (-0.06--0.03)	0.01 (-0.01-0.02)	-0.05 (-0.08--0.03)	-11.38 (-37.23-14.48)	46.47 (20.50-72.43)	-57.84 (-94.48- -21.20)
eGFR (MDRD; ml/min/1.73)	-0.77 (-2.42-0.88)	-0.34 (-1.99-1.32)	-0.43 (-2.77-1.91)	-0.24 (-1.88-1.41)	-0.28 (-1.93-1.37)	0.04 (-2.29-2.37)
Sodium (mmol/l)	-0.17 (-0.51-0.18)	0.01 (-0.34-0.36)	-0.18 (-0.67-0.31)	-0.11 (-0.36-0.14)	0.02 (-0.23-0.28)	-0.13 (-0.48-0.23)
Potassium (mmol/l)	0.11 (0.04-0.17)	0.10 (0.03-0.16)	0.01 (-0.08-0.10)	2.83 (1.31-4.36)	2.56 (1.01-4.10)	0.28 (-1.90-2.45)
Cholesterol (mg/dl)	2.20 (-1.62-6.03)	-5.33 (-9.17- -1.49)	7.53 (2.10-12.96)	1.78 (-0.23-3.79)	-2.01 (-4.03-0.02)	3.79 (0.93-6.65)
LDL (mg/dl)	-0.74 (-5.23-3.75)	-4.18 (-8.68-0.31)	3.44 (-2.92-9.80)	67.60 (-66.73-201.92)	102.62 (-31.71-236.95)	-35.02 (-225.24-155.20)
HDL (mg/dl)	4.21 (2.42-6.01)	-1.52 (-3.31-0.28)	5.73 (3.19-8.27)	8.29 (5.73-10.85)	-0.92 (-3.48-1.64)	9.21 (5.58-12.84)
HbA1c (%)	0.11 (0.07-0.14)	0.05 (0.01-0.08)	0.06 (0.01-0.11)	2.11 (1.46-2.75)	0.98 (0.32-1.64)	1.13 (0.20-2.05)
D-dimer (ng/ml) (log transformed)	-0.02 (-0.07-0.03)	-0.04 (-0.09-0.02)	0.02 (-0.06-0.09)	-0.97 (-5.20-3.26)	-4.48 (-8.76--0.19)	3.51 (-2.51-9.53)
Fibrinogen (g/l) (log transformed)	-0.02 (-0.05-0.00)	-0.00 (-0.03-0.03)	-0.02 (-0.06-0.02)	-1.39 (-4.25-1.47)	1.37 (-1.53-4.26)	-2.76 (-6.84-1.32)
Troponin T (ng/ml) (log transformed)	-0.87 (-1.26--0.48)	-0.81 (-1.20- -0.42)	-0.07 (-0.62-0.49)	-71.08 (-101.50- -40.67)	-62.97 (-93.38- -32.55)	-8.12 (-51.13-34.90)
NT-proBNP (pg/ml) (log transformed)	-0.13 (-0.25--0.01)	0.01 (-0.11-0.13)	-0.15 (-0.32-0.02)	-2.38 (-6.37-1.62)	2.87 (-1.14-6.88)	-5.25 (-10.90-0.41)
CRP (mg/dl) (log transformed)	-0.14 (-0.27--0.00)	0.07 (-0.07-0.21)	-0.20 (-0.40--0.01)	27.89 (-10.86-66.65)	-2.94 (-42.02-36.14)	30.83 (-24.23-85.90)

Parameter	*W16 absolute change* Intervention LS means (95% CI), n, %	*W16 absolute change* Placebo LS means (95% CI), n, %	*W16 absolute change* Difference LS means (95% CI)	*W16 percentage change* Intervention LS means (95% CI)	*W16 percentage change* Placebo LS means (95% CI)	*W16 percentage change* Difference LS means (95% CI)
TSH (mU/l)	-0.28 (-0.40--0.16)	-0.05 (-0.17-0.07)	-0.23 (-0.40--0.06)	-17.89 (-132.29-96.52)	92.67 (-22.22-207.55)	-110.55 (-272.95-51.85)
**CMR Imaging**						
LVEF (%)	2.30 (1.70-2.89)	1.33 (0.73-1.92)	0.97 (0.13-1.82)	4.22 (3.15-5.29)	2.57 (1.48-3.65)	1.66 (0.13-3.18)
LVEDVI (ml/m²)	2.11 (1.01-3.20)	0.68 (-0.42-1.78)	1.43 (-0.13-2.99)	2.81 (1.37-4.25)	1.38 (-0.06-2.81)	1.44 (-0.61-3.48)
LVESVI (ml/m²)	-0.73 (-1.50-0.03)	-0.72 (-1.48-0.05)	-0.02 (-1.11-1.07)	-1.72 (-3.91-0.47)	-0.96 (-3.15-1.23)	-0.76 (-3.86-2.34)
LVMI (g/m²)	0.38 (-0.41-1.17)	0.39 (-0.40-1.18)	-0.01 (-1.13-1.11)	4.25 (-3.82-12.31)	3.45 (-4.59-11.48)	0.80 (-10.67-12.27)
GLS (%)	0.48 (0.17-0.79)	0.22 (-0.09-0.54)	0.25 (-0.19-0.70)	2.72 (1.10-4.35)	1.81 (0.17-3.45)	0.92 (-1.40-3.23)
RVEF (%)	1.00 (0.22-1.79)	0.55 (-0.24-1.34)	0.45 (-0.67-1.57)	2.15 (0.73-3.57)	1.43 (-0.00-2.86)	0.73 (-1.30-2.75)
RVEDVI (ml/m²)	1.98 (0.79-3.17)	0.59 (-0.60-1.78)	1.38 (-0.30-3.07)	2.81 (1.35-4.27)	1.37 (-0.09-2.83)	1.45 (-0.63-3.52)
RVESVI (ml/m²)	0.29 (-0.71-1.29)	-0.52 (-1.52-0.48)	0.81 (-0.60-2.23)	7.27 (-3.63-18.16)	1.67 (-9.23-12.56)	5.60 (-9.88-21.08)
LA area (cm²)	-1.20 (-1.75--0.65)	-0.69 (-1.24--0.13)	-0.51 (-1.29-0.27)	-3.12 (-5.43--0.81)	-1.59 (-3.94-0.76)	-1.53 (-4.83-1.77)
RA area (cm²)	-0.54 (-1.12-0.05)	-0.22 (-0.82-0.37)	-0.31 (-1.15-0.52)	0.86 (-1.48-3.20)	-0.27 (-2.67-2.12)	1.13 (-2.22-4.48)
Native T1 (ms)	-13.40 (-17.57--9.23)	-11.07 (-15.28--6.86)	-2.33 (-8.26-3.60)	-1.15 (-1.52--0.79)	-0.94 (-1.31--0.58)	-0.21 (-0.73-0.31)
Native T2 (ms)	-0.66 (-1.26--0.06)	-0.33 (-0.94-0.27)	-0.33 (-1.18-0.53)	-4.17 (-14.72-6.38)	-12.27 (-22.91--1.64)	8.10 (-6.89-23.10)

Parameter	*W16 absolute change* *Intervention LS means (95% CI), n, %*	*Placebo LS means (95% CI), n, %*	*Difference LS means (95% CI)*	*W16 percentage change* *Intervention LS means (95% CI)*	*Placebo LS means (95% CI)*	*Difference LS means (95% CI)*
Perimyocardial enhancement (non-ischemic), n,%	61 (49.6)	65 (53.7)				
Pericardial effusion, n, %	85 (69.1)	80 (66.1)				
>1cm, n,%	4 (3.3)	1 (0.8)				

Abbreviations: least squares -LS; confidence interval – CI; body mass index - BMI, blood pressure - BP, corona-virus disease 2019 - COVID-19; estimated glomerular filtration rate – eGFR, calculated using the Modification of Diet in Renal Disease – MDRD, equation), N-terminal pro–B-type natriuretic peptide - NT-proBNP, New York Health Association -NYHA; Quality of life -QoL, was assessed using the 36-Item Short Form Health Survey (SF-36) physical component summary (PCS) and mental component summary (MCS) scores; low-density lipoprotein cholesterol – LDL; high-density lipoprotein cholesterol – HDL; glycated hemoglobin A – HbA1c. C-reactive protein – CRP; thyroid stimulating hormone - TSH; left ventricular – LV; right ventricular – RV; LV ejection fraction – LVEF; LV enddiastolic volume index - LV-EDVI; LV endsystolic volume index – LV ESVI; LV mass index – LVMI; global longitudinal strain - GLS left atrium - LA; right atrium – RA;

3.4 Table S5. Proportional Differences between Treatment Groups (Intention-to-Treat Population)

Parameter	BL		Week 16	
	Proportional differences	95% CI	Proportional differences	95% CI
>5 symptoms, n,%	-0.6	-9.5-8.4	-7.7	-18.9-3.6
Fatigue, n,%	1.7	-3.2-6.6	-6.3	-14.0-1.3
Headache, n,%	-2.7	-14.5-9.1	-0.2	-12.4-12.1
Shortness of breath, n,%	-1.5	-6.9-3.8	0.3	-9.1-9.7
Loss of smell, n,%	8.8	1.1-16.5	4.7	-2.7-12.1
Persistent cough, n,%	8.4	-3.5-20.3	6.1	-5.1-17.2
Sore throat, n,%	2.5	-9.9-14.9	-10.5	-22.8-1.9
Fever, n,%	-5.2	-15.7-5.2	-2.7	-11.8-6.4
Unusual muscle pains, n,%	-6.2	-16.1-3.7	-8.4	-20.0-3.1
Skipped meals, n,%	-4.5	-15.6-6.6	-4.4	-14.8-6.0
Chest tightness, n,%	-2.2	-11.1-6.7	-0.2	-12.2-11.8
Diarrhea, n,%	-1.2	-12.0-9.6	-0.4	-10.7-10.0
Hoarse Voice, n,%	1.2	-9.9-12.3	2.8	-8.2-13.9
Abdominal Pain, n,%	-2.2	-14.2-9.8	-11.9	-23.6--0.2
Delirium, n,%	2.1	-9.3-13.5	2.9	-8.3-14.1
Loss of Consciousness, n,%	3.7	-6.8-14.2	-0.9	-6.9-5.1
Palpitations (excessive tachycardia), n,%	1.3	-10.6-13.1	3.0	-10.0-16.1
Canadian Chest pain Scale ≥II, n,%	-0.4	-11.1-10.2	-4.8	-17.3-7.6
NYHA ≥III, n,%	-0.4	-11.6-10.9	-8.1	-20.6-4.3
Myocardial LGE (non-ischemic)	7.1	-5.0-19.2	-3.3	-15.8-9.2

Abbreviations: Confidence intervals – CI; late gadolinium enhancement – LGE, New York Heart Association – NYHA,

3.5 Table S6. Proportional Differences between Treatment Groups (Per-Protocol Population)

Parameter	BL Proportional differences	BL 95% CI	Week 16 Proportional differences	Week 16 95% CI
>5 symptoms, n,%	-0.6	-9.6-8.4	-7.7	-19.0-3.6
Fatigue, n,%	1.7	-3.3-6.7	-6.4	-14.1-1.3
Headache, n,%	-3.6	-15.4-8.3	-1.0	-13.3-11.3
Shortness of breath, n,%	-0.7	-5.9-4.5	0.3	-9.0-9.6
Loss of smell, n,%	8.8	1.1-16.6	4.8	-2.7-12.2
Persistent cough, n,%	8.4	-3.5-20.4	6.1	-5.0-17.2
Sore throat, n,%	2.6	-9.9-15.0	-10.5	-22.9-1.8
Fever, n,%	-5.3	-15.8-5.2	-2.7	-11.9-6.5
Unusual muscle pains, n,%	-6.2	-16.2-3.7	-9.3	-20.9-2.2
Skipped meals, n,%	-5.4	-16.5-5.8	-5.3	-15.7-5.1
Chest tightness, n,%	-2.2	-11.2-6.8	-0.2	-12.3-11.9
Diarrhea, n,%	-1.2	-12.1-9.6	-0.4	-10.8-10.1
Hoarse Voice, n,%	2.0	-9.1-13.1	3.7	-7.4-14.7
Abdominal Pain, n,%	-3.0	-15.1-9.0	-12.0	-23.7--0.3
Delirium, n,%	1.3	-10.1-12.7	2.1	-9.2-13.3
Loss of Consciousness, n,%	2.9	-7.6-13.4	-0.9	-7.0-5.1
Palpitations (excessive tachycardia), n,%	1.9	-9.9-13.8	2.6	-10.5-15.7
Canadian Chest pain Scale ≥II, n,%	-0.4	-11.2-10.3	-5.7	-18.2-6.8
NYHA ≥III, n,%	-0.4	-11.7-10.9	-8.2	-20.7-4.3
Myocardial LGE (non-ischemic)	7.2	-5.0-19.3	-4.1	-16.7-8.4

Abbreviations: Confidence intervals – CI; late gadolinium enhancement – LGE, New York Heart Association – NYHA,

3.6 Table S7. Compliance and Tolerability in the Intention-to-Treat Population (ITT).

Parameter	Intervention Median [Q1, Q3], n, %	Placebo Median [Q1, Q3], n, %
Regular end of treatment, n,%	124 (100.0)	122 (100.0)
Compliance	100.0 (100.0, 100.0)	100.0 (100.0, 100.0)
Frequency of prescribed medication consumed	1.0 (1.0, 1.0)	1.0 (1.0, 1.0)
Total cumulative steroid dose [mg] (Prednisolone) based on drug accountability	980.0 (965.0, 1000.0)	975.0 (955.0, 990.0)
Total cumulative steroid dose [mg] (Prednisolone) based on patient diary	972.5 (952.5, 982.5)	970.0 (945.0, 980.0)
Total cumulative steroid dose [mg] (Losartan) based on drug accountability	4500.0 (4325.0, 4700.0)	4487.5 (4337.5, 4725.0)
Total cumulative steroid dose [mg (Losartan) based on patient diary	4436.0 (4329.5, 4548.5)	4436.0 (4249.0, 4486.0)
12.5 mg as maximum Losartan dose based on patient diary, n,%	1 (0.8)	0 (0.0)
25 mg as maximum Losartan dose based on patient diary, n,%	0 (0.0)	1 (0.8)
37.5 mg as maximum Losartan dose based on patient diary, n,%	4 (3.2)	2 (1.6)
50 mg as maximum Losartan dose based on patient diary, n,%	117 (94.4)	119 (97.5)
Reduction of Prednisolone within 4 weeks, n,%	14 (11.3)	8 (6.6)
Change of Losartan treatment due to side effects, n, %	12 (9.7)	13 (10.7)
Losartan dose reduction due to side effects, n,%	2 (1.6)	4 (3.3)
Losartan temporarily treatment discontinuation due to side effects, n,%	8 (6.5)	9 (7.4)
Losartan permanently treatment discontinuation due to side effects, n,%	2 (1.6)	0 (0.0)
Change of Prednisolone treatment due to side effects, n,%	17 (13.7)	17 (13.9)
Prednisolone dose reduction due to side effects, n,%	13 (10.5)	13 (10.7)
Prednisolone temporarily treatment discontinuation due to side effects, n,%	3 (2.4)	6 (4.9)
Prednisolone permanently treatment discontinuation due to side effects, n,%	2 (1.6)	0 (0.0)

3.7 Table S8. Compliance and Tolerability in the Per-Protocol Population (PP).

Parameter	Intervention Median [Q1, Q3], n, %	Placebo Median [Q1, Q3], n, %
Regular end of treatment, n,%	123 (100.0)	121 (100.0)
Compliance	100.0 (100.0, 100.0)	100.0 (100.0, 100.0)
Frequency of prescribed medication consumed	1.0 (1.0, 1.0)	1.0 (1.0, 1.0)
Total cumulative steroid dose [mg] (Prednisolone) based on drug accountability	980.0 (965.0, 1000.0)	977.5 (955.0, 990.0)
Total cumulative steroid dose [mg] (Prednisolone) based on patient diary	975.0 (955.0, 985.0)	970.0 (945.0, 980.0)
Total cumulative steroid dose [mg] (Losartan) based on drug accountability	4500.0 (4325.0, 4700.0)	4487.5 (4337.5, 4725.0)
Total cumulative steroid dose [mg (Losartan) based on patient diary	4436.0 (4335.0, 4561.0)	4436.0 (4249.0, 4486.0)
12.5 mg as maximum Losartan dose based on patient diary, n,%	1 (0.8)	0 (0.0)
25 mg as maximum Losartan dose based on patient diary, n,%	0 (0.0)	1 (0.8)
37.5 mg as maximum Losartan dose based on patient diary, n,%	4 (3.3)	2 (1.7)
50 mg as maximum Losartan dose based on patient diary, n,%	116 (94.3)	118 (97.5)
Reduction of Prednisolone within 4 weeks, n,%	13 (10.6)	7 (5.8)
Change of Losartan treatment due to side effects, n,%	11 (8.9)	13 (10.7)
Losartan dose reduction due to side effects, n,%	2 (1.6)	4 (3.3)
Losartan temporarily treatment discontinuation due to side effects, n,%	7 (5.7)	9 (7.4)
Losartan permanently treatment discontinuation due to side effects, n,%	2 (1.6)	0 (0.0)
Change of Prednisolone treatment due to side effects, n,%	16 (13.0)	17 (14.0)
Prednisolone dose reduction due to side effects, n,%	13 (10.6)	13 (10.7)
Prednisolone temporarily treatment discontinuation due to side effects, n, %	2 (1.6)	6 (5.0)
Prednisolone permanently treatment discontinuation due to side effects, n, %	2 (1.6)	0 (0.0)

3.8 Table S9. Safety outcomes stratified by treatment group in the safety population (SAF).

Parameter	Intervention n, %	Placebo n, %
AE, n,%	97 (72.4)	88 (64.7)
AE related to Prednisolone, n,%	44 (32.8)	35 (25.7)
AE related to Losartan, n,%	29 (21.6)	20 (14.7)
AE related to Gadovist, n,%	4 (3.0)	5 (3.7)
SAE, n,%	3 (2.2)	6 (4.4)
Hospitalization, n,%	2 (1.5)	2 (1.5)
Important medical event, n,%	1 (0.7)	4 (2.9)
Worsening of cardiovascular symptoms (increase in NYHA class at any follow up visit), n,%	51 (38.1)	59 (43.4)

Safety analyses were performed in the safety population (SAF; all participants who received at least one dose of study medication, n = 270). Adverse events were summarized by MedDRA System Organ Class (SOC) and Preferred Term (PT). Percentages are based on the number of participants in each treatment-group SAF; participants could report more than one event.

Abbreviations: AE – adverse event; SAE – serious adverse event; NYHA denotes the New York Heart Association functional classification of heart failure.

3.9 **Table S10. Adverse Events by Treatment Group in the Safety Population, Summarized by MedDRA System Organ Class.**

	Intervention		Placebo		Total	
SOC	N	%	N	%	N	%
Infections and infestations	44	32.84	38	27.94	82	30.37
General disorders and administration site conditions	33	24.63	26	19.12	59	21.85
Nervous system disorders	21	15.67	23	16.91	44	16.30
Cardiac disorders	20	14.93	18	13.24	38	14.07
Gastrointestinal disorders	11	8.21	18	13.24	29	10.74
Psychiatric disorders	10	7.46	11	8.09	21	7.78
Injury, poisoning and procedural complications	5	3.73	10	7.35	15	5.56
Investigations	9	6.72	4	2.94	13	4.81
Skin and subcutaneous tissue disorders	10	7.46	3	2.21	13	4.81
Musculoskeletal and connective tissue disorders	7	5.22	5	3.68	12	4.44
Vascular disorders	4	2.99	4	2.94	8	2.96
Eye disorders	4	2.99	3	2.21	7	2.59
Respiratory, thoracic and mediastinal disorders	2	1.49	5	3.68	7	2.59
Reproductive system and breast disorders	3	2.24	2	1.47	5	1.85
Ear and labyrinth disorders	1	0.75	2	1.47	3	1.11
Metabolism and nutrition disorders	2	1.49	1	0.74	3	1.11
Immune system disorders	2	1.49	0	0.00	2	0.74
Blood and lymphatic system disorders	1	0.75	0	0.00	1	0.37
Renal and urinary disorders	1	0.75	0	0.00	1	0.37

Safety analyses were performed in the safety population (SAF; all participants who received at least one dose of study medication, n = 270). Adverse events were summarized by MedDRA System Organ Class (SOC) and Preferred Term (PT). Percentages are based on the number of participants in each treatment-group SAF; participants could report more than one event.

Supplementary Note 1. Trial Protocol

The full MYOFLAME-19 trial protocol (Version 3.1, dated 29 May 2024) is reproduced on the following pages.

DocuSign Envelope ID: 4F4388D5-A568-4FAC-82D1-39CB3AB82D1B

Clinical Study Protocol

Full Study Title: Randomised placebo controlled clinical trial of efficacy of MYOcardial protection in postacute inFLAMmatory cardiac involvEment due to COVID-19 (MYOFLAME-19)

Titel der Studie: Randomisierte, placebo-kontrollierte klinische Prüfung der Wirksamkeit des myokardialen Schutzes bei postakuter entzündlicher Herzbeteiligung infolge COVID-19 (MYOFLAME-19)

Sponsor: Goethe University Frankfurt, represented by the President; Theodor-Adorno-Platz 6; D-60323 Frankfurt/Main, Germany

Document Type:	Clinical Study Protocol
Protocol Version, Date:	Version 3.1; 29th May 2024
EudraCT number:	2022-001682-12
Study Type;	Phase III
Investigational Medicinal Product:	Prednisolone oral<br>Losartan oral<br>Placebo for Prednisolone<br>Placebo for Losartan

STATEMENT OF CONFIDENTIALITY:
The information in this document contains scientific and commercial information that is privileged or confidential and may not be disclosed unless such disclosure is required by federal law or regulations. In any event, persons to whom the information is disclosed must be informed that the information is privileged or confidential and may not be disclosed by them. These restrictions on disclosure will apply equally to all future information supplied to you, which is indicated as privileged or confidential.

DocuSign Envelope ID: 4F4388D5-A568-4FAC-82D1-39CB3AB82D1B

Roles and Responsibilities

**Sponsor**	
Name:	**Goethe University Frankfurt**, represented by the President
Address:	Theodor-Adorno-Platz 6; D-60323 Frankfurt/Main
**Representative of the Sponsor**	
Name:	[redacted]
Address:	[redacted] University Hospital Frankfurt; Theodor-Stern-Kai 7, D-60590 Frankfurt/Main
Phone:	[redacted]
E-Mail:	[redacted]
**Principal Investigator**	
Name:	[redacted]
Address:	[redacted] Goethe University Frankfurt Theodor-Stern-Kai 7, D-60590 Frankfurt/Main
Phone:	[redacted]
E-Mail:	[redacted]
**Deputy Principal Investigator**	
Name:	[redacted]
Address:	[redacted] Goethe University Frankfurt Goethe University, University Hospital Frankfurt Theodor-Stern-Kai 7, D-60590 Frankfurt/Main
Phone:	[redacted]
E-Mail:	[redacted]

**Sponsor's Project Medical Officer (PMO)**	
Name:	[redacted]
Address:	[redacted] Goethe University Frankfurt Goethe University, University Hospital Frankfurt Theodor-Stern-Kai 7, D-60590 Frankfurt/Main
Phone:	[redacted]
E-Mail:	[redacted]
**Contract Research Organisation (delegated for project management, monitoring, data managent, pharmacovivigilance, and biostatistics)**	
Name:	Alcedis GmbH
Address:	Winchesterstraße 3, 335394 Gießen
Phone:	[redacted]
E-Mail:	[redacted]
**Responsible Statistician**	
Name:	[redacted]
Address:	Alcedis GmbH Winchesterstraße 3, 35394 Gießen
Phone	[redacted]
E-Mail:	[redacted]

DocuSign Envelope ID: 4F4388D5-A568-4FAC-82D1-39CB3AB82D1B

Sponsor Signature Page

Protocol title: **Randomised placebo controlled clinical trial of efficacy of MYOcardial protection with postacute inFLAMmatory cardiac involvEment due to COVID-19**

Study Code: **MYOFLAME-19**

Phase: **III**

Sponsor: **University Frankfurt of Johann Wolfgang Goethe University**

We, the undersigned, have read this protocol and agree that it contains all necessary information required to conduct the trial and that the protocol is in compliance with International Conference on Harmonization (ICH) and Good Clinical Practice (GCP) guidelines and applicable local regulations.

Sponsor Representative:

DocuSigned by:

25.06.2024 | 16:22 [redacted]

Date [redacted] Goethe University Frankfurt)

Principal Investigators:

DocuSigned by:

25.06.2024 | 16:22 [redacted]

Date [redacted] Goethe University Frankfurt)

DocuSigned by:

25.06.2024 | 19:1[redacted]

Date [redacted]
Goethe University Frankfurt)

Project Medical Officer (PMO):

DocuSigned by:

25.06.2024 | 16:22 [redacted]

Date [redacted] Goethe University Frankfurt)

Statistician:

DocuSigned by:

25.06.2024 | 16:36 [redacted]

Date [redacted] (Alcedis GmbH, Gießen)

Drug Safety Manager:

DocuSigned by:

25.06.2024 | 20:1[redacted]

Date [redacted] (Alcedis GmbH, Gießen)

DocuSign Envelope ID: 4F4388D5-A568-4FAC-82D1-39CB3AB82D1B

Investigator Signature Page

Protocol title: Randomised placebo controlled clinical trial of efficacy of MYOcardial protection with postacute inFLAMmatory cardiac involvEment due to COVID-19

Study Code: MYOFLAME-19

EudraCT-No: 2022-001682-12

Protocol Version: V3.1

Date of Protocol: 29th May 2024

I confirm that I have read this study protocol, I understand it, and I will work according to this protocol and to the ethical principles stated in the latest version of the Declaration of Helsinki, the applicable guidelines for good clinical practices, or the applicable laws and regulations of the country of the study site for which I am responsible, whichever provides the greater protection of the individual. I am aware of my responsibilities as an Investigator / Representative under the GCP national regulations and trial protocol. I agree to appropriately direct and assist the staff under my control, who will be involved in this clinical trial. This is documented in a training log.

Site Number: ______________________

Site Name: ______________________

Site Address: ______________________

____

____

Date Investigator, Print Name Signature

Date Representative, Print Name Signature

Version History

Version	Date	Comments	Changes in PIS
1.1	06/09/2022		Yes
2.0	27.02.2023		Yes
3.0	28.11.2023		Yes
3.1	29.05.2024		Yes

1 STUDY SYNOPSIS 8
2 LIST OF ABBREVIATIONS 13
3 BACKGROUND AND RATIONALE 15
3.1 BACKGROUND 15
3.1.1 Context of disease and participant population 15
3.1.2 Pathophysiology of COVID19-related cardiovascular involvement 15
3.1.3 Current treatment options 17
3.2 RATIONALE 17
3.2.1 Rationale for the choice of investigational medicinal products 17
3.2.2 Rationale for diagnostic approach 19
3.2.3 Rationale for Dosage Selection 20
3.3 BENEFIT/RISK ASSESSMENT 20
3.3.1 Potential Benefits 20
3.3.2 Important known and potential risks 20
3.3.3 Assessment of Benefit and Risk 21
4 STUDY OBJECTIVES 22
4.1 EFFICACY 22
4.1.1 Primary efficacy objective 22
4.1.2 Primary efficacy endpoint 22
4.1.3 Secondary efficacy objectives 22
4.1.4 Secondary efficacy endpoints 22
4.2 SAFETY ENDPOINTS 22
4.2.1 Safety objective 22
4.2.2 Safety Parameters 22
4.2.3 Study Drug Levels 23
5 STUDY POPULATION 23
5.1 ENROLMENT AND STUDY CENTRES 23
5.2 TARGET POPULATION 23
5.3 INCLUSION CRITERIA 23
5.4 EXCLUSION CRITERIA 23
5.5 JUSTIFICATION OF INCLUSION CRITERIA 24
6 STUDY DESIGN 25
6.1 SUMMARY OF STUDY DESIGN 25
6.2 FOLLOW-UP 25
6.3 FLOWCHART – STUDY DESIGN 26
6.4 RANDOMIZATION AND BLINDING 26
6.4.1 Randomization 26
6.4.2 Blinding 26
6.4.3 Unblinding for Suspected Unexpected Serious Adverse Reactions (SUSAR) 26
6.4.4 Emergency Procedures for Unblinding 27
6.5 STUDY MEDICATION 27
6.5.1 Active Study Medication 27
6.5.2 Placebo 27
6.6 CONCOMITANT MEDICATION AND TREATMENT 28
7 STUDY PLAN 29
8 INVESTIGATIONAL MEDICINAL PRODUCTS 30
8.1 INVESTIGATIONAL MEDICINAL PRODUCT (IMP) 30
8.2 ADMINISTRATION, DOSAGE AND DURATION OF TREATMENT 30
8.3 STUDY TREATMENT RETURN AND RECONCILIATION 30
8.3.1 Drug Accountability 30
8.3.2 Drug destruction 31
8.4 SUPPLY, PACKAGING, LABELLING AND STORAGE 31
8.4.1 Supply, Packaging 31
8.4.2 Labelling and Storage 31
9 CONDUCT OF THE STUDY 32

DocuSign Envelope ID: 4F4388D5-A568-4FAC-82D1-39CB3AB82D1B

9.1 SCHEDULING OF STUDY PROCEDURES 32
9.2 CLINICAL PROCEDURES AND EVALUATIONS 37
9.2.1 Informed Consent 37
9.2.2 Demographics 37
9.2.3 Medical history: 37
9.2.4 Vital signs and BP Diary 37
9.2.5 Symptoms scores and Quality of Life 37
9.2.6 Laboratory tests: 37
9.2.7 ECG: 38
9.2.8 CMR: 38
9.2.9 CPET: 38
9.2.10 Participant Treatment Diary: 38
9.2.11 Outcome Endpoints: 38
9.3 MANAGEMENT OF ADVERSE EVENTS 39
9.3.1 Adverse Events Reporting 39
9.3.2 Other adverse events 40
9.4 HANDLING OF SAFETY PARAMETERS 40
9.4.1 Serious Adverse Events (Immediately Reportable to the Sponsor or Project Management) 40
9.4.2 Treatment and Follow-up of Adverse Events 40
9.4.3 Follow-up of Abnormal Laboratory Test Values 41
9.4.4 Pregnancy 41
9.5 DISCONTINUATION OF THE TREATMENT 41
9.6 PREMATURE WITHDRAWAL OR TERMINATION OF THE STUDY 41
9.6.1 Premature Withdrawal of the Participant 41
9.6.2 Criteria for Termination of the Study 41
9.6.3 Plan for Treatment after the End of Study Treatment 42

10 CRITERIA FOR EVALUATION OF STUDY RESULTS 43

10.1 CRITERIA FOR EVALUATION OF EFFICACY 43
10.1.1 Primary Efficacy Endpoint 43
10.1.2 Secondary efficacy endpoints 43
10.2 CRITERIA FOR EVALUATION OF SAFETY 43
10.3 DESCRIPTION OF POPULATION AND PARTICIPANT GROUPS FOR ANALYSES 43
10.3.1 Full Analysis Set (FAS) 43
10.3.2 Intention to treat set (mITT) 43
10.3.3 Per Protocol Population 43

11 STATISTICAL METHODS 44

11.1 SAMPLE SIZE ESTIMATION 44
11.2 BIAS AND PREVENTION METHODS 45
11.3 SUMMARY STATISTICS AND GRAPHICAL PRESENTATION 45
11.4 OUTCOME ANALYSES 46
11.4.1 Primary Efficacy Analysis 46
11.4.2 Secondary Efficacy Analysis: 46
11.4.3 Safety Analyses 46
11.4.4 Interim analysis 48
11.4.5 Other types of analyses 48
11.4.6 Handling of missing data and values above/below the LOQ 48
11.4.7 Handling of therapy changes and unscheduled visits 48
11.5 REPLACEMENT POLICY (ENSURING ADEQUATE NUMBERS OF EVALUABLE SUBJECTS) 48
11.5.1 For Centres 48
11.6 LIMITATIONS 48

12 ORGANISATIONAL STRUCTURE 49

12.1 SPONSOR 49
12.2 CONTRACT RESEARCH ORGANISATION (CRO) 49
12.3 STEERING COMMITTEE 49
12.4 DATA SAFETY AND MONITORING COMMITTEE (DSMC) 49
12.5 CORE LABORATORY FOR CMR 49
12.6 FUNDING 49
12.7 INSURANCE 49

DocuSign Envelope ID: 4F4388D5-A568-4FAC-82D1-39CB3AB82D1B

13 DATA COLLECTION AND MONITORING 50
13.1 CASE REPORT FORMS (CRF) 50
13.2 DATA MANAGEMENT AND CLEANING 50
13.3 MONITORING 50
13.3.1 Virtual and/or Onsite Monitoring 50
13.3.2 In-house Monitoring 50
13.4 AUDIT/INSPECTION 50

14 INVESTIGATOR RESPONSIBILITIES AND OBLIGATIONS 51
14.1 DECLARATION OF HELSINKI 51
14.2 ETHICS COMMITTEE AND REGULATORY AUTHORITY REVIEW 51
14.3 INFORMED CONSENT AND PARTICIPANT PROTECTION 51
14.3.1 Participant Informed Consent 51
14.3.2 Participant Data Protection 51
14.4 STUDY PROTOCOL ADHERENCE AND MODIFICATIONS 51
14.4.1 Protocol Adherence 51
14.4.2 Changes to Protocol and Related Procedures 52
14.5 INVESTIGATIONAL PRODUCT CONTROL 52
14.6 DATA COLLECTION AND DOCUMENTATION 52
14.7 REPORTING OF AEs AND SAEs 52
14.8 RECORDS RETENTION 52
14.9 CONFIDENTIALITY OF TRIAL DOCUMENTS AND PARTICIPANT RECORDS 52
14.10 INTELLECTUAL PROPERTY AND COPYRIGHT PROTECTION OF CLINICAL TRIAL DATA 53
14.11 PUBLICATION OF DATA 53
14.12 DIRECT ACCESS TO SOURCE DATA/DOCUMENTS 53
14.13 TRIAL NETWORK REGISTRATION 53

15 STUDY DURATION AND GLOBAL END OF STUDY DEFINITION 54
16 REFERENCES 55
17 NORMATIVE REFERENCES 61
18 APPENDICES 62
18.1 DEFINITIONS ACCORDING ICH GUIDELINES FOR CLINICAL SAFETY DATA MANAGEMENT, DEFINITIONS AND STANDARDS FOR EXPEDITED REPORTING, TOPIC E2 62
18.2 ATTACHMENTS 63
18.2.1 Myoflame-19 Study Information (Webpage, Flyer) 63
18.2.2 Myoflame-19 Study Onlineregistration 64
18.2.3 Myoflame-19 Study Information Screening Questionnaire (per Phone or via verschluesselte Email) 65
18.2.4 Long COVID Questionnaire (Sudre et al), in German. 66
18.2.5 Modified Chest Discomfort Severity Score 68
18.2.6 MRC Dyspnea Severity Score 69
18.2.7 RAND 36-item Health Survey, Version 2. 70
18.2.8 Participant Blood Pressure and Heart Rate Diary and Participant Medication Diary . 76
18.2.9 IET-CVI CMR Protocol 77
18.2.10 CE Certificate SuiteHeart Neosoft 81
18.2.11 CE Certificate Omron 82
18.2.12 Members of Steering and Data Safety Monitoring Committees 83
18.2.13 Highly effective methods for contraception 85

1 STUDY SYNOPSIS

**Protocol title :**	**Randomised placebo controlled clinical trial of efficacy of MYOcardial protection with postacute inFLAMmatory cardiac involvEment due to COVID-19 (MYOFLAME-19)**
**Sponsor :**	Johann Wolfgang Goethe University Frankfurt
**Project phase :**	III
**Indication/Diagnosis:**	Inflammatory cardiovascular involvement due to COVID-19, defined by CMR
**Rationale**	Postacute sequelae of COVID-19 infection (PASC) are increasingly recognised complications and are defined by lingering symptoms, not present prior to the infection, typically persisting for more than 4 weeks(1). Cardiac symptoms due to postacute inflammatory cardiac involvement affect a broad segment of people, who were previously well and may have had only mild acute illness (PASC-cardiovascular syndrome, PASC-CVS). Symptoms may be contiguous with the acute illness, however, more commonly they occur after a delay. Symptoms related to the cardiovascular system include exertional dyspnoea, exercise intolerance chest tightness, pulling or burning chest pain, and palpitations. Phenotypically, it is characterised by chronic perivascular and myopericardial inflammation. Cardiac symptoms may be accompanied by manifestations of other organ systems, including fatigue, brain fog, myalgias, skin and joint manifestations, etc, now commonly referred to as the Long COVID or PASC syndrome(2).<br>Evidence suggests inflammatory autoimmune mechanisms, which develop de novo in response to the infection[3]. In PASC-CVS, early subtle inflammatory heart changes with mild functional impairment are often undetectable by routine diagnostic tests, nor accompanied by significant rise in troponin(1). Studies using CMR imaging have identified changes consistent with non-ischaemic cardiovascular inflammatory involvement[22–27], including increased myocardial mapping values and perimyocardial late gadolinium enhancement. Participants may also have subnormal LVEF, however, structural heart disease by profoundly reduced LVEF or dilated heart cavities, or necrotic or thromboembolic complications are not typical findings(3). Likewise, areas of substantial necrosis typically found in classical viral myocarditis(4,5), are rare. These abnormalities can be detected as early as 2 weeks after the infection(6,7) and can be observed several months after the infection(8).<br>Early intervention with immunosuppression and antiremodelling therapy may reduce symptoms and myocardial impairment, by minimising the disease activity and inducing disease remission. Low dose maintenance therapy may help to maintain the disease activity at the lowest possible level. Clinical trials of immunosuppression in participants with viral myocarditis in advanced stages of heart failure have not shown an improved outcome, however there was an improvement of LVEF with antiremodelling therapy in participants with reduced function (9–12). The benefits of early initiations of antiremodelling therapy to reduce symptoms of exercise intolerance are well recognised(13–15), but not commonly employed outside the contexts of heart failure(16) or hypertension. As most participants with inflammatory heart disease only have mild and nonspecific symptoms and few or no structural abnormalities, they are left untreated (standard of care). The aim of this study is to examine the efficacy of a combined immunosuppressive/antiremodelling therapy in participants with PASC symptoms and inflammatory cardiac involvement determined by CMR, to reduce the symptoms and inflammatory myocardial injury and thereby stop the progression to reduced LVEF, HF and death.

**Key Inclusion Criteria:**	• Participants ≥ 18 years<br>• Participants with documented recent COVID19 infection (>4 weeks)<br>• PASC Syndrome, defined by persistence or new symptoms, not present prior to the infection.<br>• CMR evidence of inflammatory cardiac involvement at BL by any of the following criteria:<br>• Increased native T1≥ 1130 ms at 3.0 Tesla (or 1030 ms at 1.5 Tesla) and/or;<br>• Increased native T2 ≥39.5 ms at 3.0 Tesla (or 49.5 at 1.5 Tesla) and/or<br>• present non-ischaemic myopericardial LGE and/or;<br>• LVEF ≥45 - ≤50%.<br>• Willingness to comply with the study procedures and study protocol
**Key Exclusion Criteria**	• Severe acute COVID illness requiring hospitalisation<br>• Known allergy to or intolerance of the study medications<br>• Symptomatic hypotension (systolic blood pressure less than 90 mm Hg), not reversible with oral hydration<br>• Any previous or current use of ACE inhibitors, AR Blockers<br>• Any previous oral prednisolone, or any other immunosuppressive or biological treatment (within prior 10 weeks)<br>• History or CMR evidence of pre-existing significant heart disease, including:<br>a. Known cardiac impairment with LVEF ≤44%<br>b. Congestive heart failure (NYHA III-IV)<br>c. Active heart failure treatment<br>d. Established ischaemic heart disease, peripheral arterial disease and/or cerebrovascular disease;<br>e. Persistent or permanent atrial fibrillation or significant heart rhythm abnormalities<br>f. Congenital or clinically relevant valvular heart disease (moderate or severe)<br>g. Specific cardiomyopathy (hypertrophic, hypertensive heart disease, amyloidosis, previous myocarditis, non-ischaemic dilated cardiomyopathy, arrhythmogenic right ventricular cardiomyopathy, non-compaction cardiomyopathy, etc).<br>• Known significant concomitant diseases that are likely to interfere with the evaluation of the participant's safety and of the study outcome (e.g. diabetes, lung or hepatic disease, epilepsy, psychiatric disorders, renal disease with a current estimated GFR <30 mL/min/1.73 m² using MDRD formula, chronic systemic infection or immunocompromise)<br>• Exceeding scanner bore and table-holding capacity: Weight >125 kg, BMI > 35 kg/m^2<br>• Contraindications to contrast-enhanced CMR imaging, e.g.<br>a. MR-unsafe implantable device<br>b. known allergy to gadolinium-based contrast agent (CBGA)<br>• For female participants:<br>a. Pregnant or breastfeeding women<br>b. Women of childbearing potential not willing to use highly effective contraception (as defined in 18.2.13)<br>• Known alcohol, drug or chemical abuse<br>• Participants currently participating in an investigational study or for whom participation is planned.<br>• Unable to provide written informed consent.<br>• Participants with CMR evidence of structural heart disease or incidental heart rhythm abnormalities will be advised to see their own doctor for further investigation.

DocuSign Envelope ID: 4F4388D5-A568-4FAC-82D1-39CB3AB82D1B

**Objectives & Endpoints:**	
Primary efficacy objective:	To determine efficacy of a combined immunosuppressive and antiremodelling therapy in COVID-19 related postacute inflammatory cardiovascular involvement determined by CMR to reduce inflammatory myocardial injury compared to placebo
Primary efficacy endpoint:	Absolute LVEF change to baseline at W16, measured by CMR, compared between the verum and placebo group by absolute treatment difference
Secondary efficacy objectives:	To determine the efficacy of a combined immunosuppressive and antiremodelling therapy for 16W in COVID-19 related postacute inflammatory cardiovascular involvement determined by CMR compared to placebo at all available time points compared to BL, by improvement in other clinical parameters.<br>1. Scar burden by late gadolinium enhancement (LGE)<br>2. Cardiopulmonary exercise testing (CPET)<br>3. Myocardial T1 and T2 mapping measures<br>4. Cardiac structure (LV volume and mass)<br>5. Myocardial deformation/strain<br>6. Aortic wall imaging (LGE) and stiffness (PWV)<br>7. Symptom Score (Modified Canadian Chest pain scale, NYHA, MRC Dyspnoea scale, Long COVID Questionnaire(2))<br>8. QoL (RAND 36-Item Health Survey Version 2.0)<br>9. Compliance/Tolerance of therapy<br>10. Assessment of treatment response<br>11. Progression to HF, MACE and death, compared to placebo after 1- and years' time.
Secondary efficacy endpoints:	Secondary endpoints will be analysed at all available visits for both treatment groups. For all continuous endpoints, "changes" refer to the difference between the visit measurement and baseline (BL, absolute and in %):<br>• Mean LGE extent (%) and change thereof compared to BL<br>• CPET (achieved Work Rate, VO_2max, VCO_2 max, RER, AT and slope) and change thereof compared to BL<br>• Mean T1 and T2 values (ms) and change thereof compared to BL<br>• Mean LV volume (ml/m^2) and mass (g/m^2) and change thereof compared to BL<br>• Mean Myocardial strain (%) and change thereof compared to BL<br>• Aortic wall thickness (LGE, mm) and change thereof compared to BL;<br>• Mean Pulse wave velocity (m/s) and change thereof compared to BL;<br>• Average Symptom Score and change thereof compared to BL;<br>• Compliance: Frequency of prescribed medication consumed, participant diary and drug adherence, total cumulative steroid dose;<br>• Tolerance: number of participants who required dose reduction or treatment cessation due to side effects, especially due to<br>o Hypotension<br>o Unblinding due to safety issues<br>• Number of Responders by achieving:<br>o partial response: a normal CMR result is defined as normal T1 and T2, normal gender-age predicted LVEF, non-dilated LV<br>o total response: in addition to the above absence of LGE<br>• Proportion of participant with HF or MACE after 1 years<br>• 1- and year Event-free survival

DocuSign Envelope ID: 4F4388D5-A568-4FAC-82D1-39CB3AB82D1B

**Key safety parameters:**	Frequency, severity and number of adverse events (AE):<br>• Proportion of participants with infectious complications (a combination of at least two of the following:<br>o fever ≥38.5°C,<br>o rise on hsCRP,<br>o neutrophilia,<br>o lymphocytosis,<br>o need for antiviral or antibiotic treatment)<br>• Proportion of participants with symptomatic hypotension (blackouts and systolic BP<90 mmHg) accompanied by a syncope<br>• Proportion of participants with symptomatic tachycardia with heartrate >110/min accompanied by a syncope;<br>• Proportion of participants with a significant rise in cardiac biomarkers (hsTNT, NTproBNP, >3-times the BL)<br>• Proportion of participants with onset of clinical hear failure.<br>• Absolute changes in lipid profile, HbA1c, thyroid function tests compared to BL<br>• Proportion of participants with a significant drop in eGFR compared to BL (>25%)<br>• Proportion of participants with worsening of cardiovascular symptoms (increase in CCS, NYHA class, clinical heart failure)<br>• Proportion of participants with acute psychotic episode<br>• Proportion of participants with hypertensive crisis (with systolic BP>180mmHg and diastolic >120 mmHg)
**Additional Assessments:**	Blood samples (whole blood, serum and plasma) will be retained for measurement for future measurements, as indicated in the section 9.2.6.
**Study design:**	**Multicentre, randomised double-blind, placebo controlled clinical trial 1:1 randomisation**
**Planned sample size:**	n=280 (including 8% drop-out), 140 in each the combined verum and combined placebo arm
**Total number of centres:**	4 centres (in Germany and Austria)
**Study medication:**	Prednisolone and Losartan
**Study groups:**	1. **Verum arm:** Prednisolone + Losartan<br>2. **Placebo arm:** Placebo 1+ 2 (corresponds to standard-of-care)
**Randomisation**	Central stratified randomisation with 1:1 of subgroups in the verum and corresponding placebo arms
**Study design and methodology:**	Two-hundred and eighty consecutive participants with PASC-CVS syndrome and evidence of inflammatory cardiac involvement on baseline CMR, and no previous history of cardiac conditions or hospitalisation during the acute illness, will be randomized 1:1 into the verum or the placebo arm.<br>The resulting treatment groups are<br>In the **Verum** arm (n=140 participants),<br>**(A)** Anti-remodelling therapy with Losartan and Immunosuppressive therapy with Prednisolone<br>In the **Placebo**-arm (n=140 participants)<br>**(B)** Placebo 1 of antiremodelling therapy and Placebo 2 of immunosuppressive therapy<br>Assessments will include clinical assessment with symptoms questionnaires, blood testing, CMR and CPET will be performed at BL and W16; pregnancy test will be performed in all women at baseline and W16. In addition, women of childbearing potential (WOCBP) will be instructed to contact their study physician immediately in the absence of menstruation or in case of other clinical evidence of pregnancy for further clarification.

	Participants will receive study treatment, as appropriate for the randomised arm. Participants will undertake titration of Losartan (starting dose 12.5 mg orally at night, with increase in dose as tolerated every 1-2 weeks, to maximally tolerated dose: maximal daily dose 50 mg). Uptitration will be dependent participants' tolerance of Losartan. Prednisolone will be commenced at 20 mg and tapered over 6 weeks to maintenance dose of 5 mg to be taken 6-16 weeks.<br>Additional visits will be performed at W2, W6 (onsite or remotely with test conducted at the general physician) and W12 for<br>• Medication review<br>• Optimisation of the therapy<br>• Safety visit (video-call).<br>Primary efficacy endpoint assessment will be conducted at W16 after commencement of treatment.<br>Participants will be followed up over a 1- year period for outcome endpoints (efficacy endpoint).
**Concomitant Treatments**	The following concomitant medication will be permitted:<br>• Paracetamol, ibuprofen on per needed basis<br>• Rate-control: betablockers or ivabradine<br>• Contraception<br>• Prior COVID vaccination<br>• Paxlovid in case of acute COVID infection<br>Colchicine, immunosuppressive therapies or interventions, and any vaccinations will not be permitted for the duration of the treatment period. Multivitamins and other supplements will be discouraged for the duration of treatment period (16 weeks) of this study.
**Duration of the study :**	Recruitment phase: Q3/2022 – Q4/2024<br>Till primary endpoint: Q2/2025<br>End of study: Q2/2026
**Data Safety and Monitoring**	A Data Safety and Monitoring Committee (DSMC) will review all safety events on an ongoing ad hoc basis by treatment code and will make recommendations to the Steering Committee on further conduct of the study.
**Statistical Analysis Approach**	All primary and secondary efficacy analysis will be performed for the mITT and for the PP population to account for protocol violations unless stated otherwise. All safety analysis will be performed for the safety set. mITT analysis will apply the analysis strategy of the treatment policy, while PP analysis will apply a while on treatment strategy.<br>***Primary Endpoint:*** the absolute LVEF change to baseline at W16, measured by CMR, will be compared in a confirmatory manner between the verum and placebo group by absolute treatment difference. For primary analysis, we analyse the change in LVEF at W16 compared to baseline between the treatment groups. Superiority of the verum group to improve LVEF compared to placebo will be tested as H0: $\mu_{VERUM} = \mu_{PL}$ vs. H1: $\mu_{VERUM} \neq \mu_{PL}$ (at the 5% significance level in a two-sided manner by an unpaired t-test.<br>***Secondary analysis:*** for all secondary endpoints, appropriate statistical tests (Fisher or Chi Square Test, T-tests, Wilcoxon tests, AN(C)OVA, log-rank, linear contrasts of (G)LMM) may be employed to compare treatment groups at time points of interest. These hypotheses tests will be of explorative nature and tested at a global 5% significance level between treatment groups/factors in a 2-sided manner.

2 LIST OF ABBREVIATIONS

Abs.	Paragraph / Absatz
ADR	adverse drug reaction
AE	adverse event
AMG	"Arzneimittelgesetz", German drug law
AT	anaerobic threshold
BL	Baseline
BMI	Body Mass Index
BP	Blood Pressure
C	Concentration
C°	Degree Centigrade
CAD	Coronary artery disease
CI	Confidence Interval
Cl	Clearance
cm	Centimeter
CMR	Cardiovascular magnetic resonance imaging
CRF	Case Report Form(s)
CRO	clinical research organisation
CSR	clinical study report
CV	cardiovascular
D	Day(s)
DMP	Data Management Plan
DVP	Data Validation Plan
ECG	Electrocardiogram
EC50	Half maximal effective concentration
EQ5D	Quality of Life Questionnaire
e.g.	Exempli gratia; for example
EMB	endomyocardial biopsy
FAS	Full Analysis Set
FBC	Full Blood Count
FDA	Food and Drug Administration (federal authority USA)
FU	Follow-Up
g	Gram
GBCA	Gadolinium based contrast agent
GCP	Good Clinical Practice
GFR	Glomerular filtration rate
GMP	Good Manufacturing Practice
h	Hours
hs	High-sensitive
hsCRP	High-sensitive c-reactive protein
HsTNT	High-sensitive troponin T
Hb1c	Glycohaemoglobin
HDL	High-density lipoprotein
HR	Heart Rate
HF	Heart Failure
IB	Investigator's Brochure
IET-CVI	Institute for Experimental and Translational Cardiovascular Imaging
ICH	International Council for Harmonization of Technical Requirements for Pharmaceuticals for Human Use
ID	Identification
IEC	Independent Ethics Committee
IME	Institute for Molecular Biology and Applied Ecology
IMP	Investigational Medicinal Product
IU	International Unit, measurement for the amount of a substance
IUD	Intrauterine device

DocuSign Envelope ID: 4F4388D5-A568-4FAC-82D1-39CB3AB82D1B

Kg	Kilogram
kPa	Kilo Pascal
L	Liter
LDH	Lactatdehydrogenase
LDL	Low-density lipoprotein
LGE	Late gadolinium enhancement
LKP	“Leiter der klinischen Prüfung”, German co-ordinating investigator
LV	Left ventricular
LVEF	Left ventricular ejection fraction
m	Meter
m^2	Meter squared
MACE	Major adverse cardiovascular events
MCH	Mean corpuscular haemoglobin
MCHC	Mean corpuscular haemoglobin concentration
MCV	Mean corpuscular volume
Min	Minutes
mg	Milligram
ms	milisecond
mL	millilitre
mmHg	Millimeter Mercury
n	Number
Ng	Nanogram
No.	Number
p.a.	Post Application
PASC	Post-acute sequelae of SARS-CoV-2 infection
PASC-CVS	PASC-Cardiovascular Syndrome
PD	Pharmaco dynamic
PK	Pharmaco kinetic
p.o.	Per os
PP	Per protocol
PTS.	Participants
RER	respiratory exchange ratio
Q	Quarter
QA	Quality Assurance
QoL	Quality of Life assessments
RBC	Red Blood Cells
s	second
SAE	serious adverse event
SAP	Statistical Analysis Plan
SCR	Screening
SDV	Source data verification
SmPC	Summary of product characteristics
t	time
TMP	Translational Medicine and Pharmacology
U	Units
ULN	Upper Limit of Normal
V	Visit
V_d	Volume of distribution
VO2max,<br>VCO2max	maximal oxygen uptake<br>maximal carbon dioxide release
W	Week
W.H.O.	World Health Organisation
Y	Year

3 BACKGROUND AND RATIONALE

3.1 Background

3.1.1 Context of disease and participant population

Postacute sequelae of COVID1-9 infection (PASC) are increasingly recognised complications and are defined by lingering symptoms, not present prior to the infection, typically persisting for more than 4 weeks(1). PASC-Cardiovascular Disease (PASC-CVD) denotes the group of prevalently older participants, where ill health in the aftermath of COVID infection relate to the accrued cardiac injury during the acute illness, (1) and burden of pre-existing cardiac conditions. On the contrary, **PASC-Cardiovascular Syndrome (PASC-CVS),** the target population and focus of this study, affects many younger individuals without previous cardiac conditions or relevant comorbidities. These individuals may only have had a mild initial illness but continue to suffer with ongoing cardiac symptoms more than 4 weeks after the acute COVID illness(2,17–19)(**Figure 1**).

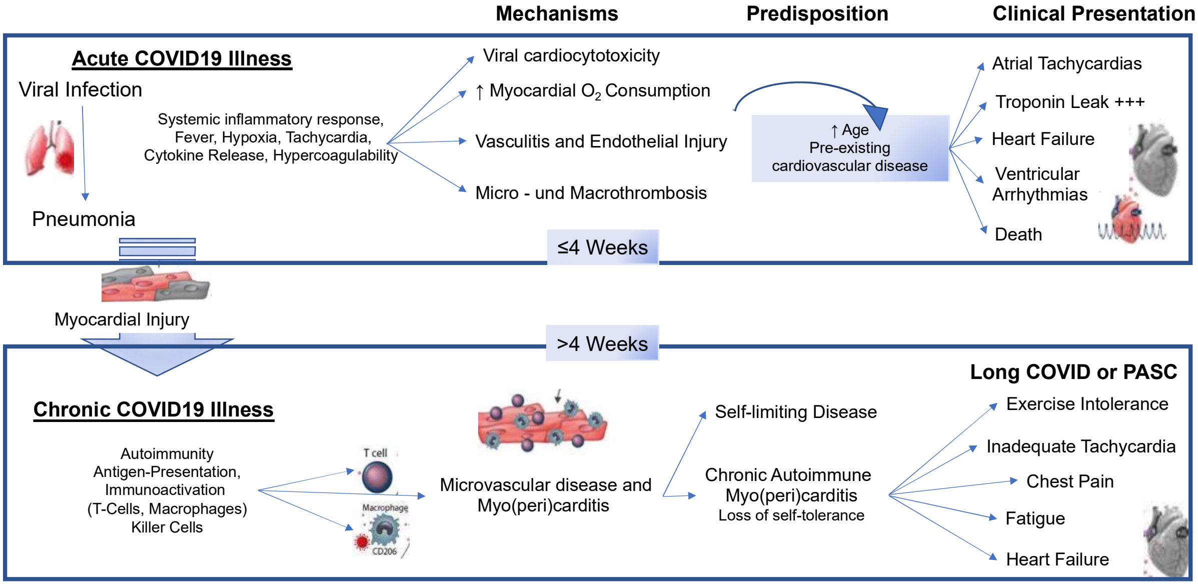

Figure 1. Pathophysiology of COVID19-related cardiovascular involvement.

Symptoms related to the cardiovascular system include exertional dyspnoea, exercise intolerance chest tightness, pulling or burning chest pain, and palpitations(1). Participants may also develop high blood pressure and inadequate/excessive tachycardia, mimicking postural orthostatic tachycardia syndrome, POTS. The cardiac symptoms may be associated with chronic inflammatory manifestations of other organ systems (neurological, musculoskeletal, dermatological, haematological, etc.), and commonly accompanied by chronic fatigue syndrome. Although in most participants the symptoms may be mild and often self-limiting within weeks after infection, they may be persistent in a good proportion of recovered participants, lingering months after the infection without resolution. In small proportion of participants, they may be severe, limiting the activities of daily and professional life. Given the ongoing impact of the COVID-19 pandemic and the chronic character of COVID-related inflammatory heart disease, the potentially high burden of HF in the coming years adds the urgency for early recognition and management.

3.1.2 Pathophysiology of COVID19-related cardiovascular involvement

The underlying pathophysiology of lingering symptoms remains poorly understood and is a subject of intense research. It is increasingly recognised that the weeks immediately after the recovery from COVID-19 infection represent a period of intense systemic disease activity in a considerable proportion of participants. The persisting symptoms may be in part due to worsening of pre-existing conditions, or cardiovascular/pulmonary injury incurred during severe course of disease. However, chronic inflammatory heart involvement affects a broad segment of a previously well population, which may only have had a mild acute illness. In line with our focus on the cardioprotective hypothesis for the intervention to reduce the heart injury specific to the postviral inflammatory heart involvement, we specifically focus on the **subgroup of population with no previously known cardiac conditions**, or evidence of specific heart conditions, prior cardiac symptoms or significant comorbidities (postacute sequelae of COVID infection – cardiovascular syndrome; PASC-CVS).

DocuSign Envelope ID: 4F4388D5-A568-4FAC-82D1-39CB3AB82D1B

COVID-19 related inflammatory cardiac involvement is characterised by chronic myopericardial inflammation, detectable by cardiovascular magnetic resonance (CMR)(1). It is often undetectable by routine diagnostic tests, nor accompanied by significant rise in troponin(1). Studies using CMR imaging in recently recovered, previously well participants have identified changes consistent with non-ischaemic perimyocardial inflammation[22–27], including increased myocardial mapping values and perimyocardial late gadolinium enhancement. Small amounts of pericardial effusion, pericardial adhesions and thin perimyocardial and intramyocardial late gadolinium enhancement is also a common PostCOVID finding. Participants may also have subnormal LVEF, however, profound drops in LVEF or dilated heart cavities, or necrotic or thromboembolic complications do not prominently feature in chronic stage(3). Likewise, areas of substantial necrosis typically found in classical viral myocarditis(4,5), are rare. These abnormalities can be detected as early as 2 weeks after the infection(6,7) and can be observed several months after the infection(8).

Evidence suggests heterogeneous immune responses, sharing the resemblance with post-viral fatigue syndrome and phenotypical signatures of chronic systemic inflammatory conditions. The postviral autoimmune response is thought to result from the disbalance between inflammation, apoptosis and repair(20)(21). Early observations revealed de novo presence of antinuclear antibodies (ANAs) with nucleolar speckled pattern, which persist months after the infection(22–27)(24,28–32). More recently, a study reported on diverse heterogenous tissue-directed immunomodulatory proteins including cytokines, chemokines, complement components and cell-surface proteins(23). Furthermore, increased immunoglobulin (Ig) titres of specific IgA and IgG were reported, whereas more recently, a signature based on total IgM and IgG3 levels, was able to predict the risk of PASC independently of the timepoint of blood sampling(33,34). Autoantibodies against vascular antigens have also been reported(26,35) Haematological manifestations also resemble those observed in autoimmune conditions(36), persisting leukopenia on account of neutron- and lymphopenia increased pro-inflammatory cytokines and immunoglobulins. PostCOVID inflammatory activity in most symptomatic participants is low-grade (significant rise in CRP is rarely observed) (37,38), however, there is a trend towards chronicity.[18,19]The chronic inflammation could also relate to a bystander or virus-mediated activation autoimmunity via autoreactive T and B cells(39).

Although neither primary targets nor exact inflammatory mechanisms are clear at present, the endothelial dysfunction, microvascular disease, and perivascular tissue oedema seem to occur systemically as the pathophysiological commonality(40–48). Studies examining the hearts in recovered, previously well participants have identified subtle heart changes consistent with non-ischaemic perimyocardial fibrosis and/or inflammation(6,8,49–52)using CMR imaging. Small amounts of pericardial effusion and adhesions, and thin perimyocardial and intramyocardial late gadolinium enhancement is also a common postCOVID finding on high resolution late gadolinium enhancement images. Often, this is accompanied with small pleural effusions and pleurisy. These abnormalities can be detected as early as 2 weeks after the infection(6,7) and may persist for several months(8). In contrast to the findings in acute cardiovascular COVID syndrome, thromboembolic complications do not prominently feature in chronic stage(3). Likewise, areas of substantial necrosis typically found in classical viral myocarditis(4,5), are rare. Studies using endomyocardial biopsy (EMB) or autopsies of participants that died during the acute illness (5,53) showed that persistence of viral COVID genome is rarely found, and active replication uncommon(54,55), substantiating the persistent autoimmune, and not viral-induced, pathophysiology of post-acute cardiac manifestations.

In July 2022, we reported on CMR outcomes in 100 participants with mild-moderate acute disease and median 71 days from the diagnosis(8). Compared to risk-factor matched controls, they had higher troponin T, native T1 and T2 mapping values by CMR and a higher rate of myocardial and pericardial LGE, clarifying that recent infection with COVID19 was the underlying mechanism for the observed difference. While 78% of participants showed evidence of cardiac abnormalities CMR, in >20% these abnormalities were significant active inflammation (e.g. T1 or T2 >4SD above the mean of the sequence-specific normal range). More recently, a total of 74% had symptoms (n=346 participants), and in 54% these symptoms persisted beyond 6-months, including persistently reduced exercise tolerance and shortness of breath on exertion. CMR revealed that in those with persistent symptoms, myocardial inflammation was ongoing. Women were more frequently symptomatic than men, with chronic active myocardial inflammation, reduced cardiac volumes (deconditioning) and preserved LVEF(56). This phenomenon is underscored by pathological myocardial remodelling, linking inflammatory injury with accumulation of interstitial fibrosis, increased ventricular and vascular stiffness. Lastly, there is a striking phenotypical resemblance to the cardiac manifestations encountered in chronic systemic inflammatory conditions, such as lupus

DocuSign Envelope ID: 4F4388D5-A568-4FAC-82D1-39CB3AB82D1B

myocarditis(57–60), systemic sclerosis(61,62) or chronic post-viral syndromes, such as Epstein-Barr(63), human immunodeficiency virus(64–66) (65–67), or paediatric COVID-associated multisystem inflammatory syndrome(68–70), further suggesting autoimmune-mediated injury, which may also clarify the gender-differences in immunological responses to COVID19 infection(21,71). These observations cumulatively suggest SARS-Cov-2 infection may be a powerful trigger of autoimmune response, promoting unfavourable and chronic cardiac pathophysiology (72), and increasing the likelihood of poor outcomes.

3.1.3 Current treatment options

There is no specific treatment for myocardial inflammation. However, it is generally agreed that all participants should receive guideline-indicated medical treatment for heart failure as indicated by the clinical presentation (LVEF <45%). This includes ACE inhibitors and/or ARBs, beta blockers, diuretics and aldosterone inhibitors. Clinical trials of immunosuppression in participants with viral myocarditis in advanced stages of heart failure have not shown an improved outcome, however there was an improvement of LVEF with antiremodelling therapy in participants with reduced function (9–12). The benefits of early initiations of antiremodelling therapy to reduce symptoms of exercise intolerance are well recognised(13–15), but not commonly employed outside the contexts of heart failure(16) or hypertension. As most participants with inflammatory heart disease only have mild and nonspecific symptoms and few or no structural abnormalities, they are left untreated (standard of care).

3.2 Rationale

We propose that early intervention with immunosuppression and antiremodelling therapy may reduce symptoms and myocardial impairment, by reducing the disease activity and favourably modify the disease process. Both drugs have been shown to improve endothelial function. Vascular leak due to the endothelial damage are an important therapeutic target in several inflammatory cardiac conditions inducing heart failure. Losartan has been shown to attenuate a number of measures of potential importance in the treatment of heart failure, including cardiac dysfunction, oxidative stress, fibrosis, and inflammatory and cell death signalling pathways in models of heart failure with preserved ejection fraction. Low dose maintenance therapy may help to keep the disease activity at the lowest possible level. The purpose of this study is to examine the efficacy of a combined immunosuppressive/antiremodelling therapy in participants with PASC symptoms and inflammatory cardiac involvement determined by CMR, to reduce the symptoms and inflammatory myocardial injury and thereby stop the progression to HF and death.

3.2.1 Rationale for the choice of investigational medicinal products

Losartan

Renin-angiotensin-aldosterone system (RAAS) plays a key role in the development and progression of cardiovascular disease, especially in arterial hypertension, heart failure and coronary artery disease. ACE inhibitors are the most used and studied type of RAAS blocker and their benefits are due to their neurohormonal modulatory effects, which have vasodilatory, anti-inflammatory, plaque-stabilizing, antithrombotic and anti-proliferative effects. ARBs have similar pharmacological properties to ACE inhibitors but may be better tolerated as dry cough is not a frequent adverse effect. Angiotensin II, a potent vasoconstrictor, is the primary active hormone of the renin/angiotensin system and an important determinant of the pathophysiology of hypertension. Angiotensin II binds to the AT1 receptor found in many tissues (e.g. vascular smooth muscle, adrenal gland, kidneys and the heart) and elicits several important biological actions, including vasoconstriction and the release of aldosterone. Angiotensin II also stimulates smooth muscle cell proliferation. Angiotensin II has been shown to be a potent inducer of oxidative stress via activation of the NAD(P)H oxidase contributing to endothelial dysfunction and vascular injury(73–75). More recently, the role of ACE 2 has been implicated in mediation of the vascular effects of COVID-19, whereby AT2 serves as the virus entry point(76). The protective anti-inflammatory role of Losartan by restoring the ACE 2 levels has been proposed(77,78).

The current ESC guidelines postulate that immunosuppression for myocardial inflammation may be beneficial in selected cases of viral-triggered myocarditis with no evidence of viral activity. The evidence is equivocal.(79) (80) The Cochrane report included 8 eligible RCTs, which were small size, and of poor methodological quality(10). The two largest studies include the EMB-based Myocarditis Treatment Trial (n=111)(9) and TIMIC study (n=85)(81) were performed in participants with advanced heart failure and reduced LVEF<45%. Both studies used high-dose therapy (high dose prednisolone 1.25 mg/kg (with gradual taper for 24 weeks) in combination with either

DocuSign Envelope ID: 4F4388D5-A568-4FAC-82D1-39CB3AB82D1B

azathioprine 1mg/kg or ciclosporin with 200-300ng/ml blood concentration. Mortality between immunosuppression and control arms was not significantly different (RR, 0.93, 95% CI 0.70 to 1.24), **however, LVEF improved in the corticosteroid group compared to the control group** (MD 7.36%, 95% CI 4.94 to 9.79) at 3 months follow-up. More recently, the CANTOS HF sub-study revealed an improvement of outcome for HF endpoints (Hospitalisation for HF, HHF)(82) in participants with known atherosclerotic cardiovascular disease who received canakinumab, an inhibitor of interleukin 1beta. The composite of HHF or HF-related mortality was also reduced by canakinumab, with unadjusted hazard ratios of 1.00 (95% CI, 0.78–1.29) for 50 mg, 0.88 (95% CI, 0.68–1.13) for 150 mg, and 0.78 (95% CI, 0.60–1.02) for 300 mg (P for trend=0.042). **Specifically, for COVID-19 population in the setting of acute COVID-19 illness:** the UK Recovery trial investigated the treatment with low-dose dexamethasone (6 mg OD for up to 10 days, equivalent to 40 mg of prednisolone)(83), leading to reduced 28-day mortality in participants hospitalized for COVID19. The three C study of canakinumab in Covid-19 Cardiac Injury is comparing 2 single, but high doses 300 mg and 600 mg IV in hospitalized participants and highly abnormal cardiac biomarkers. The baseline characteristics for the first 20 randomized participants reveal a predominantly male (75%), the elderly population (median 67 years) with hypertension (80%) and hyperlipidaemia (75%). CRPs and trop T have been markedly elevated (median 16.2 mg/dL and 21 ng/L, respectively), clarifying the focus of these trial to alleviate the acute inflammatory response and reduce the adverse effects of the febrile illness on the heart muscle through tachycardia, hypoxia, and hypercoagulability(84).

In contrast to the above studies conducted in acute COVID illness, our focus is on the participants **with long-COVID syndrome** with ongoing symptoms >4 weeks from the start of the infection. We aim to examine the efficacy of immunosuppressive and antiremodelling therapy to reduce the long-term cardiovascular consequences in participants with CMR evidence of cardiac involvement. Moderation of the immune response by immunosuppressive therapy with prednisolone is a fundamental strategy to prevent the development of chronic immunological pathways, promoting the organ injury and ongoing antigen-presentation events. In long COVID, cardiac biomarkers are less informative regarding the presence of injury; troponin is detectable, but rarely significantly raised. Despite increased pro-inflammatory cytokines and immunoglobulins, **a rise in C-reactive protein is rarely observed**(37,38); unless this is due to the development of anti-CRP antibodies(36), this suggests low-grade chronic inflammation. The cross-play between the development of vascular antibodies and vascular injury and permeability, observed in rheumatological conditions(85), and syndromes of chronic fatigue(86), has been also reported to relate to severity of acute COVID illness(87).

The Post COVID vascular injury(88) induces tissue hypoxia; reinforced by immunothrombosis(89) and inadequate tachycardia it hinders the effective tissue perfusion and sets off the mechanisms of tissue remodelling and repair(90). Pathological remodelling is associated with fibrosis, inflammation and cellular dysfunction (e.g. abnormal cardiomyocyte/non-cardiomyocyte interactions, oxidative stress, endoplasmic reticulum stress, autophagy alterations, impairment of metabolism and signalling pathways), and apoptosis. The antiremodelling effects of renin-angiotensin aldosterone system (RAAS) inhibitors relate to interfering with the reduced endothelial nitric oxide (NO) synthase-derived NO availability, activation of cardiac and leukocyte-dependent oxidant stress pathways, inflammatory pathway activation, matrix-metalloproteinase activation, or stem cell transfer and delivery of novel paracrine factors (91).

Early initiation of Losartan confers vasculoprotective and antiremodelling effect(73–75,92–94), improves myocardial perfusion and exercise tolerance, with a chance of recovery. Losartan has been shown to be effective in patients with functional classes II-IV of the New-York Heart Association (NYHA) by preventing or even reverse cardiac remodelling, which is associated with adverse outcome in s with preserved ejection fraction, such as diabetes, hypertensive and hypertrophic heart disease, where an addition of Losartan improved outcomes.(95–99). Other relevant studies in heart failure with angiotensin type 1 (AT1)-receptor blockers include VALIANT (Valsartan in Acute Myocardial Infarction Trial), Val-HeFT (Valsartan Heart Failure Trial) and CHARM (Candesartan in Heart Failure Assessment of Reduction in Mortality and Morbidity). In the above studies, AT1 receptor blockers also had beneficial effects on cardiac haemodynamics (decreased left and right ventricular filling pressures, reduced total peripheral vascular resistance, increased cardiac output and improved cardiac index), as well as reduced neuroendocrine activation. In the context of myocardial inflammation, PRADA (Prevention of cardiac dysfunction during adjuvant breast cancer therapy) remains the only study to-date to demonstrate a cardioprotective role of candesartan in cancer-therapy-related cardiomyopathy(100). Several studies deployed night-time administration regime to differentiate between the antihypertensive and

DocuSign Envelope ID: 4F4388D5-A568-4FAC-82D1-39CB3AB82D1B

cardioprotective effect. (101) given at night. The night-time regime is especially well tolerated in the population of young participants with no history of high blood pressure. More recently, the role of ACE2 has been implicated in mediation of the vascular effects of COVID-19, whereby AT2 serves as the virus entry point(76). The protective anti-inflammatory role of Losartan by restoring the ACE 2 levels has been proposed(77,78). Antiremodelling therapy (i.e. renin-angiotensin and aldosterone blockers, sacubitril) remains the mainstay therapy in s with symptomatic HF (NYHA II-III), increased NT-proBNP and reduced ejection fraction (≤45%)(16). Due to excessive tachycardia and intravascular volume depletion, a majority of **PASC patients do not show an increase of NT-proBNP**. PASC participants have only minor structural heart abnormalities with subnormal LVEF, hence sensitive diagnostic techniques are required to detect these abnormalities. A formal clinical diagnosis for this pathophysiological entity is currently not available; and it may be established, if successful, based on inclusion/exclusion criteria as a result of this study.
In line with the ESC Prevention of cardiovascular disease programme(102), ACE inhibitors/ARBs belong the group of cardioprotective drugs, with the following indications:

- Hypertension (HTN), alone or in combination with diuretic or calcium-channel blocker
- Heart failure **or asymptomatic left ventricular dysfunction**(103)
- Secondary prevention of coronary artery disease
- Diabetes mellitus and diabetic nephropathy

Current therapeutic indications specific for Losartan (in line with SmPC) include:

1. Treatment of essential hypertension in adults and in children and adolescents 6-18 years of age.
2. Treatment of renal disease in adult participants with hypertension and type 2 diabetes mellitus with proteinuria
3. Treatment of chronic heart failure in adult participants
4. Reduction in the risk of stroke in adult hypertensive participants with left ventricular hypertrophy documented by ECG

Prednisolone

Prednisolone is one of the highly potent glucocorticoid steroids with anti-inflammatory, hormonal and metabolic effects, which are qualitatively similar to those of hydrocortisone, a naturally occurring corticosteroid.

Prednisolone is indicated in the management of systemic inflammatory conditions that benefit from short- or long-term glucocorticoid therapy. Examples of these include (selected with relevance to the proposed clinical indication in the present trial):

1. Arteritis/Collagen disorders: E.g. systemic lupus erythematosus, polymyositis, polymyalgia rheumatica and temporal (giant cell) arteritis, mixed connective tissue disease syndrome, acute rheumatic carditis.
2. Rheumatic disorders: usually given as an adjunctive therapy for short term administration during an acute episode or exacerbation of rheumatoid arthritis, psoriatic arthritis.
3. Skin conditions: life-threatening or incapacitating skin conditions such as pemphigus and exfoliative dermatitis.
4. Gastro-Intestinal disease: ulcerative colitis and regional ileitis (Crohn's Disease).
5. Respiratory disease: sarcoidosis (especially with hypercalcaemia), allergic bronchial asthma, and other bronchospastic conditions.
6. Haematological disorders: various blood dyscrasias eg selected cases of haemolytic anaemia, thrombocytopenic purpura.
7. Miscellaneous: lupus nephritis, nephrotic syndrome.

3.2.2 Rationale for diagnostic approach

Cardiovascular magnetic resonance imaging (CMR) constitutes advanced imaging technology providing in-depth, versatile, accurate and non-invasive means of cardiovascular phenotyping. CMR can inform on spectrum of cardiovascular pathophysiology, as well as dynamic evolution of changes by safe, serial examinations. CMR provides sensitive imaging on the inflammation-related changes in the heart muscle and vascular wall (inflammation, oedema, thickening, scarring), reduced ventricular and vascular deformation and stiffness, as well as vascular wall inflammation. T1 and T2 mapping provide means of quantifiable tissue characterization, which relate directly to the myocardial tissue disease activity and severity[5]. Native T1 is sensitive for detection of abnormal myocardial remodelling processes, whereas T2 indicates the presence of oedema. Together, these two imaging markers help deciphering the predominant driver of signal change as either

DocuSign Envelope ID: 4F4388D5-A568-4FAC-82D1-39CB3AB82D1B

inflammatory (raised native T2) or fibrotic (normal native T2)[5]. T1 and T2 mapping values correlate with histologic evidence of myocardial inflammation, severity of left ventricular (LV) remodelling and longitudinal strain, and activity of myocardial inflammation in participants with systemic inflammatory conditions [4,8,9]. Late gadolinium enhancement is an established imaging technique for visualisation of regional myocardial injury, such as necrosis, oedema, or scar, and instrumental in recognising pericardial involvement. Myocardial perfusion imaging is an established clinical technique to evaluation myocardial blood flow. In this study, the imaging protocol will be based on **IET-CVI Imaging Protocol**, which relate to a highly standardised set of imaging parameters and operating procedures, which are locked, standardised, validated and evidence based. It will provide the basis for imaging tools deployable in the future routine clinical use.

3.2.3 Rationale for Dosage Selection

Losartan

Symptomatic hypotension is the most expected side effect due to Losartan in the present normotensive participant population (104,105). To counter these effects a lower starting dose of 12.5 mg will be used and commenced at night-time. Participants may choose taking the medicaton in several divided doses over the day. Participants will be informed about these possible side-effects and will be supported throughout the dose-adjustement uptitration period. A high fluid intake (2-3 litres per day) and less restrictive/liberal salt intake will be recommended(1). All participants will be provided by a home blood pressure machine and required to measure blood pressure regularly and record the values. No other RAAS antihypertensive or vasodilative treatment will be administered during this study. Concomitant regular use of NSAIDs, including Ibuprofen, will be discouraged.

Losartan (or placebo 1): a total of 50 mg orally/a day, to be taken at nighttime

- the starting dose 12.5 mg
- in all participants, gradual uptitration will be attempted in steps of 12.5 mg every 2 weeks up to a maximally tolerated dose (maximal dose 50 mg).
- we expect the individual maintenance dose to be achieved by week 6.

Prednisolone

Low-dose short-term steroid regime is safe and well-tolerated and prevents the undesirable side effects of mainly associated with the high-dose longterm steroid treatment, including euphoria, hypertension, tachycardia, cushingoid effects, or secondary infections, etc. A low maintenance dose allows good tolerability, while preserving the remission. Fluid retention due to prednisolone may in fact be beneficial to overcoming the intravascular fluid loss, exacerbating the symptoms of excessive tachycardia. Furthermore, we will minimize the occurrence of undesirable effects in the present study. We will use the lowest effective dose will be used for the minimum treatment period. PostCOVID inflammatory activity in most symptomatic participants is low-grade (significant rise in CRP is rarely observed), however, there is a trend towards chronicity.

Prednisolone (or placebo 2): oral, once a day, to be taken in the morning,

- the starting dose of 20 mg a day
- gradual taper by 5 mg every 2 weeks (week 1-2: 20 mg, week 3-4: 15 mg, week 5-6: 10 mg, week 6-16: 5 mg once a day (maintenance dose).

3.3 Benefit/Risk Assessment

3.3.1 Potential Benefits

Corticosteroids provide symptomatic treatment by virtue of their anti-inflammatory effects; they are never curative. The intended use is to reduce the activation of the immune system in response to the COVID-19 infection and prevent widespread cardiovascular injury. Therapeutic intervention is needed to minimize damage from the uncontrolled disease.

3.3.2 Important known and potential risks

Losartan has been in active clinical use in the U.S. since 1995 and in Germany since 1996. The precations for use and contraindications are well established, and pateints will be screened for these conditions prior to enrolment into the study (hypersensitivity, angioedema), pregnancy (Losartan is contraindicated in 2nd and 3rd trimester of pregnancy) or breastfeeding. Women of childbearing potential will be advised to use a reliable form of contraception (Pearl index of less than 1) for the duration of the study; examples may include sterilisation, birth control pill, intrauterine device. For more information on highly effective contraception methods please refer to Appendix

DocuSign Envelope ID: 4F4388D5-A568-4FAC-82D1-39CB3AB82D1B

18.2.13. Participants actively breastfeeding will not be included in this study. Other listed precautions (electrolyte imbalance, hyperkalaemia, hepatic impairment, renal impairment, heart failure with concomitant renal impairment, significant valvular disease, hypertrophic cardiomyopathy) relate to the special populations with comorbidities that will not be included in this study. Blood tests will be conducted prior to commencement of Losartan to screen for renal and hepatic impairment.

Prednisolone received its marketing authorisation in 1955 for treatment of rheumatoid arthritis and has been in active used since. The precations for use and contraindications are well established, and pateints will be screened for these conditions prior to enrolment into the study. The incidence of undesirable effects, including HPA-axis suppression, is predictable and correlates with the relative potency of the drug, dosage, the timing of administration and the duration of treatment. They are primarily encountered with chronic use of high doses (daily doses of more than 0.5 - 1 mg/kg, which will not be used in the current study). Prednisolone in higher doses may cause:

- Psychological symptoms: irritability, euphoric, depressed, and labile mood), psychotic reactions (including mania, delusions, hallucinations, and aggravation of schizophrenia, behavioural disturbances, irritability, anxiety, sleep disturbances, and cognitive dysfunction including confusion and amnesia have been reported. Participants with history of psychiatric disorders will not be included in the current study).
- Susceptibility to infections and their severity due to suppression of the inflammatory response. Short-term high intensity immunosuppressive therapy may increase the incidence of cutaneous candidiasis; however, severe systemic infections are exceedingly rare. Participants with known chronic systemic infection or immunocompromise will not be included.
- Corticosteroids may worsen diabetes mellitus and hypertension, especially in overweight participants (participants with BMI ≥ 35 kg/m^2 or ≥125kg bw will not be included in the current study).
- Corticosteroids may worsen osteoporosis. Because prednisolone is intended as a short-term use only, formal osteoporosis prophylaxis is not necessary. Participants will not be advised to take vitamin D supplements for the duration of 16-week intervention.
- Corticosteroids may cause fluid retention, especially in participants with renal insufficiency or congestive heart failure, who will not be included in the present study. Participants with previous steroid use and complications, including previous steroid myopathy, peptic ulceration, hypothyroidism, glaucoma etc) will be excluded.

Participants will be closely monitored for these symptoms and will undergo regular interim clinical assessments in W2, W6 (onsite or at the general physician) and W12.

3.3.3 Assessment of Benefit and Risk

Given the low doses and close monitoring, we believe that the benefits outweigh the risks of any potenial harm and justify the conduct of this study. We also provide a risk management plan for possible symptoms and measures to counter these. All participants will be closely monitored for any side effects.

4 Study Objectives

4.1 Efficacy

4.1.1 Primary efficacy objective

The primary objective is to determine the efficacy of a combined immunosuppressive and antiremodelling therapy in COVID-19 related inflammatory cardiovascular involvement by CMR to reduce inflammatory myocardial injury compared to placebo.

4.1.2 Primary efficacy endpoint

The primary endpoint of this study is absolute LVEF change to baseline at W16, measured by CMR, compared between the verum and placebo group by absolute treatment difference

4.1.3 Secondary efficacy objectives

The secondary objectives are to determine the efficacy of a combined immunosuppressive and antiremodelling therapy in COVID-19 related inflammatory cardiovascular involvement by CMR to reduce inflammatory myocardial injury compared to placebo by improvement in other clinical parameters.

4.1.4 Secondary efficacy endpoints

The secondary endpoints include the following continuous endpoints, where "changes" refer to the difference between baseline and W16 (BL, absolute and in %):

- Mean LGE extent (%) and change thereof compared to BL
- CPET (achieved Work Rate, VO_2max, VCO_2 max, RER, AT, slope) and change thereof compared to BL
- Mean T1 and T2 values (ms) and change thereof compared to BL
- Mean LV and RV volumes (ml/m2) and LV mass (g/m2) as well as derived parameters and change thereof compared to BL
- Mean Myocardial strain (%) and change thereof compared to BL
- Mean Pulse wave velocity (m/s) and change thereof compared to BL
- Aortic wall thickness (LGE, mm); and change thereof compared to BL
- Average Symptom Score and change thereof compared to BL
- Compliance: Frequency of prescribed medication consumed, participant diary and drug adherence, total cumulative steroid dose;
- Tolerance: number of participants who required dose reduction or treatment cessation due to side effects, especially due to
    - Hypotension
    - Unblinding due to emergency safety issues
- Number of Responders by achieving:
    - partial response: a normal CMR result is defined as normal T1 and T2, normal gender-age predicted LVEF, non-dilated LV
    - total response: in addition to the above absence of LGE
- Proportion of participant with HF or MACE after 1 year
- 1- year Event-free survival

4.2 Safety endpoints

4.2.1 Safety objective

The primary safety objective is to demonstrate that a combined immunosuppressive and antiremodelling therapy in proposed doses in this participant population is safe.

4.2.2 Safety Parameters

Safety parameters include the number or frequency, severity, and number of adverse events (AE), serious AE (SAEs), defined by:

- Proportion of participants with serious infectious complications (fever ≥38.5°C, accompanied by rise on hsCRP, neutrophilia, lymphocytosis, septic shock, need for antiviral or antibiotic treatment)
- Proportion of participants with symptomatic hypotension (dizziness, blackouts, and systolic BP<90 mmHg) or bradycardia (heart rate <40/min)
- Proportion of participants with a significant rise in cardiac biomarkers (hsTNT, NTproBNP, >3-times the BL)
- Proportion of participants with a significant drop in eGFR compared to BL (>25%) compared to BL
- Proportion of participants developing hypertensive crisis

DocuSign Envelope ID: 4F4388D5-A568-4FAC-82D1-39CB3AB82D1B

4.2.3 Study Drug Levels
Study drug levels are well established and will not be performed within this study.

5 Study Population

5.1 Enrolment and study centres
6 study centres will participate in this study (3 Germany, 1 Austria).

5.2 Target Population
Participants recently recovered from COVID-19 infection, experiencing cardiac symptoms in the aftermath of COVID-19 infection, with CMR evidence of myocardial inflammation and/or remodelling (as defined in inclusion criteria) and no previously known cardiovascular disease (**PASC-CVS**). A formal clinical diagnosis or an ICD code for this pathophysiological entity is currently not available. Participants will be recruited consecutively with no preference for men or women. However, pregnant, or breast-feeding women will be excluded.

5.3 Inclusion Criteria
- Participants ≥ 18 years
- Participants with recent COVID19 infection (> 4 weeks), details in 6.1.
- PASC Syndrome, defined by persistence or new symptoms, not present prior to the infection.
- CMR evidence of inflammatory cardiac involvement at BL by any of the following criteria:
  - Increased native T1≥ 1130 ms at 3.0 Tesla (or 1030 ms at 1.5 Tesla) and/or;
  - Increased native T2 ≥39.5 ms at 3.0 Tesla (or 49.5 at 1.5 Tesla) and/or
  - present non-ischaemic myopericardial LGE and/or;
  - LVEF ≥45 - ≤50%.
- Willingness to comply with the study procedures and study protocol.

5.4 Exclusion Criteria
- Severe course of acute COVID illness requiring hospitalisation
- Known allergy to or intolerance of the study medications
- Symptomatic hypotension (systolic blood pressure less than 90 mm Hg), not reversible with oral hydration
- Any previous or current use of ACE inhibitors, AR Blockers
- Any previous oral prednisolone, or any other immunosuppressive or biological treatment (within 10 weeks)
- History or CMR evidence of preexisting heart disease, including:
  a. Known cardiac impairment with LVEF ≤44%
  b. Congestive heart failure (NYHA III-IV)
  c. Active heart failure treatment
  d. Established ischaemic heart disease, peripheral arterial disease and/or cerebrovascular disease
  e. Persistent or permanent atrial fibrillation or significant heart rhythm abnormalities.
  f. Congenital or clinically relevant valvular heart disease (moderate or severe)
  g. Specific cardiomyopathy (hypertrophic, hypertensive heart disease, amyloidosis, previous myocarditis, non-ischaemic dilated cardiomyopathy, arrhythmogenic right ventricular cardiomyopathy, non-compaction cardiomyopathy, etc).
  h. Known significant concomitant diseases that are likely to interfere with the evaluation of the participant’s safety and of the study outcome (e.g. diabetes, lung or hepatic disease, epilepsy, psychiatric disorders, renal disease with a current estimated GFR <30 mL/min/1.73 m² using MDRD formula, chronic systemic infection or immunocompromise)
  i. Exceeding scanner bore and table-holding capacity: Weight >125 kg, BMI > 35 kg/m^2
- Contraindications to contrast-enhanced CMR imaging, e.g.
  a. MR-unsafe implantable device
  b. known allergy to gadolinium-based contrast agent (GBCA)
- For female participants:

DocuSign Envelope ID: 4F4388D5-A568-4FAC-82D1-39CB3AB82D1B

a. Pregnant or lactating women
b. Women of childbearing potential not willing to use effective contraception (as defined in18.2.13.

- Known alcohol, drug, or chemical abuse
- Participants currently participating in an investigational study or for whom participation is planned.
- Unable to provide written informed consent

Participants with CMR evidence of structural heart disease or incidental heart rhythm abnormalities will be advised to see their own doctor for further investigations.

5.5 Justification of inclusion criteria

CMR based myocardial mapping provides means of quantifiable tissue characterization, which relates directly to the myocardial disease activity and severity (106,107). Native myocardial mapping values relate to myocardial tissue remodelling processes due to co-existing or pre-existing inflammation. T1 and T2 mapping values correlate with histologic evidence of myocardial remodelling and inflammation, the severity of left ventricular (LV) remodelling and longitudinal strain, and activity of myocardial inflammation in participants with systemic inflammatory conditions. Native myocardial mapping values track the response to anti-inflammatory treatment, which is paralleled by a reduction in C-reactive protein level. Myocardial native T1 and T2 values were found significantly and prevalently elevated in several disease models of systemic inflammation, including SLE, RA, systemic sclerosis, and sarcoidosis participants, irrespective of the presence of symptoms, age, or disease duration. In these conditions, native T1 the strongest independent discriminator between healthy myocardium of controls and participants with systemic disease, underscored by only marginally improved prediction of outcomes by addition of late gadolinium enhancement (LGE).

DocuSign Envelope ID: 4F4388D5-A568-4FAC-82D1-39CB3AB82D1B

6 Study Design

6.1 Summary of Study Design

This is a prospective multicentre, randomised, double-blind, placebo controlled clinical trial the efficacy of immunosuppressive and antiremodelling therapy will be compared to placebo in participants with COVID-19-related cardiac involvement determined by CMR imaging.
Participants with laboratory evidence of recent COVID-19 infection (>4 weeks, defined as > 28 days from the date of the clinical diagnosis) and cardiac symptoms, not present prior to infection, will be screened for eligibility. Participants will have to provide a confirmatory evidence of severe acute respiratory syndrome coronavirus 2 (SARS-CoV2) infection by detection of ribonucleic acid in swab test of upper respiratory tract or antigen test using an approved method. Given the discontinuation of systematic public testing, home antigen tests, sickness note, or evidence of antibody response, a doctor's sickness notice for the COVID illness can also serve as a proof to a prior infection. Chest pain, dyspnoea, palpitations, syncope are considered cardiac symptoms. Written informed consent will be obtained from all participants prior to undergoing baseline assessments (CMR, clinical assessments with standardised questionaries for symptoms, ECG, CPET, blood tests for laboratory assessments and emerging biomarker analyses). Biobanking blood samples will be stored at -80°C until usage. CPET will also be performed, subject to local availability.
Participants fulfilling inclusion and exclusion criteria will be randomised equally (1:1) into the combined verum and combined placebo arm. A total 280 participants (140 per treatment group) will be randomised.

In the Verum arm:

Losartan + Prednisolone

In the Placebo arm

Placebo 1 + Placebo 2 (corresponds to the standard-of-care)

Losartan (or placebo 1): a total of 50 mg orally/a day, to be taken at nighttime
- the starting dose 12.5 mg
- in all participants, gradual uptitration will be attempted in steps of 12.5 mg every 2 weeks up to a maximally tolerated dose (maximal dose 50 mg).
- we expect the individual maintenance dose to be achieved by week 5.

Prednisolone (or placebo 2): oral, once a day, to be taken in the morning,
- the starting dose of 20 mg a day
- gradual taper by 5 mg every 2 weeks (week 1-2: 20 mg, week 3-4: 15 mg, week 5-6: 10 mg, week 7-16: 5 mg (maintenance dose).

Prior to commencement of the study medication, participants will receive full instruction about the medication regime. They will be explained about the uptitration of Losartan and taper of Prednisolone. Study treatment will be initiated in the morning and the evening of Day 1, after all baseline assessments are completed and the participant has been randomized. Participants will pursue upitration of Losartan. If the next higher dose after each study drug increase is not tolerated, the dose will be reduced to the previous tolerated dose.
Participants will be followed up by 2 remote visits at W2 and W12 while on therapy and an onsite (with general practitioner) safety visit at W6 (tolerance ± 10days). Additional follow-ups may be arranged for, as required. Primary objective will be assessed at an onsite visit after completion of W16.

6.2 Follow-up

The treatment period will be 16 weeks (W0-W16). SCR/BL and W0 may be up to 6 weeks apart. Additionally, data relevant for analysis of outcome endpoints will be collected as described in Section 9.2.11 at 52 wks (±14 days) (1 years, visit 6). Interim visits can be early/delayed by a maximum +/- 10 days for visits 1 – 3; delays will not be regarded as protocol deviation. For Visit W16(V4) of a maximum of +/- 14 days and visit 5 14 days are permitted and not regarded as a protocol deviation.

Drug titration will be dependent on investigator assessment of participant's symptoms and interrogation of participant's blood pressure diary.
Final efficacy assessments will take place after 16 weeks of study treatment and include
a second CMR, a clinical and laboratory assessments. If locally available, CPET will be performed. Frozen blood samples will be retained for emerging biomarker analyses.

6.3 Flowchart – Study Design

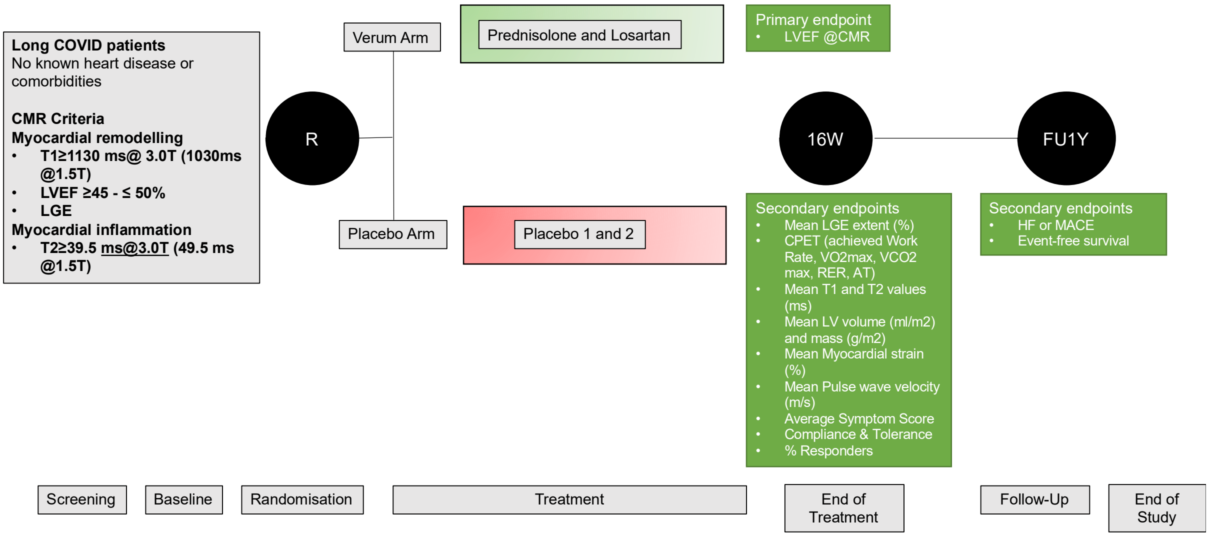

6.4 Randomization and Blinding

6.4.1 Randomization

A computer prepared randomization list will be generated by an unblinded person authorized by the Sponsor. The randomisation register will only be accessible to the authorised personnel.

Randomization is intended to limit the occurrence of (un-)conscious bias in the conduct and interpretation of clinical trials by balancing out (un-)known participant characteristics within each group. The random allocation of the next participant should be concealed to those allowed to request the randomization of a participant. Concealment of the randomisation aims to avoid (selection) bias arising from the knowledge of the upcoming treatment allocation.

6.4.2 Blinding

The study will be performed in a double-blind fashion. This means that the investigator, the site staff, the participants, the sponsor personnel and all other individuals involved in the study will remain blinded to treatment allocation until database lock, with the exception of individuals who are responsible for generating randomization lists and clinical supply depot/distribution. People who are fully or partially unblinded will be clearly identified and their unblinding status will be documented in the trial master file.

The blinding will continue to the end of the study, with unblinding of the statistician-only at 16W for all required analyses at that point. This will preserve the blinding to the group allocation for future analyses (follow-up at 1).

6.4.3 Unblinding for Suspected Unexpected Serious Adverse Reactions (SUSAR)

If a SUSAR occurs in a participant taking part in the study, the Drug Safety Manger will perform unblinding of the treatment allocation for that participant in order to meet regulatory requirements. The unblinded information will only be accessible to individuals who are responsible for expedited safety reporting to regulatory authorities, ethics committee and to the DSMC. All other people involved in the study, will remain blinded.

DocuSign Envelope ID: 4F4388D5-A568-4FAC-82D1-39CB3AB82D1B

6.4.4 Emergency Procedures for Unblinding

Unblinding of subject´s treatment assignments may take place in a medical emergency when it appears necessary to ensure the subject's safety and would be instrumental in further treatment decisions.
The decision to unblind resides solely with the investigator and must be documented and justified. After unblinding, the participant must be discontinued from study medication.
Investigators are encouraged to discuss with the Project Medical Officer if they believe that unblinding is necessary. The investigator should make every attempt to contact the Sponsor before unblinding any participant's treatment assignment but must contact the Sponsor within 24 hours after the event and explain any premature unblinding of the IMP (e.g., accidental unblinding, unblinding due to a serious adverse event).
Unblinding will be performed using emergency envelopes with restricted access to qualified personnel at sites. The unblinding of subject's treatment has to be documented with date, time and initials of unblinding investigator and with reason for opening. If appropriate an emergency unblinding of subject´s treatment assignment will be documented on Serious Adverse Event Form /Serious Adverse Event CRF.
In case of accidental unblinding, all of the above procedures should be followed, although a Serious Adverse Event Form /Serious Adverse Event CRF page should not be completed. The unblinding details should be documented and filed in subject's medical records, CRF, ISF and in project file. The opened emergency envelope is to be archived in the ISF. Following unblinding, decisions for further proceedings with participant will be at the sponsor´s discretion.

6.5 Study medication

6.5.1 Active Study Medication

**Active substance**	**Losartan**
Dosage form	Capsules à 12.5 mg and 25 mg
Starting dose	12.5 mg once a day
Taper regime	gradual increase in doses of 12.5mg every 2 weeks maximally tolerated dose
Maintenance dose	Maximal 50 mg
Duration of treatment	Maximum of 16 weeks
Mode of application	Oral
Time of application	Evening (0-0-1), or in divided doses (1-0-1)
Storage	Storage in a dry place at room temperature (≤ 25 °C)

Guidance – as tolerated: Gradual increase by 12.5 mg every 2 weeks (week 1-2: 12,5 mg, week 3-4: 25 mg, week 5-6: 37.5 mg, week 7-16: 50 mg (maintenance dose).

**Active substance**	**Prednisolone**
Dosage form	Capsules à 5 mg and á 10 mg
Starting dose	20 mg once a day
Taper regime	Taper by 5 mg every two weeks
Maintenance dose	5 mg
Duration of treatment	Maximum of 16 weeks (1-0-0)
Mode of application	Oral
Time of application	Morning dose (1-0-0)
Storage	Storage in a dry place at room temperature (≤ 25 °C)

Guidance – as tolerated: Fixed taper by 5 mg every 2 weeks (week 1-2: 20 mg, week 3-4: 15 mg, week 5-6: 10 mg, week 7-16: 5 mg (maintenance dose).

6.5.2 Placebo

The placebo contains all of the inactive ingredients and none of the active ones. Placebo will match active study drug in smell, taste, colour and appearance to assure proper blinding.

Matching Capsules of Losartan and Prednisolone.

**Substance**	**Placebo 1**
Dosage form	Look-alike Capsules à 12.5 mg and 25 mg
Starting dose	12.5 mg once a day

Taper regime	gradual increase by 12.5 mg to maximally tolerated dose every 2 weeks
Maintenance dose	Maximal 50 mg
Duration of treatment	Maximum of 16 weeks
Mode of application	Oral
Time of application	Evening (0-0-1), or in divided doses (1-0-1)
Storage	Storage in a dry place at room temperature (≤ 25 °C)

**Substance**	**Placebo 2**
Dosage form	Look-alike Capsules à 5 mg and á 10 mg
Starting dose	20 mg once a day
Taper regime	Taper by 5 mg every two weeks
Maintenance dose	5 mg
Duration of treatment	Maximum of 16 weeks (1-0-0)
Mode of application	Oral
Time of application	Morning dose (1-0-0)
Storage	Storage in a dry place at room temperature (≤ 25 °C)

6.6 Concomitant Medication and Treatment

Concomitant medication is non-investigational medical product, given to clinical trial participants as required in the protocol as part of their standard care for a condition, which is not the indication for which the IMP is being tested, and is therefore not the object of the study. All concomitant medication(s) administered must be reported in the case report form.
Specifically:
Treatment with **paracetamol** or **ibuprofen** per needed basis (no regular therapy) as simple analgetic will be permitted.
Treatment with **colchicine** will **not** be permitted during the study period, due to confounding effects in placebo/prednisolone arm. Should pericarditis symptoms persist beyond the study period, colchicine can be administered after the completion of the study.
In participants with palpitations or excessive tachycardia (HR>85/min at rest), addition of low dose **Bisoprolol** (1.25mg- 2.5 mg orally once a day) or **Ivabradine** (2.5 -5 mg orally twice a day) may be considered(108).(1)
Prior COVID Vaccination is not an exclusion criterion. Any vaccination during the treatment period will not be permitted.
Initiation of new supplement therapy, including multivitamins, containing vitamin B, D, C and Coenzyme10 will be discouraged for the duration of the treatment period of this study (16 weeks).
In case of acute COVID infection during the treatment period, Paxlovid therapy will be permitted in line with the evidence.(109)(110)
For prevention of pregnancy during the trial period **highly effective methods of contraception** will be mandated in women of childbearing potential (WOCBP), as defined in 18.2.13. For the purpose of this document, a woman is considered of childbearing potential, i.e. fertile, following menarche and until becoming post-menopausal unless permanently sterile. Permanent sterilisation methods include hysterectomy, bilateral salpingectomy, and bilateral oophorectomy. A postmenopausal state is defined as no menses for 12 months without an alternative medical cause. A high follicle-stimulating hormone (FSH) level in the postmenopausal range may be used to confirm a post-menopausal state in women not using hormonal contraception or hormone replacement therapy. However, in the absence of 12 months of amenorrhea, a single FSH measurement is insufficient.

DocuSign Envelope ID: 4F4388D5-A568-4FAC-82D1-39CB3AB82D1B

7 Study Plan

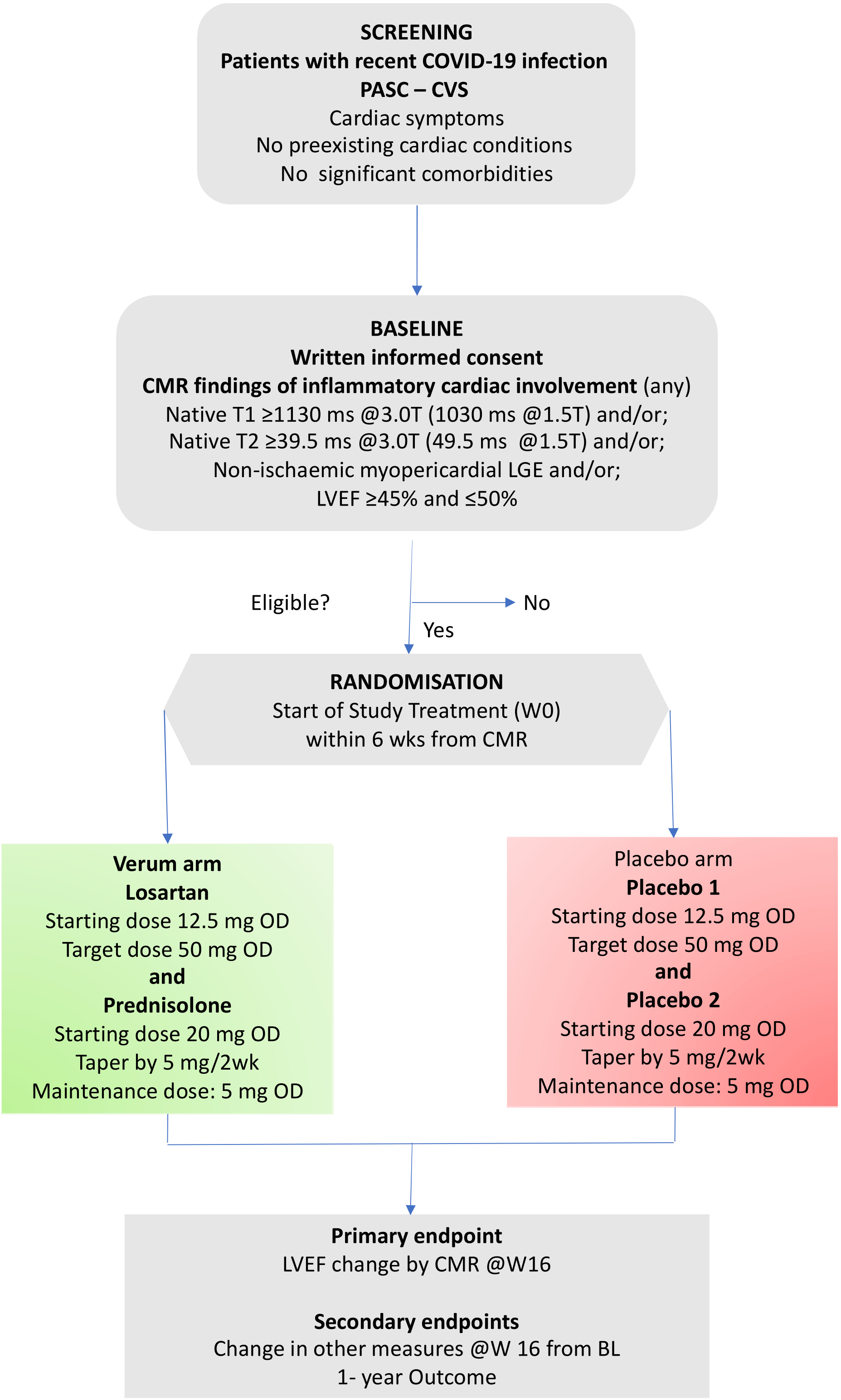

DocuSign Envelope ID: 4F4388D5-A568-4FAC-82D1-39CB3AB82D1B

8 Investigational Medicinal Products

8.1 Investigational Medicinal Product (IMP)

The IMPs in this study are Losartan/Prednisolone or their respective placebo therapy.

8.2 Administration, Dosage and Duration of Treatment

The study medication will be administered orally according to the treatment schedule. Schedules of Prednisolone taper and Losartan uptitration are foreseen for treatment and placebo arm. These proposed schedules are considered as guidelines, which can be modified (see below) in case of intolerance.

Losartan (0-0-1) at night
Week 1-2: 12.5 mg once a day
Week 3-4: 25 mg once a day
Week 5-6: 37.5mg once a day
Week 7-16: 50 mg once a day

Prednisolone (1-0-0) in the morning
Weeks 1-2: 20 mg once a day
Weeks 3-4: 15 mg once a day
Weeks 5-6: 10 mg once a day
Weeks 7-16: 5 mg once a day (maintenance dose).

We suggest 8:00 am for admiration of prednisolone and 21:00 as the optimal time for administration of Losartan.
Specifically for Losartan: if the next higher dose is not tolerated, the participant should return to the last tolerated dose. Alternatively, participants may be asked to consider changing their daily regime to divided doses for 1 week. The highest tolerated dose should be administered until the end of the treatment period (for a total of 16 weeks post randomization).
Specifically for Prednisolone: if the starting dose of 20 mg leads to excessive sinus tachycardia, (defined as resting heart rate of more than 90 bpm), the dose can be reduced to 10 mg, and continued unchanged for up to 6 weeks.
If a dose of IMP is missed: Losartan/Placebo 1 should be administered as soon as possible on the same evening, otherwise omitted till the following evening. Prednisolone/Placebo 2 can be taken up to 14:00 on the same day, or otherwise omitted till the following morning. Evening administration of prednisolone must be avoided. Thereafter, participants can resume dosing on their usual day of administration. It is not necessary to administer two doses on the same day.
All interim changes to the regime or omitted/ missed doses will be documented in a note to file.
In the unlikely event that the study drug requires replacement or a need for a top-up of an already dispensed drug, Heidelberg pharmacy will be permitted to ship the study drug, which is equivalent to the original group allocation, directly to the participant using the temperature-controlled transport. A permission to share contact details with the pharmacy to undertake the transport arrangements will be obtained from the participant.

8.3 Study Treatment Return and Reconciliation

8.3.1 Drug Accountability

It is the responsibility of the investigator to ensure that detailed treatment dispensing log of study medication is maintained at each study site. The study medication shall be inventoried at each monitoring visit. After completion of the study, including final drug accountability, all unused study materials must be destroyed on site or returned to pharmacy for destruction.
The participant's diary and the number of opened Blisters will be checked at every visit. Drug accountability will be performed for all open blisters. Participants will be instructed to return any unused medication at week 16.

Records or logs must comply with applicable regulations and guidelines, and should include:

- Amount received and placed in storage area.
- Amount currently in storage area.

- Label ID number or batch number and use date or expiry date.
- Dates and initials of person responsible for each study medication inventory entry/movement.
- Amount dispensed to each participant, including unique participant identifiers.
- Amount transferred to another area/site for dispensing or storage.
- Non-study disposition (e.g., lost, wasted, broken).
- Amount destroyed at study site.

8.3.2 Drug destruction

Participants will be instructed to return all unused study medication in its original packaging together with their medication diary at on-site visits to examine study treatment accountability and compliance.
For this study, study drugs may be destroyed on site provided the following minimal standards are met:

- On-site disposal practices must not expose humans to risks from the drug.
- On-site disposal practices and procedures are in agreement with applicable laws and regulations, including any special requirements for controlled or hazardous substances.
- Written procedures for on-site disposal are available and followed. The procedures must be filed with the Sponsor SOPs.
- Records are maintained that allow for traceability of each container, including the date disposed of / delivered, and quantity disposed. The method of disposal, i.e., incinerator, licensed sanitary landfill, or licensed waste disposal vendor must be documented.
- Accountability and disposal records are complete, up-to-date, and available for the sponsor or its delegate to review throughout the clinical trial period as per the study agreement.
- Any unused study drugs can only be destroyed after being inspected and reconciled by the responsible Study Monitor.
- It is the Investigator’s responsibility to arrange for disposal of all empty containers, provided that procedures for proper disposal have been established according to applicable federal, state, local, and institutional guidelines and procedures, and provided that appropriate records of disposal are kept.

8.4 Supply, Packaging, Labelling and Storage

8.4.1 Supply, Packaging

The study drugs will be provided by Pharmacy in form of Capsules for the duration of the treatment period.

8.4.2 Labelling and Storage

The labels will contain the information in German, according to national law. All packaging and labelling operations will be performed according to GMP and GCP rules. Investigational site dispensary staff will dispense the IMPs according to the protocol.
All clinical drug supplies are to be stored in a secure, limited-access area in accordance with the following storage conditions: Room temperature (≤ 25 °C), avoiding sub-zero temperatures.
The study supplies need to be protected from light (to be kept in closed cartons or boxes).

DocuSign Envelope ID: 4F4388D5-A568-4FAC-82D1-39CB3AB82D1B

.

9 Conduct of The Study

9.1 Scheduling of Study Procedures

Participants recovered from COVID-19 infection (>4 weeks), experiencing cardiac symptoms in the aftermath of COVID-19 infection, and no previously known cardiovascular disease (PASC-CVS) will be screened for eligibility prior to baseline visit to ascertain the CMR evidence of myocardial inflammation and/or remodelling (as defined in inclusion criteria).

Registration

Participants will be able to register their interest to participate in the study via a **secure online portal**, set up by the Institute of Experimental and Translational Cardiovascular Imaging. Alternatively, they will be able to contact the recruitment staff via phone. By submitting the form candidates will confirm their agreement to be contacted in line with GDPR.

Screening

A minimal set of information will be provided by the participants by filling out a standardised questionnaire on symptoms, medical history, and medications, date and type of COVID-19 diagnosis, COVID vaccination, inclusion and exclusion criteria, and contraindications to cardiac magnetic resonance imaging (Screening Questionnaire – Appendix 18.2.1). Participants will confirm their agreement with the data collection in line with GDPR. The screening questionnaire will serve the purpose of eligibility assessment. The pseudonymised data of all participants undergoing screening procedures will be listed in the Participant Screening Log and included in the Screening Form of eCRF.

Baseline Assessments (-6wks to Day 0)

The following evaluations will be performed at the baseline:

- Written informed consent
- Clinical Assessments
  - Demographics and Medical History
  - Concomitant Medication
  - Symptoms Scores (Questionnaires: Modified Canadian Chest pain scale, NYHA, MRC Dyspnoea scale, Long COVID Questionnaire);
  - QoL Questionnaire (RAND 36-Item Health Survey Version 2.0)
  - Clinical Assessments: vital signs, including heart rate (HR), blood pressure, height, and weight.
- Laboratory assessments (approximately 50 ml venous blood):
  - Local laboratory assessments (1 small EDTA; 1 serum, 1 coagulation): Full blood count, blood biochemistry (Na, K, creatinine/eGFR (MDRD), liver function tests (bilirubin, AST/ALT/GGT/Alkaline Phosphatase), CRP, troponin, NTproBNP, D-dimer, Fibrinogen; Lipid profile, HbA1c, TSH, rheumatology screen (ANA, ENA, ANCA, complement C3, C4)
  - Biobanking samples ((2 big EDTA 8 ml for plasma extraction, 2 small EDTA 4 ml for whole blood freezing, 2 big Serum probes)) will be retained and stored at -80 °C for measurement for future measurements (OLINK analyses for proteomics genomics, metabolomics)
  - pregnancy test in all females
- 12 lead ECG (intervals (ms): PQ; QRS, QT)
- Cardiac magnetic resonance imaging (Cardiac volumes, LVEF, RVEF, LV mass, LV strain myocardial mapping, myocardial late gadolinium enhancement, aortic wall imaging (LGE), central aortic pulse wave velocity)
- CPET (in participants selected for randomisation, if locally available): achieved Work Rate, VO_2max, VCO_2 max, RER, AT, slope.
- Assessment of AEs

Randomisation and start of the study medication (Day 0, W0):

- All baseline assessments have been completed
- All eligibility criteria have been met
- Within 6 weeks from baseline CMR assessment
- Introduction into treatment schemes and treatment diary keeping

DocuSign Envelope ID: 4F4388D5-A568-4FAC-82D1-39CB3AB82D1B

- Introduction into Blood pressure device (CE certificate in Appendix 18.2.11) and home-based BP measurements/BP diary keeping

Interim Video Evaluation (W2, W12; ± 10 days; any additional visits)

- Remote consultations per Videolink
- Vital signs: BP and HR Diary
- Symptoms Scores (Questionnaires: Modified Canadian Chest pain scale, NYHA, MRC Dyspnoea scale, Long COVID Questionnaire)
- Transfer of the participant's BP and IMP diaries.
- Assessment of compliance and tolerance:
  - Recording of changes in concomitant medications
  - Study drug dose adjustment and accountability
- Assessment of adverse events

Interim assessment (W6) ± 10days; onsite or remote assessment, with participants' conducting the safety blood test and ECG at their registered general practitioners and the results to be evaluated by the site investigator.

- Clinical Assessments
  - Symptoms Scores (Questionnaires: Modified Canadian Chest pain scale, NYHA, MRC Dyspnoea scale, Long COVID Questionnaire);
  - QoL Questionnaire (RAND 36-Item Health Survey Version 2.0)
  - Clinical Assessments: vital signs, including heart rate (HR), blood pressure, height, and weight.
- Laboratory assessments (approximately 50 ml venous blood)
  - Local laboratory assessments (1 small EDTA; 1 serum, 1 coagulation): Full blood count, blood biochemistry (Na, K, creatinine/eGFR (MDRD), CRP, troponin, NTproBNP, D-dimer, Fibrinogen;
  - Biobanking samples (2 big EDTA 8 ml for plasma extraction, 2 small EDTA 4 ml for whole blood-freezing, 2 big Serum probes) will be retained and stored for measurement for future measurements (OLINK proteomics analyses: Target 48/96 Cytokines; genomics, metabolomics)
- 12 lead ECG (intervals (ms): rhythm, axis, PQ; QRS, QT
- Transfer of the participant's BP and IMP diaries.
- Assessment of compliance and tolerance:
  - Recording of changes in concomitant medications
  - Study drug dose adjustment and accountability
- Assessment of Adverse Events

The remote visit is subject to local blood tests and the ECG being performed by the local physician (family doctor). In this case the biomarker blood collection will not be performed.

Final Assessments (W16; ±14 days)

The following evaluations will be performed at the final assessment

- Clinical Assessments
  - Symptoms Scores (Questionnaires: Modified Canadian Chest pain scale, NYHA, MRC Dyspnoea scale, Long COVID Questionnaire);
  - QoL Questionnaire (RAND 36-Item Health Survey Version 2.0)
  - Clinical Assessments: vital signs, including heart rate (HR), blood pressure, height, and weight.
- Laboratory assessments (approximately 50 ml venous blood)
  - Local laboratory assessments (1 small EDTA; 1 serum, 1 coagulation): Full blood count, blood biochemistry (Na, K, creatinine/eGFR (MDRD), CRP, troponin, NTproBNP, D-dimer, Fibrinogen; Lipid profile, HbA1c, TSH
  - Biobanking samples ((2 big EDTA 8 ml for plasma extraction, 2 small EDTA 4 ml for whole blood-freezing, 2 big Serum probes) will be retained and stored for measurement for future measurements (OLINK proteomics analyses: Target 48/96 Cytokines; genomics, metabolomics)
  - pregnancy test in all females
- 12 lead ECG (intervals (ms): rhythm, axis, PQ; QRS, QT

- Cardiac magnetic resonance imaging (Cardiac volumes, LVEF, RVEF, LV mass, LV strain myocardial mapping, myocardial late gadolinium enhancement, aortic wall imaging (LGE), central aortic pulse wave velocity)
- CPET (if locally available): achieved Work Rate, VO_2max, VCO_2 max, RER, AT, slope
- Transfer of the participant's BP and IMP diaries.
- Assessment of compliance and tolerance:
  - Recording of changes in concomitant medications
  - Study drug dose adjustment and accountability
- Assessment of AEs

A shortened W16 visit focused on the primary endpoint (native CMR scan only) can be considered for participants that continue to experience strong fatigue symptoms at discretion of the local investigator.

Unscheduled visits:

Besides the scheduled visits described above, unscheduled visits may be arranged at any time for additional monitoring or questions at the discretion of the investigator. Any assessments or tests performed during these additional visits will be captured in Note to File Pages in the eCRF.

The overview of study procedures is provided in Table 1.

Table 1

	Activities/Examinations	Baseline	Study Period (Treatment)					Follow Up
	Weeks	**BL**	**W0§**	W2§	**W6**	W12§	**W16**	FU-Y1#
	Visit window	-6W-0D		+/- 10D			+/- 14D	+/- 14D
	Visits	0	**1**	2	**3**	4	**6**	6
Clinical assessment	Informed Consent (written)	**X**						
	Inclusion/Exclusion criteria	X						
	Demographic data and medical history	X						
	Concomitant Medication	X		X	**X**	X	**X**	
	Symptoms Scores Questionnaires	X		X	**X**	X	**X**	
	QoL Questionnaires	X			**X**		**X**	
	Vital signs	X	X***	X***	**X**	X***	**X**	
	Outcome Endpoints							X
Laboratory	*FBC, blood chemistry, liver function tests (only at BL), CRP, troponin, NTproBNP, D-dimer, Fibrinogen	**X**			**X**		**X**	
	Lipid profile, HbA1c, thyroid function tests	**X**					**X**	
	Blood samples stored (-80°C)	**X**			**X**		**X**	
	**Pregnancy test*	**X**					**X**	
ECG	12 lead ECG ((intervals (ms): PQ; QRS, QT)	**X**			**X**		**X**	
CMR	Myocardial mapping	**X**					**X**	
	Strain & Ejection fraction	**X**					**X**	
	LV volume and mass	**X**					**X**	
	Aortic wall imaging (LGE) and stiffness (PWV)	**X**					**X**	

DocuSign Envelope ID: 4F4388D5-A568-4FAC-82D1-39CB3AB82D1B

	Activities/Examinations	Baseline	Study Period (Treatment)					Follow Up
	Weeks	**BL**	**W0§**	W2§	**W6**	W12§	**W16**	FU-Y1#
	Visit window	-6W-0D		+/- 10D			+/- 14D	+/- 14D
	Visits	0	**1**	2	**3**	4	**6**	6
	Myocardial LGE	**X**					**X**	
CPET	achieved Work Rate, VO_2max, VCO_2 max, RER, AT, slope	**X**					**X**	
Participants´ diaries	***Home-based BP and HR measurements		X***	→	→	→	→	
	IMP diary		**X**	X	**X**	X	**X**	
	Provision of IMP to randomized participants	**X**						
	Compliance			X	**X**	X	**X**	
	Adverse Events	→	→	→	→	→	→	

*The processing of laboratory analyses will go ahead for participants fulfilling CMR criteria only

**all female participants at baseline and W16. In addition, women of childbearing potential (WOCBP) will be instructed to contact their study physician immediately in the absence of menstruation or in case of other clinical evidence of pregnancy for further clarification. Please also refer to Section 9.4.4 Pregnancy.

*** home-based measurement of blood pressure/heart rate

§will be performed as a telephone call or video evaluation (may be performed as remote visit at W6 in case of long-distance travelling).

#Follow-ups 1Y and 5Y: Proportion of participant with HF or MACE after 1 years; 1- year Event-free survival. Please refer to Section 9.2.11 (Outcome Endpoints) for details on collection and analysis of 1-Year Year outcomes.

DocuSign Envelope ID: 4F4388D5-A568-4FAC-82D1-39CB3AB82D1B

9.2 Clinical Procedures and Evaluations

All data will be documented in eCRF.

9.2.1 Informed Consent

Participants, registered via online portal or phoneline, and meeting eligibility criteria, will be contacted and informed about the study, including undergoing baseline, interim and final procedures, possible benefits, and risks. They will be explained that should they fulfil the CMR criteria for inflammatory cardiac involvement, they will undergo randomisation and receive the treatment. They will have the opportunity to discuss with the local investigator. Participants will be provided with written study material and given time (a minimum 24h) to reach the decision. Should they decide to proceed, a baseline visit will be arranged. Participants will provide written informed consent by signing an informed consent form (ICF).

9.2.2 Demographics

Clinical meta-data and demographics will be obtained for all participants, including age [years], sex [male/female/diverse], height [cm], weight [kg], BMI [kg/m²], ethnicity.

9.2.3 Medical history:

Medical history will be obtained at baseline, including symptoms, any relevant prior clinical history or previous medication will be collected.

9.2.4 Vital signs and BP Diary

Vital signs will be obtained and recorded at on-site and during the remote study visits using home-based BP devices. The measurements will include systolic and diastolic blood pressure [mmHg], heart rate [HR, bpm]. Participants will be provided with a CE-marked home blood pressure and HR monitoring device (Omron X2 Smart ®, CE mark provided in appendix 18.2.11). They will be instructed how to obtain and record the measurements, to be provided to the local investigator at the follow-ups. A template BP diary will be provided (Appendix 18.2.8). They will be asked to measure BP twice a day during the up-titration period (6 weeks), followed by once a day three times weekly.

Recordings from participants' own HR recording devices may be used in addition, to expand the record of HR at rest, exercise, and night-time. An SOP for home blood pressure monitoring will be provided.

9.2.5 Symptoms scores and Quality of Life

Symptoms scores and QoL will be assessed at baseline, W6 and W16 using standardised questionnaires provided in Appendix (18.2.3 – 18.2.7).

Symptoms Scores:

Symptoms Score will consist of standardised questionnaires, as previously published(2)(Appendix 18.2.3). This will be extended for cardiac-specific question using the Modified Canadian Chest Pain Scale(111), MRC Dyspnoea Scale(112) ((Appendix (110)18.2.5 and 18.2.6).

Quality of Life Assessments (QoL): All participants will complete RAND 36-item Health Survey Scores, Version 2 (Appendix 18.2.7)

9.2.6 Laboratory tests:

Venous blood sampling of approximately 50 ml will be performed prior to CMR using the venous access at baseline and W16.

Local laboratory assessments will go ahead in participants fulfilling CMR criteria only:

Full blood count (FBCs, haemoglobin, haematocrit, red blood cell count, MCV, MCH, MCHC, RBC morphology, white blood cell count (neutrophils and lymphocyte absolute) and platelets will be measured at the BL, and W16.

Coagulation screen: D-dimer, fibrinogen.

Blood biochemistry: (Na, K, creatinine/eGFR (MDRD), liver function tests (bilirubin, AST/ALT/GGT/Alkaline Phosphatase), CRP, troponin, NTproBNP, D-dimer, Fibrinogen; Cardiovascular risk factors and Rheumatological profile: TSH, Lipid profile (HDL, LDL, triglycerides, cholesterol, glucose, (apo)lipoproteins levels), and HbA1c will be measured at baseline only. Rheumatological screen will be conducted by testing for ANA, ENA, ANCA, C3, C4, and CK.

Biobanking samples will be collected at all on-site visits and stored at -80 degrees for future exploratory analyses using OLINK proteomics platforms: Target 48/96 Cytokines; genomics, metabolomics) (2 big EDTA 8 ml for plasma extraction, 2 small EDTA 4 ml for full blood freezing, 2 big Serum probes). As the studied indication is subject of intensive research and the publication of research findings is very dynamic in this field, the exact analyses cannot be specified at the time of initial protocol finalisation. Unused blood samples will be destroyed 25 years after End of Study.

An SOP for blood sampling will be provided. The collection of biomarker samples will be optional for W6 Visit.

9.2.7 ECG:

A standard 12-lead Electrocardiogram (ECG) will be performed at the baseline and W16 of the study. The following parameters will be collected: rhythm, axis, intervals (ms): PR, QRS, QT. An SOP for ECG will be provided.

9.2.8 CMR:

Participants will undergo cardiac magnetic resonance imaging (CMR) at baseline and W16 using standardised imaging protocol. Clinical scanners will be used at all sites. Participants will receive i.v. administration of gadolinium-based contrast agent (GBCA, gadobutrol, Gadovist® 0.1 mmol/kg, Bayer AG, Germany). Administration of Ivabradine may be considered prior to CMR in participants with resting heart rates ≥75/min, either orally, in tablets of 2.5 mg-15 mg(113), or intravenously, up to 10 mg(114). As chest pain is a prominent symptom of PostCOVID cardiac involvement, myocardial perfusion with Regadenosone (400 mcg/5 ml) is offered as an optional test.

Assessments will include cardiac volumes (ml; LVEDV, LVESV, RVEDV, RVESV), function (%; LVEF, RVEF), left ventricular strain (%; LV strain), LV mass (g), atrial size (LA area, cm2), native T1 and T2 mapping (ms), myocardial late gadolinium enhancement (presence, type, extent), aortic wall imaging (LGE, thickness(mm), and central aortic pulse wave velocity (m/s).

A standardised imaging protocol (IET-CVI Examcard) will be deployed at all sites (Appendix 18.2.9) and used specifically for the purpose of this study. Sites will be provided with an imaging manual and inducted into scanning procedures. Quality control will be conducted using remote supervision of scanning procedures.

All images will be analysed centrally at the IET-CVI Central Core Lab (Goethe University, Frankfurt), including the eligibility assessment. All imaging recordings will be sent securely via encrypted link to the IET-CVI Core Laboratory under pseudonym. All CMR postprocessing will be performed using automated and standardised postprocessing procedures with minimal observer input. All observers will be blinded to the underlying group allocation. Data analysis will be performed by dedicated core-lab study personnel for the whole duration of the study. An SOP for CMR imaging will be provided.

9.2.9 CPET:

Cardiopulmonary exercise test (CPET) will be performed at the baseline and W16, subject to local availability. The following parameters will be collected: achieved Work Rate, VO_2max, VCO_2 max, RER, AT, slope. An SOP for CPET will be provided.

9.2.10 Participant Treatment Diary:

Participants' adherence to therapy will be assessed by examining the treatment diary. A template of participant diary will be provided to the participants (Appendix 18.2.8). In this diary, intake of therapy will be documented daily. The diary will be provided to the local investigator at the follow-ups (W2, W6, W12, W16, and interim visits).

9.2.11 Outcome Endpoints:

For 1-Year Year follow up, outcome endpoints, as secondary study endpoints, will be collected. Therefore, the participant as well as the participant´s family doctor will be contacted by the site personnel to retrieve information on outpatient visits, hospitalizations, and medical procedures within 1- years' time. If available, the participant and the participant´s family doctor will be asked to provide copies of relevant documents (e.g. physician letters) which are to be filed in the medical record at the study site as source documents. Site stuff will abstract relevant data and document these data in the eCRF.

Based on the data documented in the eCRF, relevant events will be adjudicated by DSMC members, in line with the standardised predefined endpoint definitions(115). Two major outcome endpoints include a major adverse cardiovascular event (MACE), and HF Endpoint. MACE is a composite of adverse cardiovascular events (cardiovascular mortality, nonfatal acute coronary syndrome (ACS), an appropriate device discharge). ACS was defined by a significant rise of hs-TropT in the presence of typical symptoms(116). HF Endpoint is a composite of death due to HF or a documented episode of hospitalization, defined by an episode of hospitalization with symptoms and signs of HF, which was accompanied by a significant NT-proBNP rise(16). An appropriate device discharge was defined as a documented shock delivered through an implanted cardioverter device that terminated a life-threatening ventricular arrythmia, i.e. ventricular tachycardia or fibrillation. The first single event per participant will be included in the analysis.

DocuSign Envelope ID: 4F4388D5-A568-4FAC-82D1-39CB3AB82D1B

9.3 Management of Adverse Events

9.3.1 Adverse Events Reporting

An adverse event (AE) is defined as any untoward medical occurrence or worsening of a pre-existing medical condition in a participant or clinical investigation subject administered an investigational (medicinal) product and that does not necessarily have a causal relationship with this treatment. An AE can therefore be any unfavourable and unintended sign (including an abnormal laboratory finding, for example), symptom, or disease temporally associated with the use of a medicinal product, whether or not considered related to the medicinal product.
Adverse events can be spontaneously reported or elicited during open-ended questioning, examination, or evaluation of a participant. (In order to prevent reporting bias, participants should not be questioned regarding the specific occurrence of one or more AEs.) Pre-existing conditions, which worsen during a study, are to be reported as Adverse Events.

Any AE that results in any of the following outcomes will be considered a Serious Adverse Event (SAE):
1. Death
2. Life-threatening situation (participant was at risk of death at the time of the event. This does not refer to an event that might have caused death if it was of greater intensity.)
3. New in-patient hospitalization or prolongation of existing index hospitalization
4. Persistent or significant disability or incapacity
5. Congenital anomaly or birth defect
6. Important medical events that may not result in death, be life-threatening, or require hospitalization but may jeopardize the participant and may require medical or surgical intervention to prevent one of the above outcomes (based upon appropriate medical judgment), e.g.,

Intensity of adverse events will be graded on a three-point scale (mild, moderate, severe) and reported in detail as indicated in the CRF (see W.H.O. Handbook for Reporting Results of Cancer Treatment).

Mild:	Discomfort noticed but no disruption of normal daily activity.
Moderate:	Discomfort sufficient to reduce or affect daily activity.
Severe:	Inability to work or perform normal daily activity

By contrast, the term "serious" is used to describe an event based on an event outcome or actions usually associated with events that pose a threat to a participant's life or functioning. Seriousness (not severity) serves as a guide for defining regulatory reporting obligations.

For all collected AEs, the clinician who examines and evaluates the participant will determine the event's causality to the study drugs or contrast agent based on temporal relationship and their clinical judgment. The relationship will be given for both non-serious and serious AEs. The degree of certainty about causality will be graded using the categories below:

- Definitely Related: There is clear evidence to suggest a causal relationship, and other possible contributing factors can be ruled out.
- Probably Related: There is evidence to suggest a causal relationship, and the influence of other factors is unlikely.
- Possibly Related: There is some evidence to suggest a causal relationship. However, the influence of other factors may have contributed to the event.
- Unlikely: A clinical event, including an abnormal laboratory test result, whose temporal relationship to drug administration makes a causal relationship improbable and in which other drugs or chemicals or underlying disease provides plausible explanations.
- Not Related: The AE is completely independent of study drug administration, or contrast agent, and/or evidence exists that the event is definitely related to another aetiology.

Pre-existing conditions should be recorded upon participant enrolment (including start date of the condition, and severity - mild, moderate, severe). After the participant signs the informed consent form, any worsening of these conditions would be recorded.
Any new conditions would be recorded including date of onset, date of resolution, severity (mild, moderate, severe, or serious as defined above) and possible relationship to study drug, or contrast

DocuSign Envelope ID: 4F4388D5-A568-4FAC-82D1-39CB3AB82D1B

agent. As part of the source notes, follow up clinical assessments, laboratory tests, ECGs and diagnostic imaging related to adverse event should be documented.

Adverse events, especially those for which the relationship to IMP is considered “related” by the investigator, should be followed until resolved or until FU visit. If a clear explanation is established, it should be recorded on the CRF.

9.3.2 Other adverse events

The following AEs should be recorded and reported to the Sponsor or Project Management within 24h of knowledge, via eCRF, as described above.

9.3.2.1 AEs of special interest:

Any AE/SAE which is considered related (according to investigator´s assessment) to the contrast agent and which persists for 4 weeks or more post its administration.

9.3.2.2 AEs of special situations (for study medication and contrast agent):

1. reports of misuse, abuse, overdose, medication error and other use outside what is foreseen in the protocol,
2. drug dependency, withdrawal syndrome,
3. occupational exposure,
4. suspected transmission of an infectious agent,
5. drug interactions

9.4 Handling of Safety Parameters

All subjects will be monitored for AEs during the study. Assessments may include monitoring of any or all of the following parameters: the subject’s clinical symptoms, laboratory, pathological, radiological or surgical findings, physical examination findings, vital signs or findings from other appropriate tests and procedures. All adverse events related to the study therapy, serious adverse events and pregnancies that occur within the AE reporting deadline will be reported to the sponsor. All clinical adverse events (AEs) encountered during the clinical study will be recorded in medical records and on the AE page of the CRF. All AEs and SAEs will be recorded by the Investigator from the time of written informed consent till 4 weeks after the last administration of IMP (W16) or till 4 weeks after last administration of gadolinium-based contrast agent, whichever occurs last. This being considered the AE reporting deadline.

9.4.1 Serious Adverse Events (Immediately Reportable to the Sponsor or Project Management)

Any clinical adverse event or abnormal laboratory test value that is serious, irrespective of the treatment received by the participant, must be reported to the sponsor within 24h of knowledge (expedited reporting). Reporting takes place regularly via the eCRF, therefore the contact details below should only be used in emergencies / in the event of technical problems.

Drug Safety Manager:
Pharmacovigilance Alcedis
Email: [redacted]
Phone: [redacted]
Address: Alcedis GmbH; Winchesterstr. 3; 35394 Gießen

Safety reporting is carried out in accordance with local legislation and the applicable guidelines.

9.4.2 Treatment and Follow-up of Adverse Events

Adverse events, especially those for which the relationship to test "drug" is “related", should be followed up until resolved or stable, when considered related. SAEs should be followed until resolved or stable when considered related. If a clear explanation is established, it should be recorded on the CRF. Treatment of AEs is at the discretion of the investigator and should follow the standards of medical care at the investigator’s institution.

9.4.3 Follow-up of Abnormal Laboratory Test Values

Clinically significant laboratory test value abnormalities will be reported on the AE page of the CRF. In the event of a new unexplained abnormal laboratory test values (according to the local reference ranges) at follow up visits, or any interim tests, should be repeated to clarify, if the abnormality is likely to be related to the study procedures. If a clear explanation is established, it should be recorded on the CRF. Adjustment of treatment may be considered by the local investigator. Participants with unrelated abnormalities will be managed according to the local clinical practice.

9.4.4 Pregnancy

All women will undergo pregnancy test at baseline and W16. The choice of test, urine or blood, will be determined by the sites, taking the accuracy and speed of processing in local laboratory in account. In addition, WOCP will be instructed to contact their study physician immediately in the absence of menstruation or in case of other clinical evidence of pregnancy for further clarification by pregnancy testing. If, following initiation of the investigational product, it is subsequently discovered that a study participant is pregnant or may have been pregnant at the time of investigational product exposure, including during at least 6 half-lives after product administration, the investigational product will be permanently discontinued. Any pregnancy withing the treatment period will be treated as AE and reported to Sponsor within 24 hours of the site's awareness of the event. A special AE-Pregnancy form will be completed. Protocol-required procedures for study discontinuation and follow-up must be performed on the participant unless contraindicated by pregnancy. Other appropriate pregnancy follow-up procedures should be considered if indicated. The sponsor of the study will initiate a follow-up of the participant upon conclusion of the pregnancy.

9.5 Discontinuation of the Treatment

Worsening of cardiovascular symptoms or severe side effects (please see safety measures) may lead to discontinuation of the study drug. Participants who discontinue therapy before W16 will continue to perform all further planned study visits. Data up until the treatment change will be used for Per-Protocol analysis, whereas the complete dataset will be used in the Intention to Treat (ITT) analysis.

9.6 Premature Withdrawal or Termination of the Study

9.6.1 Premature Withdrawal of the Participant

Participants may voluntarily withdraw their consent to study participation at any time without giving any reason. The investigator may also, at their discretion, withdraw the participant from participating in this study at any time, or the sponsor may discontinue the study.

Reasons for early withdrawal from the study should be documented in the eCRF as:

- Study closed / terminated
- Participant died or is lost to follow-up
- Investigator's decision
- Participant withdrew consent to trial participation

For participants who withdraw consent to trial participation, participation ends immediately. Date of withdrawal from the study, with reason for withdrawal (if applicable), will be documented in the participant's medical record and recorded on the eCRF. In the case of death, a death certificate should be obtained, if possible, with the cause of death evaluated and documented.

9.6.2 Criteria for Termination of the Study

Both the sponsor and the investigator reserve the right to terminate the study at any time. Should this be necessary, both parties will arrange the procedures on an individual study basis after review and consultation. In terminating the study, the sponsor and the investigator will assure that adequate consideration is given to the protection of the participant's interests.

Following criteria could lead to a discontinuation or early termination of the study:

- Participants' safety
- Negative benefit / risk assessment due to new information
- Recruitment or procedural issues.

In case of premature termination of the study, all collected data will be analysed and a report has to be written. The sponsor must inform the competent authority, federal regulatory authority, the ethics committees and other authorities of member states of the European Union where the study is conducted within 15 days, giving detailed reason for the premature termination.

DocuSign Envelope ID: 4F4388D5-A568-4FAC-82D1-39CB3AB82D1B

9.6.3 Plan for Treatment after the End of Study Treatment

The investigator will inform the participants about all available standard-of-care treatments for after the completion of the study and the decision will be left to the participant and investigator's discretion.

10 Criteria for Evaluation of Study results

10.1 Criteria for evaluation of Efficacy

10.1.1 Primary Efficacy Endpoint

Absolute LVEF change to baseline at W16, measured by CMR, compared between the verum and placebo group by absolute treatment difference.

10.1.2 Secondary efficacy endpoints

The secondary efficacy parameters are listed in Section 4.1.4. The results of the findings for secondary endpoints are supportive and not confirmatory on their own. Therefore, the effect of the randomly allocated study treatment on the secondary endpoints are reported, in addition to measures of effect sizes, standard errors, confidence intervals, with nominal p values, unadjusted for testing multiplicity.

10.2 Criteria for Evaluation of Safety

The safety parameters of interest are listed in Section 4.2.2.

10.3 Description of Population and Participant Groups for Analyses

Participants with long COVID19 with evidence of cardiac involvement by CMR criteria and no known previous cardiovascular disease, who fulfil the inclusion and not fulfil the exclusion criteria. Participants will be randomised into verum and placebo groups.

10.3.1 Full Analysis Set (FAS)

The full analysis set (FAS) is defined to include all participants who were randomized into the study.

10.3.2 Intention to treat set (mITT)

The primary analysis will be performed on the ITT population. All participants who were randomized will be included in the ITT analysis. The intention to treat set is defined as the subgroup of participants of the FAS who had two assessments with CMR (BL and follow-up) and who received at least 4 weeks of treatment according to their treatment arm after study inclusion as eligible for data analysis. This set will be basis for the primary analysis. Participants excluded from statistical analysis will be listed by reason in concordance with ICH E3.

10.3.3 Per Protocol Population

Per protocol population (PP) is defined to include all participants who have completed BL examinations and another CMR at 16 weeks and have complied 75% with the study drug regimen will be included in the per-protocol analysis if the data confirms its feasibility and validity. Specific reasons for warranting exclusion will be documented prior to the closing of the database. Not all protocol deviators and violators will be excluded from the per protocol population.

DocuSign Envelope ID: 4F4388D5-A568-4FAC-82D1-39CB3AB82D1B

11 Statistical Methods

11.1 Sample Size Estimation

We consider a treatment difference in mean LVEF change of 2-4% with SD=9%, and a 1:1 treatment allocation, corresponding to an effect size of Cohen’s d=0.22 to 0.44, i.e. a small to moderate effect. Based on the calculations with G*Power 3.1.9.4, the unpaired t-test with a two-sided α=5%, a power of 80%, and an assumed Cohen´s effect size of 0.35 leads to 130 participants per study arm (260 participants for both arms). Assuming a drop-out rate of about 8% following randomization, a total of 280 participants will have to be randomized.

A SCR failure rate of 50% will be expected (presence of cardiovascular abnormalities in appr. 25% of participants and no detectable abnormalities in 25%). That means that approx. 560 to 600 participants will be expected to be screened.

Justification of primary endpoint:

The aim of this study is to allow the comparison of changes in LVEF, measured by CMR, between the study arms LVEF is a standard measure of cardiac performance in clinical trials. It can also be translated into other imaging modalities (echocardiography, cardiac CT). Based on the substantial validation and standardisation evidence supporting the accuracy of volumetric analyses (summarised in(107)), CMR is the gold-standard technique for measurement of LVEF. The chosen primary endpoint based on CMR is an objective endpoint and will be derived using highly standardised procedures. The automatization and AI-supported acquisition and postprocessing permit only observers' interference, yielding a reliable and reproducible objective parameter. The superior inter-study reproducibility of CMR based measurements compared with echocardiography allows for considerably lower calculated sample sizes (reductions of 55% to 93%) to show clinically relevant changes in LV dimensions and function. This is irrespective of the hearts structure (normal, dilated or hypertrophied)(117).

Literature Review for primary objective and endpoint:

A LVEF rate of 52-72% is defined as normal; the smaller LVEF, the worse the participant’s status(118). In 2020, Rodríguez-Santamarta et al reported ECG findings of 37 consecutive participants admitted to the intensive care unit (ICU) with ARDS secondary to COVID-19 in a single centre(119). Participants were divided into two groups: < 50% LVEF and ≥ 50% LVEF. Mean LVEF of all participants was 55.9 +/- SD 8.9 %, 40.8 +/- SD 3.8 % in the LVEF < 50% group and 58.9 +/- 6.2 % in the LVEF ≥ 50% LVEF group. Participants within the group with low LVEF had also higher high-sensitivity(8) troponin T (ng/ml) levels (170 ng/ml difference) (30). Puntmann et al also observed a mean LVEF of 57% +/- SD 6% in COVID-19 participants (n=100) compared to 60% +/- 5% in healthy controls (n=50), with significant differences in troponin (hsTnT) and hsCRP (4). Similar findings were observed in other centres.

In 2000, the effects of Losartan showed a beneficial effect compared to captopril for participants with heart failure and preserved EF NYHA≤II (ELITE, n=722, baseline mean LVEF 51% for all groups). Losartan was better tolerated and the losartan group had a significantly lower mortality (46% RR) and hospitalization rate (32%RR).(120) Losartan was generally well tolerated and comparable to enalapril in terms of exercise tolerance and reducing dyspnoea and fatigue in short-term (12-week) study of participants (n=116) (121)with heart failure. In subgroup with Losartan 50mg improved ejection fraction from 25.4±10.7 % to 27.7±11.4 (n=40, MD 2.3±44 %, p=0.02, measured by echocardiography).

Recalculating the SD for the LVEF change, a standard deviation of 9% is also valid for LVEF and LVEF change. It has been shown that immunosuppressive therapy with methylprednisolone can be effective in suppression of life-threatening ventricular tachyarrhythmias in chronic myocarditis(122), while the large Myocarditis Treatment Trial was unable to show a significant difference in LVEF after 28 weeks of treatment with prednisone compared to placebo in 1995(9). On average, the participants of the Myocarditis Treatment Trial improved their LVEF measurement by 11% points in LVEF in 28 weeks, i.e. 46% (35/24, 24% to 35%); all included participants had LVEF <45% at inclusion. It is notable that the referenced studies have either not studied COVID-19 participants but participants with hypertension or signs of myocarditis or lack information on the development of LVEF over time for COVID-19 participants. We deduce that a standard deviation of 9% is a valid assumption for the change in LVEF and that a small effect may only be expected since all participants will have a basic treatment which does have a large effect even in the placebo group following the Myocarditis Treatment trial. Thus, we will assume a 2-4% treatment difference in LVEF change at W16 to baseline. Drop-outs may occur due to withdrawal of informed consent, or loss of follow-up; given the size of the study, we expect this not to exceed 8%.(123)

DocuSign Envelope ID: 4F4388D5-A568-4FAC-82D1-39CB3AB82D1B

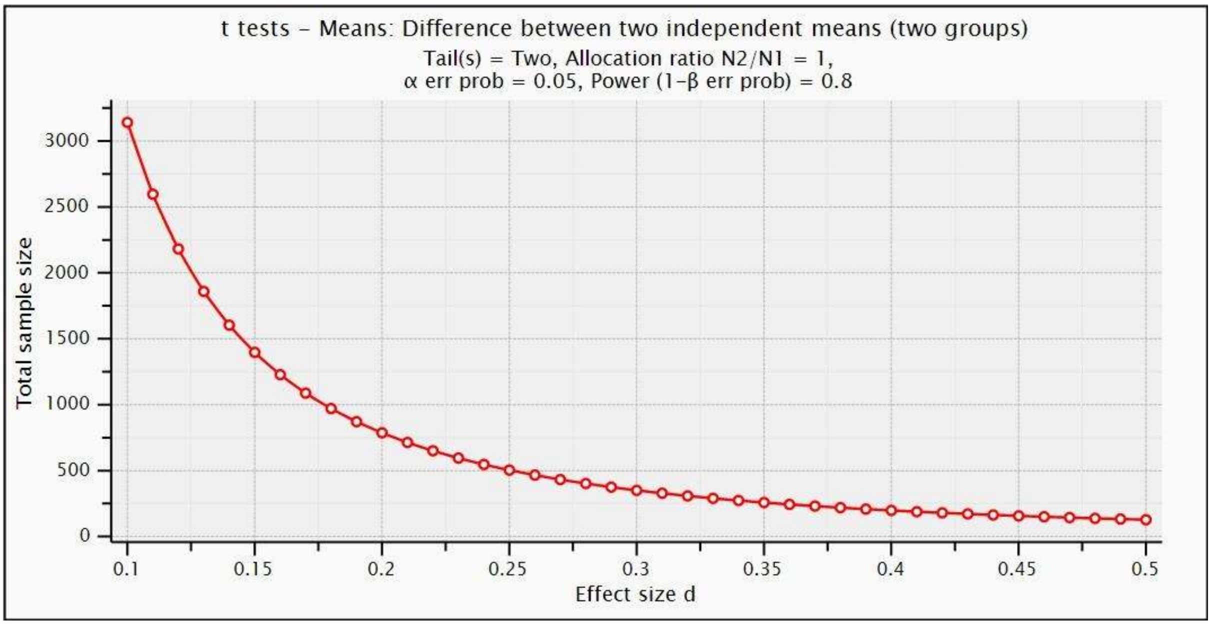

11.2 Bias and prevention methods

A priori concealment of randomisation allocation of participants will be applied to minimize potential for (un-)conscious selection bias. Since this is a double-blind placebo-controlled trial, blinding is performed and reduced the risk of assessment bias, since the treating physician is not aware of the treatment. Furthermore, the chosen primary endpoint based on CMR is an objective endpoint, which will be derived using standardised automated and AI-supported acquisition and postprocessing procedures, permitting only minimal observer interference, by this providing robust and reproducible measurements. The core-lab readers involved in any visual or manual evaluation of endpoint (e.g. PWV, LGE), are highly trained in SOPs, blind to the underlying clinical information to reduce likelihood of subjective assessments. Considering the objective primary endpoint parameter (LVEF by CMR), we expect the bias to be neglectable and results should be robust and well interpretable. To confirm this, results will be discussed in the framework of other published studies with similar objectives and endpoints. Owing to double blinding, the bias of participant reported outcomes (PRO) may be reduced since the participant is blinded for his/her treatment.

11.3 Summary statistics and graphical presentation

All summary statistics will be presented for the corresponding analysis set and treatment groups and stratified by treatment and visit. Descriptive summary statistics will at least comprise the number of missing values, the absolute and relative number (N, %) of any ordinal or nominal parameter. Similarly, for metric parameters the number of missing values, the arithmetic mean, SD, median, Q1, Q3, minimum, maximum will be reported. Two-sided 95% confidence intervals of the expectation values of the population (estimated by the mean) and probabilities within the population (estimated by the observed frequencies) may be given for the treatment difference of the primary and secondary endpoints, if the data is distributed accordingly. If required, continuous parameters will be tested for normal distribution (Kolmogorov-Smirnov-Test/Shapiro-Wilk-Test/Residual plots/Q-Q-plots) or other, if predefined or straightforward. If necessary, adequate data transformation, such as a Box-Cox transformation (e.g. logarithmic), will be performed to achieve normality. Beside the primary endpoint, some parameters will be illustrated using adequate graphical illustrations such as boxplots, mean plots, bar charts or flow charts. If the number of observations becomes too small (< 10), either dot plots will be considered for continuous parameter or graphical illustration will be skipped. For dichotomous endpoints shift-tables or contingency tables may be used.

Unless stated otherwise, time-to-event data will in general be reported using the Kaplan-Meier (KM; Product Limit) estimators (including 95% confidence intervals) or life tables (discrete timeline) and illustrated with the corresponding curve including the number of participants at risk. Participants who drop-out or who are excluded for safety reasons will in general be censored in efficacy analyses unless specified otherwise or implausible. Cumulative occurrence rates are reported

similarly, and will be plotted, where occurrence rates are requested. If less than 5 values per variable are available, the data may be enumerated instead of being summarized.
Model fit of all statistical models will be determined. Besides the analysis of residuals, the (adjusted) coefficient of determination R^2 and the likelihood ratio test may be used to evaluate the general model fit of linear models. In case of logistic models, a Hosmer-Lemeshow test may be applied. Other statistical models will be handled accordingly.
Only deviations from the general overview will be noted in the subsequent sub-sections of the efficacy analysis for the corresponding parameter. Further details will be provided in the Statistical Analysis Plan (SAP).

11.4 Outcome analyses

All primary and secondary efficacy analysis will be performed for the mITT and for the PP population to account for protocol violations unless stated otherwise. mITT analysis will apply the analysis strategy of the treatment policy, while PP analysis will apply a while on treatment strategy. All primary and secondary endpoints will also be analysed in a descriptive manner using summary statistics stratified by treatment and visit. The mean (or median), respectively proportion, of the treatment difference in selected endpoints will be reported at all visits of interest together with the 95% confidence interval (or IQR). All named analyses will be performed on participant level. Centre effects will be investigated as random effects or descriptively.

11.4.1 Primary Efficacy Analysis

For primary analysis, the absolute LVEF change to baseline at W16, measured by CMR, will be compared between the verum and placebo group by absolute treatment difference.
The unpaired t-test will be applied. The null hypothesis H0: $\mu VERUM = \mu PL$ vs. H1: $\mu VERUM \neq \mu PL$ (with μ representing the expectation value of the absolute change of LVEF from BL at W16) will be tested confirmatively at the 5% significance level.
Secondary endpoints will be compared exploratively between treatment arms or subgroups thereof at a 5% significance level in a 2-sided manner (hypotheses formulated analogously to the primary hypothesis). Further exploratory investigation may be employed.
In case of multiplicity the Bonferroni-Holm or Tukey adjustment will be applied to adjust p-values. Then adjusted and unadjusted p-values will be reported.

***Primary Endpoint Analysis*:**

Objective: To determine the efficacy of immunosuppressive and antiremodelling therapy in COVID-19-induced cardiac involvement determined by CMR to reduce inflammatory myocardial injury compared to placebo
Primary Endpoint: the absolute LVEF change to baseline at W16, measured by CMR, will be compared between the verum and placebo group by absolute treatment difference.
For primary analysis, we analyse the change in LVEF at W16 compared to baseline between the treatment groups.
Superiority of the verum group to improve LVEF compared to placebo will be tested as H0: $\mu VERUM = \mu PL$ vs. H1: $\mu VERUM \neq \mu PL$ (see section (11.1) at the 5% significance level in a two-sided manner by an unpaired t-test.

11.4.2 Secondary Efficacy Analysis:

For all secondary endpoints, descriptive statistics will be used to compare baseline characteristics between the two groups. Continuous variables will be expressed as mean ± SD, categorical variables will be expressed in counts with percentages.
The differences in CMR parameters and other continuous secondary outcomes from baseline to 16 weeks post randomization between the two treatment groups will be analysed using an ANCOVA. Treatment effect estimates and 95% confidence intervals will be determined. These hypotheses tests will be of explorative nature and tested at a global 5% significance level between treatment groups/factors in a 2-sided manner. Correlations between secondary endpoints may be assessed using contingency tables (relative risk, odds ratio), correlation coefficient (Pearson, Spearman, Kendall's Tau) or, depending on the nature of the correlation.

11.4.3 Safety Analyses

The safety analysis set is defined as all participants who received at least one dose of IMP treatment (verum or placebo) during the study. The difference in continuous outcomes will be analysed as described above in Section 11.3. For dichotomous safety outcomes, the difference in proportions experiencing these will be estimated, with 95% confidence intervals.

Evaluation of adverse events

DocuSign Envelope ID: 4F4388D5-A568-4FAC-82D1-39CB3AB82D1B

Safety parameters will be described as

- Frequency, type, severity and relatedness of adverse events

Endpoints of adverse events:

- Proportion of participants with infectious complications (a combination of at least two of the following: fever ≥38.5°C, rise on hsCRP, neutrophilia, lymphocytosis, need for antibiotic treatment)
- Proportion of participants with symptomatic hypotension (systolic BP<90 mmHg, accompanied by a blackout) or tachycardia (palpitations and heart rate >110/min, accompanied by a syncope)
- Proportion of participants with a significant rise in cardiac biomarkers (hsTNT, NTproBNP, >3-times the BL)
- Proportion of participants with a significant drop in eGFR compared to BL (>25%)
- Proportion of participants with worsening of cardiovascular symptoms (increase in NYHA class, clinical heart failure)
- Absolute changes in lipid profile, HbA1c, thyroid function tests compared to BL
- Proportion of participants with acute psychotic episode
- Proportion of participants with hypertensive crisis (systolic BP>180mmHg and diastolic >120 mmHg)

Safety data will be analysed describing frequency, severity and types of adverse events for all treatment groups. The proportion of AEs (including AESI and special situations) related to the contrast agent will also be analysed.
All adverse events will be coded and tabulated by system organ class and preferred term for individual events within each system organ class and will be presented in descending frequency. Adverse events will also be tabulated by severity and relationship to the study medication. Serious adverse events will be summarized separately. Listings will be produced in concordance with ICH E3, including actions taken and outcome.
A treatment comparison of the occurrence of frequent AEs (especially cardiac related) may be performed using a Chi Square Test (or exact Fisher Test in case of small abs. frequencies) with α=5%. Time-to-event analysis may be performed for frequent AEs of interest. Analysis methods may employ Kaplan-Maier curves, Log-rank tests, lifetable analysis, or Cox-regressions. A competing risk analysis will then also be considered.

<u>Analysis of HF events, MACE and death</u>

- Proportion of participant with HF or MACE after 1 years
- 1- year survival

The number of frequency of events of HF, MACE and death will be given up to 1 year FU. If at least approximately 15-20 events are observed per group, a competing risk analysis will be performed to investigate time to HF and MACE after 1 year. The time to first occurring event (HF, MACE, death) will be modelled. Participants who drop out prior or survive event-free over the timeframe will be censored. Death will be modelled as additional status. The time-to event will be compared between treatments and between the event of HF and MACE by hazard ratio. The same analysis will be performed for only HF and only MACE with death as competing risk.
Additionally, Kaplan-Meier survival analysis will be performed for the event of death and treatment comparison using a Log-rank test at α=5%. The odds ratio with 2-sided 95% confidence interval will be determined to compare the odds of dying (mortality rates) between treatments at 1-year FU. If only few events are observed, the absolute time to the corresponding events will be assessed for 1-year –FU and compared between the treatment groups using a Wilcoxon rank sum test.
<u>Compliance and tolerance</u>
Compliance will be assessed by the participant diary and drug accountability (e.g. empty Dossett boxes) for IMP intake. Summary statistics will be given for the treatment intake (IMP), i.e. consumed medication prescribed, stratified by treatment and visits. Relevant information of the participant diary will be summarized and reported descriptively or as documented.
Tolerance will be assessed by the absolute and relative (%) frequency of participants changing their treatment number of participants required dose reduction or treatment cessation due to side effects.

DocuSign Envelope ID: 4F4388D5-A568-4FAC-82D1-39CB3AB82D1B

Cumulative oral corticosteroid dose will be calculated and described by summary statistics at all visits and illustrated by mean +/- SD or boxplots over time stratified by treatment group.

11.4.4 Interim analysis

No interim analyses are planned in the study.

11.4.5 Other types of analyses

Baseline demographics, ECG, CPET, participant diary, physical examination, medical history, concomitant medication, will be described in terms of summary statistics or as documented, and compared by two-group comparisons of the averages or counts(frequencies), as appropriate for the type of the data. Biobanking will allow future exploratory blood analyses if budget permits and summarised as comparisons of absolute values as well as profiles of group signatures between the two groups.

11.4.6 Handling of missing data and values above/below the LOQ

In general, missing data of a parameter will not be transformed and remains untouched, unless > 5% of the data is missing or defined otherwise. Summary statistics will generally be given including the underlying number of valid individual values and missing data.

For the primary analysis, the unpaired t-test will be applied. If a large number of values are missing, LOCF will be considered for the LVEF to determine the primary endpoint. If data is imputed, primary analysis will be compared between the imputed data and the observed cases.

Values below/above the LOQ will in general be imputed by a suitable fixed value (e.g. LLOQ, ULOQ, Zero), depending on the nature of the laboratory value and its time of assessment. All handling of values above/below the LOQ and handling of missing values will be described in the Statistical Analysis Plan (SAP).

11.4.7 Handling of therapy changes and unscheduled visits

Unscheduled visits will not be included in the analysis. Participants who change therapy before W16 will continue to perform all further planned study visits. Data up until the treatment change will be used for Per-Protocol analysis (as a while on treatment strategy for the intercurrent event of a treatment change), whereas the complete dataset will be used in the Intention to Treat (ITT) analysis (Following a treatment policy strategy).

Protocol deviations, drop-outs and participants excluded from analysis All protocol deviations, drop-outs of the study and participants excluded from analysis will be described by summary statistics and presented in a CONSORT. Where possible, they will be categorized by reason. Drop-outs include withdrawals, screening failures and participants who are lost-to-follow-up.

11.5 Replacement Policy (Ensuring Adequate Numbers of Evaluable Subjects)

Up to a maximum of 20 participants (i.e., appr. 6%) will be replaced due to dropouts and missing values of primary endpoint. Reasons for dropouts have to be documented in the CRF.

11.5.1 For Centres

A centre may be replaced for the following administrative reasons: excessively slow recruitment or poor protocol adherence or any other suspected compliance problem with GCP.

11.6 Limitations

The study is a double blind RCT study, as such there are considerable measures to control the bias in place. Thus, the impact of the expected bias is judged as neglectable (see section 11.2). Owing to CMR-based inclusion criteria using standardised imaging method, we expect the results to be reliable and generalizable.

Recruitment might be a risk factor, if many participants do not show cardiac abnormalities in CMR, which is an inclusion criterion for the study. We have chosen the inclusion criteria to mitigate this risk. We have further calculated and planned for SCR failures. We have also chosen centres with a long-standing expertise in Cardiac Imaging, inflammatory cardiac conditions, and rheumatology e.g., Frankfurt am Main, Vienna, Kiel) and high flow of COVID-19 participants (Vienna, Greifswald) to minimize the risk of recruitment problems.

12 Organisational Structure

12.1 Sponsor

Goethe University Frankfurt,
represented by the President
Theodor-Adorno-Platz 6; D-60323 Frankfurt/Main

12.2 Contract Research Organisation (CRO)

Alcedis GmbH is the appointed CRO to be responsible for overall study management, including preparation of all study material and procedures, in-house and on-site data monitoring, data handling and safety reporting, data quality assurance, and statistical reporting. Alcedis GmbH will also be responsible for organization of meetings, site initiation and training, on-site monitoring and study close-out. Alcedis GmbH will work with other CROs but holds the overall responsibility for the trial management. Alcedis GmbH will issue regular progress reports and newsletters to be sent to investigators and committees.

12.3 Steering Committee

A Steering Committee will be appointed, which will include the Study Chair, independent experts external to the study, and a minimum of four investigators participating in the trial. The Steering Committee will be responsible for providing clinical and methodological guidance, including overall study design, execution, analysis, and publication of the main study results. The Steering Committee will oversee the management of the clinical trial sites and will also act as the Publication Committee.

While the study is ongoing, the Committee will approve any protocol amendment that may become necessary and is responsible for maintaining the scientific integrity of the study. Steering Committee meetings may include representatives from the Sponsor, the Core Laboratory directors, and from Alcedis GmbH.

12.4 Data Safety and Monitoring Committee (DSMC)

An independent DSMC will be established in line with EMEA/CHMP/EWP/5872/03 Corr recommendations. This will consist of a group of independent experts external to a study assessing the progress, safety data and, if needed critical efficacy endpoints of a clinical study. The DSMC will review safety data on the ongoing trial on a regular basis and when necessary, they will recommend to the sponsor whether to continue, modify or terminate the trial. They may review unblinded study information (on a participant level or treatment group level) during the conduct of the study only in case this is considered as needed to fulfil the DSMC tasks. The committee consists of two physicians and a statistician who are not otherwise associated with the study. A list of the DSMC members can be found in Appendix 18.2.12.

The DSMC will conduct routine reviews of accumulating trial data in order to make recommendations concerning safety concerns and the appropriateness of continuing the study, as documented in the DSMC Charter. Additionally, the DSMC will be provided with the documented data regarding 1-Year follow up to perform adjudication in line with the relevant standardised endpoint definition.

12.5 Core Laboratory for CMR

All CMR assessments will be performed independently from the local study team and interpreted according to previously established criteria in a CMR Core Laboratory (IET-CVI, Goethe University Frankfurt).

12.6 Funding

This is an investigator-initiated research study supported by Bayer AG.

12.7 Insurance

The sponsor applies for an appropriate and adequate insurance according to the local legislation. All participants enrolled in the study will be insured for study-related procedures as specified in this protocol.

DocuSign Envelope ID: 4F4388D5-A568-4FAC-82D1-39CB3AB82D1B

13 Data Collection and Monitoring

13.1 Case Report Forms (CRF)

Electronic data capture will be used for this trial, meaning that all study data will be entered in electronic case report forms (eCRF) at the investigational site. Data collection will be completed by authorized study site personnel designated by the Investigator. Appropriate training and security measures will be completed with the Investigator and all authorized study site personnel prior to the study being initiated and any data being entered into the system for any study participants.

The study data will be housed on a secure in-house server at the CRO throughout the duration of study, and up to 10 years after the study is complete. An encrypted compact disc of the tabulated study data will be stored at the CRO for 25 years after completion of the study

13.2 Data Management and Cleaning

Records for all participants from whom an Informed Consent is obtained will be stored on a secure eCRF that will be maintained at the Data Management Centre. All eCRF corrections are to be made by an investigator or other authorized study site personnel. The investigator/co-investigator must confirm by his/her electronic signature in a specific section of the eCRF that he/she has reviewed the data, and that the data is complete and accurate. Data validation procedures will be described in detail in the Data Management Plan.

13.3 Monitoring

13.3.1 Virtual and/or Onsite Monitoring

The CRO is responsible for monitoring according to applicable local Good Clinical Practice (GCP) standards and International Conference on Harmonization (ICH) guidelines to ensure the completeness, correctness, and consistency of the data and to assess whether the study is executed according to this protocol. Specific items to be checked are listed in the Investigator's Study File (risk-based monitoring).

To verify that the CRFs are completed accurately and in accordance with source documents, source data verification will be performed. The CRFs and related source documents will be reviewed in detail by the on-site monitor during each visit. Checks for completeness and correctness of the data will be done by comparing CRF entries with information in the participants' local medical records.

13.3.2 In-house Monitoring

In-house data review and cleaning will be performed by the CRO. In case of missing, erroneous or incomplete data, further information will be requested by the CRO via electronic Data Clarification Forms (eDCFs). These are sent directly to the investigator and copied to the on-site monitor.

13.4 Audit/Inspection

The Sponsor, the CRO and/or a competent authority may perform audits. The auditor/inspector must have access to all study and source documentation, facilities and equipment used in this study. The Steering Committee will supervise audit procedures and is entitled to initiate audits on its own.

14 Investigator Responsibilities And Obligations

14.1 Declaration of Helsinki

The study will be carried out in accordance with the provisions of the Declaration of Helsinki (last revised version, see Appendix 18.1) and with applicable local GCP standards.

14.2 Ethics Committee and Regulatory Authority Review

According to local laws and regulations, the study protocol, and the Participant ICF must be approved by a local EC/RA for each participating centre.

It is the responsibility of the investigator to submit the protocol for institutional review. A copy of the letter of approval from the local EC/RA, with a content in accordance with local regulations, must have been received by the CRO prior to shipment of study drugs to the investigational site. Major changes to the protocol, as well as a change of a principal investigator, must be approved by the local EC/RA and documentation of this approval must be provided. Records of the local EC/RA review and approval of all documents pertaining to this study must be kept on file by the investigator in the Investigator’s Study File.

Apart from the investigational procedures specified in the protocol, investigators are not allowed to perform ancillary studies without written approval from the Steering Committee and the local EC/RA.

14.3 Informed Consent and Participant Protection

14.3.1 Participant Informed Consent

It is an obligation of the investigator to obtain informed consent from the participant by means of a dated and signed ICF before any study-related procedure is performed. The ICF must be written in the local language in accordance with local laws and regulations. ‘Informed consent’ also implies individual discussion with the participant about the nature of study treatment and examinations to be conducted in a language that is easy to comprehend. The participant should fully understand that his/her refusal to participate in the study will not affect the quality of medical care. In addition, the participant must be informed that, without disclosing their name, relevant medical data will be disclosed to CRO(s), that his/her medical records will be inspected during on-site monitoring and may be inspected again by auditors and/or regulatory authorities.

Should a protocol amendment be made, the ICF may be revised to reflect the changes in the protocol. It is the responsibility of the investigator to ensure that an amended ICF is reviewed and approved by the local EC/RA, and that it is signed by all participants subsequently entered in the study and those currently in the study, if affected by the amendment.

The informed consent form personally signed and dated by the participant must be kept on file by the investigator(s) and documented in the case report form and the subject's medical records. The investigator confirms obtaining the written informed consent to the sponsor.

If new safety information results in significant changes in the risk / benefit assessment, the consent form should be reviewed and updated if necessary. All subjects excluding participants that have terminated the study should be informed of the new information and must give their written informed consent to continue the study. If the family doctors are informed of their participants’ participation in the clinical study, this should be mentioned in the consent form.

14.3.2 Participant Data Protection

The participants should be informed in writing that his/her medical data relevant to this study will be stored and analysed while maintaining confidentiality in accordance with local data protection laws. All data transferred to the CRF, and any process derived from the CRF will be handled anonymously. This will ensure that the identity of the individual will be protected. The data of participants that have prematurely left the study will be fully anonymised and no longer traceable to the person.

The participant should also be informed in writing about the possibility of audits by authorized representatives of the Sponsor, the CRO or a designee and/or regulatory agencies in which case a review of those parts of the hospital records relevant to the study may be required.

14.4 Study Protocol Adherence and Modifications

14.4.1 Protocol Adherence

The protocol must be read thoroughly, and the instructions must be followed exactly. The same applies to instructions given in the eCRF and to any additional instructions issued by the CRO. Whenever a deviation occurs in the interest of the participant’s wellbeing, the on-site monitor must be informed, and a course of action must be agreed upon. All deviations will be kept in the protocol deviation log.

14.4.2 Changes to Protocol and Related Procedures

Changes to the protocol should only be made in the form of protocol amendments. If substantial changes to the design of the study are made, local EC/RA should be notified and, if required, approve the change before inclusion of new participants.
The CRO is responsible for the distribution of a protocol amendment to investigators. Investigators are responsible for the distribution of an amendment to all staff involved in the study and to the local EC/BPharm.

14.5 Investigational Product Control

It is the investigator's responsibility to ensure that study drugs are stored in a secure area (locked, limited personnel access), and dispensed appropriately. The investigator is responsible for maintaining accurate records of the dispensing of the study medication in a study drug accountability log. All study drug supplies are for this protocol only and not for any other use. After completion of the study, all unused study materials must be destroyed on site or returned to pharmacy.

14.6 Data Collection and Documentation

All source data will be labelled with pseudonym. For every participant, the participant file must clearly indicate that the participant has given informed consent and participates in the study. For all study assessments, the file should include clinic visit and interim contact dates, records of vital signs, medical history, clinical assessment findings, procedures performed and their findings, laboratory results, concomitant treatment, any AEs encountered and other notes as appropriate. This constitutes 'source data'. All entries on the eCRFs must be backed up by source data unless specified otherwise. Source data must be made available for perusal by the on-site monitor during a monitoring visit. In order to allow detection of inaccuracies in transcribing data from original records into the eCRF, all original laboratory reports must be kept available for review in the participant file.
The CRFs must be kept in order and up-to-date so that they always reflect the latest observations on the participants enrolled in the study. Each participant's study file should have attached to it the original signed ICF. When the study is completed, the ICF should be kept on file with a copy of the completed eCRF in the study file provided, or a note should be made indicating where the study records can be located. All records should be kept in accordance with applicable national laws and regulations. The data of participants that have prematurely left the study will be fully anonymised and no longer traceable to the person.

14.7 Reporting of AEs and SAEs

It is a regulatory obligation of the investigator and her/his staff to record and report any serious clinical event or adverse experience that occurs while a participant is participating in this study. Detailed instructions for AE and SAE reporting are given in Sections 9.3.1 and 9.4.1 of this protocol. The instructions given in this Section must be observed closely. Noncompliance is a serious protocol violation and may lead to the closure of the centre involved. If required by local regulations, the investigator must also inform the local EC/BPharm about SAEs.

14.8 Records Retention

The investigator must maintain adequate and accurate records to enable the conduct of the study to be fully documented and the study data to be subsequently verified. These documents should be classified into two different separate categories (1) investigator's study file, and (2) participant source documents. The investigator's study file will contain the protocol/amendments, case report and query forms, Ethics Committee Review Board and RA approval (if required) with correspondence, sample informed consent, drug records, staff curriculum vitae and authorization forms and other appropriate documents/correspondence etc. The participant's source documents (usually defined by the project in advance to record key efficacy/safety parameters independent of the CRFs) would include participant visit records, physician's and nurse's notes, appointment book, original laboratory reports, ECG and special assessment reports, signed ICF(s), consultant letters, and participant screening and enrolment logs. The investigator must keep these two categories of documents on file after completion or discontinuation of the study according to local requirements. Should the investigator wish to assign the study records to another party or move them to another location, the CRO must be notified in advance.

14.9 Confidentiality of Trial Documents and Participant Records

The investigator must assure that participant anonymity will be maintained and that their identities shall be protected from unauthorized parties. On eCRFs or other documents submitted to CRO and/or to the Sponsor, participants should not be identified by their names, but by an identification

code. The investigator should keep a participant enrolment log relating codes to the names of participants. The investigator should maintain documents that are not for submission to the CRO and/or the Sponsor in strict confidence. The data of participants that have prematurely left the study will be fully anonymised and no longer traceable to the person.

14.10 Intellectual property and copyright Protection of clinical trial data

The data collected within this clinical trial is subject the GDPR article 15.4 on the grounds of **the context of scientific research in public interest**, for which purpose the data is obtained. It is paramount to safeguard the preservation of the authenticity of the study and scientific rigor of its conduct including blinding procedures, as well as intellectual property, which mandate a minimal exposure of any study material. Thus participants randomised into the study will not receive any study results, be shown images of the heart or provided with DVDs. The data of participants that have prematurely left the study will be fully anonymised and no longer traceable to the person. Participants with clinically significant findings in the baseline scan (LVEF <45%, structural heart disease, etc) or blood results indicating medical emergency, will not be eligible for the study. They will be provided with a report and instructed to see their doctor to arrange diagnostic procedures using routine clinical pathways.

14.11 Publication of Data

The sponsor will be responsible for preparation of a complete, integrated final clinical study report of the study. This report will be signed by the principal investigator of the study. The investigator / investigator(s) will each receive a copy of this report. Publication(s) will be prepared according to standard guidelines (e.g., Good Publication Practice and Recommendations for the Conduct, Reporting, Editing, and Publication of Scholarly Work in Medical Journals) [38].

14.12 Direct Access to Source Data/Documents

The investigator shall supply the CRO, the Sponsor and/or regulatory agencies on request with any required background data from the study documentation or clinic records. This is particularly important when errors in data entry are suspected. In case of special problems and/or governmental queries or requests for audit inspections, it is also necessary to have access to the complete study records, provided that participant confidentiality is protected. All source data will be labelled with pseudonym. The data of participants that have prematurely left the study will be fully anonymised and no longer traceable to the person.

14.13 Trial Network Registration

The study will be registered on http://clinicaltrials.gov prior to participant enrolment.

15 Study Duration and Global End of Study Definition

The study starts with the first participant consented (fully signed ICF). The global end of the study is defined as the last visit of the last participant randomized.

Eligible participants will be randomised and followed for 18 weeks (16 weeks treatment, 2 weeks AE reporting deadline).
We estimate: Recruitment for the study is estimated to take appr. 12 months. The study duration till the primary endpoint is estimated to take appr. 6 months, and cleaning 3 months, database lock, analysis and reporting 3 months while the whole study is expected to be completed in Q4/2028

The study end is defined as database hard lock after data cleaning process is completed.

Study start date:	Q4 / 2022
Till primary endpoint:	Q2/2025
Study end date:	Q2/ 2026

DocuSign Envelope ID: 4F4388D5-A568-4FAC-82D1-39CB3AB82D1B

16 References

1. Committee W., Gluckman TJ., Bhave NM., et al. 2022 ACC Expert Consensus Decision Pathway on Cardiovascular Sequelae of COVID-19 in Adults: Myocarditis and Other Myocardial Involvement, Post-Acute Sequelae of SARS-CoV-2 Infection, and Return to Play. J Am Coll Cardiol 2022;79(17):1717–56. Doi: 10.1016/j.jacc.2022.02.003.

2. Sudre CH., Murray B., Varsavsky T., et al. Attributes and predictors of long COVID. Nat Med 2021;27(4):626–31. Doi: 10.1038/s41591-021-01292-y.

3. Pretorius E., Vlok M., Venter C., et al. Persistent clotting protein pathology in Long COVID/Post-Acute Sequelae of COVID-19 (PASC) is accompanied by increased levels of antiplasmin. Cardiovasc Diabetol 2021;20(1):172. Doi: 10.1186/s12933-021-01359-7.

4. Puntmann VO., Zeiher AM., Nagel E. T1 and T2 mapping in myocarditis: seeing beyond the horizon of Lake Louise criteria and histopathology. Expert Rev Cardiovasc Ther 2018;16(5):319–30. Doi: 10.1080/14779072.2018.1455499.

5. Francone M., Chimenti C., Galea N., et al. CMR Sensitivity Varies With Clinical Presentation and Extent of Cell Necrosis in Biopsy-Proven Acute Myocarditis. JACC Cardiovasc Imaging 2014;7(3):254–63. Doi: 10.1016/j.jcmg.2013.10.011.

6. Starekova J., Bluemke DA., Bradham WS., et al. Evaluation for Myocarditis in Competitive Student Athletes Recovering From Coronavirus Disease 2019 With Cardiac Magnetic Resonance Imaging. Jama Cardiol 2021;6(8):945–50. Doi: 10.1001/jamacardio.2020.7444.

7. Daniels CJ., Rajpal S., Greenshields JT., et al. Prevalence of Clinical and Subclinical Myocarditis in Competitive Athletes With Recent SARS-CoV-2 Infection. Jama Cardiol 2021;6(9):1078–87. Doi: 10.1001/jamacardio.2021.2065.

8. Puntmann VO., Carerj ML., Wieters I., et al. Outcomes of Cardiovascular Magnetic Resonance Imaging in Patients Recently Recovered From Coronavirus Disease 2019 (COVID-19). JAMA Cardiol 2020;5(11):1265–73. Doi: 10.1001/jamacardio.2020.3557.

9. Mason JW., O'Connell JB., Herskowitz A., et al. A Clinical Trial of Immunosuppressive Therapy for Myocarditis. New Engl J Medicine 1995;333(5):269–75. Doi: 10.1056/nejm199508033330501.

10. Chen HS., Wang W., Wu S., Liu JP. Corticosteroids for viral myocarditis. Cochrane Db Syst Rev 2013;(10). Doi: 10.1002/14651858.cd004471.pub3.

11. Maisch B., Herzum M., Hufnagel G., Bethge C., Schönian U. Immunosuppressive treatment for myocarditis and dilated cardiomyopathy. Eur Heart J 1995;16 Suppl O:153–61.

12. Kindermann I., Barth C., Mahfoud F., et al. Update on Myocarditis. J Am Coll Cardiol 2012;59(9):779–92. Doi: 10.1016/j.jacc.2011.09.074.

13. Vescovo G., Libera LD., Serafini F., et al. Improved Exercise Tolerance After Losartan and Enalapril in Heart Failure. Circulation 1998;98(17):1742–9. Doi: 10.1161/01.cir.98.17.1742.

14. Warner JG., Metzger DC., Kitzman DW., Wesley DJ., Little WC. Losartan improves exercise tolerance in patients with diastolic dysfunction and a hypertensive response to exercise. J Am Coll Cardiol 1999;33(6):1567–72. Doi: 10.1016/s0735-1097(99)00048-0.

15. Little WC., Zile MR., Klein A., Appleton CP., Kitzman DW., Wesley-Farrington DJ. Effect of Losartan and Hydrochlorothiazide on Exercise Tolerance in Exertional Hypertension and Left Ventricular Diastolic Dysfunction. Am J Cardiol 2006;98(3):383–5. Doi: 10.1016/j.amjcard.2006.01.106.

16. McDonagh TA., Metra M., Adamo M., et al. 2021 ESC Guidelines for the diagnosis and treatment of acute and chronic heart failure. Eur Heart J 2021;42(36):3599–726. Doi: 10.1093/eurheartj/ehab368.

17. Mandal S., Barnett J., Brill SE., et al. "Long-COVID": a cross-sectional study of persisting symptoms, biomarker and imaging abnormalities following hospitalisation for COVID-19. Thorax 2020;76(4):396–8. Doi: 10.1136/thoraxjnl-2020-215818.

18. Callard F., Perego E. How and why patients made Long Covid. Soc Sci Med 2021;268:113426. Doi: 10.1016/j.socscimed.2020.113426.

19. Sivan M., Taylor S. NICE guideline on long covid. Bmj 2020;371:m4938. Doi: 10.1136/bmj.m4938.

20. Tschöpe C., Ammirati E., Bozkurt B., et al. Myocarditis and inflammatory cardiomyopathy: current evidence and future directions. Nat Rev Cardiol 2021;18(3):169–93. Doi: 10.1038/s41569-020-00435-x.

21. García LF. Immune Response, Inflammation, and the Clinical Spectrum of COVID-19. Front Immunol 2020;11:1441. Doi: 10.3389/fimmu.2020.01441.

DocuSign Envelope ID: 4F4388D5-A568-4FAC-82D1-39CB3AB82D1B

22. Richter AG., Shields AM., Karim A., et al. Establishing the prevalence of common tissue-specific autoantibodies following severe acute respiratory syndrome coronavirus 2 infection. Clin Exp Immunol 2021;205(2):99–105. Doi: 10.1111/cei.13623.

23. Wang EY., Mao T., Klein J., et al. Diverse functional autoantibodies in patients with COVID-19. Nature 2021;595(7866):283–8. Doi: 10.1038/s41586-021-03631-y.

24. Lerma LA., Chaudhary A., Bryan A., Morishima C., Wener MH., Fink SL. Prevalence of Autoantibody Responses in Acute Coronavirus Disease 2019 (COVID-19). J Transl Autoimmun 2020;3:100073. Doi: 10.1016/j.jtauto.2020.100073.

25. Meisel C., Akbil B., Meyer T., et al. Mild COVID-19 despite autoantibodies to type I IFNs in Autoimmune-Polyendocrine-Syndrome Type 1 (APS-1). J Clin Invest 2021. Doi: 10.1172/jci150867.

26. Rodriguez-Perez AI., Labandeira CM., Pedrosa MA., et al. Autoantibodies against ACE2 and angiotensin type-1 receptors increase severity of COVID-19. J Autoimmun 2021;122:102683–102683. Doi: 10.1016/j.jaut.2021.102683.

27. Dotan A., Muller S., Kanduc D., David P., Halpert G., Shoenfeld Y. The SARS-CoV-2 as an instrumental trigger of autoimmunity. Autoimmun Rev 2021;20(4):102792–102792. Doi: 10.1016/j.autrev.2021.102792.

28. Peker BO., Şener AG., Aydoğmuş FK. Antinuclear antibodies (ANAs) detected by indirect immunofluorescence (IIF) method in acute COVID-19 infection; future roadmap for laboratory diagnosis. J Immunol Methods 2021;499:113174–113174. Doi: 10.1016/j.jim.2021.113174.

29. Chang SH., Minn D., Kim YK. Autoantibodies in moderate and critical cases of COVID-19. Clin Transl Sci 2021;14(5):1625–6. Doi: 10.1111/cts.13036.

30. Pascolini S., Vannini A., Deleonardi G., et al. COVID-19 and Immunological Dysregulation: Can Autoantibodies be Useful? Clin Transl Sci 2021;14(2):502–8. Doi: 10.1111/cts.12908.

31. Gao Z., Zhang H., Liu C., Dong K. Autoantibodies in COVID-19: frequency and function. Autoimmun Rev 2021;20(3):102754–102754. Doi: 10.1016/j.autrev.2021.102754.

32. Sacchi MC., Tamiazzo S., Stobbione P., et al. SARS-CoV-2 infection as a trigger of autoimmune response. Clin Transl Sci 2021;14(3):898–907. Doi: 10.1111/cts.12953.

33. Cervia C., Zurbuchen Y., Taeschler P., et al. Immunoglobulin signature predicts risk of post-acute COVID-19 syndrome. Nat Commun 2022;13(1):446. Doi: 10.1038/s41467-021-27797-1.

34. Cervia C., Nilsson J., Zurbuchen Y., et al. Systemic and mucosal antibody responses specific to SARS-CoV-2 during mild versus severe COVID-19. J Allergy Clin Immun 2021;147(2):545-557.e9. Doi: 10.1016/j.jaci.2020.10.040.

35. Wallukat G., Hohberger B., Wenzel K., et al. Functional autoantibodies against G-protein coupled receptors in patients with persistent Long-COVID-19 symptoms. J Transl Autoimmun 2021;4:100100. Doi: 10.1016/j.jtauto.2021.100100.

36. Figueredo MA., Rodriguez A., Ruiz-Yagüe M., et al. Autoantibodies against C-reactive protein: clinical associations in systemic lupus erythematosus and primary antiphospholipid syndrome. J Rheumatology 2006;33(10):1980–6.

37. Wen W., Su W., Tang H., et al. Immune cell profiling of COVID-19 patients in the recovery stage by single-cell sequencing. Cell Discov 2020;6(1):31. Doi: 10.1038/s41421-020-0168-9.

38. Cox RJ., Brokstad KA. Not just antibodies: B cells and T cells mediate immunity to COVID-19. Nat Rev Immunol 2020;20(10):1–2. Doi: 10.1038/s41577-020-00436-4.

39. Moody R., Wilson K., Flanagan KL., Jaworowski A., Plebanski M. Adaptive Immunity and the Risk of Autoreactivity in COVID-19. Int J Mol Sci 2021;22(16):8965. Doi: 10.3390/ijms22168965.

40. Aydemir E., Bayat AH., Ören B., Atesoglu HI., Göker YŞ., Özçelik KÇ. Retinal vascular findings in patients with COVID-19. Ther Adv Ophthalmol 2021;13:25158414211030420. Doi: 10.1177/25158414211030419.

41. Landecho MF., Yuste JR., Gándara E., et al. COVID-19 retinal microangiopathy as an in vivo biomarker of systemic vascular disease? J Intern Med 2021;289(1):116–20. Doi: 10.1111/joim.13156.

42. Hohberger B., Ganslmayer M., Lucio M., et al. Retinal Microcirculation as a Correlate of a Systemic Capillary Impairment After Severe Acute Respiratory Syndrome Coronavirus 2 Infection. Frontiers Medicine 2021;8:676554. Doi: 10.3389/fmed.2021.676554.

43. Khaddaj-Mallat R., Aldib N., Bernard M., et al. SARS-CoV-2 deregulates the vascular and immune functions of brain pericytes via Spike protein. Neurobiol Dis 2021;161:105561. Doi: 10.1016/j.nbd.2021.105561.

DocuSign Envelope ID: 4F4388D5-A568-4FAC-82D1-39CB3AB82D1B

44. Dirican A., Ildir S., Uzar T., Karaman I., Ozkaya S. The role of endotheliitis in COVID-19: Real-world experience of 11 190 patients and literature review for a pathophysiological map to clinical categorisation. Int J Clin Pract 2021;75(11):e14843. Doi: 10.1111/ijcp.14843.

45. Kirschenbaum D., Imbach LL., Rushing EJ., et al. Intracerebral endotheliitis and microbleeds are neuropathological features of COVID-19. Neuropath Appl Neuro 2021;47(3):454–9. Doi: 10.1111/nan.12677.

46. Tarnawski AS., Ahluwalia A. Endothelial cells and blood vessels are major targets for COVID-19-induced tissue injury and spreading to various organs. World J Gastroentero 2022;28(3):275–89. Doi: 10.3748/wjg.v28.i3.275.

47. McGonagle D., O'Donnell JS., Sharif K., Emery P., Bridgewood C. Immune mechanisms of pulmonary intravascular coagulopathy in COVID-19 pneumonia. Lancet Rheumatology 2020;2(7):e437–45. Doi: 10.1016/s2665-9913(20)30121-1.

48. Birnhuber A., Fließer E., Gorkiewicz G., et al. Between inflammation and thrombosis: endothelial cells in COVID-19. Eur Respir J 2021;58(3):2100377. Doi: 10.1183/13993003.00377-2021.

49. Huang L., Zhao P., Tang D., et al. Cardiac Involvement in Patients Recovered From COVID-2019 Identified Using Magnetic Resonance Imaging. Jacc Cardiovasc Imaging 2020;13(11):2330–9. Doi: 10.1016/j.jcmg.2020.05.004.

50. Brito D., Meester S., Yanamala N., et al. High Prevalence of Pericardial Involvement in College Student Athletes Recovering From COVID-19. Jacc Cardiovasc Imaging 2021;14(3):541–55. Doi: 10.1016/j.jcmg.2020.10.023.

51. Rajpal S., Tong MS., Borchers J., et al. Cardiovascular Magnetic Resonance Findings in Competitive Athletes Recovering From COVID-19 Infection. Jama Cardiol 2021;6(1):116–8. Doi: 10.1001/jamacardio.2020.4916.

52. Clark DE., Parikh A., Dendy JM., et al. COVID-19 Myocardial Pathology Evaluation in Athletes With Cardiac Magnetic Resonance (COMPETE CMR). Circulation 2021;143(6):609–12. Doi: 10.1161/circulationaha.120.052573.

53. Escher F., Pietsch H., Aleshcheva G., et al. Detection of viral SARS-CoV-2 genomes and histopathological changes in endomyocardial biopsies. Esc Hear Fail 2020;7(5):2440–7. Doi: 10.1002/ehf2.12805.

54. Pietsch H., Escher F., Aleshcheva G., et al. Proof of SARS-CoV-2 Genomes in Endomyocardial Biopsy with Latency After Acute Infection. Int J Infect Dis 2020;102:70–2. Doi: 10.1016/j.ijid.2020.10.012.

55. Laurence C., Haini M., Thiruchelvam T., et al. Endomyocardial Biopsy in a Pediatric Patient With Cardiac Manifestations of COVID-19. Circulation Hear Fail 2020;13(11):e007384–e007384. Doi: 10.1161/circheartfailure.120.007384.

56. Takahashi T., Ellingson MK., Wong P., et al. Sex differences in immune responses that underlie COVID-19 disease outcomes. Nature 2020;588(7837):315–20. Doi: 10.1038/s41586-020-2700-3.

57. Winau L., Baydes RH., Braner A., et al. High-sensitive troponin is associated with subclinical imaging biosignature of inflammatory cardiovascular involvement in systemic lupus erythematosus. Ann Rheum Dis 2018;77(11):1590. Doi: 10.1136/annrheumdis-2018-213661.

58. Mavrogeni S., Koutsogeorgopoulou L., Markousis-Mavrogenis G., et al. Cardiovascular magnetic resonance detects silent heart disease missed by echocardiography in systemic lupus erythematosus. Lupus 2017;27(4):564–71. Doi: 10.1177/0961203317731533.

59. Ishimori ML., Martin R., Berman DS., et al. Myocardial ischemia in the absence of obstructive coronary artery disease in systemic lupus erythematosus. Jacc Cardiovasc Imaging 2010;4(1):27–33. Doi: 10.1016/j.jcmg.2010.09.019.

60. Puntmann VO., D'Cruz D., Smith Z., et al. Native Myocardial T1 Mapping by Cardiovascular Magnetic Resonance Imaging in Subclinical Cardiomyopathy in Patients With Systemic Lupus Erythematosus. Circ: Cardiovasc Imaging 2018;6(2):295–301. Doi: 10.1161/circimaging.112.000151.

61. Hachulla A-L., Launay D., Gaxotte V., et al. Cardiac magnetic resonance imaging in systemic sclerosis: a cross-sectional observational study of 52 patients. Ann Rheum Dis 2009;68(12):1878–84. Doi: 10.1136/ard.2008.095836.

62. Rodriguez-Reyna TS., Morelos-Guzman M., Hernandez-Reyes P., et al. Assessment of myocardial fibrosis and microvascular damage in systemic sclerosis by magnetic resonance imaging and coronary angiotomography. Rheumatology 2014;54(4):647–54. Doi: 10.1093/rheumatology/keu350.

63. Pankuweit S., Klingel K. Viral myocarditis: from experimental models to molecular diagnosis in patients. Heart Fail Rev 2012;18(6):683–702. Doi: 10.1007/s10741-012-9357-4.

64. Holloway CJ., Ntusi N., Suttie J., et al. Comprehensive Cardiac Magnetic Resonance Imaging and Spectroscopy Reveal a High Burden of Myocardial Disease in HIV Patients. Circulation 2013;128(8):814–22. Doi: 10.1161/circulationaha.113.001719.

65. Luetkens JA., Doerner J., Schwarze-Zander C., et al. Cardiac Magnetic Resonance Reveals Signs of Subclinical Myocardial Inflammation in Asymptomatic HIV-Infected Patients. Circulation Cardiovasc Imaging 2016;9(3):e004091. Doi: 10.1161/circimaging.115.004091.

DocuSign Envelope ID: 4F4388D5-A568-4FAC-82D1-39CB3AB82D1B

66. Leuw P de., Arendt CT., Haberl AE., et al. Myocardial Fibrosis and Inflammation by CMR Predict Cardiovascular Outcome in People Living With HIV. Jacc Cardiovasc Imaging 2021;14(8):1548–57. Doi: 10.1016/j.jcmg.2021.01.042.

67. Ntusi N., O'Dwyer E., Dorrell L., et al. HIV-1–Related Cardiovascular Disease Is Associated With Chronic Inflammation, Frequent Pericardial Effusions, and Probable Myocardial Edema. Circulation Cardiovasc Imaging 2018;9(3):e004430. Doi: 10.1161/circimaging.115.004430.

68. Ravichandran S., Tang J., Grubbs G., et al. SARS-CoV-2 immune repertoire in MIS-C and pediatric COVID-19. Nat Immunol 2021;22(11):1452–64. Doi: 10.1038/s41590-021-01051-8.

69. Consiglio CR., Cotugno N., Sardh F., et al. The Immunology of Multisystem Inflammatory Syndrome in Children with COVID-19. Cell 2020;183(4):968-981.e7. Doi: 10.1016/j.cell.2020.09.016.

70. Whittaker E., Bamford A., Kenny J., et al. Clinical Characteristics of 58 Children With a Pediatric Inflammatory Multisystem Syndrome Temporally Associated With SARS-CoV-2. Jama 2020;324(3):259–69. Doi: 10.1001/jama.2020.10369.

71. Ueha S., Shand FHW., Matsushima K. Cellular and Molecular Mechanisms of Chronic Inflammation-Associated Organ Fibrosis. Front Immunol 2012;3:71. Doi: 10.3389/fimmu.2012.00071.

72. Knight JS., Caricchio R., Casanova JL., et al. The intersection of COVID-19 and autoimmunity. J Clin Invest 2021;131(24). Doi: 10.1172/jci154886.

73. Schiffrin EL., Hypertension CI of HRMRG on. Beyond blood pressure: the endothelium and atherosclerosis progression. Am J Hypertens 2002;15(S5):115S-122S. Doi: 10.1016/s0895-7061(02)03006-6.

74. Touyz RM. Molecular and cellular mechanisms in vascular injury in hypertension: role of angiotensin II – editorial review. Curr Opin Nephrol Hy 2005;14(2):125–31. Doi: 10.1097/00041552-200503000-00007.

75. Püntmann VO., Hussain MB., Mayr M., Xu Q., Singer DRJ. Role of oxidative stress in angiotensin-II mediated contraction of human conduit arteries in patients with cardiovascular disease. Vasc Pharmacol 2005;43(4):277–82. Doi: 10.1016/j.vph.2005.08.015.

76. Silhol F., Sarlon G., Deharo J-C., Vaïsse B. Downregulation of ACE2 induces overstimulation of the renin–angiotensin system in COVID-19: should we block the renin–angiotensin system? Hypertens Res 2020;43(8):854–6. Doi: 10.1038/s41440-020-0476-3.

77. Zaheer J., Kim H., Kim JS. Correlation of ACE2 with RAS components after Losartan treatment in light of COVID-19. Sci Rep-Uk 2021;11(1):24397. Doi: 10.1038/s41598-021-03921-5.

78. Ferrario CM., Jessup J., Chappell MC., et al. Effect of Angiotensin-Converting Enzyme Inhibition and Angiotensin II Receptor Blockers on Cardiac Angiotensin-Converting Enzyme 2. Circulation 2005;111(20):2605–10. Doi: 10.1161/circulationaha.104.510461.

79. Sinagra G., Porcari A., Gentile P., et al. Viral presence-guided immunomodulation in lymphocytic myocarditis: an update. Eur J Heart Fail 2021;23(2):211–6. Doi: 10.1002/ejhf.1969.

80. Caforio ALP., Pankuweit S., Arbustini E., et al. Current state of knowledge on aetiology, diagnosis, management, and therapy of myocarditis: a position statement of the European Society of Cardiology Working Group on Myocardial and Pericardial Diseases. Eur Heart J 2013;34(33):2636–48. Doi: 10.1093/eurheartj/eht210.

81. Frustaci A., Russo MA., Chimenti C. Randomized study on the efficacy of immunosuppressive therapy in patients with virus-negative inflammatory cardiomyopathy: the TIMIC study. Eur Heart J 2009;30(16):1995–2002. Doi: 10.1093/eurheartj/ehp249.

82. Everett BM., Cornel J., Lainscak M., et al. Anti-Inflammatory Therapy with Canakinumab for the Prevention of Hospitalization for Heart Failure. Circulation 2019;139(10):1289–99. Doi: 10.1161/circulationaha.118.038010.

83. Group RC., Horby P., Lim WS., et al. Dexamethasone in Hospitalized Patients with Covid-19. New Engl J Med 2020;384(8):693–704. Doi: 10.1056/nejmoa2021436.

84. Sheng CC., Sahoo D., Dugar S., et al. Canakinumab to reduce deterioration of cardiac and respiratory function in SARS-CoV-2 associated myocardial injury with heightened inflammation (canakinumab in Covid-19 cardiac injury: The three C study). Clin Cardiol 2020;43(10):1055–63. Doi: 10.1002/clc.23451.

85. Cabral-Marques O., Riemekasten G. Functional autoantibodies targeting G protein-coupled receptors in rheumatic diseases. Nat Rev Rheumatol 2017;13(11):648–56. Doi: 10.1038/nrrheum.2017.134.

86. Freitag H., Szklarski M., Lorenz S., et al. Autoantibodies to Vasoregulative G-Protein-Coupled Receptors Correlate with Symptom Severity, Autonomic Dysfunction and Disability in Myalgic Encephalomyelitis/Chronic Fatigue Syndrome. J Clin Medicine 2021;10(16):3675. Doi: 10.3390/jcm10163675.

DocuSign Envelope ID: 4F4388D5-A568-4FAC-82D1-39CB3AB82D1B

87. Cabral-Marques O., Halpert G., Schimke LF., et al. Autoantibodies targeting GPCRs and RAS-related molecules associate with COVID-19 severity. Nat Commun 2022;13(1):1220. Doi: 10.1038/s41467-022-28905-5.

88. Madamanchi NR., Vendrov A., Runge MS. Oxidative Stress and Vascular Disease. Arteriosclerosis Thrombosis Vasc Biology 2005;25(1):29–38. Doi: 10.1161/01.atv.0000150649.39934.13.

89. Bonaventura A., Vecchié A., Dagna L., et al. Endothelial dysfunction and immunothrombosis as key pathogenic mechanisms in COVID-19. Nat Rev Immunol 2021;21(5):319–29. Doi: 10.1038/s41577-021-00536-9.

90. Weber KT., Sun Y., Bhattacharya SK., Ahokas RA., Gerling IC. Myofibroblast-mediated mechanisms of pathological remodelling of the heart. Nat Rev Cardiol 2012;10(1):15–26. Doi: 10.1038/nrcardio.2012.158.

91. Landmesser U., Wollert KC., Drexler H. Potential novel pharmacological therapies for myocardial remodelling. Cardiovasc Res 2009;81(3):519–27. Doi: 10.1093/cvr/cvn317.

92. Husain K., Hernandez W., Ansari RA., Ferder L. Inflammation, oxidative stress and renin angiotensin system in atherosclerosis. World J Biological Chem 2015;6(3):209–17. Doi: 10.4331/wjbc.v6.i3.209.

93. Morgan BJ. Vascular consequences of intermittent hypoxia. Adv Exp Med Biol 2007;618:69–84. Doi: 10.1007/978-0-387-75434-5_6.

94. Vallés PG., Bocanegra V., Costantino VV., Lorenzo AFG., Benardon ME., Cacciamani V. The renal antioxidative effect of losartan involves heat shock protein 70 in proximal tubule cells. Cell Stress Chaperones 2020;25(5):753–66. Doi: 10.1007/s12192-020-01119-8.

95. Brenner BM., Cooper ME., Zeeuw D de., Investigators RS. Effects of losartan on renal and cardiovascular outcomes in patients with type 2 diabetes and nephropathy. Acc Curr J Rev 2002;11(1):26. Doi: 10.1016/s1062-1458(01)00549-9.

96. Dahlöf B., Devereux RB., Kjeldsen SE., et al. Cardiovascular morbidity and mortality in the Losartan Intervention For Endpoint reduction in hypertension study (LIFE): a randomised trial against atenolol. Lancet 2002;359(9311):995–1003. Doi: 10.1016/s0140-6736(02)08089-3.

97. Goel SS., Kleiman NS., Zoghbi WA., Reardon MJ., Kapadia SR. Renin-Angiotensin System Blockade in Aortic Stenosis: Implications Before and After Aortic Valve Replacement. J Am Hear Assoc Cardiovasc Cerebrovasc Dis 2020;9(18):e016911. Doi: 10.1161/jaha.120.016911.

98. Shimada YJ., Passeri JJ., Baggish AL., et al. Effects of losartan on left ventricular hypertrophy and fibrosis in patients with nonobstructive hypertrophic cardiomyopathy. Jacc Hear Fail 2013;1(6):480–7. Doi: 10.1016/j.jchf.2013.09.001.

99. Araujo AQ., Arteaga E., Ianni BM., Buck PC., Rabello R., Mady C. Effect of Losartan on Left Ventricular Diastolic Function in Patients With Nonobstructive Hypertrophic Cardiomyopathy. Am J Cardiol 2005;96(11):1563–7. Doi: 10.1016/j.amjcard.2005.07.065.

100. Gulati G., Heck SL., Geisler J., et al. EFFECT OF CANDESARTAN AND METOPROLOL ON SUBCLINICAL MYOCARDIAL INJURY DURING ANTHRACYCLINE THERAPY: DATA FROM THE PREVENTION OF CARDIAC DYSFUNCTION DURING ADJUVANT BREAST CANCER THERAPY (PRADA) STUDY. J Am Coll Cardiol 2016;67(13):1530. Doi: 10.1016/s0735-1097(16)31531-5.

101. Effects of an Angiotensin-Converting–Enzyme Inhibitor, Ramipril, on Cardiovascular Events in High-Risk Patients. New Engl J Medicine 2000;342(10):748–748. Doi: 10.1056/nejm200003093421023.

102. Visseren FLJ., Mach F., Smulders YM., et al. 2021 ESC Guidelines on cardiovascular disease prevention in clinical practice. Eur Heart J 2021;42(34):3227–337. Doi: 10.1093/eurheartj/ehab484.

103. Gulati G., Heck SL., Ree AH., et al. Prevention of cardiac dysfunction during adjuvant breast cancer therapy (PRADA): a 2 × 2 factorial, randomized, placebo-controlled, double-blind clinical trial of candesartan and metoprolol. Eur Heart J 2016;37(21):1671–80. Doi: 10.1093/eurheartj/ehw022.

104. Rauti R., Shahoha M., Leichtmann-Bardoogo Y., et al. Effect of SARS-CoV-2 proteins on vascular permeability. Elife 2021;10:e69314. Doi: 10.7554/elife.69314.

105. Nader D., Fletcher N., Curley GF., Kerrigan SW. SARS-CoV-2 uses major endothelial integrin αvβ3 to cause vascular dysregulation in-vitro during COVID-19. Plos One 2021;16(6):e0253347. Doi: 10.1371/journal.pone.0253347.

106. Puntmann VO., Peker E., Chandrashekhar Y., Nagel E. T1 Mapping in Characterizing Myocardial Disease. Circ Res 2016;119(2):277–99. Doi: 10.1161/circresaha.116.307974.

107. Puntmann VO., Valbuena S., Hinojar R., et al. Society for Cardiovascular Magnetic Resonance (SCMR) expert consensus for CMR imaging endpoints in clinical research: part I - analytical validation and clinical qualification. J Cardiovasc Magn Reson : Off J Soc Cardiovasc Magn Reson 2018;20(1):67. Doi: 10.1186/s12968-018-0484-5.

DocuSign Envelope ID: 4F4388D5-A568-4FAC-82D1-39CB3AB82D1B

108. Brugada J., Katritsis DG., Arbelo E., et al. 2019 ESC Guidelines for the management of patients with supraventricular tachycardiaThe Task Force for the management of patients with supraventricular tachycardia of the European Society of Cardiology (ESC)Developed in collaboration with the Association for European Paediatric and Congenital Cardiology (AEPC). Eur Hear J 2019;41(5):655–720. Doi: 10.1093/eurheartj/ehz467.

109. Shah MM., Joyce B., Plumb ID., et al. Paxlovid Associated with Decreased Hospitalization Rate Among Adults with COVID-19 — United States, April–September 2022. Morb Mortal Wkly Rep 2022;71(48):1531–7. Doi: 10.15585/mmwr.mm7148e2.

110. Hammond J., Leister-Tebbe H., Gardner A., et al. Oral Nirmatrelvir for High-Risk, Nonhospitalized Adults with Covid-19. N Engl J Med 2022;386(15):1397–408. Doi: 10.1056/nejmoa2118542.

111. Dorian P., Cvitkovic SS., Kerr CR., et al. A novel, simple scale for assessing the symptom severity of atrial fibrillation at the bedside: The CCS-SAF Scale. Can J Cardiol 2006;22(5):383–6. Doi: 10.1016/s0828-282x(06)70922-9.

112. Paladini L., Hodder R., Cecchini I., Bellia V., Incalzi RA. The MRC dyspnoea scale by telephone interview to monitor health status in elderly COPD patients. Resp Med 2010;104(7):1027–34. Doi: 10.1016/j.rmed.2009.12.012.

113. Pichler P., Pichler-Cetin E., Vertesich M., et al. Ivabradine Versus Metoprolol for Heart Rate Reduction Before Coronary Computed Tomography Angiography. Am J Cardiol 2012;109(2):169–73. Doi: 10.1016/j.amjcard.2011.08.025.

114. Cademartiri F., Garot J., Tendera M., Zamorano JL. Intravenous ivabradine for control of heart rate during coronary CT angiography: A randomized, double-blind, placebo-controlled trial. J Cardiovasc Comput 2015;9(4):286–94. Doi: 10.1016/j.jcct.2015.04.005.

115. Hicks KA., Mahaffey KW., Mehran R., et al. 2017 Cardiovascular and Stroke Endpoint Definitions for Clinical Trials. Circulation 2018;137(9):961–72. Doi: 10.1161/circulationaha.117.033502.

116. Jaffe AS., Morrow DA., Group ESD., et al. Fourth universal definition of myocardial infarction (2018). Eur Heart J 2018;40(3):237–69. Doi: 10.1093/eurheartj/ehy462.

117. Grothues F., Smith GC., Moon JCC., et al. Comparison of interstudy reproducibility of cardiovascular magnetic resonance with two-dimensional echocardiography in normal subjects and in patients with heart failure or left ventricular hypertrophy. Am J Cardiol 2002;90(1):29–34. Doi: 10.1016/s0002-9149(02)02381-0.

118. Lang RM., Badano LP., Mor-Avi V., et al. Recommendations for Cardiac Chamber Quantification by Echocardiography in Adults: An Update from the American Society of Echocardiography and the European Association of Cardiovascular Imaging. J Am Soc Echocardiog 2015;28(1):1-39.e14. Doi: 10.1016/j.echo.2014.10.003.

119. Rodríguez-Santamarta M., Minguito-Carazo C., Echarte-Morales JC., Castillo-García SD., Valdivia-Ruiz J., Fernández-Vázquez F. Echocardiographic findings in critical patients with COVID-19. Revista Espanola De Cardiol Engl Ed 2020;73(10):861–3. Doi: 10.1016/j.rec.2020.06.030.

120. Pitt B., Segal R., Martinez FA., et al. Randomised trial of losartan versus captopril in patients over 65 with heart failure (Evaluation of Losartan in the Elderly Study, ELITE). Lancet 1997;349(9054):747–52. Doi: 10.1016/s0140-6736(97)01187-2.

121. Lang RM., Elkayam U., Yellen LG., et al. Comparative effects of losartan and enalapril on exercise capacity and clinical status in patients with heart failure. The Losartan Pilot Exercise Study Investigators. J Am Coll Cardiol 1997;30(4):983–91. Doi: 10.1016/s0735-1097(97)00253-2.

122. Cooper LT. Ventricular Arrhythmias and Sudden Cardiac Death in Lymphocytic Myocarditis ∗. J Am Coll Cardiol 2020;75(9):1058–60. Doi: 10.1016/j.jacc.2020.01.032.

123. Nagel E., Greenwood JP., McCann GP., et al. Magnetic Resonance Perfusion or Fractional Flow Reserve in Coronary Disease. New Engl J Med 2019;380(25):2418–28. Doi: 10.1056/nejmoa1716734.

124. Kieser M., Friede T. Simple procedures for blinded sample size adjustment that do not affect the type I error rate. Stat Med 2003;22(23):3571–81. Doi: 10.1002/sim.1585.

125. Manali ED., Stathopoulos GT., Kollintza A., et al. The Medical Research Council chronic dyspnea score predicts the survival of patients with idiopathic pulmonary fibrosis. Resp Med 2007;102(4):586–92. Doi: 10.1016/j.rmed.2007.11.008.

DocuSign Envelope ID: 4F4388D5-A568-4FAC-82D1-39CB3AB82D1B

17 Normative references

World Medical Association Declaration of Helsinki - ETHICAL PRINCIPLES FOR MEDICAL RESEARCH INVOLVING HUMAN SUBJECTS Link
Regulation (EU) No 536/2014 of the European Parliament and of the Council of 16 April 2014 on clinical trials on medicinal products for human use, and repealing Directive 2001/20/EC (Text with EEA relevance)Text with EEA relevance Link
Detailed guidance on the request to the competent authorities for authorisation of a clinical trial on a medicinal product for human use, the notification of substantial amendments and the declaration of the end of the trial (CT-1) 2010/C 82/01: Link
Detailed guidance on the application format and documentation to be submitted in an application for an Ethics Committee opinion on the clinical trial on medicinal products for human use (2006). Link
ICH E6 (R2) Good clinical practice Link
ICH E9 Statistical Principles for Clinical Trials Link
ICH E2A Clinical safety data management: definitions and standards for expedited reporting Link
The General Data Protection Regulation (GDPR) Regulation (EU) 2016/679 Link
Guideline on data monitoring committees"(Doc. Ref. EMEA/CHMP /EWP /5872/03 Corr Link

DocuSign Envelope ID: 4F4388D5-A568-4FAC-82D1-39CB3AB82D1B

18 Appendices

18.1 Definitions according ICH Guidelines for Clinical Safety Data Management, Definitions and Standards for Expedited Reporting, Topic E2

An adverse event is any untoward medical occurrence in a participant or clinical investigation subject, administered a pharmaceutical product and which does not necessarily have to have a causal relationship with this treatment.

Adverse reactions are all untoward and unintended responses to an investigational medicinal product related to any dose administered.

A serious adverse event or serious adverse reaction is any experience that suggests a significant hazard, contraindication, side effect or precaution. It is any Adverse Event that at any dose fulfils at least one of the following criteria:

- is fatal (results in death) (NOTE: death is an outcome, not an event)
- is life-threatening (NOTE: the term "life-threatening" refers to an event in which the participant was at immediate risk of death at the time of the event; it does not refer to an event which could hypothetically have caused a death had it been more severe.)
- required in-patient hospitalization or prolongation of existing hospitalization
- results in persistent or significant disability / incapacity
- is a congenital anomaly / birth defect
- is medically significant or requires intervention to prevent one or other of the outcomes listed above

Medical and scientific judgment should be exercised in deciding whether expedited reporting to the sponsor is appropriate in other situations, such as important medical events that may not be immediately life-threatening or result in death or hospitalization but may jeopardize the participant or may require intervention to prevent one of the outcomes listed in the definitions above. These situations should also usually be considered serious.

Examples of such events are intensive treatment in an emergency room or at home for allergic bronchospasm; blood dyscrasias or convulsions that do not result in hospitalization; or development of drug dependency or drug abuse.

An unexpected Adverse Event is one, the nature or severity of which is not consistent with the applicable summary of medicinal product characteristics.

Causality is initially assessed by the investigator. With respect to report and documentation obligation (regulatory authorities, ethics committees and other investigators) for Serious Adverse Events, causality can be one of 2 possibilities:

YES: Certain, probable or possible: Reasonable evidence or argument to suggest a suspected causal relationship (ENTR / CT 3, 6.2.2, Annex 1).
(USA: A reasonably related AE is one that is possibly, probably, or definitely related to study agent)
Or
NO: Unlikely: When there is sufficient information to accept a lack of a causal relationship, in the sense of impossible and improbable.

A suspected unexpected serious adverse reaction (SUSAR) is a serious adverse reaction, the nature, or severity of which is not consistent with the applicable summary of medicinal product characteristics.

It is important that the severity of an adverse event is not confounded with the seriousness of the event. For example, vomiting which persists for many hours may be severe, but is not necessarily a serious adverse event. On the other hand, stroke which results in only a limited degree of disability may be considered a mild stroke but would be a serious adverse event.

Such preliminary reports will be followed by detailed descriptions later which will include copies of hospital case reports, autopsy reports and other documents.

For serious adverse events, the following must be assessed and recorded on the adverse events page of the Case Report Form: intensity, relationship to test substance, action taken, and outcome to date.

Document and report obligation have to be adhered according to the national and international laws and regulations.

DocuSign Envelope ID: 4F4388D5-A568-4FAC-82D1-39CB3AB82D1B

18.2 Attachments

18.2.1 Myoflame-19 Study Information (Webpage, Flyer)

Ihre COVID19-Infektion liegt mehr als 4 Wochen zurück. Sie leiden an Symptomen wie Belastungsintoleranz, Kurzatmigkeit, Engegefühl in der Brust, ziehende oder brennende Schmerzen in der Brust oder Herzklopfen.
Dann suchen wir möglicherweise genau SIE!
Sie dürfen keine vorbestehenden Herzerkrankungen haben und mussten während der akuten COVID-19 Erkrankung nicht im Krankenhaus betreut werden.

Myoflame-19 Studie Information
Nach COVID-19 bestehen häufig noch nach Wochen Beschwerden, die auf Veränderungen des Herz-Kreislaufsystems zurückzuführen sind. Hierzu gehören Belastungsintoleranz, Kurzatmigkeit, Engegefühl in der Brust, ziehende oder brennende Schmerzen in der Brust und Herzklopfen. Diese Beschwerden treten unabhängig von vorbestehenden Herzkrankheiten oder Grundkrankheiten auf.
Es gibt derzeit keine Standardtherapie für diese Beschwerden. Mit der Studie **Myoflame-19** wollen wir überprüfen, ob eine frühe Anwendung von Herzmitteln und Entzündung-Hemmern langfristige schädliche Auswirkung am Herz-Kreislaufsystem vermeiden können. Nach einer ersten Herz-MRT Untersuchung wird entschieden, ob Sie für eine Medikamentengabe in Betracht kommen. Wenn ja, erhalten Sie nach dem Zufallsprinzip entweder ein Medikament oder deren Placebo. Die Wirkung wird nach 4 Monaten durch eine zweite Herz-MRT Untersuchung erfasst. Zusätzlich prüfen wir, ob es mit Medikamenten schneller zu einer Verbesserung der Symptome kommt.

Die Studie wird von [redacted] und [redacted] des Universitätsklinikums Frankfurt konzipiert und geleitet, die Durchführung der Studie erfolgt in Kooperation mit 4 weiteren Krankenhäusern (innerhalb Deutschlands und Österreich).

Für die Studie werden Erwachsene Teilnehmer mit einer dokumentierten COVID-19 Infektion gesucht, die mindestens 4 Wochen zurück liegt. Sie leiden an fortbestehenden Beschwerden, die Sie vor der Infektion nicht hatten, wie Belastungsintoleranz, Kurzatmigkeit, Engegefühl in der Brust, ziehende oder brennende Schmerzen in der Brust oder Herzklopfen. Sie haben keine ausgeprägten vorbekannten Erkrankungen des Herzens, der Lunge oder anderer Organe. Während der akuten COVID-19 Infektion dürften Sie nicht im Krankenhaus betreut worden sein.

Link zu weiteren Informationen Webseite: www.myoflame.com

Sollten Sie an der Studie Interesse haben bitten wir Sie, sich über das folgende Formular zu registrieren. Im Anschluss werden wir Ihnen noch ein Formular mit Fragen über Ihren allgemeinen Gesundheitszustand auf Sie mittels verschlüsselter Email zukommen lassen (Dauer circa 10 Minuten) oder werden Sie telefonisch kontaktiert. Nach interner Prüfung Ihrer Angaben werden Sie zum einen ersten Termin zu Baseline Evaluierung eingeladen. Dies wird von dem Studienzentrum koordiniert, dass ihrem Wohnort am nächsten liegt. Wir versuchen innerhalb einer Woche alle geeigneten Participanten zu kontaktieren. Wenn Sie nach 3 Wochen keine Rückmeldung erhalten haben, wurden die Einschlusskriterien für die Teilnahme an der Studie erfüllt. Bitte sehen Sie dann von weiteren Nachfragen ab.

DocuSign Envelope ID: 4F4388D5-A568-4FAC-82D1-39CB3AB82D1B

18.2.2 Myoflame-19 Study Onlineregistration (https://www.myoflame.com/kontakt)

Name *
->Name

Vorname *
->Vorname

Telefonnummer *
->Telefonnummer

E-Mail-Adresse *
->E-Mail-Adresse
E-Mail-Adresse bestätigen *
->E-Mail-Adresse bestätigen

PLZ *
->PLZ

Ort *
->Ort

Mit dem Absenden meiner Daten erkläre ich mich damit einverstanden, dass meine Kontaktdaten und meine medizinischen Angaben zu den Gesundheitsfragen von Mitarbeitern des Instituts für experimentelle und translationale kardiovaskuläre Bildgebung, Universitätsklinikum Frankfurt, Theodor-Stern-Kai 7, 60590 Frankfurt am Maingespeichert und zu Zwecken der Vorprüfung auf meine Eignung zur Studienteilnahme und zur Kontaktaufnahme in Bezug auf meine Anfrage verarbeitet werden.

Personenbezogene Daten (Privatadresse, Kontaktinformationen und die Gesundheitsdaten) werden selbstverständlich vertraulich behandelt und nicht an Dritte weitergegeben. Weitere Informationen über die Datenverarbeitung gemäß Artikel 13&14 DSGVO befindet sich in der beiliegenden Broschüre**„INFORMATIONSPFLICHT bei der Erhebung personenbezogener Daten im Universitätsklinikum Frankfurt gemäß der EU-Datenschutz-Grundverordnung (DSGVO)“**.

Abweichend zu Kontaktformularen für allgemeine Fragen werden hier auch medizinische Daten erfasst. Für diese Daten gilt: Wenn es zu keiner Studienteilnahme kommen sollte, werden Ihre in diesem Formular eingetragenen Daten nach Zusendung der entsprechenden Antwort an Sie wieder gelöscht. Sollten Sie für die Studie in Frage kommen, erhalten Sie automatisch vor Studienteilnahme weitere Informationen zur Datenverarbeitung vom teilnehmenden ausgewähltem Prüfzentrum.
Mit dem Absenden meiner Daten erkläre ich mich ebenfalls damit einverstanden, dass meine erhobenen Daten pseudonymisiert in einem gesicherten elektronischen Prüfbogen (electronic Case Report Form, eCRF) gespeichert werden für die gesamte Aufbewahrungsfrist der klinischen Prüfung. Ich habe die vorstehende Erklärung gelesen und bin damit einverstanden.

DocuSign Envelope ID: 4F4388D5-A568-4FAC-82D1-39CB3AB82D1B

18.2.3 Myoflame-19 Study Information Screening Questionnaire (per Phone or via verschluesselte Email)

Participant Identifier: ___________________
(vom Prüfzentrum auszufüllen)

Vorname*:
Nachname*:
Telefon (Mobil)*:
E-Mail*:
Anschrift:
Postleitzahl*:
Ort:

Geschlecht:

- ☐ Weiblich
- ☐ Männlich
- ☐ Divers

Gewicht (kg):
Alter (in Jahren):
Größe (in cm):
Datum Ihrer COVID19 Infektion:
Mit welchem Test nachgewiesen:

- ☐ Schnelltest
- ☐ PCR
- ☐ Antikörper Nachweis einer abgelaufenen COVID Infektion
- ☐ Krankschreibung im Rahmen der COVID Infektion
- ☐ Andere (bitte beschreiben):_________________________________

Nehmen Sie bereits an einer anderen klinischen Studie teil oder planen dies:

- ☐ Ja
- ☐ Nein

Informationen bezüglich der MRT-Untersuchung:

- ☐ Ich hatte schon einmal eine MRT-Untersuchung
- ☐ Ich habe eine Allergie gegen MRT-Kontrastmittel
- ☐ Ich habe metallische Implantate
- ☐ Ich leider unter Platzangst
- ☐ keine der genannten Optionen

COVID Daten:

- ☐ Ich wurde wegen COVID im Krankenhaus aufgenommen
- ☐ Ich hatte bereits eine frühere COVID Infektion
- ☐ Ich bin 1x gegen COVID geimpft
- ☐ Ich bin 2x gegen COVID geimpft
- ☐ Ich bin 3x oder mehrfach gegen COVID geimpft

Jetzige Beschwerden:

- ☐ Brustschmerzen /Engegefühl
- ☐ Herzklopfen /Herzrasen/hoher Ruhepuls
- ☐ Kurzatmigkeit / Belastungsintoleranz
- ☐ Keine der genannten Beschwerden

Zu Ihrer Gesundheit:

- ☐ Ich habe keine Vorerkrankungen
- ☐ Ich habe Diabetes
- ☐ Ich habe erhöhten Blutdruck
- ☐ Ich habe erhöhtes Cholesterin
- ☐ Ich bin Raucher
- ☐ Ich habe eine bekannte Herzerkrankung
- ☐ Ich habe eine bekannte Lungenerkrankung
- ☐ Ich habe eine Nierenerkrankung

DocuSign Envelope ID: 4F4388D5-A568-4FAC-82D1-39CB3AB82D1B

☐ Ich hatte einen Schlaganfall bzw. habe Epilepsie
☐ Ich hatte/habe eine Tumorerkrankung (Krebs)
☐ Ich habe Rheuma /Autoimmunerkrankung
☐ Ich habe andere chronische Erkrankungen
☐ Ich wurde schon einmal operiert
☐ Ich nehme Medikamente

Wenn ja, bitte Auflistung aller Medikamente:

____

____

____

Wurden Sie innerhalb der letzten 6 Monate mit einem der folgenden Medikamente behandelt:

☐ ACE-Hemmer (z.B. Ramipril, Lisinopril, etc.)
☐ Angiotensin-Rezeptor-Blocker (z.B. Losartan, Candesartan, Valsartan, Entresto, etc.)
☐ Prednisolon
☐ Immunsuppressiva
☒ Keine von genannten Medikamenten

______________________________ ______________________________

Name, Vorname (in Buchstaben), Rolle Datum, Signatur

Datenschutzerklärung

Mit dem Absenden meiner Daten erkläre ich mich damit einverstanden, dass meine Kontaktdaten und meine medizinischen Angaben zu den Gesundheitsfragen von den Mitarbeitern von [Prüfzentrum] gespeichert und zu Zwecken der Vorprüfung auf meine Eignung zur Studienteilnahme und zur Kontaktaufnahme in Bezug auf meine Anfrage verarbeitet werden. Personenbezogene Daten (Privatadresse, Kontaktinformationen und die Gesundheitsdaten) werden selbstverständlich vertraulich behandelt und nicht an Dritte weitergegeben.

Weitere Informationen über die Datenverarbeitung gemäß Artikel 13&14 DSGVO befindet sich in der beiliegenden Broschüre „INFORMATIONSPFLICHT bei der Erhebung personenbezogener Daten im Universitätsklinikum Frankfurt gemäß der EU-Datenschutz-Grundverordnung (DSGVO)“.

Abweichend zu Kontaktformularen für allgemeine Fragen werden hier auch medizinische Daten erfasst. Sollten Sie für die Studie in Frage kommen, erhalten Sie automatisch vor Studienteilnahme weitere Informationen zur Datenverarbeitung vom teilnehmenden ausgewähltem Prüfzentrum. Wenn es zu keiner Studienteilnahme kommen sollte, werden Ihre in diesem Formular eingetragenen Daten nach Erreichen der erforderlichen Patientenzahlen statistisch zusammengefasst und dann gelöscht.

Mit dem Absenden meiner Daten erkläre ich mich ebenfalls damit einverstanden, dass meine erhobenen Daten pseudonymisiert in einem gesicherten elektronischen Prüfbogen (electronic Case Report Form, eCRF) gespeichert werden für die gesamte Aufbewahrungsfrist der klinischen Prüfung. Ich habe die vorstehende Erklärung gelesen und bin damit einverstanden. *

18.2.4 Long COVID Questionnaire (Sudre et al), in German.

Bestehen bei Ihnen die folgenden Beschwerden:			
FA	Fatigue (Fatigue)	0-no (nein), 1-yes (Ja), 99-unknown (unbekannt)	
PEM	Post-exertional Malaise*	0-no (nein), 1-yes (Ja), 99-unknown (unbekannt)	

HA	Headache (Kopfschmerzen)	0-no (nein), 1-yes (Ja), 99-unknown (unbekannt)	
POTS	Excessive Tachycardia, Herzrasen (POTS)	0-no (nein), 1-yes (Ja), 99-unknown (unbekannt)	
SOB	Shortness of breath (Kurzatmigkeit)	0-no (nein), 1-yes (Ja), 99-unknown (unbekannt)	
LOS	Loss of smell (Geruchsverlust)	0-no (nein), 1-yes (Ja), 99-unknown (unbekannt)	
PC	Persistent cough (Hustenreiz)	0-no (nein), 1-yes (Ja), 99-unknown (unbekannt)	
ST	Sore throat (Halsschmerzen)	0-no (nein), 1-yes (Ja), 99-unknown (unbekannt)	
FV	Fever (Fieber)	0-no (nein), 1-yes (Ja), 99-unknown (unbekannt)	
UMP	Unusual muscle pains (Muskel/Gliederschmerzen)	0-no (nein), 1-yes (Ja), 99-unknown (unbekannt)	
SM	Skipped meals (Übersprungene Mahlzeiten)	0-no (nein), 1-yes (Ja), 99-unknown (unbekannt)	
CP	Chest Pain (Brustschmerzen)	0-no (nein), 1-yes (Ja), 99-unknown (unbekannt)	When yes - > Modified CCS
DI	Diarrhoea (Durchfall)	0-no (nein), 1-yes (Ja), 99-unknown (unbekannt)	
HV	Hoarse Voice (Heisere Stimme)	0-no (nein), 1-yes (Ja), 99-unknown (unbekannt)	
AP	Abdominal Pain (Bauchschmerzen)	0-no (nein), 1-yes (Ja), 99-unknown (unbekannt)	
DE	Brain Fog (Brain Fog)	0-no (nein), 1-yes (Ja), 99-unknown (unbekannt)	
LOC	Loss of Consciousness (Ohmacht)	0-no (nein), 1-yes (Ja), 99-unknown (unbekannt)	When yes: -> Near faint (Nahezu ohnmächtig) -> Syncope (Synkope) - > Reanimation

*Post-Exertional Malaise (PEM) ist die Verschlechterung der Symptome nach selbst geringer körperlicher oder geistiger Anstrengung, wobei sich die Symptome typischerweise 12 bis 48 Stunden nach der Aktivität verschlimmern und Tage oder sogar Wochen anhalten.

DocuSign Envelope ID: 4F4388D5-A568-4FAC-82D1-39CB3AB82D1B

18.2.5 Modified Chest Discomfort Severity Score

Chest discomfort was graded using a modified Chest Discomfort Scale based on the Canadian Chest Pain Scale(111), which was not limited to the typical anginal-type of chest pain, but also included, deep, dull, pulling, burning or sharp chest discomfort or tightness, radiating into neck, back, shoulders or arms. To differentiate this from precordial catch symptoms and abdominal stiches, the criterion for chest pain was if lasting for more than 10 minutes.

The original Chest Pain Scale	Description of Symptoms (Beschreibung der Beschwerden)
0	No symptoms (Keine Beschwerden)
I	Presence of chest discomfort during strenuous, rapid, or prolonged ordinary activity (walking or climbing the stairs).<br>(Vorhandensein von Brustbeschwerden während anstrengender, schneller oder längerer normaler Aktivität (Gehen oder Treppensteigen).
II	Presence of chest discomfort during or after ordinary activities, when they are performed rapidly, or by change of position, under emotional stress, but also walking uphill, climbing more than one flight of ordinary stairs at a normal pace and under normal conditions.<br>(Vorhandensein von Brustbeschwerden während oder nach gewöhnlichen Aktivitäten, wenn sie schnell ausgeführt werden, oder durch Positionswechsel, unter emotionalem Stress, aber auch beim Bergaufgehen, beim Steigen von mehr als einer gewöhnlichen Treppe in normalem Tempo und unter normalen Bedingungen.)
III	Presence of chest discomfort during or after activities of daily life at normal pace and conditions. (Vorhandensein von Brustbeschwerden während oder nach Aktivitäten des täglichen Lebens bei normalem Tempo und normalen Bedingungen.)
IV	No exertion needed to trigger chest pain, present at rest, recurring, or present at all times. (Keine Anstrengung erforderlich, um Brustschmerzen auszulösen, die in Ruhe vorhanden, wiederkehrend oder jederzeit vorhanden sind.)

18.2.6 MRC Dyspnea Severity Score

Dyspnoea was graded using modified Medical Research Council Dyspnea(125).

MRC Dyspnoea Scale	Description of Symptoms (Beschreibung der Beschwerden)
0	No symptoms (Keine Beschwerden)
1	Breathless with strenuous exercise (Atemlos bei anstrengender Belastung)
2	Short of breath when hurrying on the level or walking up a slight hill (Kurzatmigkeit, wenn man auf der Ebene eilt oder einen leichten Hügel hinaufgeht)
3	Walks slower than people of the same age on the level or stops for breath while walking at own pace on the level (Es geht langsamer als Gleichaltrige auf der Ebene oder hält an, um Luft zu holen, während er in seinem eigenen Tempo auf der Ebene geht)
4	Stops for breath after walking 100m (Atempause nach 100m Gehen)
5	Too breathless to leave the house or breathless when dressing (Zu atemlos, um das Haus zu verlassen oder atemlos beim Anziehen)

18.2.7 RAND 36-item Health Survey, Version 2.

This Quality-of-Life Survey will be completed on paper for BL and W16 by the participant and transferred to the eCRF by the site staff

RAND CORPORATION HEALTH

36-Item Short Form Survey Instrument (SF-36)

RAND 36-Item Health Survey 1.0 Questionnaire Items

Choose one option for each questionnaire item.

1. In general, would you say your health is:

- 1 - Excellent
- 2 - Very good
- 3 - Good
- 4 - Fair
- 5 - Poor

---

2. **Compared to one year ago**, how would you rate your health in general **now**?

- 1 - Much better now than one year ago
- 2 - Somewhat better now than one year ago
- 3 - About the same
- 4 - Somewhat worse now than one year ago
- 5 - Much worse now than one year ago

During the **past 4 weeks**, have you had any of the following problems with your work or other regular daily activities **as a result of your physical health?**

	Yes	No
13. Cut down the **amount of time** you spent on work or other activities	◯ 1	◯ 2
14. **Accomplished less** than you would like	◯ 1	◯ 2
15. Were limited in the **kind** of work or other activities	◯ 1	◯ 2
16. Had **difficulty** performing the work or other activities (for example, it took extra effort)	◯ 1	◯ 2

During the **past 4 weeks**, have you had any of the following problems with your work or other regular daily activities **as a result of any emotional problems** (such as feeling depressed or anxious)?

	Yes	No
17. Cut down the **amount of time** you spent on work or other activities	◯ 1	◯ 2
18. **Accomplished less** than you would like	◯ 1	◯ 2
19. Didn't do work or other activities as **carefully** as usual	◯ 1	◯ 2

20. During the **past 4 weeks**, to what extent has your physical health or emotional problems interfered with your normal social activities with family, friends, neighbors, or groups?

◯ 1 - Not at all

◯ 2 - Slightly

◯ 3 - Moderately

◯ 4 - Quite a bit

◯ 5 - Extremely

The following items are about activities you might do during a typical day. Does **your health now limit you** in these activities? If so, how much?

	Yes, limited a lot	Yes, limited a little	No, not limited at all
3. **Vigorous activities**, such as running, lifting heavy objects, participating in strenuous sports	◯ 1	◯ 2	◯ 3
4. **Moderate activities**, such as moving a table, pushing a vacuum cleaner, bowling, or playing golf	◯ 1	◯ 2	◯ 3
5. Lifting or carrying groceries	◯ 1	◯ 2	◯ 3
6. Climbing **several** flights of stairs	◯ 1	◯ 2	◯ 3
7. Climbing **one** flight of stairs	◯ 1	◯ 2	◯ 3
8. Bending, kneeling, or stooping	◯ 1	◯ 2	◯ 3
9. Walking **more than a mile**	◯ 1	◯ 2	◯ 3
10. Walking **several blocks**	◯ 1	◯ 2	◯ 3
11. Walking **one block**	◯ 1	◯ 2	◯ 3
12. Bathing or dressing yourself	◯ 1	◯ 2	◯ 3

These questions are about how you feel and how things have been with you **during the past 4 weeks.** For each question, please give the one answer that comes closest to the way you have been feeling.

How much of the time during the **past 4 weeks...**

	All of the time	Most of the time	A good bit of the time	Some of the time	A little of the time	None of the time
23. Did you feel full of pep?	◯ 1	◯ 2	◯ 3	◯ 4	◯ 5	◯ 6
24. Have you been a very nervous person?	◯ 1	◯ 2	◯ 3	◯ 4	◯ 5	◯ 6
25. Have you felt so down in the dumps that nothing could cheer you up?	◯ 1	◯ 2	◯ 3	◯ 4	◯ 5	◯ 6
26. Have you felt calm and peaceful?	◯ 1	◯ 2	◯ 3	◯ 4	◯ 5	◯ 6
27. Did you have a lot of energy?	◯ 1	◯ 2	◯ 3	◯ 4	◯ 5	◯ 6
28. Have you felt downhearted and blue?	◯ 1	◯ 2	◯ 3	◯ 4	◯ 5	◯ 6
29. Did you feel worn out?	◯ 1	◯ 2	◯ 3	◯ 4	◯ 5	◯ 6
30. Have you been a happy person?	◯ 1	◯ 2	◯ 3	◯ 4	◯ 5	◯ 6
31. Did you feel tired?	◯ 1	◯ 2	◯ 3	◯ 4	◯ 5	◯ 6

32. During the **past 4 weeks**, how much of the time has **your physical health or emotional problems** interfered with your social activities (like visiting with friends, relatives, etc.)?

◯ 1 - All of the time

◯ 2 - Most of the time

◯ 3 - Some of the time

◯ 4 - A little of the time

◯ 5 - None of the time

21. How much **bodily** pain have you had during the **past 4 weeks**?

- 1 - None
- 2 - Very mild
- 3 - Mild
- 4 - Moderate
- 5 - Severe
- 6 - Very severe

22. During the **past 4 weeks**, how much did **pain** interfere with your normal work (including both work outside the home and housework)?

- 1 - Not at all
- 2 - A little bit
- 3 - Moderately
- 4 - Quite a bit
- 5 - Extremely

How TRUE or FALSE is each of the following statements for you.

	Definitely true	Mostly true	Don't know	Mostly false	Definitely false
33. I seem to get sick a little easier than other people	○ 1	○ 2	○ 3	○ 4	○ 5
34. I am as healthy as anybody I know	○ 1	○ 2	○ 3	○ 4	○ 5
35. I expect my health to get worse	○ 1	○ 2	○ 3	○ 4	○ 5
36. My health is excellent	○ 1	○ 2	○ 3	○ 4	○ 5

ABOUT

The RAND Corporation is a research organization that develops solutions to public policy challenges to help make communities throughout the world safer and more secure, healthier and more prosperous. RAND is nonprofit, nonpartisan, and committed to the public interest.

1776 Main Street
Santa Monica, California 90401-3208

RAND® is a registered trademark. Copyright © 1994-2016 RAND Corporation.

DocuSign Envelope ID: 4F4388D5-A568-4FAC-82D1-39CB3AB82D1B

18.2.8 Participant Blood Pressure and Heart Rate Diary and Participant Medication Diary

Date	Time	Systolic Blood pressure (mmHg)	Diastolic Blood Pressure (mmHg)	Heart rate (bpm)	Prednisolone (dose, mg)	Losartan (dose, mg)
W1-W6	AM					
	PM					
From W7-10, 3 times a week, or when feeling unwell	AM					

DocuSign Envelope ID: 4F4388D5-A568-4FAC-82D1-39CB3AB82D1B

18.2.9 IET-CVI CMR Protocol

Institute for Experimental and Translational
Cardio Vascular Imaging
University Hospital Frankfurt
Goethe CVI

IET-CVI CMR Imaging and Postprocessing Protocol

Executive Summary

Randomised placebo controlled clinical trial of efficacy of MYOcardial protection in patients with postacute inFLAMmatory cardiac involvEment due to COVID-19 (MYOFLAME-19)

CMR Protocol

Team

Core Lab Lead: [redacted]

Project Management: [redacted]

Study Trial Objectives and Design

To determine efficacy of a combined immunosuppressive and antiremodelling therapy in COVID-19 related postacute inflammatory cardiovascular involvement determined by CMR to reduce inflammatory myocardial injury compared to placebo

Rationale for Using CMR

Cardiovascular magnetic resonance imaging (CMR) constitutes advanced imaging technology providing in-depth, versatile, accurate and non-invasive means of cardiovascular phenotyping. CMR can inform on spectrum of cardiovascular pathophysiology, as well as dynamic evolution of changes by safe, serial examinations. CMR provides sensitive imaging on the inflammation-related changes in the heart muscle and vascular wall (inflammation, oedema, thickening, scarring), reduced ventricular and vascular deformation and stiffness, as well as vascular wall inflammation(1). T1 and T2 mapping provide means of quantifiable tissue characterization, which relate directly to the myocardial tissue disease activity and severity(2). Native T1 is sensitive for detection of abnormal myocardial remodelling processes, whereas T2 indicates the presence of oedema. Together, these two imaging markers help deciphering the predominant driver of signal change as either inflammatory (raised native T2) or fibrotic (normal native T2)(3). T1 and T2 mapping values correlate with histologic evidence of myocardial inflammation, severity of left ventricular (LV) remodelling and longitudinal strain, and activity of myocardial inflammation in patients with systemic inflammatory conditions. Late gadolinium enhancement is an established imaging technique for visualisation of regional myocardial injury, such as necrosis, oedema, or scar, and instrumental in recognising pericardial and vascular involvement(4–6). Myocardial perfusion imaging is an established clinical technique to evaluation myocardial blood flow. In this study, the imaging protocol will be based on **IET-CVI Imaging Protocol**, which relate to a highly standardised set of imaging parameters and operating procedures, which are locked, standardised, validated and evidence based. It will provide the basis for imaging tools deployable in the future routine clinical use.

DocuSign Envelope ID: 4F4388D5-A568-4FAC-82D1-39CB3AB82D1B

IET-CVI CMR Image Acquisition Protocol

Standardized CMR Imaging protocol with uniform imaging parameters will be installed on local scanners (1.5 and 3.0 Tesla) equipped with advanced cardiac software and a multichannel coil (Siemens Healthineers, Erlangen, Germany, Software Version VE11, CE marked). Scanner maintenance, calibration and quality assurance procedures were regularly provided by the vendor.

Imaging parameters, scanning, shimming procedures for all sequences were standardized and mandatorily performed by all operators in all scans. Comparability and reproducibility of measurements were determined at each location. All imaging parameters have been reported previously(7,8). Slice thickness in all acquisitions was set uniformly at 8 mm. All acquired images will be routinely examined for quality during data acquisition, especially the quality of the mapping images during the acquisition, for artefacts and their influence on the measurements. All staff conducting the image acquisition will undergo induction and training in all CMR scanning procedures with regular quality control assessments to enable the required level of standardization and image quality.

The image acquisitions will include:

1. Survey (3-dimensional localizer)
2. Cines (2-, 3- and 4-chamber long axis views, short-axis stack)
3. T1 and T2 mapping (3-short axis subset, 4 chamber long axis)
4. LGE (2-, 3- and 4-chamber long axis views, short-axis stack) in MAG and PSIR
5. LGE (candy-cane, 3-cross sectional axial slices) in MAG and PSIR
6. PWV inplane flow acquisition

DocuSign Envelope ID: 4F4388D5-A568-4FAC-82D1-39CB3AB82D1B

IET-CVI CMR Image Postprocessing Core Lab

1. The IET-CVI CMR Core Lab will serve as the core lab for this trial.
2. Standard Operating Procedures will apply
3. The analysis for this trial is blinded to clinical and other data from patients.
4. Image data and accompanying information will be kept pseudonymized
5. Reader: significant (>12 months) experience in the evaluation procedure.
6. CMR analyses will be performed according to standard operating procedures
7. Software and Data Format:
    a. Suiteheart (Neosoft SuiteHeart; Neosoft LPC, WI, US) will be used for CMR analyses. This software is CE marked, and the software respects all Core lab requirements for the CMR analysis.
    b. Evaluation contours and quantitative results will be transferred in the format as CSV data, respectively.

CMR: Diagnostic Targets and Parameters

1. LV/RV Volume
    - LVEDVI, LVESVI
    - RVEDVI, RVESVI
2. LV/RV Function
    - LVEF, RVEF
    - Strain: Global longitudinal strain (GLS), segmental GLS (segments 1-12)
    - LV-CI
3. LV Mass
    - LV mass index (g/m height), LV mass index (g/m^2 BSA)
4. LV Oedema
    - Septal myocardial T1
    - Septal myocardial T2
    - Segmental myocardial T1 (segments 1-12)
    - Segmental myocardial T2 (segments 1-12)
5. LV inflammatory injury
    - Presence of myocardial LGE [categorical value 0-none, 1-present]
    - Type of myocardial LGE [0- none, 1-ischaemic, 2-non-ischaemic]
    - Extent of myocardial LGE [% of LV Volume
6. Vascular inflammatory injury
    - Presence of ascending and descending aortic wall LGE [categorical value 0-none, 1-present]
    - Thickness of ascending and descending aortic wall LGE [mm]
    - Central aortic pulse wave velocity

Incidental Findings

Images will not be assessed for incidental findings. If the reader identifies a clinically significant abnormality beyond the scope of the trial, it will be reported to the Safety committee of the trial.

Data Transfer and Storage

The images will deidentified using web-based IET-CVI Core Lab anonymization tool. The software will anonymize the patient inside of the sender hospital and will send the de-identify patient to the IET-CVI Core Lab research PACS. The technical Director of the IET-CVI Core Lab will program the Anonymization tool by the requirements of the protocol of the local

DocuSign Envelope ID: 4F4388D5-A568-4FAC-82D1-39CB3AB82D1B

investigators.
Images will be transferred in DICOM format via a secured https internet transfer protocol. Every site will be provided with own login and password to. Images will be imported into the research PACS IET-CVI for Analysis. A standardized report will be issues for all acquisitions within this study.

Frankfurt, June 6th 2022

[redacted]

References

1. Puntmann VO., Valbuena S., Hinojar R., et al. Society for Cardiovascular Magnetic Resonance (SCMR) expert consensus for CMR imaging endpoints in clinical research: part I - analytical validation and clinical qualification. J Cardiov Magn Reson 2018;20(1):67. Doi: 10.1186/s12968-018-0484-5.

2. Puntmann VO., Zeiher AM., Nagel E. T1 and T2 mapping in myocarditis: seeing beyond the horizon of Lake Louise criteria and histopathology. Expert Rev Cardiovasc Ther 2018;16(5):319–30. Doi: 10.1080/14779072.2018.1455499.

3. Hinojar R., Foote L., Ucar EA., et al. Native T1 in Discrimination of Acute and Convalescent Stages in Patients With Clinical Diagnosis of Myocarditis A Proposed Diagnostic Algorithm Using CMR. Jacc Cardiovasc Imaging 2015;8(1):37–46. Doi: 10.1016/j.jcmg.2014.07.016.

4. Winau L., Baydes RH., Braner A., et al. High-sensitive troponin is associated with subclinical imaging biosignature of inflammatory cardiovascular involvement in systemic lupus erythematosus. Ann Rheum Dis 2018;77(11):1590. Doi: 10.1136/annrheumdis-2018-213661.

5. Varma N., Hinojar R., D'Cruz D., et al. Coronary Vessel Wall Contrast Enhancement Imaging as a Potential Direct Marker of Coronary Involvement Integration of Findings From CAD and SLE Patients. Jacc Cardiovasc Imaging 2014;7(8):762–70. Doi: 10.1016/j.jcmg.2014.03.012.

6. Puntmann VO., Nagel E., Hughes AD., et al. Gender-Specific Differences in Myocardial Deformation and Aortic Stiffness at Rest and Dobutamine Stress. Hypertension 2012;59(3):712–8. Doi: 10.1161/hypertensionaha.111.183335.

7. Puntmann VO., Carerj ML., Wieters I., et al. Outcomes of Cardiovascular Magnetic Resonance Imaging in Patients Recently Recovered From Coronavirus Disease 2019 (COVID-19). Jama Cardiol 2020;5(11):1265–73. Doi: 10.1001/jamacardio.2020.3557.

8. Leuw P de., Arendt CT., Haberl AE., et al. Myocardial Fibrosis and Inflammation by CMR Predict Cardiovascular Outcome in People Living With HIV. Jacc Cardiovasc Imaging 2021;14(8):1548–57. Doi: 10.1016/j.jcmg.2021.01.042.

DocuSign Envelope ID: 4F4388D5-A568-4FAC-82D1-39CB3AB82D1B

18.2.10 CE Certificate SuiteHeart Neosoft

bsi.

By Royal Charter

EC Certificate - Full Quality Assurance System

Directive 93/42/EEC on Medical Devices, Annex II excluding Section 4

**No.**	**CE 618294**
Issued To:	**Neosoft, LLC**<br>**N27W23910A Paul Road**<br>**Pewaukee**<br>**Wisconsin**<br>**53072**<br>**USA**

In respect of:

Design and manufacture of medical software applications used in imaging and post processing of MR images for analysing cardiac function.

on the basis of our examination of the quality assurance system under the requirements of Council Directive 93/42/EEC, Annex II excluding section 4. The quality assurance system meets the requirements of the directive. For the placing on the market of class III products an Annex II section 4 certificate is required.

For and on behalf of BSI, a Notified Body for the above Directive (Notified Body Number 2797):

[redacted]

[redacted] Senior Vice President Medical Devices

First Issued: **2015-08-07** Date: **2020-03-09** Expiry Date: **2024-05-26**

...making excellence a habit.™

Page 1 of 1

Validity of this certificate is conditional on the quality system being maintained to the requirements of the Directive as demonstrated through the required surveillance activities of the Notified Body. This approval excludes all products designed and/or manufactured by a third party on behalf of the company named on this certificate, unless specifically agreed with BSI.
This certificate was issued electronically and is bound by the conditions of the contract.

Information and Contact: BSI, Say Building, John M. Keynesplein 9, 1066 EP Amsterdam, The Netherlands Tel: + 31 20 346 0780
BSI Group The Netherlands B.V. registered in The Netherlands under 33264284.
A member of BSI Group of Companies.

DocuSign Envelope ID: 4F4388D5-A568-4FAC-82D1-39CB3AB82D1B

18.2.11 CE Certificate Omron

OMRON

No: OHQ(CS)-DoC(MDR)-5674624

EC Declaration of Conformity

Manufacturer:	OMRON HEALTHCARE Co., Ltd.
Single Registration Number:	JP-MF-000007213
Address:	53, Kunotsubo, Terado-cho, Muko, KYOTO, 617-0002 JAPAN
European Authorised Representative:	OMRON HEALTHCARE EUROPE B.V.
Address:	Scorpius 33, 2132 LR Hoofddorp, The Netherlands
Product Category:	Electronic Sphygmomanometers/Blood Pressure Monitors
Model (code):	X2 Smart (HEM-7143T2-ESL)
Basic UDI-DI:	4015672113054W
MDR Classification:	Class IIa (MDR Annex VIII Rule 10)

We herewith declare, under our sole responsibility, that the above mentioned product meets the provisions of the following European Union Regulations, Council Directives and Standards. All supporting documentation is retained at the premises of the manufacturer and the European Authorized Representative.
This Declaration of Conformity is valid in connection with all the shipping inspection reports for the respective batch of produced devices.

General applicable regulations:	Medical Device Regulation (EU) 2017/745	
Standards:	EN 1041:2008+A1:2013	EN ISO 10993-1:2009/AC:2010
	EN 1060-1:1995+A2:2009	EN ISO 10993-5:2009
	EN 1060-3:1997+A2:2009	EN ISO 10993-10:2013
	EN 60601-1:2006+A1:2013	EN ISO 13485:2016
	EN 60601-1-2:2015	EN ISO 14971:2012
	EN 60601-1-6:2010+A1:2015	EN ISO 15223-1:2016
	EN 60601-1-11:2015	EN ISO 81060-2:2019+A1:2020
	EN 62304:2006+A1:2015	
	EN 62366-1:2015	
	EN IEC 80601-2-30:2019	
Notified Body:	TÜV Rheinland LGA Products GmbH	
Address:	Tillystrasse 2, 90431 Nuremberg, Germany	
ID No:	Notified under number 0197 to the EC Commission	
Certificate Registration No:	Annex IX : HZ 2102042-1	

General applicable directives:	Radio Equipment Directive 2014/53/EU	
Standards:	EN 300 328 V2.2.2	EN 301 489-1 V2.2.3
	EN 301 489-17 V3.2.4	EN 62479:2010
	EN IEC 62368-1:2020+A11:2020	

General applicable directives:	RoHS Directive 2011/65/EU, (EU)2015/863 and (EU)2017/2102
Product Category for RoHS:	Category 8 (Medical devices)
Standards:	EN IEC 63000:2018

Place / Date: Kyoto / September 13, 2021

Signature: [redacted]

Name: [redacted]

Position: General Manager
Regulatory Affairs Department

All for Healthcare

OMRON HEALTHCARE Co., Ltd.
53, Kunotsubo, Terado-cho, Muko, KYOTO, 617-0002 JAPAN

DocuSign Envelope ID: 4F4388D5-A568-4FAC-82D1-39CB3AB82D1B

18.2.12 Members of Steering and Data Safety Monitoring Committees

Steering Committee			
Member	Post	Profession	Email/Phone
[redacted] (Chair)	University of Glasgow [redacted] 126 University Place Glasgow G12 8TA	[redacted]	Email: [redacted] Phone: [redacted]
[redacted]	[redacted] University of London	[redacted]	Email: [redacted]
[redacted]	[redacted] DeBakey VA Medical Center, Houston TX	[redacted]	Email: [redacted]
[redacted]	[redacted] Universitätsklinikum Frankfurt [redacted] Theodor-Stern-Kai 7 60590 Frankfurt	[redacted]	Email: [redacted]
[redacted]	Infektologikum Frankfurt Stresemannallee 3, 60596 Frankfurt am Main	[redacted]	Email: [redacted]
[redacted]	[redacted] University Hospital Frankfurt Theodor-Stern Kai 7 60590 Frankfurt-am-Main Germany	[redacted]	Email: [redacted]
[redacted]	[redacted] University Hospital Frankfurt Theodor-Stern Kai 7	[redacted]	Email: [redacted]

DocuSign Envelope ID: 4F4388D5-A568-4FAC-82D1-39CB3AB82D1B

	60590 Frankfurt-am-Main<br>Germany		
[redacted]	Medical University of Vienna,<br>[redacted]<br>Waehringer Guertel 18-20<br>1090 Vienna, Austria	[redacted]	[redacted]
[redacted]	[redacted]<br>Medizinische Universität Wien<br>Waehringer Guertel 18-20<br>1090 Vienna, Austria	[redacted]	Email:<br>[redacted]
**Data Safety Monitoring Comittee**			
[redacted] (Chair)	[redacted]<br>University of Oxford<br>Windmill Road<br>Headington, Oxford, OX3 7LD	[redacted]	Email:<br>[redacted]
[redacted]	[redacted] Yale School of Medicine; [redacted] 800 Howard Avenue,<br>New Haven, CT 06519	[redacted]	Email: [redacted]
[redacted]	[redacted]<br>Klinikum und Fachbereich Medizin der Goethe-Universität<br>[redacted]<br>Theodor-Stern-Kai 7<br>60590 Frankfurt am Main	[redacted]	Email: [redacted]

18.2.13 Highly effective methods for contraception

Methods that can achieve a failure rate of less than 1% per year when used consistently and correctly are considered as highly effective birth control methods.
Such methods include:

- combined (estrogen and progestogen containing) hormonal contraception associated with inhibition of ovulation.
    - oral
    - intravaginal
    - transdermal
- progestogen-only hormonal contraception associated with inhibition of ovulation:
    - oral
    - injectable
    - implantable
- intrauterine device (IUD)
- intrauterine hormone-releasing system
- bilateral tubal occlusion
- vasectomised partner (Vasectomised partner is a highly effective birth control method provided that partner is the sole sexual partner of the woman of childbearing potential trial participant and that the vasectomised partner has received medical assessment of the surgical success)
- sexual abstinence (sexual abstinence is considered a highly effective method only if defined as refraining from heterosexual intercourse during the entire period of risk associated with the study treatments)

DocuSign

Abschlusszertifikat

Umschlag-ID: 4F4388D5A5684FAC82D139CB3AB82D1B
Status: Abgeschlossen

Betreff: Mit Docusign abschließen: Myoflame19_Version 3.1 Protocol_20240529_clean.pdf

Quellumschlag:

Dokumentenseiten: 85
Signaturen: 6
Umschlagersteller:

Zertifikatsseiten: 5
Initialen: 0
[redacted]

Signatur mit Anleitung: Aktiviert
Winchsterstrasse 3

Umschlag-ID-Stempel: Aktiviert
Gießen, Hessen 35394

Zeitzone: (UTC+01:00) Amsterdam, Berlin, Bern, Rom, Stockholm, Wien
[redacted]

IP-Adresse: [redacted]

Eintragsverfolgung

Status: Original
25.06.2024 16:14:11

Inhaber: [redacted]

Standort: DocuSign

Unterzeichnerereignisse	Signatur	Zeitstempel
[redacted]<br>Pharmacovigilance<br>Alcedis GmbH<br>Sicherheitsstufe: E-Mail, Kontoauthentifizierung (keine)	DocuSigned by: [redacted]<br>Signaturübernahme: Hochgeladenes Signaturbild<br>Mit IP-Adresse: [redacted]	Gesendet: 25.06.2024 16:20:27<br>Eingesehen: 25.06.2024 20:19:09<br>Signiert: 25.06.2024 20:19:28

Vereinbarung bezüglich elektronischer Unterlagen und Signaturen:
Nicht über DocuSign angeboten

[redacted]<br>Biometry<br>Alcedis GmbH<br>Sicherheitsstufe: E-Mail, Kontoauthentifizierung (keine)	DocuSigned by: [redacted]<br>Signaturübernahme: Hochgeladenes Signaturbild<br>Mit IP-Adresse: [redacted]	Gesendet: 25.06.2024 16:20:27<br>Eingesehen: 25.06.2024 16:36:09<br>Signiert: 25.06.2024 16:36:24

Vereinbarung bezüglich elektronischer Unterlagen und Signaturen:
Nicht über DocuSign angeboten

[redacted]<br>Sicherheitsstufe: E-Mail, Kontoauthentifizierung (keine)	DocuSigned by: [redacted]<br>Signaturübernahme: Hochgeladenes Signaturbild<br>Mit IP-Adresse: [redacted]	Gesendet: 25.06.2024 16:20:28<br>Eingesehen: 25.06.2024 16:22:19<br>Signiert: 25.06.2024 16:22:31

Vereinbarung bezüglich elektronischer Unterlagen und Signaturen:
Akzeptiert: 06.11.2023 13:51:24
ID: f06e3449-abad-405c-84cc-67c29b38baf2

[redacted]<br>Sicherheitsstufe: E-Mail, Kontoauthentifizierung (keine)	DocuSigned by: [redacted]<br>Signaturübernahme: Vorgegebener Stil<br>Mit IP-Adresse: [redacted]	Gesendet: 25.06.2024 16:20:28<br>Eingesehen: 25.06.2024 19:18:16<br>Signiert: 25.06.2024 19:18:27

Vereinbarung bezüglich elektronischer Unterlagen und Signaturen:
Akzeptiert: 25.06.2024 19:18:16
ID: 5f68f54a-f5ad-4cae-9895-5e4aa40d9e5a

Vor-Ort-Unterzeichner – Ereignisse	Signatur	Zeitstempel

Bearbeiterversandereignisse	Status	Zeitstempel

Beauftragtenzustellereignisse	Status	Zeitstempel

Vermittlerversandereignisse	Status	Zeitstempel

Zertifizierter Versand - Ereignisse	Status	Zeitstempel

Kopienereignisse	Status	Zeitstempel
[redacted]<br>Clinical Operations<br>Alcedis GmbH<br>Sicherheitsstufe: E-Mail, Kontoauthentifizierung (keine)	**Kopiert**	Gesendet: 25.06.2024 16:20:29

Vereinbarung bezüglich elektronischer Unterlagen und Signaturen:
Nicht über DocuSign angeboten

Zeugen-Ereignisse	Signatur	Zeitstempel

Notarereignisse	Signatur	Zeitstempel

Umschlagereignisse – Überblick	Status	Zeitstempel
Umschlag gesendet	Hash-codiert/verschlüsselt	25.06.2024 16:20:29
Zertifiziert zugestellt	Sicherheitsprüfung ausgeführt	25.06.2024 19:18:16
Signiervorgang abgeschlossen	Sicherheitsprüfung ausgeführt	25.06.2024 19:18:27
Abgeschlossen	Sicherheitsprüfung ausgeführt	25.06.2024 20:19:28

Zahlungen	Status	Zeitstempel

Vereinbarung bezüglich elektronischer Unterlagen und Signaturen

ELECTRONIC RECORD AND SIGNATURE DISCLOSURE

From time to time, Alcedis GmbH (we, us or Company) may be required by law to provide to you certain written notices or disclosures. Described below are the terms and conditions for providing to you such notices and disclosures electronically through the DocuSign system. Please read the information below carefully and thoroughly, and if you can access this information electronically to your satisfaction and agree to this Electronic Record and Signature Disclosure (ERSD), please confirm your agreement by selecting the check-box next to ‘I agree to use electronic records and signatures’ before clicking ‘CONTINUE’ within the DocuSign system.

Getting paper copies

At any time, you may request from us a paper copy of any record provided or made available electronically to you by us. You will have the ability to download and print documents we send to you through the DocuSign system during and immediately after the signing session and, if you elect to create a DocuSign account, you may access the documents for a limited period of time (usually 30 days) after such documents are first sent to you. After such time, if you wish for us to send you paper copies of any such documents from our office to you, you will be charged a $0.00 per-page fee. You may request delivery of such paper copies from us by following the procedure described below.

Withdrawing your consent

If you decide to receive notices and disclosures from us electronically, you may at any time change your mind and tell us that thereafter you want to receive required notices and disclosures only in paper format. How you must inform us of your decision to receive future notices and disclosure in paper format and withdraw your consent to receive notices and disclosures electronically is described below.

Consequences of changing your mind

If you elect to receive required notices and disclosures only in paper format, it will slow the speed at which we can complete certain steps in transactions with you and delivering services to you because we will need first to send the required notices or disclosures to you in paper format, and then wait until we receive back from you your acknowledgment of your receipt of such paper notices or disclosures. Further, you will no longer be able to use the DocuSign system to receive required notices and consents electronically from us or to sign electronically documents from us.

All notices and disclosures will be sent to you electronically

Unless you tell us otherwise in accordance with the procedures described herein, we will provide electronically to you through the DocuSign system all required notices, disclosures, authorizations, acknowledgements, and other documents that are required to be provided or made available to you during the course of our relationship with you. To reduce the chance of you inadvertently not receiving any notice or disclosure, we prefer to provide all of the required notices and disclosures to you by the same method and to the same address that you have given us. Thus, you can receive all the disclosures and notices electronically or in paper format through the paper mail delivery system. If you do not agree with this process, please let us know as described below. Please also see the paragraph immediately above that describes the consequences of your electing not to receive delivery of the notices and disclosures electronically from us.

How to contact Alcedis GmbH:

You may contact us to let us know of your changes as to how we may contact you electronically, to request paper copies of certain information from us, and to withdraw your prior consent to receive notices and disclosures electronically as follows:
To contact us by email send messages to: [redacted]

To advise Alcedis GmbH of your new email address

To let us know of a change in your email address where we should send notices and disclosures electronically to you, you must send an email message to us at [redacted] and in the body of such request you must state: your previous email address, your new email address. We do not require any other information from you to change your email address.

If you created a DocuSign account, you may update it with your new email address through your account preferences.

To request paper copies from Alcedis GmbH

To request delivery from us of paper copies of the notices and disclosures previously provided by us to you electronically, you must send us an email to [redacted] and in the body of such request you must state your email address, full name, mailing address, and telephone number. We will bill you for any fees at that time, if any.

To withdraw your consent with Alcedis GmbH

To inform us that you no longer wish to receive future notices and disclosures in electronic format you may:

i. decline to sign a document from within your signing session, and on the subsequent page, select the check-box indicating you wish to withdraw your consent, or you may;

ii. send us an email to [redacted] and in the body of such request you must state your email, full name, mailing address, and telephone number. We do not need any other information from you to withdraw consent.. The consequences of your withdrawing consent for online documents will be that transactions may take a longer time to process..

Required hardware and software

The minimum system requirements for using the DocuSign system may change over time. The current system requirements are found here: https://support.docusign.com/guides/signer-guide-signing-system-requirements.

Acknowledging your access and consent to receive and sign documents electronically

To confirm to us that you can access this information electronically, which will be similar to other electronic notices and disclosures that we will provide to you, please confirm that you have read this ERSD, and (i) that you are able to print on paper or electronically save this ERSD for your future reference and access; or (ii) that you are able to email this ERSD to an email address where you will be able to print on paper or save it for your future reference and access. Further, if you consent to receiving notices and disclosures exclusively in electronic format as described herein, then select the check-box next to 'I agree to use electronic records and signatures' before clicking 'CONTINUE' within the DocuSign system.

By selecting the check-box next to 'I agree to use electronic records and signatures', you confirm that:

- You can access and read this Electronic Record and Signature Disclosure; and
- You can print on paper this Electronic Record and Signature Disclosure, or save or send this Electronic Record and Disclosure to a location where you can print it, for future reference and access; and
- Until or unless you notify Alcedis GmbH as described above, you consent to receive exclusively through electronic means all notices, disclosures, authorizations, acknowledgements, and other documents that are required to be provided or made available to you by Alcedis GmbH during the course of your relationship with Alcedis GmbH.

Supplementary Note 2. Statistical Analysis Plan

The statistical analysis plan (Version 1.0, dated 11 June 2025) is reproduced on the following pages.

Docusign Envelope ID: 4EE6D746-0E71-4D08-A616-DB8FFDA8C1D5

a HUMA company

Statistical Analysis Plan

MYOFLAME-19

Randomised placebo controlled clinical trial of efficacy of MYOcardial protection with postacute inFLAMmatory cardiac involvEment due to COVID-19

**Project No:**	7021000_MYOFLAME-19
**Study Code:**	MYOFLAME-19
**Medication:**	Verum (Prednisolone + Losartan), Placebo (Placebo 1 + 2)
**Dokument ID:**	MYOFLAME_SAP_Final_v1.0_2025JUN11.docx
**Version:**	Version 1.0
**Date:**	11.06.2025
**Sponsor:**	Goethe University Frankfurt<br>represented by the President<br>Theodor-Adorno-Platz 6<br>D-60323 Frankfurt/Main, Germany
**Author:**	Alcedis GmbH<br>Winchesterstraße 3<br>35394 Gießen, Germany<br>[redacted]
**Protokoll (Version, Datum):**	Version 3.1, 29MAY2024

Docusign Envelope ID: 4EE6D746-0E71-4D08-A616-DB8FFDA8C1D5

MYOFLAME-19

Randomised placebo controlled clinical trial of efficacy of MYOcardial protection with postacute inFLAMmatory cardiac involvEment due to COVID-19

We, the undersigned, have read the Statistical Analysis Plan and agree that it contains all information required for statistical analysis of the data collected in the above-named study.

Date, Signature

Sponsor 12.06.2025 | 09:09 CEST [redacted]

Date [redacted], Goethe University Frankfurt (Principal Investigator, Representative of the Sponsor)

Author 12.06.2025 | 08:18 MESZ [redacted]

Date [redacted]

Docusign Envelope ID: 4EE6D746-0E71-4D08-A616-DB8FFDA8C1D5

Table of contents

1 List of Abbreviations 4
2 Introduction 5
2.1 Background 5
2.2 Study design 6
3 Analysis data and patient populations 7
3.1 Study objective 7
3.2 Analysis populations 8
3.3 Patients withdrawal(s) 8
3.4 Endpoints 9
4 Methods of analysis 10
4.1 Hypotheses 10
4.2 Statistical methods 11
4.3 Statistical analysis 12
4.4 Methods for handling missing data 12
4.5 Methods for handling inconsistent data 13
4.6 Methods for handling outliers 13
4.7 Methods for point and interval estimates 13
4.8 Validation of statistical methods 13
4.9 Methods for handling multicenter trial data 13
4.10 Treatment interactions 13
4.11 Methods for handling repeated measurements 13
4.12 Calculation of derived variables 13
4.13 Use of baseline values 17
4.14 Use of covariates 18
4.15 Identification of fixed and random factors 18
4.16 Subset analyses 18
4.17 Interim and follow-up analyses 19
4.18 Study stopping rules 19
4.19 Statistical significance levels 19
4.20 Methods for handling dropouts and protocol violators 19
4.21 Methods for handling more than two treatment groups 19
4.22 Methods for handling concomitant medications 19
4.23 Handling of therapy changes and unscheduled visits 20
4.24 Changes to the planned analyses 20
5 Mock-ups for tables, listings and figures 20
6 Statistical software 20
7 Medical dictionaries 20
8 Coding conventions 20
9 Output format 20
10 List of the tables, lists, and figures planned for the Final Statistical Report 20
11 History table 21

Docusign Envelope ID: 4EE6D746-0E71-4D08-A616-DB8FFDA8C1D5

1 List of Abbreviations

Abbreviation	Definition
AE	Adverse event
AoAsc	Aorta ascenders
AoDesc	Aorta descenders
AT	Anaerobic threshold
BL	Baseline
BP	Blood Pressure
BSA	Body Surface Area
CMR	Cardiovascular magnetic resonance imaging
CVD	Cardiovascular disease
CVS	Cardiovascular syndrome
EFS	Event-free survival
FAS	Full Analysis Set
FSH	Follicle stimulating hormone
FU	Follow-Up
GLS	Global longitudinal strain
HDL	High Density Lipoprotein
HF	Heart Failure
IMP	Investigational medicinal product
ITT	Intention to Treat Set
LDL	Low Density Lipoprotein
LGE	Late gadolinium enhancement
LipoA	Lipoprotein A
LLOQ	Lower limit of quantitation
LOQ	Limit of quantitation
LV	Left ventricular
LVEDV	Left ventricular end-diastolic volume
LVEDVI	Left ventricular end-diastolic volume index
LVEF	Left ventricular ejection fraction
LVESV	Left ventricular end-systolic volume
LVESVI	Left ventricular end-systolic volume index
LVM	Left ventricular mass
LVMI	Left ventricular mass index
MACE	Major adverse cardiovascular events
OS	Overall survival
PASC	Postacute sequale of COVID1-9 infection
POTS	Postural orthostatic tachycardia syndrome
PP	Per Protocol Population

Docusign Envelope ID: 4EE6D746-0E71-4D08-A616-DB8FFDA8C1D5

PWV	Pulse wave velocity
QoL	Quality of Life
RER	Respiratory exchange ratio
RV	Right ventricular
RVEDV	Right ventricular end-diastolic volume
RVEDVI	Right ventricular end-diastolic volume index
RVEF	Right ventricular ejection fraction
RVESV	Right ventricular end-systolic volume
RVESVI	Right ventricular end-systolic volume index
SAE	Serious adverse event
SAF	Safety Analysis Set
ULOQ	Upper limit of quantitation
VLDL	Very low density lipoprotein
WOCBP	Women of childbearing potential

2 Introduction

2.1 Background

Postacute sequelae of COVID-19 infection (PASC) are increasingly recognised complications and are defined by lingering symptoms, not present prior to the infection, typically persisting for more than 4 weeks. Cardiac symptoms due to postacute inflammatory cardiac involvement affect a broad segment of people, who were previously well and may have had only mild acute illness (PASC-cardiovascular syndrome, PASC-CVS). Symptoms may be contiguous with the acute illness, however, more commonly they occur after a delay. Symptoms related to the cardiovascular system include exertional dyspnoea, exercise intolerance chest tightness, pulling or burning chest pain, and palpitations. Phenotypically, it is characterised by chronic perivascular and myopericardial inflammation. Cardiac symptoms may be accompanied by manifestations of other organ systems, including fatigue, brain fog, myalgias, skin and joint manifestations, etc, now commonly referred to as the Long COVID or PASC syndrome.

Evidence suggests inflammatory autoimmune mechanisms, which develop de novo in response to the infection. In PASC-CVS, early subtle inflammatory heart changes with mild functional impairment are often undetectable by routine diagnostic tests, nor accompanied by significant rise in troponin. Studies using CMR imaging have identified changes consistent with non-ischaemic cardiovascular inflammatory involvement, including increased myocardial mapping values and perimyocardial late gadolinium enhancement. Participants may also have subnormal LVEF, however, structural heart disease by profoundly reduced LVEF or dilated heart cavities, or necrotic or thromboembolic complications are not typical findings. Likewise, areas of substantial necrosis typically found in classical viral myocarditis, are rare. These

Docusign Envelope ID: 4EE6D746-0E71-4D08-A616-DB8FFDA8C1D5

abnormalities can be detected as early as 2 weeks after the infection and can be observed several months after the infection.

Early intervention with immunosuppression and antiremodelling therapy may reduce symptoms and myocardial impairment, by minimising the disease activity and inducing disease remission. Low dose maintenance therapy may help to maintain the disease activity at the lowest possible level. Clinical trials of immunosuppression in participants with viral myocarditis in advanced stages of heart failure have not shown an improved outcome, however there was an improvement of LVEF with antiremodelling therapy in participants with reduced function. The benefits of early initiations of antiremodelling therapy to reduce symptoms of exercise intolerance are well recognised, but not commonly employed outside the contexts of heart failure or hypertension. As most participants with inflammatory heart disease only have mild and nonspecific symptoms and few or no structural abnormalities, they are left untreated (standard of care). The aim of this study is to examine the efficacy of a combined immunosuppressive/antiremodelling therapy in participants with PASC symptoms and inflammatory cardiac involvement determined by CMR, to reduce the symptoms and inflammatory myocardial injury and thereby stop the progression to reduced LVEF, HF and death.

For more details see protocol.

2.2 Study design

This is a prospective multicentre, randomised, double-blind, placebo controlled clinical trial. The efficacy of immunosuppressive and antiremodelling therapy will be compared to placebo in participants with COVID-19-related cardiac involvement determined by CMR imaging.

Participants with laboratory evidence of recent COVID-19 infection (>4 weeks, defined as > 28 days from the date of the clinical diagnosis) and cardiac symptoms, not present prior to infection, will be screened for eligibility. Participants will have to provide a confirmatory evidence of severe acute respiratory syndrome coronavirus 2 (SARS-CoV2) infection by detection of ribonucleic acid in swab test of upper respiratory tract or antigen test using an approved method. Given the discontinuation of systematic public testing, home antigen tests, sickness note, or evidence of antibody response, a doctor’s sickness notice for the COVID illness can also serve as a proof to a prior infection. Chest pain, dyspnoea, palpitations, syncope are considered cardiac symptoms. Written informed consent will be obtained from all participants prior to undergoing baseline assessments (CMR, clinical assessments with standardised questionaries for symptoms, ECG, CPET, blood tests for laboratory assessments and emerging biomarker analyses). Biobanking blood samples will be stored at -80°C until usage. CPET will also be performed, subject to local availability.

Docusign Envelope ID: 4EE6D746-0E71-4D08-A616-DB8FFDA8C1D5

Participants fulfilling inclusion and exclusion criteria will be randomised equally (1:1) into the combined verum and combined placebo arm. A total 280 participants (140 per treatment group) will be randomised.

In the Verum arm:

Losartan + Prednisolone

In the Placebo arm:

Placebo 1 + Placebo 2 (corresponds to the standard-of-care)

The following Flowchart shows the study design:

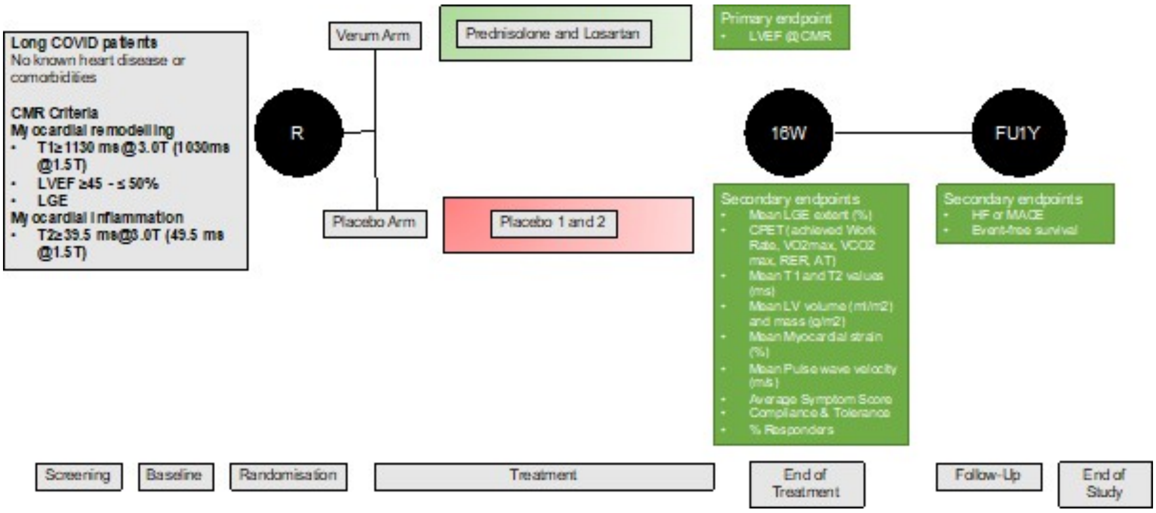

3 Analysis data and patient populations

In the following document the planned statistical analyses of the study will be outlined, i.e. the statistical methods will be described in detail and the tables and figures of the statistical report will be defined.

3.1 Study objective

The primary objective is to determine the efficacy of a combined immunosuppressive and antiremodelling therapy in COVID-19 related inflammatory cardiovascular involvement by CMR to reduce inflammatory myocardial injury compared to placebo.

The secondary objectives are to determine the efficacy of a combined immunosuppressive and antiremodelling therapy in COVID-19 related inflammatory cardiovascular involvement by CMR to reduce inflammatory myocardial injury compared to placebo by improvement in other clinical parameters.

The primary safety objective is to demonstrate that a combined immunosuppressive and antiremodelling therapy in proposed doses in this participant population is safe.

Docusign Envelope ID: 4EE6D746-0E71-4D08-A616-DB8FFDA8C1D5

3.2 Analysis populations

Participants with long COVID19 with evidence of cardiac involvement by CMR criteria and no known previous cardiovascular disease, who fulfil the selection criteria. Participants will be randomized into the verum and placebo groups.

Full Analysis Set (FAS): The full analysis set includes all participants who were randomized.

Intention to Treat Set (ITT): The intention to treat set is defined as the subgroup of participants of the FAS who had two assessments with CMR (BL and follow-up) and who received at least 4 weeks of treatment* according to their treatment arm after study inclusion.

Per Protocol Population (PP): The per protocol population is defined to include all participants who have completed BL examinations and another CMR at 16 weeks and have complied 75% with the study drug regimen**. Specific reasons for warranting exclusion will be documented prior to database closure. Not all protocol deviators and violators will be excluded from the per protocol population.

Safety Analysis Set (SAF): The safety analysis set is defined as all participants who received at least one dose of IMP treatment (verum or placebo) during the study.

The baseline analysis will be performed on the FAS and ITT population. All primary and secondary efficacy analyses will be performed for the ITT and for the PP population to account for protocol violations unless stated otherwise. All safety analyses will be performed for the SAF.

All protocol deviations, drop-outs of the study and participants excluded from analysis will be described by summary statistics and presented in a CONSORT. Where possible, they will be categorized by reason. Drop-outs include withdrawals, screening failures and participants who are lost-to-follow-up.

---

* 4 week of treatment will be checked in the eCRF form “Visit at Week 6” in the section “Study Medication”. If there is a medication dose documented, it can be assumed that the patient was treated for 4 weeks. If no dose is given, use the patient dairy (if available) and the “End of treatment” section in the eCRF form “Drug Accountability at End of the Treatment”.

** The patient diaries are partly incomplete. Therefore complied 75% with the study drug is defined as documented treatment at Visit at Week 2, 6 and 12. If in each visit a dose is documented, the patient is considered as 75% compliant. In case of missing entries also consider the patient diaries. If there are still unclear patients, please discuss with sponsor (individual case solution).

3.3 Patients withdrawal(s)

Participants may voluntarily withdraw their consent to study participation at any time without giving any reason. The investigator may also, at their discretion, withdraw the participant from participating in this study at any time, or the sponsor may discontinue the study.

Docusign Envelope ID: 4EE6D746-0E71-4D08-A616-DB8FFDA8C1D5

Reasons for early withdrawal from the study should be documented in the eCRF as:

- Study closed / terminated
- Participant died or is lost to follow-up
- Investigator's decision
- Participant withdrew consent to trial participation

For participants who withdraw consent to trial participation, participation ends immediately. Date of withdrawal from the study, with reason for withdrawal (if applicable), will be documented in the participant's medical record and recorded on the eCRF. In the case of death, a death certificate should be obtained, if possible, with the cause of death evaluated and documented.

3.4 Endpoints

Primary endpoint

The primary endpoint of this study is absolute LVEF change to baseline at W16, measured by CMR, compared between the verum and placebo group.

Secondary endpoints

The secondary endpoints include the following continuous endpoints, where "changes" refer to the difference between baseline and W16 (BL, absolute and in %):

- Mean LGE extent (%) and change thereof compared to BL**
- CPET (achieved Work Rate, VO_2max, VCO_2max, RER, AT, slope) and change thereof compared to BL
- Mean T1 and T2 values (ms) and change thereof compared to BL
- Mean LV and RV volumes (ml/m^2) and LV mass (g/m^2) as well as derived parameters and change thereof compared to BL
- Mean Myocardial strain (%) and change thereof compared to BL
- Mean Pulse wave velocity (PWV) (m/s) and change thereof compared to BL**
- Aortic wall thickness (LGE, mm) and change thereof compared to BL
- Average Symptom Scores (Modified Canadian Chest pain scale, NYHA, MRC Dyspnoea scale, Long COVID Questionnaire) and QoL (RAND 36-Item Health Survey Version 2.0) and change thereof compared to BL
- Compliance: Frequency of prescribed medication consumed, participant diary and drug adherence, total cumulative steroid dose
- Tolerance: number of participants who required dose reduction or treatment cessation due to side effects, especially due to
  - Hypotension

Docusign Envelope ID: 4EE6D746-0E71-4D08-A616-DB8FFDA8C1D5

  - Unblinding due to emergency safety issues (see "Prüfarztordner, Formblatt" and ask Alcedis PL for further information)
- Number of Responders at week 16 by achieving:
  - partial response: a normal CMR result is defined as normal T1 and T2, normal gender-age predicted LVEF, non-dilated LV (for definition of normal CMR results see 4.12)
  - total response: in addition to the above absence of LGE (for definition see 4.12)
- Proportion of participants with HF or MACE after 1 years*
- 1-year Event-free survival*

*The 1-year FU analysis is NOT part of this SAP, the analysis will NOT be done by Alcedis.
**This endpoint will be analyzed later, because the data is not valid now. The analysis will NOT be done by Alcedis.

<u>Safety endpoints</u>

Safety parameters include the number or frequency, severity, and number of adverse events (AE), serious AE (SAEs), defined by:

- Proportion of participants with serious infectious complications (fever ≥38.5°C, accompanied by rise on hsCRP, neutrophilia, lymphocytosis, need for antiviral or antibiotic treatment)
- Proportion of participants with symptomatic hypotension (dizziness, blackouts, and systolic BP<90 mmHg) or bradycardia (heart rate <40/min)*
- Proportion of participants with a significant rise in cardiac biomarkers (hsTNT, NTproBNP, >3-times the BL)
- Proportion of participants with a significant drop in eGFR compared to BL (>25%) compared to BL
- Proportion of participants with developing hypertensive crisis

* AEs will be coded according to MedDRA, for "bradycardia" the LLT code 10006093, LLT term=Bradycardia, PT term=Bradycardia, SOC term=Cardiac disorders will be used to identify patients with bradycardia. "heart rate < 40/min" will not be considered.

4 Methods of analysis

4.1 Hypotheses

For primary analysis, the absolute LVEF change to baseline at W16, measured by CMR, will be compared between the verum and placebo group.

Docusign Envelope ID: 4EE6D746-0E71-4D08-A616-DB8FFDA8C1D5

The unpaired t-test will be applied. The null hypothesis H0: μVERUM = μPL vs. H1: μVERUM ≠ μPL (with μ representing the expectation value of the absolute change of LVEF from BL at W16) will be tested confirmatively at the 5% significance level.
Secondary endpoints will be compared exploratively between treatment arms or subgroups thereof at a 5% significance level in a 2-sided manner (hypotheses formulated analogously to the primary hypothesis). Further exploratory investigation may be employed.
In case of multiplicity the Bonferroni-Holm or Tukey adjustment will be applied to adjust p-values. Then adjusted and unadjusted p-values will be reported.

4.2 Statistical methods

All summary statistics will be presented for the corresponding analysis set and treatment groups and stratified by treatment and visit. Descriptive summary statistics will at least comprise the number of missing values, the absolute and relative number (N, %) of any ordinal or nominal parameter. Similarly, for metric parameters the number of missing values, the arithmetic mean, SD, median, Q1, Q3, minimum, maximum will be reported. Two-sided 95% confidence intervals of the expectation values of the population (estimated by the mean) and probabilities within the population (estimated by the observed frequencies) will be given for the treatment difference of the primary and secondary endpoints if the data is distributed accordingly. Continuous parameters will be tested for normal distribution by Shapiro-Wilk-Test. If necessary, adequate data transformation, such as a Box-Cox transformation (e.g. logarithmic), will be performed to achieve normality. Beside the primary endpoint, some parameters will be illustrated using adequate graphical illustrations such as boxplots, mean plots, bar charts or flow charts. If the number of observations becomes too small (< 10), either dot plots will be considered for continuous parameter or graphical illustration will be skipped.
All primary and secondary endpoints will also be analyzed in a descriptive manner using summary statistics stratified by treatment and visit. All named analyses will be performed on participant level. The differences in CMR parameters and other continuous secondary outcomes from baseline to 16 weeks post randomization between the two treatment groups will be analyzed using an ANCOVA. Treatment effect estimates and 95% confidence intervals will be determined. These hypotheses tests will be of explorative nature and tested at a global 5% significance level between treatment groups/factors in a 2-sided manner.
It was planned, that compliance will be assessed by the participant diary and drug accountability (e.g. empty Dossett boxes) for IMP intake. According to incomplete patient diaries, compliance will be defined according to the information in the visits. Summary statistics will be given for the treatment intake (IMP), i.e. consumed medication prescribed, stratified by treatment and visit. Relevant information of the participant diary will be summarized and reported descriptively.

Docusign Envelope ID: 4EE6D746-0E71-4D08-A616-DB8FFDA8C1D5

Tolerance will be assessed by the absolute and relative (%) frequency of participants changing their treatment and number of participants requiring dose reduction or treatment cessation due to side effects.

Safety data will be analyzed describing frequency, severity and types of adverse events for all treatment groups. The proportion of AEs (including AESI and special situations) related to the contrast agent will also be analysed. All adverse events will be coded and tabulated by system organ class and preferred term for individual events within each system organ class and will be presented by descending frequency. Adverse events will also be tabulated by severity and relationship to the study medication. Serious adverse events will be summarized separately. Listings will be produced, including actions taken and outcome.

A treatment comparison of the occurrence of frequent AEs (especially cardiac related; frequent AE means at least 10% of all patients had this AE, if no AEs are present in 10% of the patients show the 3 most often documented AEs) will be performed using a Chi Square Test (or exact Fisher Test in case of small abs. frequencies) with α=5%. Time-to-event analysis will be performed for frequent AEs of interest. Analysis methods will employ Kaplan-Maier curves, Log-rank tests and lifetable analysis.

Unless stated otherwise, time-to-event data will in general be reported using the Kaplan-Meier (KM; Product Limit) estimators (including 95% confidence intervals) or life tables (discrete timeline) and illustrated with the corresponding curve including the number of participants at risk. Participants who drop-out or who are excluded for safety reasons will in general be censored in efficacy analyses unless specified otherwise or implausible. Cumulative occurrence rates are reported similarly, and will be plotted, where occurrence rates are requested. If less than 5 values per variable are available, the data will be enumerated instead of being summarized.

4.3 Statistical analysis

First, the disposition will be given. Then the baseline data, e.g. socio-demographic data, medical history, previous and concomitant medication as well as previous and concomitant diseases will be shown.

Regarding the primary endpoint the absolute LVEF change will be displayed.

Then the secondary endpoints will be shown. First, parameters of CMR and CPET, followed by symptom scores and compliance and tolerance. Afterwards the safety analysis will be shown.

4.4 Methods for handling missing data

Missing data of a parameter will not be transformed and remain untouched. Summary statistics will generally be given including the underlying number of valid individual values and missing data.

Docusign Envelope ID: 4EE6D746-0E71-4D08-A616-DB8FFDA8C1D5

For the primary analysis, the unpaired t-test will be applied.

4.5 Methods for handling inconsistent data

Not applicable.

4.6 Methods for handling outliers

Outliers will not be excluded.

4.7 Methods for point and interval estimates

Not applicable.

4.8 Validation of statistical methods

Model fit of all statistical models will be determined. Besides the analysis of residuals, the (adjusted) coefficient of determination R^2 and the likelihood ratio test will be used to evaluate the general model fit of linear models. In case of logistic models, a Hosmer-Lemeshow test will be applied. Other statistical models will be handled accordingly.

Only deviations from the general overview will be noted in the subsequent sub-sections of the efficacy analysis for the corresponding parameter.

4.9 Methods for handling multicenter trial data

Participants from all study centers will be pooled and tabulated as a single treatment group.

4.10 Treatment interactions

No relevant interactions are known.

4.11 Methods for handling repeated measurements

Not applicable.

4.12 Calculation of derived variables

Age at informed consent [years]: Year of informed consent – Year of birth

Body Surface Area (BSA) [m^2]: The Body Surface Area will be calculated using the Mosteller formula. BSA [m^2] = (Height [cm] x Weight [kg]/3600)$^{1/2}$, use Weight and Height from the Baseline form and the Visit at Week 16 and calculate the following variables for the respective time points.

Left ventricular end-diastolic volume index [ml/m^2]: LVEDVI= LVEDV (left ventricular end-diastolic volume)/body surface area (for Baseline and Visit at Week 16)

Left ventricular end-systolic volume index [ml/m^2]: LVESVI = LVESV (left ventricular end-systolic volume)/body surface area (for Baseline and Visit at Week 16)

Docusign Envelope ID: 4EE6D746-0E71-4D08-A616-DB8FFDA8C1D5

Right ventricular end-diastolic volume index [ml/m^2]: RVEDVI=RVEDV (right ventricular end-diastolic volume)/body surface area (for Baseline and Visit at Week 16)

Right ventricular end-systolic volume index [ml/m^2]: RVESVI=RVESV (right ventricular end-systolic volume)/body surface area (for Baseline and Visit at Week 16)

LV mass index [g/m^2]: LVMI=LVM (left ventricular mass)/body surface area (for Baseline and Visit at Week 16)

Percentage change: (value at visit – value at baseline)/value at baseline * 100

RAND 36-item Health Survey, 36-Item Short Form Survey Instrument (SF-36)
The Short Form-36 is a generic measure of health-related quality of life and has been shown to discriminate between subjects with different chronic conditions and between subjects with different severity levels of the same disease. The SF-36 has also demonstrated sensitivity to significant treatment effects in a variety of participantpopulations. It consists of 36 items. For each dimension, item scores are coded, summed and transformed to a scale from 0 (worst health) to 100 (best health) according to German population norm 1994 scoring rules.

SF-36 recording items

Item numbers	Original response category
1 2 20 22 34 36	1
	2
	3
	4
	5
3 4 5 6 7 8 9 10 11 12	1
	2
	3
13 14 15 16 17 18 19	1
	2
21 23 26 27 30	1
	2
	3
	4
	5
	6
24 25 28 29 31	1
	2
	3
	4
	5
	6
32 33 35	1
	2
	3
	4

	5

SF-36 subscales

The items in the same scale will be averaged together to create the 8 subscale scores. Items with missing data will not be taken into account when calculating the scale scores. Hence, scale scores represent the average for all items in the scale that the patient answered. For each component score, if 50% or less of the component score is missing, the mean of the remaining component score will be imputed as the value for the missing component score. If more than 50% of the component score is missing for the item, the imputed value will be set to missing.

**Subscale**	**Number of items**	**Items to calculate average**
Physical functioning (PF)	10	3 4 5 6 7 8 9 10 11 12
Role limitations due to physical health (RP)	4	13 14 15 16
Role limitations due to emotional problems (RE)	3	17 18 19
Vitality (VT)	4	23 27 29 31
Emotional well-being (MH)	5	24 25 26 28 30
Social functioning (SF)	2	20 32
Bodily pain (BP)	2	21 22
General health (GH)	5	1 33 34 35 36

SF-36 summary component scores

In order to create the physical component score and the mental component score the Z-transformed scale values will be multiplied by the respective coefficients for the physical or mental factor and then added together.

- Physical Component Summary scale (PCS)
- Mental Component Summary scale (MCS)

If one Z-score is missing, the aggregate physical or mental summary scale will be set to missing.

Reference: Morfeld M, Kirchberger I, Bullinger M, SF-36: Fragebogen zum Gesundheitszustand, HOGREFE Verlag, 2. Version (2011).

75% Compliance: 75% Compliance with the study drug is defined as documented treatment at Week 2, 6 and 12. If in each visit a dose is documented, the patient is considered as 75% compliant. In case of missing entries also consider the patient diaries. If there are still unclear patients, please discuss with sponsor (individual case solution).

Start of therapy/ first dose of study treatment: Use first date from patient diary, regardless of whether both medications have been started or not.

Docusign Envelope ID: 4EE6D746-0E71-4D08-A616-DB8FFDA8C1D5

Frequency of prescribed medication consumed: Number of days on which both drugs (Losartan (or placebo 1) and Prednisolone (or placebo 2)) were taken divided by number of days on which both drugs should be taken. Days on which both drugs should be taken are days between start of therapy and end of therapy.

Total cumulative dose [mg]: The total cumulative dose is calculated as the sum of all doses taken.

Total amount of contrast agent Gadovist® [ml]: in case of missing data the total cumulative dose of Gadovist will be calculated as body weight at the time of the visit x 0.1; for example for a 70kg person, the dose given is 7 ml.

Normal CMR results:

- Normal T1: 1.5 tesla <1006 ms; 3.0 Tesla <1106 ms
- Normal T2: 1.5 Tesla: <47.4 ms; 3.0 Tesla <37.4 ms
- Normal gender-age predicted LVEF:

	< 35 years	>= 35 years
Female	57-81%	57-81 %
Male	57-77 %	59-83 %

- Non-dilated LV: The value LV-EDV index (ml/m2) will be used to check whether there is a non-dilated LV or not. LV-EDV will be divided by BSA (LVEDV Index) at week 16 and then compared to the values in the following table:

	< 35 years	>= 35 years
Female	62-98	51-95
Male	68-112	53-97

- Absence of LGE: Myocardial gadolinium enhancement
    - Has late myocardial gadolinium enhancement been detected? = no -> Absence
    - Has late myocardial gadolinium enhancement been detected? = yes - non ischaemic -> Presence (yes – ischaemic should not occur (violation of IC/EC), if it occurs in the data, please consult with the sponsor)

eGFR: The eGFR will be calculated with the **2021 CKD-EPI Creatinine** method.

eGFR [ml/min/1.73 m²] = $142 \times (Scr/A)^{B} \times 0.9938^{age} \times$ (1.012 if female), where A and B are the following:

**Female**		**Male**	
**Scr ≤ 0.7**	A = 0.7<br>B = -0.241	**Scr ≤ 0.9**	A = 0.9<br>B = -0.302

Docusign Envelope ID: 4EE6D746-0E71-4D08-A616-DB8FFDA8C1D5

**Scr > 0.7**	A = 0.7<br>B = -1.2	**Scr > 0.9**	*A* = 0.9<br>*B* = -1.2

Compare to https://www.mdcalc.com/calc/3939/ckd-epi-equations-glomerular-filtration-rate-gfr.

Duration of post covid illness [months]: Time from last COVID infection to CMR Baseline date calculated as (Baseline CMR date – date of last COVID infection +1)/30.4. If the last COVID infection was in the last 6 weeks (< 1,5 months), then use the penultimate COVID infection.

AEs of interest: AEs of interest are defined as the following events:

- All SAEs
- Losartan AEs (causal relationship to Losartan):
  - Hypotension with systolic BP <90 mmHg (AE_hypotension)
  - Dizziness, syncope (PT coding)
  - Angioedema (PT coding)
  - Rash and allergic pruritus (PT coding)
- Prednisolon AEs (causal relationship to Prednisolon):
  - Weight increased, Abnormal weight gain (PT coding) and/or more than 5 kg between baseline and visit week 16
  - Tachycardia with dose reduction (PT coding, action taken = dose decreased, permanently discontinued or temporarily discontinued)
  - Infections and parasitic diseases related to Prednisolon (PT coding, SOC="Infections and parasitic diseases" and related to Prednisolon)
- Gadovist AEs (causal relationship to Gadovist)

Time to first AE of interest: Time to first AE of interest is defined as time from start of therapy until first date of AE of interest or death by any cause and will be analyzed by Kaplan-Meier methods. Patients without documented AE of interest or death will be censored with their last activity date in the database known to be without AE of interest. If there are AEs, that occur in > 10% of all patients, a separate time-to-event analysis will be done for each of these AEs.

4.13 Use of baseline values

Baseline values LVEF, Absolute Work rate, VO_2max, VCO_2max, RER, AT, VE/ VCO_2, T1, T2, LVEDVI, LVESVI, RVEDVI, RVESVI, LVMI, GLS, AoAsc wall thickness, AoDesc wall thickness, Modified Canadian Chest pain scale, MRC Dyspnea Severity Score, NYHA Dyspnea Severity Score, PCS, MCS, HDL, LDL, Triglycerides, Cholesterol, Glucose, LipoA, HbA1c, TSH, eGFR, Sodium, Potassium, Creatinine, Troponin, NTproBNP, CRP, D-dimer, Fibrinogen and Hemoglobin will be used to calculate change from baseline.

Docusign Envelope ID: 4EE6D746-0E71-4D08-A616-DB8FFDA8C1D5

4.14 Use of covariates

Not applicable.

4.15 Identification of fixed and random factors

For the ANCOVA the variable defined in 4.16 will be used as fixed factors.

4.16 Subset analyses

All analysis will be stratified by treatment group (verum vs. placebo).

In addition, for the primary and secondary endpoints the tables are divided into treatment groups and the following subgroups:

- Heart rate at baseline (≥ 75 bpm vs. < 75 bpm)
- Heart rate control* (Patients with heart rate control vs. patients without heart rate control)
- Heart rate control during the study** (Patients with heart rate control during the study vs. patients without heart rate control during the study)
- COVID/Influenza during the study (Patients with COVID/Influenza during the study vs. Patients without COVID/Influenza during the study)***
- Antidepressants (yes vs. no)****
- Vaccination against COVID (Patients with at least one vaccination vs. Patients without vaccination)
- Duration between first COVID infection and Random date [days] (< Median vs. ≥ Median)
- Duration between last COVID infection and Random date [days] (< Median vs. ≥ Median)
- NTproBNP at baseline (≥ 125 pg/ml vs. < 125 pg/ml)
- D-dimer at baseline (≥ 500 ng/ml vs. < 500 ng/ml) or (< Median vs. ≥ Median), if the first distribution doesn't make sense
- Sex (Male vs. Female)
- Age (< Median vs. ≥ Median)

---

*Heart rate control is defined as Ivabradine (C01EB17), Bisoprolol (C07AB07) and β-blocker (C07 all). Use ATC coding. Time point of medication is not relevant.

**Start date of medication will be compared with the randomization date of the patient. Start date= unknown will be considered as prior therapy. If the start date is ≥ randomization date the medication is considered as treatment during the study.

***Use PT coding of AEs.

**** Use ATC coding of concomitant medications.

Docusign Envelope ID: 4EE6D746-0E71-4D08-A616-DB8FFDA8C1D5

4.17 Interim and follow-up analyses

No interim analyses are planned in this trial.

4.18 Study stopping rules

None.

4.19 Statistical significance levels

5% significance level. This will only be seen as confirmatory for the primary endpoint. All other p-values have to be interpreted in an explorative manner.

4.20 Methods for handling dropouts and protocol violators

Up to a maximum of 20 participants (i.e., appr. 6%) will be replaced due to dropouts and missing values of primary endpoint. Reasons for dropouts have to be documented in the CRF.

4.21 Methods for handling more than two treatment groups

Not applicable.

4.22 Methods for handling concomitant medications

All concomitant medication(s) administered must be reported in the case report form. Specifically:

Treatment with paracetamol or ibuprofen per needed basis (no regular therapy) as simple analgetic will be permitted.

Treatment with colchicine will not be permitted during the study period, due to confounding effects in placebo/prednisolone arm. Should pericarditis symptoms persist beyond the study period, colchicine can be administered after the completion of the study.

In participants with palpitations or excessive tachycardia (HR>85/min at rest), low dose Bisoprolol (1.25mg- 2.5 mg orally once a day) or Ivabradine (2.5 -5 mg orally twice a day) may be considered.

Prior COVID Vaccination is not an exclusion criterion. Any vaccination during the treatment period will not be permitted.

Initiation of new supplement therapy, including multivitamins, containing vitamin B, D, C and Coenzyme10 will be discouraged for the duration of the treatment period of this study (16 weeks).

In case of acute COVID infection during the treatment period, Paxlovid therapy will be permitted in line with the evidence.

All women will be considered as women of childbearing potential.

Docusign Envelope ID: 4EE6D746-0E71-4D08-A616-DB8FFDA8C1D5

4.23 Handling of therapy changes and unscheduled visits

Unscheduled visits will not be included in the analysis. Participants who change therapy before W16 will continue to perform all further planned study visits. Data up until the treatment change will be used for Per-Protocol analysis (as a while on treatment strategy for the intercurrent event of a treatment change), whereas the complete dataset will be used in the Intention to Treat (ITT) analysis (Following a treatment policy strategy).

4.24 Changes to the planned analyses

It was planned that compliance will be assessed by the participant diary and drug accountability (e.g. empty Dossett boxes) for IMP intake. According to incomplete patient diaries, we defined compliance according to the information in the visits.

5 Mock-ups for tables, listings and figures

For tables, listings and figures planned the grey text in curly braces shows the programmer where the parameter to be analyzed may be found and will not appear in the report. Class X (e.g. verum) and Class Y (e.g. placebo) are placeholders for the subgroup categories defined above. Of course, more than two categories are also possible.

The planned tables, list and figures are shown in the following document: MYOFLAME_SAP_Mockups_Final_v1.0_2025JUN10.docx

6 Statistical software

Statistical analyses will be performed using Statistical Analysis Software (SAS).

7 Medical dictionaries

Adverse event (AE) terms will be coded according to the Medical Dictionary of Regulatory Activities (MedDRA). For prior and concomitant diseases, the ICD10 catalog will be used. Concomitant medication will be coded according to ATC (Level 2).

8 Coding conventions

There are no special coding conventions. Free texts will not be encoded but listed only.

9 Output format

If a report is written in addition to the TLF, the respective tables and figures will be presented in the text and can be clear assigned. The text will be written in Arial, 11 pt with 1.5 line spacing, table in Arial, 10 pt.

10 List of the tables, lists, and figures planned for the Final Statistical Report

Refer to Mockups.

Docusign Envelope ID: 4EE6D746-0E71-4D08-A616-DB8FFDA8C1D5

11 History table

Version	Version of Mock-ups	Date (DD-MMM-YY)	Author	Sections changed	Brief description of change
Final v1.0	Final v1.0	11-JUN-2025	[redacted]	None	First version, hence no changes

Docusign Envelope ID: 4EE6D746-0E71-4D08-A616-DB8FFDA8C1D5

Mock-ups for tables, listings and figures

MYOFLAME-19

Randomised placebo controlled clinical trial of efficacy of MYOcardial protection with postacute inFLAMmatory cardiac involvEment due to COVID-19

**Project No:**	7021000_MYOFLAME-19
**Study Code:**	MYOFLAME-19
**Medication:**	Verum (Prednisolone + Losartan), Placebo (Placebo 1 + 2)
**Dokument ID:**	MYOFLAME_SAP_Mockups_Final_v1.0_2025JUN11.docx
**Version:**	Version 1.0
**Date:**	11.06.2025
**Sponsor:**	Goethe University Frankfurt<br>Institute for Experimental and Translational Cardiovascular Imaging<br>Theodor-Stern-Kai 7<br>60590 Frankfurt/Main, Germany
**Author:**	Alcedis GmbH<br>Winchesterstrasse 3<br>35394 Giessen, Germany<br>[redacted]

Docusign Envelope ID: 4EE6D746-0E71-4D08-A616-DB8FFDA8C1D5

Contents

Tables 8
Table 1: Disposition ... 8
Figure 1: Consort diagram ... 10
Baseline 11
Table 2: Socio-demographic data (for FAS and ITT separately) ... 11
Table 3: Medical history (for FAS and ITT separately) ... 14
Table 4: Relevant prior and/or concomitant medication (for FAS and ITT separately) ... 19
Table 5: Previous and concomitant diseases (for FAS and ITT separately) ... 20
Primary endpoints 21
Table 6: LVEF (absolute change) from baseline to 16 weeks post randomization – Test for normal distribution (for ITT and PP separately) ... 21
Table 7: Absolute LVEF (%) change to baseline at W16 (for ITT and PP separately) ... 22
Table 8: ANCOVA – LS-Means – Differences in LVEF (absolute change) from baseline to 16 weeks post randomization between the two treatment groups (for ITT and PP separately) ... 23
Figure 2: LVEF- Least Square mean changes (absolute) from baseline to week 16 between the two treatment groups (for ITT and PP separately) ... 24
Secondary endpoints – CMR and CPET 25
Table 9: Absolute achieved Work Rate change to baseline at W16 – Test for normal distribution (for ITT and PP separately) ... 25
Table 10: CPET- Absolute achieved Work Rate (W/min) change to baseline at W16 (for ITT and PP separately) ... 26
Table 11: CPET- ANCOVA – LS-Means – Differences in achieved Work Rate (absolute change) from baseline to 16 weeks post randomization between the two treatment groups (for ITT and PP separately) ... 27
Figure 3: CPET- achieved Work Rate - Least Square mean changes (absolute) from baseline to week 16 between the two treatment groups (for ITT and PP separately) ... 28
Table 12: Percentage achieved Work Rate change to baseline at W16 – Test for normal distribution (for ITT and PP separately) ... 29
Table 13: CPET- Percentage achieved Work Rate (W/min) change to baseline at W16 (for ITT and PP separately) ... 30
Table 14: CPET- ANCOVA – LS-Means – Differences in achieved Work Rate (percentage change) from baseline to 16 weeks post randomization between the two treatment groups (for ITT and PP separately) ... 31
Figure 4: CPET- achieved Work Rate - Least Square mean changes (percentage) from baseline to week 16 between the two treatment groups (for ITT and PP separately) ... 32
Table 15: Absolute VO_2max change to baseline at W16 – Test for normal distribution (for ITT and PP separately) ... 33
Table 16: CPET- Absolute VO_2max (l/min) change to baseline at W16 (for ITT and PP separately) ... 33
Table 17: CPET- ANCOVA – LS-Means – Differences in VO_2max (absolute change) from baseline to 16 weeks post randomization between the two treatment groups (for ITT and PP separately) ... 33
Figure 5: CPET- VO_2max - Least Square mean changes (absolute) from baseline to week 16 between the two treatment groups (for ITT and PP separately) ... 33
Table 18: Percentage VO_2max change to baseline at W16 – Test for normal distribution (for ITT and PP separately) ... 33
Table 19: CPET- Percentage VO_2max (l/min) change to baseline at W16 (for ITT and PP separately) ... 33

Docusign Envelope ID: 4EE6D746-0E71-4D08-A616-DB8FFDA8C1D5

Table 20: CPET- ANCOVA – LS-Means – Differences in VO_2max (percentage change) from baseline to 16 weeks post randomization between the two treatment groups (for ITT and PP separately) 33
Figure 6: CPET- VO_2max - Least Square mean changes (percentage) from baseline to week 16 between the two treatment groups (for ITT and PP separately) 33
Table 21: Absolute VCO_2max change to baseline at W16 – Test for normal distribution (for ITT and PP separately) 34
Table 22: CPET- Absolute VCO_2max (l/min) change to baseline at W16 (for ITT and PP separately).... 34
Table 23: CPET- ANCOVA – LS-Means – Differences in VCO_2max (absolute change) from baseline to 16 weeks post randomization between the two treatment groups (for ITT and PP separately) 34
Figure 7: CPET- VCO_2max - Least Square mean changes (absolute) from baseline to week 16 between the two treatment groups (for ITT and PP separately) 34
Table 24: Percentage VCO_2max change to baseline at W16 – Test for normal distribution (for ITT and PP separately).... 34
Table 25: CPET- Percentage VCO_2max (l/min) change to baseline at W16 (for ITT and PP separately) 34
Table 26: CPET- ANCOVA – LS-Means – Differences in VCO_2max (percentage change) from baseline to 16 weeks post randomization between the two treatment groups (for ITT and PP separately) 34
Figure 8: CPET- VCO_2max - Least Square mean changes (percentage) from baseline to week 16 between the two treatment groups (for ITT and PP separately) 34
Table 27: Absolute RER change to baseline at W16 – Test for normal distribution (for ITT and PP separately).... 35
Table 28: CPET- Absolute RER change to baseline at W16 (for ITT and PP separately).... 35
Table 29: CPET- ANCOVA – LS-Means – Differences in RER (absolute change) from baseline to 16 weeks post randomization between the two treatment groups (for ITT and PP separately) 35
Figure 9: CPET- RER - Least Square mean changes (absolute) from baseline to week 16 between the two treatment groups (for ITT and PP separately) 35
Table 30: Percentage RER change to baseline at W16 – Test for normal distribution (for ITT and PP separately) 35
Table 31: CPET- Percentage RER change to baseline at W16 (for ITT and PP separately).... 35
Table 32: CPET- ANCOVA – LS-Means – Differences in RER (percentage change) from baseline to 16 weeks post randomization between the two treatment groups (for ITT and PP separately) 35
Figure 10: CPET- RER - Least Square mean changes (percentage) from baseline to week 16 between the two treatment groups (for ITT and PP separately) 35
Table 33: Absolute AT change to baseline at W16 – Test for normal distribution (for ITT and PP separately) 36
Table 34: CPET- Absolute AT (l/min) change to baseline at W16 (for ITT and PP separately) 36
Table 35: CPET- ANCOVA – LS-Means – Differences in AT (absolute change) from baseline to 16 weeks post randomization between the two treatment groups (for ITT and PP separately) 36
Figure 11: CPET- AT - Least Square mean changes (absolute) from baseline to week 16 between the two treatment groups (for ITT and PP separately) .36
Table 36: Percentage AT change to baseline at W16 – Test for normal distribution (for ITT and PP separately) 36
Table 37: CPET- Percentage AT (l/min) change to baseline at W16 (for ITT and PP separately).... 36
Table 38: CPET- ANCOVA – LS-Means – Differences in AT (percentage change) from baseline to 16 weeks post randomization between the two treatment groups (for ITT and PP separately) 36
Figure 12: CPET- AT - Least Square mean changes (percentage) from baseline to week 16 between the two treatment groups (for ITT and PP separately) 36
Table 39: Absolute VE/VCO_2 slope change to baseline at W16 – Test for normal distribution (for ITT and PP separately).... 37
Table 40: CPET- Absolute VE/VCO_2 slope (l/min) change to baseline at W16 (for ITT and PP separately) 37

Docusign Envelope ID: 4EE6D746-0E71-4D08-A616-DB8FFDA8C1D5

Table 41: CPET- ANCOVA – LS-Means – Differences in VE/VCO_2 slope (absolute change) from baseline to 16 weeks post randomization between the two treatment groups (for ITT and PP separately) 37
Figure 13: CPET- VE/VCO_2 slope - Least Square mean changes (absolute) from baseline to week 16 between the two treatment groups (for ITT and PP separately) 37
Table 42: Percentage VE/VCO_2 slope change to baseline at W16 – Test for normal distribution (for ITT and PP separately) 37
Table 43: CPET- Percentage VE/VCO_2 slope (l/min) change to baseline at W16 (for ITT and PP separately) 37
Table 44: CPET- ANCOVA – LS-Means – Differences in VE/VCO_2 slope (percentage change) from baseline to 16 weeks post randomization between the two treatment groups (for ITT and PP separately) 37
Figure 14: CPET- VE/VCO_2 slope - Least Square mean changes (percentage) from baseline to week 16 between the two treatment groups (for ITT and PP separately) 37
Table 45: Absolute T1 change to baseline at W16 – Test for normal distribution (for ITT and PP separately) 38
Table 46: Absolute T1 (ms) change to baseline at W16 (for ITT and PP separately) 38
Table 47: ANCOVA – LS-Means – Differences in T1 (absolute change) from baseline to 16 weeks post randomization between the two treatment groups (for ITT and PP separately) 38
Figure 15: T1 - Least Square mean changes (absolute) from baseline to week 16 between the two treatment groups (for ITT and PP separately) 38
Table 48: Percentage T1 change to baseline at W16 – Test for normal distribution (for ITT and PP separately) 38
Table 49: Percentage T1 (ms) change to baseline at W16 (for ITT and PP separately) 38
Table 50: ANCOVA – LS-Means – Differences in T1 (percentage change) from baseline to 16 weeks post randomization between the two treatment groups (for ITT and PP separately) 38
Figure 16: T1 - Least Square mean changes (percentage) from baseline to week 16 between the two treatment groups (for ITT and PP separately) 38
Table 51: Absolute T2 change to baseline at W16 – Test for normal distribution (for ITT and PP separately) 39
Table 52: Absolute T2 (ms) change to baseline at W16 (for ITT and PP separately) 39
Table 53: ANCOVA – LS-Means – Differences in T2 (absolute change) from baseline to 16 weeks post randomization between the two treatment groups (for ITT and PP separately) 39
Figure 17: T2 - Least Square mean changes (absolute) from baseline to week 16 between the two treatment groups (for ITT and PP separately) 39
Table 54: Percentage T2 change to baseline at W16 – Test for normal distribution (for ITT and PP separately) 39
Table 55: Percentage T2 (ms) change to baseline at W16 (for ITT and PP separately) 39
Table 56: ANCOVA – LS-Means – Differences in T2 (percentage change) from baseline to 16 weeks post randomization between the two treatment groups (for ITT and PP separately) 39
Figure 18: T2 - Least Square mean changes (percentage) from baseline to week 16 between the two treatment groups (for ITT and PP separately) 39
Table 57: Absolute LVEDVI change to baseline at W16 – Test for normal distribution (for ITT and PP separately) 40
Table 58: Absolute LVEDVI (ml/m^2) change to baseline at W16 (for ITT and PP separately) 40
Table 59: ANCOVA – LS-Means – Differences in LVEDVI (absolute change) from baseline to 16 weeks post randomization between the two treatment groups (for ITT and PP separately) 40
Figure 19: LVEDVI - Least Square mean changes (absolute) from baseline to week 16 between the two treatment groups (for ITT and PP separately) 40
Table 60: Percentage LVEDVI change to baseline at W16 – Test for normal distribution (for ITT and PP separately) 40
Table 61: Percentage LVEDVI (ml/m^2) change to baseline at W16 (for ITT and PP separately) 40
Table 62: ANCOVA – LS-Means – Differences in LVEDVI (percentage change) from baseline to 16 weeks post randomization between the two treatment groups (for ITT and PP separately) 40
Figure 20: LVEDVI - Least Square mean changes (percentage) from baseline to week 16 between the two treatment groups (for ITT and PP separately).40

Docusign Envelope ID: 4EE6D746-0E71-4D08-A616-DB8FFDA8C1D5

Table 63: Absolute LVESVI change to baseline at W16 – Test for normal distribution (for ITT and PP separately) ... 41
Table 64: Absolute LVESVI (ml/m^2) change to baseline at W16 (for ITT and PP separately) ... 41
Table 65: ANCOVA – LS-Means – Differences in LVESVI (absolute change) from baseline to 16 weeks post randomization between the two treatment groups (for ITT and PP separately) ... 41
Figure 21: LVESVI - Least Square mean changes (absolute) from baseline to week 16 between the two treatment groups (for ITT and PP separately) ... 41
Table 66: Percentage LVESVI change to baseline at W16 – Test for normal distribution (for ITT and PP separately) ... 41
Table 67: Percentage LVESVI (ml/m^2) change to baseline at W16 (for ITT and PP separately) ... 41
Table 68: ANCOVA – LS-Means – Differences in LVESVI (percentage change) from baseline to 16 weeks post randomization between the two treatment groups (for ITT and PP separately) ... 41
Figure 22: LVESVI - Least Square mean changes (percentage) from baseline to week 16 between the two treatment groups (for ITT and PP separately) . 41
Table 69: Absolute RVEDVI change to baseline at W16 – Test for normal distribution (for ITT and PP separately) ... 42
Table 70: Absolute RVEDVI (ml/m^2) change to baseline at W16 (for ITT and PP separately) ... 42
Table 71: ANCOVA – LS-Means – Differences in RVEDVI (absolute change) from baseline to 16 weeks post randomization between the two treatment groups (for ITT and PP separately) ... 42
Figure 23: RVEDVI - Least Square mean changes (absolute) from baseline to week 16 between the two treatment groups (for ITT and PP separately) ... 42
Table 72: Percentage RVEDVI change to baseline at W16 – Test for normal distribution (for ITT and PP separately) ... 42
Table 73: Percentage RVEDVI (ml/m^2) change to baseline at W16 (for ITT and PP separately) ... 42
Table 74: ANCOVA – LS-Means – Differences in RVEDVI (percentage change) from baseline to 16 weeks post randomization between the two treatment groups (for ITT and PP separately) ... 42
Figure 24: RVEDVI - Least Square mean changes (percentage) from baseline to week 16 between the two treatment groups (for ITT and PP separately) 42
Table 75: Absolute RVESVI change to baseline at W16 – Test for normal distribution (for ITT and PP separately) ... 43
Table 76: Absolute RVESVI (ml/m^2) change to baseline at W16 (for ITT and PP separately) ... 43
Table 77: ANCOVA – LS-Means – Differences in RVESVI (absolute change) from baseline to 16 weeks post randomization between the two treatment groups (for ITT and PP separately) ... 43
Figure 25: RVESVI - Least Square mean changes (absolute) from baseline to week 16 between the two treatment groups (for ITT and PP separately) ... 43
Table 78: Percentage RVESVI change to baseline at W16 – Test for normal distribution (for ITT and PP separately) ... 43
Table 79: Percentage RVESVI (ml/m^2) change to baseline at W16 (for ITT and PP separately) ... 43
Table 80: ANCOVA – LS-Means – Differences in RVESVI (percentage change) from baseline to 16 weeks post randomization between the two treatment groups (for ITT and PP separately) ... 43
Figure 26: RVESVI - Least Square mean changes (percentage) from baseline to week 16 between the two treatment groups (for ITT and PP separately) 43
Table 81: Absolute LVMI change to baseline at W16 – Test for normal distribution (for ITT and PP separately) ... 44
Table 82: Absolute LVMI (g/m^2) change to baseline at W16 (for ITT and PP separately) ... 44
Table 83: ANCOVA – LS-Means – Differences in LVMI (absolute change) from baseline to 16 weeks post randomization between the two treatment groups (for ITT and PP separately) ... 44
Figure 27: LVMI - Least Square mean changes (absolute) from baseline to week 16 between the two treatment groups (for ITT and PP separately) ... 44
Table 84: Percentage LVMI change to baseline at W16 – Test for normal distribution (for ITT and PP separately) ... 44
Table 85: Percentage LVMI (g/m^2) change to baseline at W16 (for ITT and PP separately) ... 44
Table 86: ANCOVA – LS-Means – Differences in LVMI (percentage change) from baseline to 16 weeks post randomization between the two treatment groups (for ITT and PP separately) ... 44
Figure 28: LVMI - Least Square mean changes (percentage) from baseline to week 16 between the two treatment groups (for ITT and PP separately) ... 44

Docusign Envelope ID: 4EE6D746-0E71-4D08-A616-DB8FFDA8C1D5

Table 87: Absolute global longitudinal strain (GLS) change to baseline at W16 – Test for normal distribution (for ITT and PP separately) 45
Table 88: Absolute global longitudinal strain (GLS) (%) change to baseline at W16 (for ITT and PP separately) 45
Table 89: ANCOVA – LS-Means – Differences in GLS (absolute change) from baseline to 16 weeks post randomization between the two treatment groups (for ITT and PP separately) 45
Figure 29: GLS - Least Square mean changes (absolute) from baseline to week 16 between the two treatment groups (for ITT and PP separately) 45
Table 90: Percentage GLS change to baseline at W16 – Test for normal distribution (for ITT and PP separately) 45
Table 91: Percentage GLS (%) change to baseline at W16 (for ITT and PP separately) 45
Table 92: ANCOVA – LS-Means – Differences in GLS (percentage change) from baseline to 16 weeks post randomization between the two treatment groups (for ITT and PP separately) 45
Figure 30: GLS - Least Square mean changes (percentage) from baseline to week 16 between the two treatment groups (for ITT and PP separately) 45
Table 93: Absolute AoAsc wall thickness change to baseline at W16 – Test for normal distribution (for ITT and PP separately) 46
Table 94: Absolute AoAsc wall thickness (mm) change to baseline at W16 (for ITT and PP separately) 46
Table 95: ANCOVA – LS-Means – Differences in AoAsc wall thickness (absolute change) from baseline to 16 weeks post randomization between the two treatment groups (for ITT and PP separately) 46
Figure 31: AoAsc wall thickness - Least Square mean changes (absolute) from baseline to week 16 between the two treatment groups (for ITT and PP separately) 46
Table 96: Percentage AoAsc wall thickness change to baseline at W16 – Test for normal distribution (for ITT and PP separately) 46
Table 97: Percentage AoAsc wall thickness (mm) change to baseline at W16 (for ITT and PP separately) 46
Table 98: ANCOVA – LS-Means – Differences in AoAsc wall thickness (percentage change) from baseline to 16 weeks post randomization between the two treatment groups (for ITT and PP separately) 46
Figure 32: AoAsc wall thickness - Least Square mean changes (percentage) from baseline to week 16 between the two treatment groups (for ITT and PP separately) 46
Table 99: Absolute AoDesc wall thickness change to baseline at W16 – Test for normal distribution (for ITT and PP separately) 47
Table 100: Absolute AoDesc wall thickness (mm) change to baseline at W16 (for ITT and PP separately) 47
Table 101: ANCOVA – LS-Means – Differences in AoDesc wall thickness (absolute change) from baseline to 16 weeks post randomization between the two treatment groups (for ITT and PP separately) 47
Figure 33: AoDesc wall thickness - Least Square mean changes (absolute) from baseline to week 16 between the two treatment groups (for ITT and PP separately) 47
Table 102: Percentage AoDesc wall thickness change to baseline at W16 – Test for normal distribution (for ITT and PP separately) 47
Table 103: Percentage AoDesc wall thickness (mm) change to baseline at W16 (for ITT and PP separately) 47
Table 104: ANCOVA – LS-Means – Differences in AoDesc wall thickness (percentage change) from baseline to 16 weeks post randomization between the two treatment groups (for ITT and PP separately) 47
Figure 34: AoDesc wall thickness - Least Square mean changes (percentage) from baseline to week 16 between the two treatment groups (for ITT and PP separately) 47
Table 105: Number of Responders* at week 16 48
Secondary endpoints- Symptom scores and Quality of life (QoL) 49
Table 106: Long COVID Questionnaire- Patients with symptoms (for ITT and PP separately) 49
Figure 35: Long COVID Questionnaire- Patients with symptoms (for ITT and PP separately) 52
Table 107: Modified Canadian Chest pain scale (for ITT and PP separately) 53
Table 108: MRC Dyspnea Severity Score (for ITT and PP separately) 54

Docusign Envelope ID: 4EE6D746-0E71-4D08-A616-DB8FFDA8C1D5

Table 109: NYHA Dyspnea Severity Score (for ITT and PP separately) 55
Table 110: QoL- SF-36 questionnaire- Physical Component Summary scale (PCS) (for ITT and PP separately) 56
Table 111: QoL- SF-36 questionnaire- Mental Component Summary scale (MCS) (for ITT and PP separately) 57
Secondary endpoints- Compliance and tolerance 58
Table 112: End of treatment (for ITT and PP separately) 58
Table 113: Compliance* (for ITT and PP separately) 59
Table 114: Frequency* of prescribed medication consumed (for ITT and PP separately) 60
Table 115: Maximum Losartan dose based on patient diary* (for ITT and PP separately) 61
Table 116: Total cumulative steroid dose (Prednisolone) based on drug accountability (for ITT and PP separately) 62
Table 117: Total cumulative steroid dose (Prednisolone) based on patient diary* (for ITT and PP separately) 63
Figure 36: Total cumulative steroid dose (Prednisolone) based on patient diary* (for ITT and PP separately) 64
Table 118: Tolerance of therapy (for ITT and PP separately) 65
Secondary endpoints- Safety 67
Table 119: Safety Overview (SAF) 67
Table 120: AEs according to MedDRA- SOC and PT- Maximum intensity per patient (SAF) 68
Table 121: SAEs according to MedDRA- SOC and PT- Maximum intensity per patient (SAF) 70
Table 122: AEs with causal relationship to Prednisolone according to MedDRA- SOC and PT- Maximum intensity per patient (SAF) 72
Table 123: AEs with causal relationship to Losartan according to MedDRA- SOC and PT- Maximum intensity per patient (SAF) 74
Table 124: Proportion of patients with ADR (relationship to Losartan and/or Prednisolone) (SAF) 76
Table 125: Outcome of AEs (event based) (SAF) 77
Table 126: Time to first AE of interest [months] derived by Kaplan-Meier methods (SAF) 78
Figure 37: Time to first AE of interest [months] derived by Kaplan-Meier methods (SAF) 79
Table 127: Further safety information for patients with SAEs (SAF) 80
Table 128: Further safety information (SAF) 81
Table 129: Total amount of contrast agent Gadovist (for ITT and PP separately) 82
Table 130: AE related to contrast agent (SAF, event based) 83
Table 131: Frequent AEs* (SAF) 84
Table 132: Absolute changes in lipid profile compared to BL (SAF) 85
Table 133: Further laboratory values compared to BL (SAF) 88
Listings 91
Listing 1: Adverse events* (SAF) 91

Docusign Envelope ID: 4EE6D746-0E71-4D08-A616-DB8FFDA8C1D5

Tables

Table 1: Disposition

Disposition	N
Number of screened patients	
Patients excluded before randomization (e.g. not meeting inclusion and/or exclusion criteria, refuse to participate, other reasons)	
Number of randomized patients (FAS)	
Patients excluded from FAS (e.g. patients without two assessments with CMR (BL and follow-up), patient didn't receive at least 4 weeks of treatment)	
Patients in ITT set	
Patients excluded from ITT (e.g. Patients without another CMR at 16 weeks, not complied 75% with the study drug regimen)	
Patients in PP	
Patients in SAF (Patients who received at least one dose of IMP treatment)	
Premature study termination *{eos21.preterminated}*	
Yes	
No	
Missing (Patients without end of observation form)	
Reason for end of study *{eos21.reason}*	
Adverse event / Toxicity (without resulting in death)	
Suspected pregnancy or inadequate contraception	
Patient refused further study treatment	
Investigator's opinion	
Lack of compliance	
Protocol deviation	

Docusign Envelope ID: 4EE6D746-0E71-4D08-A616-DB8FFDA8C1D5

Disposition	N
Lost to follow-up	
Death	
Withdrawal of informed consent	
Other reason	

Docusign Envelope ID: 4EE6D746-0E71-4D08-A616-DB8FFDA8C1D5

Figure 1: Consort diagram

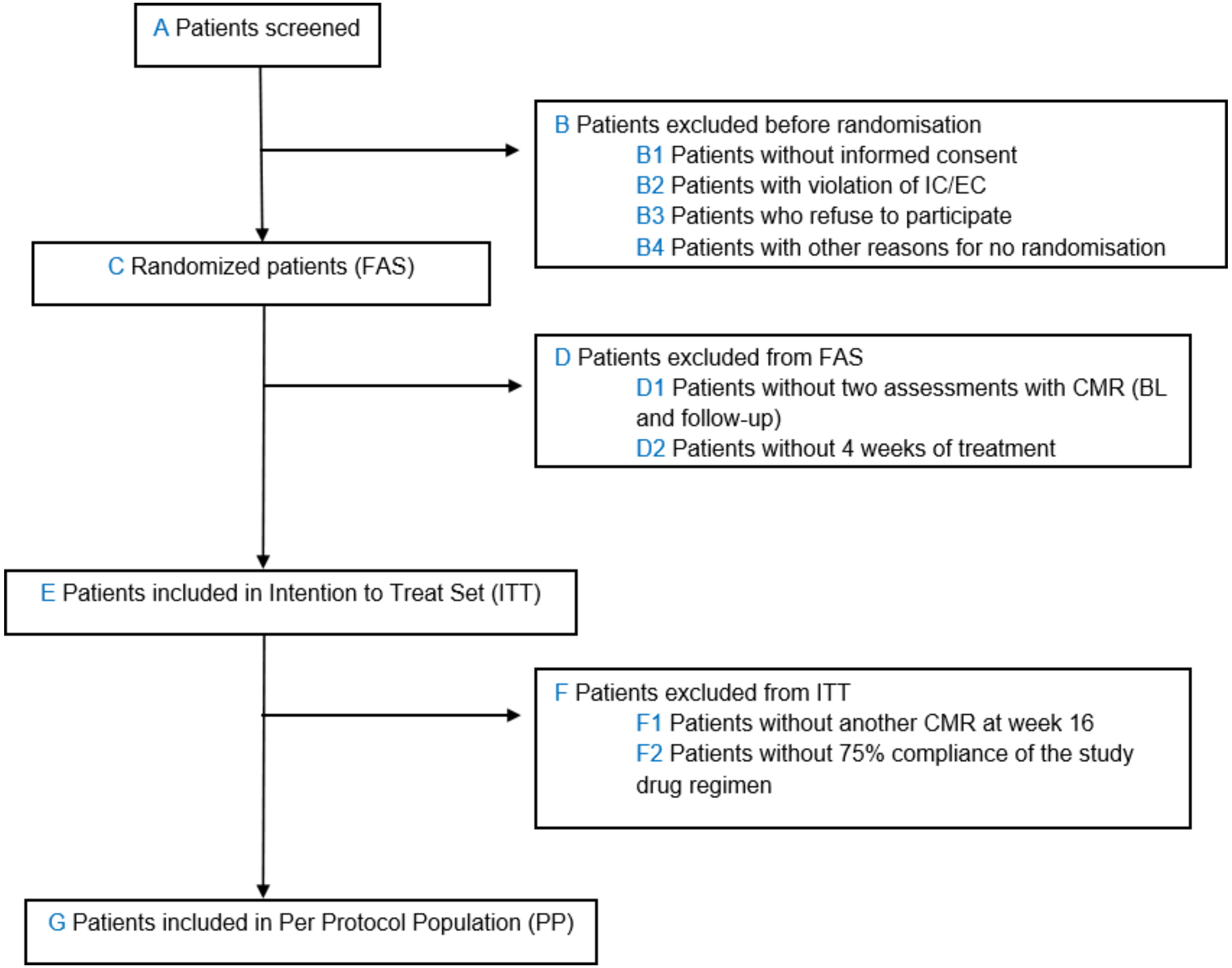

Docusign Envelope ID: 4EE6D746-0E71-4D08-A616-DB8FFDA8C1D5

Baseline

Class X (e.g. verum) and Class Y (e.g. placebo) are placeholders for the subgroup categories.

Table 2: Socio-demographic data (for FAS and ITT separately)

Socio-demographic data	Class X [N, %]	Class Y [N, %]	Total [N, %]
Number of patients	xx (100.00)	xx (100.00)	xx (100.00)
Age at baseline [years] *{prescreen.age}*			
N			
Mean			
SD			
Min			
Q1			
Median			
Q3			
Max			
Nmiss			
Gender *{prescreen.gender}*			
Male			
Female			
Diverse			
Missing			
Weight at baseline [kg] *{baseline.bodyweight}*			
N			
Mean			
SD			

Docusign Envelope ID: 4EE6D746-0E71-4D08-A616-DB8FFDA8C1D5

Min
Q1
Median
Q3
Max
Nmiss

Height at baseline [cm] *{baseline.bodysize}*

N
Mean
SD
Min
Q1
Median
Q3
Max
Nmiss

BMI at baseline [kg/m^2] *{baseline.bmi}*

N
Mean
SD
Min
Q1
Median
Q3
Max
Nmiss

Docusign Envelope ID: 4EE6D746-0E71-4D08-A616-DB8FFDA8C1D5

Ethnic origin *{prescreen.ethnic}*

Caucasian

Black

Asian

Other

Missing

Docusign Envelope ID: 4EE6D746-0E71-4D08-A616-DB8FFDA8C1D5

Table 3: Medical history (for FAS and ITT separately)

Medical history	Class X [N, %]	Class Y [N, %]	Total [N, %]
Number of patients	xx (100.00)	xx (100.00)	xx (100.00)
Number of COVID-19 infections per patient (categorical) *{anamnesis.covid_done}*			
1			
2			
3			
4			
≥ 5			
Unknown			
Number of COVID-19 infections per patient *{anamnesis.covid_done}*			
N			
Mean			
SD			
Min			
Q1			
Median			
Q3			
Max			
Nmiss			
Duration of post covid illness *{calculated}*			
N			
Mean			
SD			
Min			

Medical history	Class X [N, %]	Class Y [N, %]	Total [N, %]
Q1			
Median			
Q3			
Max			
Nmiss			
Duration of febrile COVID illness* [days] (per infection)			
N			
Mean			
SD			
Min			
Q1			
Median			
Q3			
Max			
Nmiss			
Hospitalization *{anamnesis_covid.hospitalized}*			
Patients with at least one hospitalization due to COVID***			
Patients without hospitalization			
Vaccination(s) against COVID-19 *{anamnesis.vac_done}*			
Yes			
No			
Missing			
Number of COVID-19 vaccinations per patient *{form anamnesis_vac}*			
1			

Docusign Envelope ID: 4EE6D746-0E71-4D08-A616-DB8FFDA8C1D5

Medical history	Class X [N, %]	Class Y [N, %]	Total [N, %]
2			
3			
≥ 4			
NYHA classification** at baseline *{anamnesis.nyha}*			
Class I			
Class II			
Class III			
Class IV			
Missing			
Symptoms at Baseline			
Fatigue (FA) *{anamnesis.fatigue}*			
Headache (HA) *{anamnesis.headache}*			
Shortness of breath (SOB) *{anamnesis.short_breath}*			
Loss of smell (LOS) *{anamnesis.odor_loss}*			
Persistent cough (PC) *{anamnesis.cough_irritation}*			
Sore throat (ST) *{anamnesis.sore_throat}*			
Fever (FV) *{anamnesis.fever}*			
Unusual muscle pains (UMP) *{anamnesis.limb_pain}*			
Skipped meals (SM) *{anamnesis.skipped_meals}*			
Chest Pain (CP) *{anamnesis.chest_pain}*			
Diarrhea (DI) *{anamnesis.diarrhea}*			
Hoarse Voice (HV) *{anamnesis.voice}*			
Abdominal Pain (AP) *{anamnesis.abdominal_pain}*			
Brain fog *{anamnesis.delirium}*			

Docusign Envelope ID: 4EE6D746-0E71-4D08-A616-DB8FFDA8C1D5

Medical history	Class X [N, %]	Class Y [N, %]	Total [N, %]
Loss of Consciousness (LOC) *{anamnesis.faint}*			
Almost unconscious *{anamnesis.faint_spec}*			
Syncope			
Resuscitation			
Excessive Tachycardia, heart racing (POTS) *{anamnesis.heart_racing}*			
MRC Dyspnea Severity Score *{anamnesis.short_breath_spec}*			
0= No symptoms (Fatigue=”no”)			
1= Breathless with strenuous exercise			
2= Short of breath when hurrying on the level or walking up a slight hill			
3= Walks slower than people of the same age on the level or stops for breath while walking at own pace on the level			
4= Stops for breath after walking 100m			
5= Too breathless to leave the house or breathless when dressing			
Modified Canadian Chest pain scale *{anamnesis.chest_pain_spec}*			
0= No symptoms (Chest pain=”no”)			
1= Presence of chest discomfort during strenuous, rapid, or prolonged ordinary activity (walking or climbing the stairs).			
2= Presence of chest discomfort during or after ordinary activities, when they are performed rapidly, or by change of position, under emotional stress, but also walking uphill, climbing more than one flight of ordinary stairs at a normal pace and under normal conditions.			
3= Presence of chest discomfort during or after activities of daily life at normal pace and conditions.			
4= No exertion needed to trigger chest pain, present at rest, recurring, or present at all times.			

* asymptomatic infections will be scored as “0 days”.

Docusign Envelope ID: 4EE6D746-0E71-4D08-A616-DB8FFDA8C1D5

** Class I: No limitation of physical activity. Ordinary physical activity does not cause symptoms.
Class II: Slight limitation of physical activity. Comfortable at rest. Ordinary physical activity results in symptoms.
Class III: Marked limitation of physical activity. Comfortable at rest. Presence of symptoms even with light loads.
Class IV: Experiences symptoms even while at rest.

*** All patients shouldn't have a hospitalization (violation of exclusion criteria).

Table 4: Relevant prior and/or concomitant medication (for FAS and ITT separately)

Relevant prior and/or concomitant medication	Class X [N, %]	Class Y [N, %]	Total [N, %]
Number of patients	xx (100.00)	xx (100.00)	xx (100.00)
Patients with at least one relevant prior and/or concomitant medication			
Relevant prior and/or concomitant medication according to WHO drug dictionary *{medication.drug}*			
ATC Name 1 (Level 2)			
ATC Name 2 (Level 2)			
…			

Table 5: Previous and concomitant diseases (for FAS and ITT separately)

Previous and concomitant diseases	Class X [N, %]	Class Y [N, %]	Total [N, %]
Number of patients	xx (100.00)	xx (100.00)	xx (100.00)
Patients with at least one previous or concomitant disease			
Previous and concomitant diseases according to ICD10 catalog *{disease.icd10}*			
Disease 1			
Disease 2			
…			

Docusign Envelope ID: 4EE6D746-0E71-4D08-A616-DB8FFDA8C1D5

Primary endpoints

Table 6: LVEF (absolute change) from baseline to 16 weeks post randomization – Test for normal distribution (for ITT and PP separately)

Test for normal distribution	p-value
Shapiro-Wilk-Test	x.xxxx

Assuming normal distribution if the p-value is greater than 0.05. If necessary, adequate data transformation, such as a Box-Cox transformation (e.g. logarithmic), will be performed to achieve normality.

Docusign Envelope ID: 4EE6D746-0E71-4D08-A616-DB8FFDA8C1D5

Table 7: Absolute LVEF (%) change to baseline at W16 (for ITT and PP separately)

Absolute LVEF (%) change to baseline at W16 *{cmr.lvef}*		Value at visit									Absolute change from baseline									p-value (T-test)
		N	Mean	SD	Min	Q1	Median	Q3	Max	Nmiss	N	Mean	SD	Min	Q1	Median	Q3	Max	Nmiss	
Class X	Baseline																			
	Week 16																			
Class Y	Baseline																			
	Week 16																			
Total	Baseline																			
	Week 16																			

If a large number of values are missing, LOCF will be considered for the LVEF to determine the primary endpoint. If data is imputed, primary analysis will be compared between the imputed data and the observed cases.

Docusign Envelope ID: 4EE6D746-0E71-4D08-A616-DB8FFDA8C1D5

Table 8: ANCOVA – LS-Means – Differences in LVEF (absolute change) from baseline to 16 weeks post randomization between the two treatment groups (for ITT and PP separately)

ANCOVA (Treatment group)- Least Square (LS)-Means	No of patients with values	BL values (Mean)	Change LS mean / Covariate estimate (95%CI)	Difference LS Means of Class X vs Class Y (95% CI)	p-value
Class X	xx	xx.x	xx.x (xx.x-xx.x)	xx.x (xx.x-xx.x)	x.xxxx
Class Y	xx	xx.x	xx.x (xx.x-xx.x)		
Covariate xxx			x.xxxx (xx.x-xx.x)		x.xxxx

Figure 2: LVEF- Least Square mean changes (absolute) from baseline to week 16 between the two treatment groups (for ITT and PP separately)

Figure		Example (no study data)
Title 1	LVEF- Least Square mean changes from baseline to week 16 between the two treatment groups	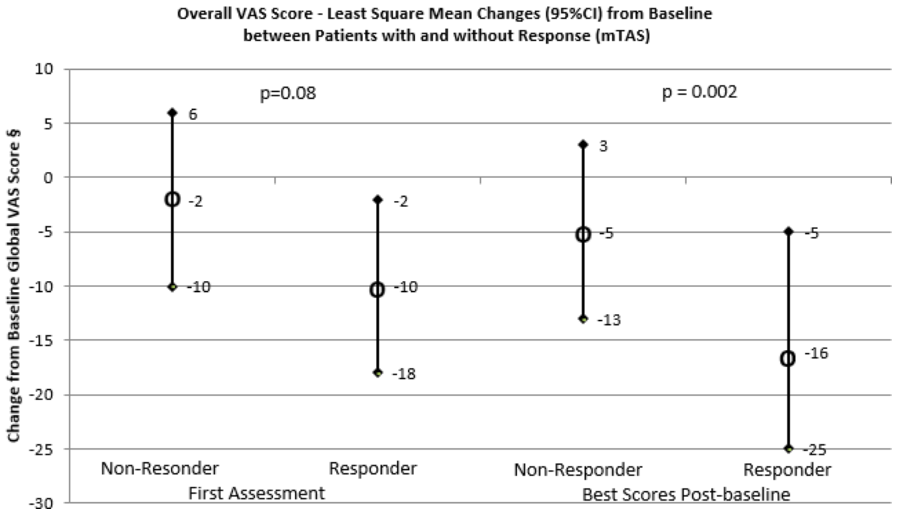
Title 2	(ITT, PP)	
Type of graph	Least square means plot	
y-axis	Week 16: verum vs. placebo	
y-axis (label)	-	
x-axis	Minimum to maximum ranges of 95% confidence intervals	
x-axis (label)	LS mean change from baseline	
Legend (if applicable)	-	
Footnote	-	
Additional information	Circles indicate LS mean. Squares indicate the 95% confidence intervals of the LS mean.	

Docusign Envelope ID: 4EE6D746-0E71-4D08-A616-DB8FFDA8C1D5

Secondary endpoints – CMR and CPET

Table 9: Absolute achieved Work Rate change to baseline at W16 – Test for normal distribution (for ITT and PP separately)

Test for normal distribution	p-value
Shapiro-Wilk-Test	x.xxxx

Assuming normal distribution if the p-value is greater than 0.05. If necessary, adequate data transformation, such as a Box-Cox transformation (e.g. logarithmic), will be performed to achieve normality.

Docusign Envelope ID: 4EE6D746-0E71-4D08-A616-DB8FFDA8C1D5

Table 10: CPET- Absolute achieved Work Rate (W/min) change to baseline at W16 (for ITT and PP separately)

Absolute achieved Work Rate (W/min) change to baseline at W16 *{baseline.awr}*		Value at visit									Absolute change from baseline								
		N	Mean	SD	Min	Q1	Median	Q3	Max	Nmiss	N	Mean	SD	Min	Q1	Median	Q3	Max	Nmiss
Class X	Baseline																		
	Week 16																		
Class Y	Baseline																		
	Week 16																		
Total	Baseline																		
	Week 16																		

Docusign Envelope ID: 4EE6D746-0E71-4D08-A616-DB8FFDA8C1D5

Table 11: CPET- ANCOVA – LS-Means – Differences in achieved Work Rate (absolute change) from baseline to 16 weeks post randomization between the two treatment groups (for ITT and PP separately)

ANCOVA (Treatment group)- Least Square (LS)-Means	No of patients with values	BL values (Mean)	Change LS mean / Covariate estimate (95%CI)	Difference LS Means of Class X vs Class Y (95% CI)	p-value
Class X	xx	xx.x	xx.x (xx.x-xx.x)	xx.x (xx.x-xx.x)	x.xxxx
Class Y	xx	xx.x	xx.x (xx.x-xx.x)		
Covariate xxx			x.xxxx (xx.x-xx.x)		x.xxxx

Docusign Envelope ID: 4EE6D746-0E71-4D08-A616-DB8FFDA8C1D5

Figure 3: CPET- achieved Work Rate - Least Square mean changes (absolute) from baseline to week 16 between the two treatment groups (for ITT and PP separately)

Figure		Example (no study data)
Title 1	achieved Work Rate - Least Square mean changes from baseline to week 16 between the two treatment groups	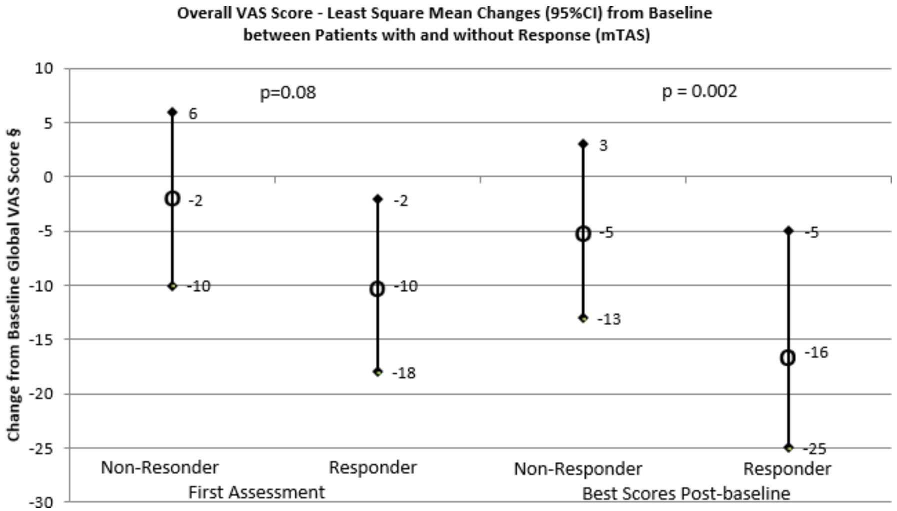
Title 2	(ITT, PP)	
Type of graph	Least square means plot	
y-axis	Week 16: verum vs. placebo	
y-axis (label)	-	
x-axis	Minimum to maximum ranges of 95% confidence intervals	
x-axis (label)	LS mean change from baseline	
Legend (if applicable)	-	
Footnote	-	
Additional information	Circles indicate LS mean. Squares indicate the 95% confidence intervals of the LS mean.	

Docusign Envelope ID: 4EE6D746-0E71-4D08-A616-DB8FFDA8C1D5

Table 12: Percentage achieved Work Rate change to baseline at W16 – Test for normal distribution (for ITT and PP separately)

Test for normal distribution	p-value
Shapiro-Wilk-Test	x.xxxx

Assuming normal distribution if the p-value is greater than 0.05. If necessary, adequate data transformation, such as a Box-Cox transformation (e.g. logarithmic), will be performed to achieve normality.

Table 13: CPET- Percentage achieved Work Rate (W/min) change to baseline at W16 (for ITT and PP separately)

Percentage achieved Work Rate (W/min) change to baseline at W16 *{baseline.awr}*		Value at visit									Percentage change from baseline								
		N	Mean	SD	Min	Q1	Median	Q3	Max	Nmiss	N	Mean	SD	Min	Q1	Median	Q3	Max	Nmiss
Class X	Baseline																		
	Week 16																		
Class Y	Baseline																		
	Week 16																		
Total	Baseline																		
	Week 16																		

Docusign Envelope ID: 4EE6D746-0E71-4D08-A616-DB8FFDA8C1D5

Table 14: CPET- ANCOVA – LS-Means – Differences in achieved Work Rate (percentage change) from baseline to 16 weeks post randomization between the two treatment groups (for ITT and PP separately)

ANCOVA (Treatment group)- Least Square (LS)-Means	No of patients with values	BL values (Mean)	Change LS mean / Covariate estimate (95%CI)	Difference LS Means of Class X vs Class Y (95% CI)	p-value
Class X	xx	xx.x	xx.x (xx.x-xx.x)	xx.x (xx.x-xx.x)	x.xxxx
Class Y	xx	xx.x	xx.x (xx.x-xx.x)		
Covariate xxx			x.xxxx (xx.x-xx.x)		x.xxxx

Docusign Envelope ID: 4EE6D746-0E71-4D08-A616-DB8FFDA8C1D5

Figure 4: CPET- achieved Work Rate - Least Square mean changes (percentage) from baseline to week 16 between the two treatment groups (for ITT and PP separately)

Figure		Example (no study data)
Title 1	achieved Work Rate - Least Square mean changes from baseline to week 16 between the two treatment groups	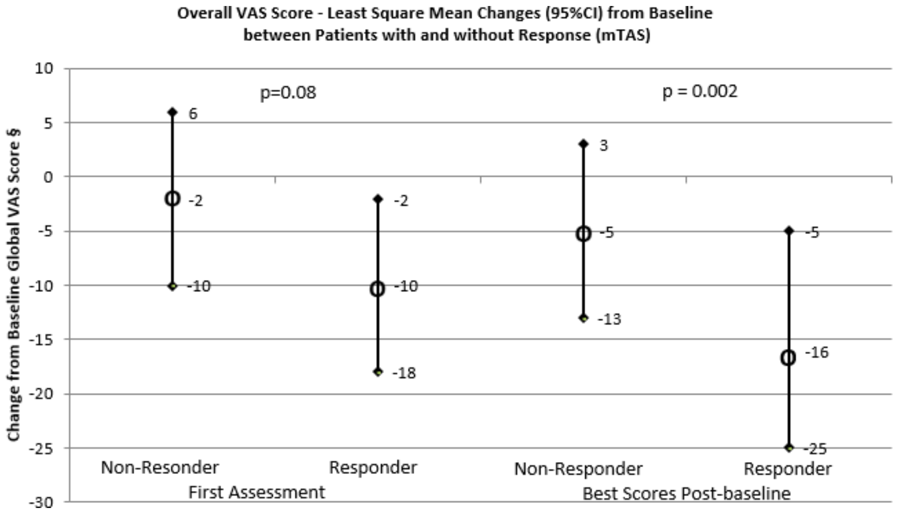
Title 2	(ITT, PP)	
Type of graph	Least square means plot	
y-axis	Week 16: verum vs. placebo	
y-axis (label)	-	
x-axis	Minimum to maximum ranges of 95% confidence intervals	
x-axis (label)	LS mean change from baseline	
Legend (if applicable)	-	
Footnote	-	
Additional information	Circles indicate LS mean. Squares indicate the 95% confidence intervals of the LS mean.	

Docusign Envelope ID: 4EE6D746-0E71-4D08-A616-DB8FFDA8C1D5

Table 15: Absolute VO_2max change to baseline at W16 – Test for normal distribution (for ITT and PP separately)
Analog to Table 9

Table 16: CPET- Absolute VO_2max (l/min) change to baseline at W16 (for ITT and PP separately)
Analog to Table 10

Table 17: CPET- ANCOVA – LS-Means – Differences in VO_2max (absolute change) from baseline to 16 weeks post randomization between the two treatment groups (for ITT and PP separately)
Analog to Table 11

Figure 5: CPET- VO_2max - Least Square mean changes (absolute) from baseline to week 16 between the two treatment groups (for ITT and PP separately)
Analog to Figure 3

Table 18: Percentage VO_2max change to baseline at W16 – Test for normal distribution (for ITT and PP separately)
Analog to Table 12

Table 19: CPET- Percentage VO_2max (l/min) change to baseline at W16 (for ITT and PP separately)
Analog to Table 13

Table 20: CPET- ANCOVA – LS-Means – Differences in VO_2max (percentage change) from baseline to 16 weeks post randomization between the two treatment groups (for ITT and PP separately)
Analog to Table 14

Figure 6: CPET- VO_2max - Least Square mean changes (percentage) from baseline to week 16 between the two treatment groups (for ITT and PP separately)
Analog to Figure 4

Docusign Envelope ID: 4EE6D746-0E71-4D08-A616-DB8FFDA8C1D5

Table 21: Absolute VCO_2max change to baseline at W16 – Test for normal distribution (for ITT and PP separately)
Analog to Table 9

Table 22: CPET- Absolute VCO_2max (l/min) change to baseline at W16 (for ITT and PP separately)
Analog to Table 10

Table 23: CPET- ANCOVA – LS-Means – Differences in VCO_2max (absolute change) from baseline to 16 weeks post randomization between the two treatment groups (for ITT and PP separately)
Analog to Table 11

Figure 7: CPET- VCO_2max - Least Square mean changes (absolute) from baseline to week 16 between the two treatment groups (for ITT and PP separately)
Analog to Figure 3

Table 24: Percentage VCO_2max change to baseline at W16 – Test for normal distribution (for ITT and PP separately)
Analog to Table 12

Table 25: CPET- Percentage VCO_2max (l/min) change to baseline at W16 (for ITT and PP separately)
Analog to Table 13

Table 26: CPET- ANCOVA – LS-Means – Differences in VCO_2max (percentage change) from baseline to 16 weeks post randomization between the two treatment groups (for ITT and PP separately)
Analog to Table 14

Figure 8: CPET- VCO_2max - Least Square mean changes (percentage) from baseline to week 16 between the two treatment groups (for ITT and PP separately)
Analog to Figure 4

Docusign Envelope ID: 4EE6D746-0E71-4D08-A616-DB8FFDA8C1D5

Table 27: Absolute RER change to baseline at W16 – Test for normal distribution (for ITT and PP separately)
Analog to Table 9

Table 28: CPET- Absolute RER change to baseline at W16 (for ITT and PP separately)
Analog to Table 10

Table 29: CPET- ANCOVA – LS-Means – Differences in RER (absolute change) from baseline to 16 weeks post randomization between the two treatment groups (for ITT and PP separately)
Analog to Table 11

Figure 9: CPET- RER - Least Square mean changes (absolute) from baseline to week 16 between the two treatment groups (for ITT and PP separately)
Analog to Figure 3

Table 30: Percentage RER change to baseline at W16 – Test for normal distribution (for ITT and PP separately)
Analog to Table 12

Table 31: CPET- Percentage RER change to baseline at W16 (for ITT and PP separately)
Analog to Table 13

Table 32: CPET- ANCOVA – LS-Means – Differences in RER (percentage change) from baseline to 16 weeks post randomization between the two treatment groups (for ITT and PP separately)
Analog to Table 14

Figure 10: CPET- RER - Least Square mean changes (percentage) from baseline to week 16 between the two treatment groups (for ITT and PP separately)
Analog to Figure 4

Docusign Envelope ID: 4EE6D746-0E71-4D08-A616-DB8FFDA8C1D5

Table 33: Absolute AT change to baseline at W16 – Test for normal distribution (for ITT and PP separately)
Analog to Table 9

Table 34: CPET- Absolute AT (l/min) change to baseline at W16 (for ITT and PP separately)
Analog to Table 10

Table 35: CPET- ANCOVA – LS-Means – Differences in AT (absolute change) from baseline to 16 weeks post randomization between the two treatment groups (for ITT and PP separately)
Analog to Table 11

Figure 11: CPET- AT - Least Square mean changes (absolute) from baseline to week 16 between the two treatment groups (for ITT and PP separately)
Analog to Figure 3

Table 36: Percentage AT change to baseline at W16 – Test for normal distribution (for ITT and PP separately)
Analog to Table 12

Table 37: CPET- Percentage AT (l/min) change to baseline at W16 (for ITT and PP separately)
Analog to Table 13

Table 38: CPET- ANCOVA – LS-Means – Differences in AT (percentage change) from baseline to 16 weeks post randomization between the two treatment groups (for ITT and PP separately)
Analog to Table 14

Figure 12: CPET- AT - Least Square mean changes (percentage) from baseline to week 16 between the two treatment groups (for ITT and PP separately)
Analog to Figure 4

Docusign Envelope ID: 4EE6D746-0E71-4D08-A616-DB8FFDA8C1D5

Table 39: Absolute VE/VCO_2 slope change to baseline at W16 – Test for normal distribution (for ITT and PP separately)
Analog to Table 9

Table 40: CPET- Absolute VE/VCO_2 slope (l/min) change to baseline at W16 (for ITT and PP separately)
Analog to Table 10

Table 41: CPET- ANCOVA – LS-Means – Differences in VE/VCO_2 slope (absolute change) from baseline to 16 weeks post randomization between the two treatment groups (for ITT and PP separately)
Analog to Table 11

Figure 13: CPET- VE/VCO_2 slope - Least Square mean changes (absolute) from baseline to week 16 between the two treatment groups (for ITT and PP separately)
Analog to Figure 3

Table 42: Percentage VE/VCO_2 slope change to baseline at W16 – Test for normal distribution (for ITT and PP separately)
Analog to Table 12

Table 43: CPET- Percentage VE/VCO_2 slope (l/min) change to baseline at W16 (for ITT and PP separately)
Analog to Table 13

Table 44: CPET- ANCOVA – LS-Means – Differences in VE/VCO_2 slope (percentage change) from baseline to 16 weeks post randomization between the two treatment groups (for ITT and PP separately)
Analog to Table 14

Figure 14: CPET- VE/VCO_2 slope - Least Square mean changes (percentage) from baseline to week 16 between the two treatment groups (for ITT and PP separately)
Analog to Figure 4

Docusign Envelope ID: 4EE6D746-0E71-4D08-A616-DB8FFDA8C1D5

Table 45: Absolute T1 change to baseline at W16 – Test for normal distribution (for ITT and PP separately)
Analog to Table 9

Table 46: Absolute T1 (ms) change to baseline at W16 (for ITT and PP separately)
Analog to Table 10

Table 47: ANCOVA – LS-Means – Differences in T1 (absolute change) from baseline to 16 weeks post randomization between the two treatment groups (for ITT and PP separately)
Analog to Table 11

Figure 15: T1 - Least Square mean changes (absolute) from baseline to week 16 between the two treatment groups (for ITT and PP separately)
Analog to Figure 3

Table 48: Percentage T1 change to baseline at W16 – Test for normal distribution (for ITT and PP separately)
Analog to Table 12

Table 49: Percentage T1 (ms) change to baseline at W16 (for ITT and PP separately)
Analog to Table 13

Table 50: ANCOVA – LS-Means – Differences in T1 (percentage change) from baseline to 16 weeks post randomization between the two treatment groups (for ITT and PP separately)
Analog to Table 14

Figure 16: T1 - Least Square mean changes (percentage) from baseline to week 16 between the two treatment groups (for ITT and PP separately)
Analog to Figure 4

Docusign Envelope ID: 4EE6D746-0E71-4D08-A616-DB8FFDA8C1D5

Table 51: Absolute T2 change to baseline at W16 – Test for normal distribution (for ITT and PP separately)
Analog to Table 9

Table 52: Absolute T2 (ms) change to baseline at W16 (for ITT and PP separately)
Analog to Table 10

Table 53: ANCOVA – LS-Means – Differences in T2 (absolute change) from baseline to 16 weeks post randomization between the two treatment groups (for ITT and PP separately)
Analog to Table 11

Figure 17: T2 - Least Square mean changes (absolute) from baseline to week 16 between the two treatment groups (for ITT and PP separately)
Analog to Figure 3

Table 54: Percentage T2 change to baseline at W16 – Test for normal distribution (for ITT and PP separately)
Analog to Table 12

Table 55: Percentage T2 (ms) change to baseline at W16 (for ITT and PP separately)
Analog to Table 13

Table 56: ANCOVA – LS-Means – Differences in T2 (percentage change) from baseline to 16 weeks post randomization between the two treatment groups (for ITT and PP separately)
Analog to Table 14

Figure 18: T2 - Least Square mean changes (percentage) from baseline to week 16 between the two treatment groups (for ITT and PP separately)
Analog to Figure 4

Docusign Envelope ID: 4EE6D746-0E71-4D08-A616-DB8FFDA8C1D5

Table 57: Absolute LVEDVI change to baseline at W16 – Test for normal distribution (for ITT and PP separately)
Analog to Table 9

Table 58: Absolute LVEDVI (ml/m^2) change to baseline at W16 (for ITT and PP separately)
Analog to Table 10

Table 59: ANCOVA – LS-Means – Differences in LVEDVI (absolute change) from baseline to 16 weeks post randomization between the two treatment groups (for ITT and PP separately)
Analog to Table 11

Figure 19: LVEDVI - Least Square mean changes (absolute) from baseline to week 16 between the two treatment groups (for ITT and PP separately)
Analog to Figure 3

Table 60: Percentage LVEDVI change to baseline at W16 – Test for normal distribution (for ITT and PP separately)
Analog to Table 12

Table 61: Percentage LVEDVI (ml/m^2) change to baseline at W16 (for ITT and PP separately)
Analog to Table 13

Table 62: ANCOVA – LS-Means – Differences in LVEDVI (percentage change) from baseline to 16 weeks post randomization between the two treatment groups (for ITT and PP separately)
Analog to Table 14

Figure 20: LVEDVI - Least Square mean changes (percentage) from baseline to week 16 between the two treatment groups (for ITT and PP separately)
Analog to Figure 4

Docusign Envelope ID: 4EE6D746-0E71-4D08-A616-DB8FFDA8C1D5

Table 63: Absolute LVESVI change to baseline at W16 – Test for normal distribution (for ITT and PP separately)
Analog to Table 9

Table 64: Absolute LVESVI (ml/m^2) change to baseline at W16 (for ITT and PP separately)
Analog to Table 10

Table 65: ANCOVA – LS-Means – Differences in LVESVI (absolute change) from baseline to 16 weeks post randomization between the two treatment groups (for ITT and PP separately)
Analog to Table 11

Figure 21: LVESVI - Least Square mean changes (absolute) from baseline to week 16 between the two treatment groups (for ITT and PP separately)
Analog to Figure 3

Table 66: Percentage LVESVI change to baseline at W16 – Test for normal distribution (for ITT and PP separately)
Analog to Table 12

Table 67: Percentage LVESVI (ml/m^2) change to baseline at W16 (for ITT and PP separately)
Analog to Table 13

Table 68: ANCOVA – LS-Means – Differences in LVESVI (percentage change) from baseline to 16 weeks post randomization between the two treatment groups (for ITT and PP separately)
Analog to Table 14

Figure 22: LVESVI - Least Square mean changes (percentage) from baseline to week 16 between the two treatment groups (for ITT and PP separately)
Analog to Figure 4

Docusign Envelope ID: 4EE6D746-0E71-4D08-A616-DB8FFDA8C1D5

Table 69: Absolute RVEDVI change to baseline at W16 – Test for normal distribution (for ITT and PP separately)
Analog to Table 9

Table 70: Absolute RVEDVI (ml/m^2) change to baseline at W16 (for ITT and PP separately)
Analog to Table 10

Table 71: ANCOVA – LS-Means – Differences in RVEDVI (absolute change) from baseline to 16 weeks post randomization between the two treatment groups (for ITT and PP separately)
Analog to Table 11

Figure 23: RVEDVI - Least Square mean changes (absolute) from baseline to week 16 between the two treatment groups (for ITT and PP separately)
Analog to Figure 3

Table 72: Percentage RVEDVI change to baseline at W16 – Test for normal distribution (for ITT and PP separately)
Analog to Table 12

Table 73: Percentage RVEDVI (ml/m^2) change to baseline at W16 (for ITT and PP separately)
Analog to Table 13

Table 74: ANCOVA – LS-Means – Differences in RVEDVI (percentage change) from baseline to 16 weeks post randomization between the two treatment groups (for ITT and PP separately)
Analog to Table 14

Figure 24: RVEDVI - Least Square mean changes (percentage) from baseline to week 16 between the two treatment groups (for ITT and PP separately)
Analog to Figure 4

Docusign Envelope ID: 4EE6D746-0E71-4D08-A616-DB8FFDA8C1D5

Table 75: Absolute RVESVI change to baseline at W16 – Test for normal distribution (for ITT and PP separately)
Analog to Table 9

Table 76: Absolute RVESVI (ml/m^2) change to baseline at W16 (for ITT and PP separately)
Analog to Table 10

Table 77: ANCOVA – LS-Means – Differences in RVESVI (absolute change) from baseline to 16 weeks post randomization between the two treatment groups (for ITT and PP separately)
Analog to Table 11

Figure 25: RVESVI - Least Square mean changes (absolute) from baseline to week 16 between the two treatment groups (for ITT and PP separately)
Analog to Figure 3

Table 78: Percentage RVESVI change to baseline at W16 – Test for normal distribution (for ITT and PP separately)
Analog to Table 12

Table 79: Percentage RVESVI (ml/m^2) change to baseline at W16 (for ITT and PP separately)
Analog to Table 13

Table 80: ANCOVA – LS-Means – Differences in RVESVI (percentage change) from baseline to 16 weeks post randomization between the two treatment groups (for ITT and PP separately)
Analog to Table 14

Figure 26: RVESVI - Least Square mean changes (percentage) from baseline to week 16 between the two treatment groups (for ITT and PP separately)
Analog to Figure 4

Docusign Envelope ID: 4EE6D746-0E71-4D08-A616-DB8FFDA8C1D5

Table 81: Absolute LVMI change to baseline at W16 – Test for normal distribution (for ITT and PP separately)
Analog to Table 9

Table 82: Absolute LVMI (g/m^2) change to baseline at W16 (for ITT and PP separately)
Analog to Table 10

Table 83: ANCOVA – LS-Means – Differences in LVMI (absolute change) from baseline to 16 weeks post randomization between the two treatment groups (for ITT and PP separately)
Analog to Table 11

Figure 27: LVMI - Least Square mean changes (absolute) from baseline to week 16 between the two treatment groups (for ITT and PP separately)
Analog to Figure 3

Table 84: Percentage LVMI change to baseline at W16 – Test for normal distribution (for ITT and PP separately)
Analog to Table 12

Table 85: Percentage LVMI (g/m^2) change to baseline at W16 (for ITT and PP separately)
Analog to Table 13

Table 86: ANCOVA – LS-Means – Differences in LVMI (percentage change) from baseline to 16 weeks post randomization between the two treatment groups (for ITT and PP separately)
Analog to Table 14

Figure 28: LVMI - Least Square mean changes (percentage) from baseline to week 16 between the two treatment groups (for ITT and PP separately)
Analog to Figure 4

Docusign Envelope ID: 4EE6D746-0E71-4D08-A616-DB8FFDA8C1D5

Table 87: Absolute global longitudinal strain (GLS) change to baseline at W16 – Test for normal distribution (for ITT and PP separately)
Analog to Table 9

Table 88: Absolute global longitudinal strain (GLS) (%) change to baseline at W16 (for ITT and PP separately)
Analog to Table 10

Table 89: ANCOVA – LS-Means – Differences in GLS (absolute change) from baseline to 16 weeks post randomization between the two treatment groups (for ITT and PP separately)
Analog to Table 11

Figure 29: GLS - Least Square mean changes (absolute) from baseline to week 16 between the two treatment groups (for ITT and PP separately)
Analog to Figure 3

Table 90: Percentage GLS change to baseline at W16 – Test for normal distribution (for ITT and PP separately)
Analog to Table 12

Table 91: Percentage GLS (%) change to baseline at W16 (for ITT and PP separately)
Analog to Table 13

Table 92: ANCOVA – LS-Means – Differences in GLS (percentage change) from baseline to 16 weeks post randomization between the two treatment groups (for ITT and PP separately)
Analog to Table 14

Figure 30: GLS - Least Square mean changes (percentage) from baseline to week 16 between the two treatment groups (for ITT and PP separately)
Analog to Figure 4

Docusign Envelope ID: 4EE6D746-0E71-4D08-A616-DB8FFDA8C1D5

Table 93: Absolute AoAsc wall thickness change to baseline at W16 – Test for normal distribution (for ITT and PP separately)
Analog to Table 9

Table 94: Absolute AoAsc wall thickness (mm) change to baseline at W16 (for ITT and PP separately)
Analog to Table 10

Table 95: ANCOVA – LS-Means – Differences in AoAsc wall thickness (absolute change) from baseline to 16 weeks post randomization between the two treatment groups (for ITT and PP separately)
Analog to Table 11

Figure 31: AoAsc wall thickness - Least Square mean changes (absolute) from baseline to week 16 between the two treatment groups (for ITT and PP separately)
Analog to Figure 3

Table 96: Percentage AoAsc wall thickness change to baseline at W16 – Test for normal distribution (for ITT and PP separately)
Analog to Table 12

Table 97: Percentage AoAsc wall thickness (mm) change to baseline at W16 (for ITT and PP separately)
Analog to Table 13

Table 98: ANCOVA – LS-Means – Differences in AoAsc wall thickness (percentage change) from baseline to 16 weeks post randomization between the two treatment groups (for ITT and PP separately)
Analog to Table 14

Figure 32: AoAsc wall thickness - Least Square mean changes (percentage) from baseline to week 16 between the two treatment groups (for ITT and PP separately)
Analog to Figure 4

Docusign Envelope ID: 4EE6D746-0E71-4D08-A616-DB8FFDA8C1D5

Table 99: Absolute AoDesc wall thickness change to baseline at W16 – Test for normal distribution (for ITT and PP separately)
Analog to Table 9

Table 100: Absolute AoDesc wall thickness (mm) change to baseline at W16 (for ITT and PP separately)
Analog to Table 10

Table 101: ANCOVA – LS-Means – Differences in AoDesc wall thickness (absolute change) from baseline to 16 weeks post randomization between the two treatment groups (for ITT and PP separately)
Analog to Table 11

Figure 33: AoDesc wall thickness - Least Square mean changes (absolute) from baseline to week 16 between the two treatment groups (for ITT and PP separately)
Analog to Figure 3

Table 102: Percentage AoDesc wall thickness change to baseline at W16 – Test for normal distribution (for ITT and PP separately)
Analog to Table 12

Table 103: Percentage AoDesc wall thickness (mm) change to baseline at W16 (for ITT and PP separately)
Analog to Table 13

Table 104: ANCOVA – LS-Means – Differences in AoDesc wall thickness (percentage change) from baseline to 16 weeks post randomization between the two treatment groups (for ITT and PP separately)
Analog to Table 14

Figure 34: AoDesc wall thickness - Least Square mean changes (percentage) from baseline to week 16 between the two treatment groups (for ITT and PP separately)
Analog to Figure 4

Table 105: Number of Responders* at week 16

Number of Responders at week 16	Class X		Class Y		Total	
	N	%	N	%	N	%
Patients with partial response						
Patients with total response						
Patients without response						
Number of patients						

*Partial response: a normal CMR result is defined as normal T1 and T2, normal gender-age predicted LVEF, non-dilated LV
Total response: in addition to the above absence of LGE

Docusign Envelope ID: 4EE6D746-0E71-4D08-A616-DB8FFDA8C1D5

Secondary endpoints- Symptom scores and Quality of life (QoL)

Table 106: Long COVID Questionnaire- Patients with symptoms (for ITT and PP separately)

Long COVID Questionnaire- Patients with symptoms		Baseline (BL)		Week 6 (W6)		Week 16 (W16)	
		N*	%	N*	%	N*	%
≥ 5 symptoms	Class X						
	Class Y						
	Total						
Fatigue (FA)	Class X						
	Class Y						
	Total						
Headache (HA)	Class X						
	Class Y						
	Total						
Shortness of breath (SOB)	Class X						
	Class Y						
	Total						
Loss of smell (LOS)	Class X						
	Class Y						
	Total						
Persistent cough (PC)	Class X						
	Class Y						
	Total						
Sore throat (ST)	Class X						
	Class Y						

Docusign Envelope ID: 4EE6D746-0E71-4D08-A616-DB8FFDA8C1D5

Long COVID Questionnaire- Patients with symptoms		Baseline (BL)		Week 6 (W6)		Week 16 (W16)	
		N*	%	N*	%	N*	%
	Total						
Fever (FV)	Class X						
	Class Y						
	Total						
Unusual muscle pains (UMP)	Class X						
	Class Y						
	Total						
Skipped meals (SM)	Class X						
	Class Y						
	Total						
Chest Pain (CP)	Class X						
	Class Y						
	Total						
Diarrhea (DI)	Class X						
	Class Y						
	Total						
Hoarse Voice (HV)	Class X						
	Class Y						
	Total						
Abdominal Pain (AP)	Class X						
	Class Y						
	Total						

Docusign Envelope ID: 4EE6D746-0E71-4D08-A616-DB8FFDA8C1D5

Long COVID Questionnaire- Patients with symptoms		Baseline (BL)		Week 6 (W6)		Week 16 (W16)	
		N*	%	N*	%	N*	%
Delirium (DE)	Class X						
	Class Y						
	Total						
Loss of Consciousness (LOC)	Class X						
	Class Y						
	Total						
Excessive Tachycardia, heart racing (POTS)	Class X						
	Class Y						
	Total						

*Patients who answered questionnaire at respective time.

Figure 35: Long COVID Questionnaire- Patients with symptoms (for ITT and PP separately)

**Figure**	
Title 1	Long COVID Questionnaire- Patients with symptoms
Title 2	(ITT, PP)
Type of graph	Bar chart
y-axis	0-100
y-axis (label)	Patients with symptoms (%)
x-axis	Groups of BL, W6 and W16 for each symptom and for ≥ 5 symptoms
x-axis (label)	≥ 5 symptoms, FA, HA, SOB, …
Legend (if applicable)	Arm A vs. Arm B
Footnote	FA=Fatigue, HA=Headache, SOB= Shortness of breath, …
Additional information	The bar chart will show one group for each symptom. One group consists of three bars, one for BL, one for W6 and one for W16. Each bar will be split in two sections, one for verum arm and one for placebo arm.

Example (no study data)

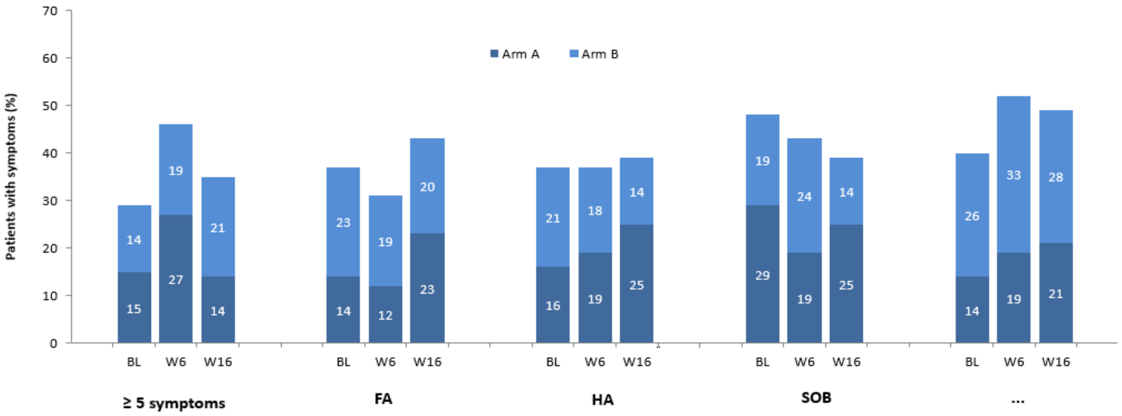

Table 107: Modified Canadian Chest pain scale (for ITT and PP separately)

Modified Canadian Chest pain scale *{anamnesis.chest_pain_spec}*		Value at visit									Absolute/Percentage* change from baseline								
		N	Mean	SD	Min	Q1	Median	Q3	Max	Nmiss	N	Mean	SD	Min	Q1	Median	Q3	Max	Nmiss
Class X	Baseline																		
	Week 6																		
	Week 16																		
Class Y	Baseline																		
	Week 6																		
	Week 16																		
Total	Baseline																		
	Week 6																		
	Week 16																		

*Table will be shown once for absolute and once for percentage change.

0= No symptoms (Chest pain=”no”)

1= Presence of chest discomfort during strenuous, rapid, or prolonged ordinary activity (walking or climbing the stairs).

2= Presence of chest discomfort during or after ordinary activities, when they are performed rapidly, or by change of position, under emotional stress, but also walking uphill, climbing more than one flight of ordinary stairs at a normal pace and under normal conditions.

3= Presence of chest discomfort during or after activities of daily life at normal pace and conditions.

4= No exertion needed to trigger chest pain, present at rest, recurring, or present at all times.

Docusign Envelope ID: 4EE6D746-0E71-4D08-A616-DB8FFDA8C1D5

Table 108: MRC Dyspnea Severity Score (for ITT and PP separately)

MRC Dyspnea Severity Score *{anamnesis.short_breath_spec}*		Value at visit									Absolute/ Percentage* change from baseline								
		N	Mean	SD	Min	Q1	Median	Q3	Max	Nmiss	N	Mean	SD	Min	Q1	Median	Q3	Max	Nmiss
Class X	Baseline																		
	Week 6																		
	Week 16																		
Class Y	Baseline																		
	Week 6																		
	Week 16																		
Total	Baseline																		
	Week 6																		
	Week 16																		

*Table will be shown once for absolute and once for percentage change.
0= No symptoms (Fatigue=”no”)
1= Breathless with strenuous exercise
2= Short of breath when hurrying on the level or walking up a slight hill
3= Walks slower than people of the same age on the level or stops for breath while walking at own pace on the level
4= Stops for breath after walking 100m
5= Too breathless to leave the house or breathless when dressing

Table 109: NYHA Dyspnea Severity Score (for ITT and PP separately)

NYHA Dyspnea Severity Score *{anamnesis.nyha}*		Value at visit									Absolute/ Percentage* change from baseline								
		N	Mean	SD	Min	Q1	Median	Q3	Max	Nmiss	N	Mean	SD	Min	Q1	Median	Q3	Max	Nmiss
Class X	Baseline																		
	Week 6																		
	Week 16																		
Class Y	Baseline																		
	Week 6																		
	Week 16																		
Total	Baseline																		
	Week 6																		
	Week 16																		

*Table will be shown once for absolute and once for percentage change.
1= Class I - No symptoms and no limitation in ordinary physical activity, e.g. shortness of breath when walking, climbing stairs etc.
2= Class II - Mild symptoms (mild shortness of breath and/or angina) and slight limitation during ordinary activity.
3= Class III - Marked limitation in activity due to symptoms, even during less-than-ordinary activity, e.g. walking short distances (20—100 m). Comfortable only at rest.
4= Class IV - Severe limitations. Experiences symptoms even while at rest. Mostly bedbound patients.

Docusign Envelope ID: 4EE6D746-0E71-4D08-A616-DB8FFDA8C1D5

Table 110: QoL- SF-36 questionnaire- Physical Component Summary scale (PCS) (for ITT and PP separately)

Physical Component Summary scale (PCS) *{qnr-rand36}*		Value at visit									Absolute/ Percentage* change from baseline								
		N	Mean	SD	Min	Q1	Median	Q3	Max	Nmiss	N	Mean	SD	Min	Q1	Median	Q3	Max	Nmiss
Class X	Baseline																		
	Week 6																		
	Week 16																		
Class Y	Baseline																		
	Week 6																		
	Week 16																		
Total	Baseline																		
	Week 6																		
	Week 16																		

*Table will be shown once for absolute and once for percentage change.

Table 111: QoL- SF-36 questionnaire- Mental Component Summary scale (MCS) (for ITT and PP separately)

Mental Component Summary scale (MCS) *{qnr-rand36}*		Value at visit									Absolute/ Percentage* change from baseline								
		N	Mean	SD	Min	Q1	Median	Q3	Max	Nmiss	N	Mean	SD	Min	Q1	Median	Q3	Max	Nmiss
Class X	Baseline																		
	Week 6																		
	Week 16																		
Class Y	Baseline																		
	Week 6																		
	Week 16																		
Total	Baseline																		
	Week 6																		
	Week 16																		

*Table will be shown once for absolute and once for percentage change.

Secondary endpoints- Compliance and tolerance

Table 112: End of treatment (for ITT and PP separately)

End of treatment	Class X [N, %]	Class Y [N, %]	Total [N, %]
Number of patients	xx (100.00)	xx (100.00)	xx (100.00)
Premature end of therapy *{eot.end_early}*			
Yes			
No			
Missing			
Reason for discontinuation of study treatment *{eot.reason}*			
Adverse event / Toxicity (without resulting in death)			
Suspected pregnancy or inadequate contraception			
Patient refused further study treatment			
Investigator's opinion			
Lack of compliance			
Protocol deviation			
Lost to follow-up			
Death			
Withdrawal of informed consent			
Other reason			

Docusign Envelope ID: 4EE6D746-0E71-4D08-A616-DB8FFDA8C1D5

Table 113: Compliance* (for ITT and PP separately)

Compliance *{calculated}*	N	Mean	SD	Min	Q1	Median	Q3	Max	Nmiss
Class X									
Class Y									
Total									

* Compliance with the study drug is defined as documented treatment at Week 2, 6 and 12.

Docusign Envelope ID: 4EE6D746-0E71-4D08-A616-DB8FFDA8C1D5

Table 114: Frequency* of prescribed medication consumed (for ITT and PP separately)

Frequency of prescribed medication consumed *{calculated}*	N	Mean	SD	Min	Q1	Median	Q3	Max	Nmiss
Class X									
Class Y									
Total									

*Frequency=1 means the medications were consumed every day, frequency=0.5 means the medications were consumed only half the days they should. The range of the frequency is between 0 and 1, a higher frequency means more frequent consumption.

Docusign Envelope ID: 4EE6D746-0E71-4D08-A616-DB8FFDA8C1D5

Table 115: Maximum Losartan dose based on patient diary* (for ITT and PP separately)

Maximum Losartan dose based on patient diary	Class X [N, %]	Class Y [N, %]	Total [N, %]
Number of patients	xx (100.00)	xx (100.00)	xx (100.00)
Maximum Losartan dose based on patient diary *{drugdiary.losartan_dose}*			
12.5 mg			
25 mg			
37.5 mg			
50 mg			

* Please note that there were incomplete or missing diaries.

Table 116: Total cumulative steroid dose (Prednisolone) based on drug accountability (for ITT and PP separately)

Total cumulative steroid dose (Prednisolone) based on drug accountability *{calculated}*	N	Mean	SD	Min	Q1	Median	Q3	Max	Nmiss
Class X									
Class Y									
Total									

Docusign Envelope ID: 4EE6D746-0E71-4D08-A616-DB8FFDA8C1D5

Table 117: Total cumulative steroid dose (Prednisolone) based on patient diary* (for ITT and PP separately)

Total cumulative steroid dose (Prednisolone) based on patient diary *{calculated}*	N	Mean	SD	Min	Q1	Median	Q3	Max	Nmiss
Class X									
Class Y									
Total									

* Please note that there were incomplete or missing diaries.

Docusign Envelope ID: 4EE6D746-0E71-4D08-A616-DB8FFDA8C1D5

Figure 36: Total cumulative steroid dose (Prednisolone) based on patient diary* (for ITT and PP separately)

**Figure**		**Example (no study data)**
Title 1	Total cumulative steroid dose (Prednisolone) based on patient diary	
Title 2	(ITT, PP)	
Type of graph	Line plot with range of SD	
y-axis	Max. cumulative dose (mg)	
y-axis (label)	Cumulative dose (mg)	
x-axis	0-122 (max. 16 weeks)	
x-axis (label)	Time (days)	
Legend (if applicable)	Mean	
Footnote		
Additional information	For each treatment group one line with the mean cumulative dose +/- SD will be shown.	

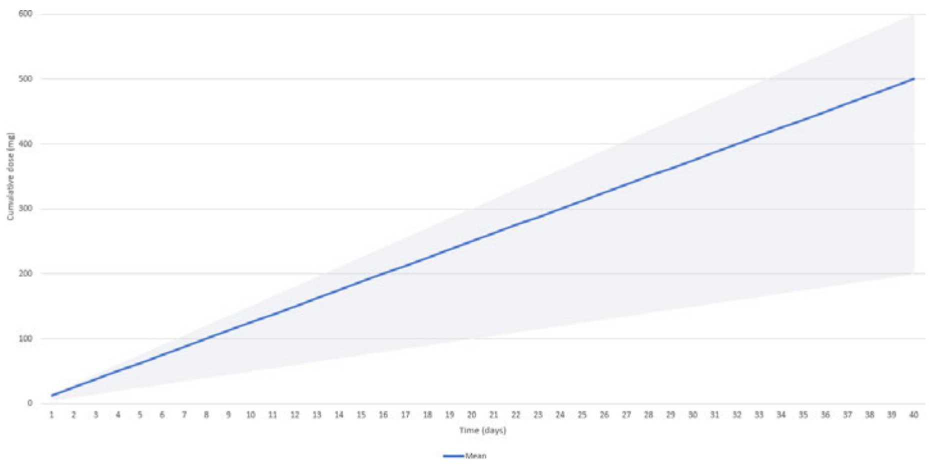

* Please note that there were incomplete or missing diaries.

Docusign Envelope ID: 4EE6D746-0E71-4D08-A616-DB8FFDA8C1D5

Table 118: Tolerance of therapy (for ITT and PP separately)

Tolerance of therapy	Class X [N, %]	Class Y [N, %]	Total [N, %]
Number of patients	xx (100.00)	xx (100.00)	xx (100.00)
Tolerance of Losartan *{ae.losartan_action}*			
Number of patients changing their treatment due to side effects (including temporarily and permanently discontinuation, dose increase and dose decrease)			
Number of patients with dose reduction due to side effects			
Number of patients with temporarily treatment discontinuation due to side effects			
Number of patients with permanently treatment discontinuation due to side effects			
Number of patients with dose reduction due to Hypotension			
Number of patients with temporarily treatment discontinuation due to Hypotension			
Number of patients with permanently treatment discontinuation due to Hypotension			
Tolerance of Prednisolone *{ae.prednisolone_action}*			
Number of patients changing their treatment due to side effects (including temporarily and permanently discontinuation, dose increase and dose decrease)			
Number of patients with dose reduction due to side effects			
Number of patients with temporarily treatment discontinuation due to side effects			
Number of patients with permanently treatment discontinuation due to side effects			
Number of patients with dose reduction due to Hypotension			
Number of patients with temporarily treatment discontinuation due to Hypotension			

Docusign Envelope ID: 4EE6D746-0E71-4D08-A616-DB8FFDA8C1D5

Tolerance of therapy	Class X [N, %]	Class Y [N, %]	Total [N, %]
Number of patients with permanently treatment discontinuation due to Hypotension			

Docusign Envelope ID: 4EE6D746-0E71-4D08-A616-DB8FFDA8C1D5

Secondary endpoints- Safety

Table 119: Safety Overview (SAF)

Safety Overview	Class X [N, %]	Class Y [N, %]	Total [N, %]
Number of patients	xx (100.00)	xx (100.00)	xx (100.00)
Patients with AE(s)			
Patients with SAE(s)			
Reason for seriousness (multiple answers possible)			
Reason 1			
Reason 2			
…			

Docusign Envelope ID: 4EE6D746-0E71-4D08-A616-DB8FFDA8C1D5

Table 120: AEs according to MedDRA- SOC and PT- Maximum intensity per patient (SAF)

AEs- Maximum intensity per patient		Mild		Moderate		Severe		Total	
		N	%	N	%	N	%	N	%
Class X	Number of patients								
	Patients without AE								
	Patients with at least one AE (Any SOC)								
	SOC 1								
	Any PT								
	PT 1								
	...								
	SOC ...								
Class Y	Number of patients								
	Patients without AE								
	Patients with at least one AE (Any SOC)								
	SOC 1								
	Any PT								
	PT 1								
	...								
	SOC ...								
Total	Number of patients								
	Patients without AE								
	Patients with at least one AE (Any SOC)								
	SOC 1								
	Any PT								
	PT 1								
	...								

Docusign Envelope ID: 4EE6D746-0E71-4D08-A616-DB8FFDA8C1D5

AEs- Maximum intensity per patient	Mild		Moderate		Severe		Total	
	N	%	N	%	N	%	N	%
SOC ...								

Docusign Envelope ID: 4EE6D746-0E71-4D08-A616-DB8FFDA8C1D5

Table 121: SAEs according to MedDRA- SOC and PT- Maximum intensity per patient (SAF)

SAEs- Maximum intensity per patient		Mild		Moderate		Severe		Total	
		N	%	N	%	N	%	N	%
Class X	Number of patients								
	Patients without SAE								
	Patients with at least one SAE (Any SOC)								
	SOC 1								
	Any PT								
	PT 1								
	...								
	SOC ...								
Class Y	Number of patients								
	Patients without SAE								
	Patients with at least one SAE (Any SOC)								
	SOC 1								
	Any PT								
	PT 1								
	...								
	SOC ...								
Total	Number of patients								
	Patients without SAE								
	Patients with at least one SAE (Any SOC)								
	SOC 1								
	Any PT								
	PT 1								
	...								

Docusign Envelope ID: 4EE6D746-0E71-4D08-A616-DB8FFDA8C1D5

SAEs- Maximum intensity per patient	Mild		Moderate		Severe		Total	
	N	%	N	%	N	%	N	%
SOC ...								

Docusign Envelope ID: 4EE6D746-0E71-4D08-A616-DB8FFDA8C1D5

Table 122: AEs with causal relationship to Prednisolone according to MedDRA- SOC and PT- Maximum intensity per patient (SAF)

AEs with causal relationship to Prednisolone		Mild		Moderate		Severe		Total	
		N	%	N	%	N	%	N	%
Class X	Number of patients								
	Patients without AE								
	Patients with at least one AE (Any SOC)								
	SOC 1								
	Any PT								
	PT 1								
	...								
	SOC ...								
Class Y	Number of patients								
	Patients without AE								
	Patients with at least one AE (Any SOC)								
	SOC 1								
	Any PT								
	PT 1								
	...								
	SOC ...								
Total	Number of patients								
	Patients without AE								
	Patients with at least one AE (Any SOC)								
	SOC 1								
	Any PT								
	PT 1								
	...								

Docusign Envelope ID: 4EE6D746-0E71-4D08-A616-DB8FFDA8C1D5

AEs with causal relationship to Prednisolone	Mild		Moderate		Severe		Total	
	N	%	N	%	N	%	N	%
SOC ...								

Docusign Envelope ID: 4EE6D746-0E71-4D08-A616-DB8FFDA8C1D5

Table 123: AEs with causal relationship to Losartan according to MedDRA- SOC and PT- Maximum intensity per patient (SAF)

AEs with causal relationship to Losartan		Mild		Moderate		Severe		Total	
		N	%	N	%	N	%	N	%
Class X	Number of patients								
	Patients without AE								
	Patients with at least one AE (Any SOC)								
	SOC 1								
	Any PT								
	PT 1								
	...								
	SOC ...								
Class Y	Number of patients								
	Patients without AE								
	Patients with at least one AE (Any SOC)								
	SOC 1								
	Any PT								
	PT 1								
	...								
	SOC ...								
Total	Number of patients								
	Patients without AE								
	Patients with at least one AE (Any SOC)								
	SOC 1								
	Any PT								
	PT 1								
	...								

Docusign Envelope ID: 4EE6D746-0E71-4D08-A616-DB8FFDA8C1D5

AEs with causal relationship to Losartan	Mild		Moderate		Severe		Total	
	N	%	N	%	N	%	N	%
SOC ...								

Docusign Envelope ID: 4EE6D746-0E71-4D08-A616-DB8FFDA8C1D5

Table 124: Proportion of patients with ADR (relationship to Losartan and/or Prednisolone) (SAF)

	N	Number of patients with ADR	Rate	95% CI
Class X				
Class Y				
Total				

Docusign Envelope ID: 4EE6D746-0E71-4D08-A616-DB8FFDA8C1D5

Table 125: Outcome of AEs (event based) (SAF)

Outcome of AEs (event based)	Class X [N, %]	Class Y [N, %]	Total [N, %]
Number of events	xx (100.00)	xx (100.00)	xx (100.00)
Fatal			
Recovered with sequelae			
Recovered without sequelae			
Ongoing			
Unknown			

Docusign Envelope ID: 4EE6D746-0E71-4D08-A616-DB8FFDA8C1D5

Table 126: Time to first AE of interest [months] derived by Kaplan-Meier methods (SAF)

Time to first AE of interest	N	First AE of interest / Death	Censored	Mean	Standard error	Median	CI 95%
Class X							
Class Y							
Total							

Docusign Envelope ID: 4EE6D746-0E71-4D08-A616-DB8FFDA8C1D5

Figure 37: Time to first AE of interest [months] derived by Kaplan-Meier methods (SAF)

**Figure**	
Title 1	Kaplan-Meier estimates for time until self-administration
Title 2	(SAF)
Type of graph	Kaplan-Meier plot
y-axis	0 to 1
y-axis (label)	Time to first AE of interest
x-axis	0 to maximum rounded up to nearest month
x-axis (label)	Months
Legend (if applicable)	Subgroup: Class X, Class Y
Footnote	-
Additional information	Patients at risk are displayed for specified timepoints on x-axis. One curve for each subgroup category will be displaying time to first AE of interest.

Example (no study data)

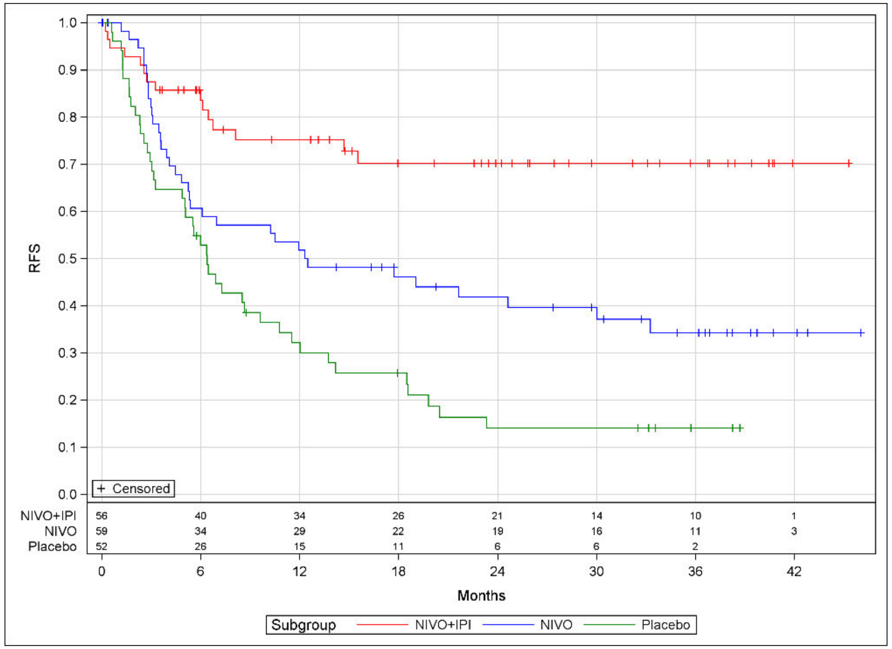

Docusign Envelope ID: 4EE6D746-0E71-4D08-A616-DB8FFDA8C1D5

Table 127: Further safety information for patients with SAEs (SAF)

Further safety information for patients with SAEs	Class X [N, %]	Class Y [N, %]	Total [N, %]
Number of patients	xx (100.00)	xx (100.00)	xx (100.00)
Patients with serious infectious complications (at least two of the following: fever ≥38.5°C, rise on hsCRP, neutrophilia, lymphocytosis, need for antiviral or antibiotic treatment) *{ae.infection}*			
Patients with symptomatic hypotension with systolic BP<90mmHg accompanied by blackout *{ae.hypotension}* or symptomatic tachycardia with heartrate>110/min accompanied by blackout *{ae.tachycardia}* or bradycardia *{ae meddra coding}*			
Patients with a significant rise in cardiac biomarkers (hsTNT, NTproBNP, >3-times the BL) *{ae.biomarker}*			
Patients with onset of clinical heart failure *{ae.heart_failure}*)			
Patients with acute psychotic episode *{ae.psychotic_episode}* or hypertensive crisis *{ae.hypertensive_crisis}*			

Table 128: Further safety information (SAF)

Further safety information	Class X [N, %]	Class Y [N, %]	Total [N, %]
Number of patients	xx (100.00)	xx (100.00)	xx (100.00)
Patients with a significant drop in eGFR compared to BL (>25%) *{compare baseline lab_val_cc.creatininec_value to all other documented eGFR values (creatininec_value) from the visits, eGFR needs to be marked as significant}*			
Patients with worsening of cardiovascular symptoms (increase in NYHA class *{compare anamnesis.nyha to all other documented nyha values from the visits (remote, visit3, visit5, unscheduled)}*)			

Docusign Envelope ID: 4EE6D746-0E71-4D08-A616-DB8FFDA8C1D5

Table 129: Total amount of contrast agent Gadovist (for ITT and PP separately)

Total amount of contrast agent Gadovist *{calculated}*		N	Mean	SD	Min	Q1	Median	Q3	Max	Nmiss
Class X	Baseline									
	Week 16									
Class Y	Baseline									
	Week 16									
Total	Baseline									
	Week 16									

Docusign Envelope ID: 4EE6D746-0E71-4D08-A616-DB8FFDA8C1D5

Table 130: AE related to contrast agent (SAF, event based)

AE related to contrast agent	Class X		Class Y		Total	
	N	%	N	%	N	%
AE related to contrast agent						
AE not related to contrast agent						
Number of AEs						

Docusign Envelope ID: 4EE6D746-0E71-4D08-A616-DB8FFDA8C1D5

Table 131: Frequent AEs* (SAF)

Frequent AEs	Class X		Class Y		Total		p-value
	N	%	N	%	N	%	(Chi Square test**)
Frequent AE 1							
Frequent AE 2							
…							
Number of patients							

*especially cardiac related; frequent AE means at least 10% of all patients had this AE, if no AEs are present in 10% of the patients show the 3 most often documented AEs
**or exact Fisher Test in case of small abs. frequencies with α=5%.

Docusign Envelope ID: 4EE6D746-0E71-4D08-A616-DB8FFDA8C1D5

Table 132: Absolute changes in lipid profile compared to BL (SAF)

Absolute changes in lipid profile compared to BL			Value at visit									Absolute change from baseline								
			N	Mean	SD	Min	Q1	Median	Q3	Max	Nmiss	N	Mean	SD	Min	Q1	Median	Q3	Max	Nmiss
HDL [mg/dl] *{lab_va_cc.hdl_value}*	Class X	Baseline																		
		Week 16																		
	Class Y	Baseline																		
		Week 16																		
	Total	Baseline																		
		Week 16																		
LDL [mg/dl] *{lab_va_cc.ldl_value}*	Class X	Baseline																		
		Week 16																		
	Class Y	Baseline																		
		Week 16																		
	Total	Baseline																		
		Week 16																		
Triglycerides [mg/dl] *{lab_va_cc.triglyceride_value}*	Class X	Baseline																		
		Week 16																		
	Class Y	Baseline																		
		Week 16																		
	Total	Baseline																		
		Week 16																		

Docusign Envelope ID: 4EE6D746-0E71-4D08-A616-DB8FFDA8C1D5

Absolute changes in lipid profile compared to BL			Value at visit									Absolute change from baseline								
			N	Mean	SD	Min	Q1	Median	Q3	Max	Nmiss	N	Mean	SD	Min	Q1	Median	Q3	Max	Nmiss
Cholesterol [mg/dl] *{lab_va_cc.cholesterolt _value}*	Class X	Baseline																		
		Week 16																		
	Class Y	Baseline																		
		Week 16																		
	Total	Baseline																		
		Week 16																		
Glucose [mg/dl] *{lab_va_cc.glucose_value}*	Class X	Baseline																		
		Week 16																		
	Class Y	Baseline																		
		Week 16																		
	Total	Baseline																		
		Week 16																		
LipoA [mg/dl] *{lab_va_cc.glucose_value}*	Class X	Baseline																		
		Week 16																		
	Class Y	Baseline																		
		Week 16																		
	Total	Baseline																		
		Week 16																		

Absolute changes in lipid profile compared to BL			Value at visit									Absolute change from baseline								
			N	Mean	SD	Min	Q1	Median	Q3	Max	Nmiss	N	Mean	SD	Min	Q1	Median	Q3	Max	Nmiss
HbA1c [%] *{lab_va_cc.hba1c_value}*	Class X	Baseline																		
		Week 16																		
	Class Y	Baseline																		
		Week 16																		
	Total	Baseline																		
		Week 16																		
TSH [mU/l] *{lab_va_cc.tsh_value}*	Class X	Baseline																		
		Week 16																		
	Class Y	Baseline																		
		Week 16																		
	Total	Baseline																		
		Week 16																		
eGFR** [ml/min/1.73 m²] *{calculated}*	Class X	Baseline																		
		Week 16																		
	Class Y	Baseline																		
		Week 16																		
	Total	Baseline																		
		Week 16																		

*If there are HbA1c values documented in another unit than %, please consult with DM and sponsor.
** The eCRF was calculated, the documented eGFR values from the eCRF were not considered.

Docusign Envelope ID: 4EE6D746-0E71-4D08-A616-DB8FFDA8C1D5

Table 133: Further laboratory values compared to BL (SAF)

Further laboratory values compared to BL			Value at visit									Absolute change from baseline								
			N	Mean	SD	Min	Q1	Median	Q3	Max	Nmiss	N	Mean	SD	Min	Q1	Median	Q3	Max	Nmiss
Sodium [mmol/l] *{lab_va_cc.sodium_value}*	Class X	Baseline																		
		Week 16																		
	Class Y	Baseline																		
		Week 16																		
	Total	Baseline																		
		Week 16																		
Potassium [mmol/l] *{lab_va_cc.potassium_value}*	Class X	Baseline																		
		Week 16																		
	Class Y	Baseline																		
		Week 16																		
	Total	Baseline																		
		Week 16																		
Creatinine [mg/dl] *{lab_va_cc.creatinine_value}*	Class X	Baseline																		
		Week 16																		
	Class Y	Baseline																		
		Week 16																		
	Total	Baseline																		
		Week 16																		

Docusign Envelope ID: 4EE6D746-0E71-4D08-A616-DB8FFDA8C1D5

Further laboratory values compared to BL			Value at visit									Absolute change from baseline								
			N	Mean	SD	Min	Q1	Median	Q3	Max	Nmiss	N	Mean	SD	Min	Q1	Median	Q3	Max	Nmiss
Troponin [ng/ml] *{lab_va_cc.cc30_value}*	Class X	Baseline																		
		Week 16																		
	Class Y	Baseline																		
		Week 16																		
	Total	Baseline																		
		Week 16																		
NTproBNP [pg/ml] *{lab_va_cc.cc32_value}*	Class X	Baseline																		
		Week 16																		
	Class Y	Baseline																		
		Week 16																		
	Total	Baseline																		
		Week 16																		
CRP [mg/dl] *{lab_va_cc.crp_value}*	Class X	Baseline																		
		Week 16																		
	Class Y	Baseline																		
		Week 16																		
	Total	Baseline																		
		Week 16																		

Further laboratory values compared to BL			Value at visit									Absolute change from baseline								
			N	Mean	SD	Min	Q1	Median	Q3	Max	Nmiss	N	Mean	SD	Min	Q1	Median	Q3	Max	Nmiss
D-dimer [ng/ml] *{lab_va_co.co06_value}*	Class X	Baseline																		
		Week 16																		
	Class Y	Baseline																		
		Week 16																		
	Total	Baseline																		
		Week 16																		
Fibrinogen [g/l] *{lab_va_co.co07_value}*	Class X	Baseline																		
		Week 16																		
	Class Y	Baseline																		
		Week 16																		
	Total	Baseline																		
		Week 16																		
Hemoglobin [g/l] *{lab_va_he.haemoglobin_value}*	Class X	Baseline																		
		Week 16																		
	Class Y	Baseline																		
		Week 16																		
	Total	Baseline																		
		Week 16																		

Docusign Envelope ID: 4EE6D746-0E71-4D08-A616-DB8FFDA8C1D5

Listings

Listing 1: Adverse events* (SAF)

Site	Patient No	Age	Sex	PT term	Start of AE	End of AE	Outcome	Action taken	Causality Losartan	Causality Prednisolone	Serious?	Reason for seriousness

*The AE listing is not displayed for the final analysis, because the study is ongoing and the unblinding will only be carried out at the end of the study.

docusign

Abschlusszertifikat

Umschlag-ID: 4EE6D746-0E71-4D08-A616-DB8FFDA8C1D5 | Status: Abgeschlossen

Betreff: Mit Docusign abschließen: MYOFLAME_SAP_Mockups_Final_v1.0_2025JUN11.docx, MYOFLAME_SAP_Final_v1...

Quellumschlag:

Dokumentenseiten: 112	Signaturen: 2	Umschlagersteller:
Zertifikatsseiten: 5	Initialen: 0	[redacted]
Signatur mit Anleitung: Aktiviert		
Umschlag-ID-Stempel: Aktiviert		
Zeitzone: (UTC+01:00) Amsterdam, Berlin, Bern, Rom, Stockholm, Wien		

Eintragsverfolgung

Status: Original<br>12.06.2025 08:12:10	[redacted]	Standort: DocuSign

Unterzeichnerereignisse	Signatur	Zeitstempel
[redacted]<br>Biometry<br>Alcedis GmbH<br>Sicherheitsstufe: E-Mail, Kontoauthentifizierung (keine), Digitales Zertifikat<br>**Signaturanbieterdetails:**<br>Signaturtyp: DocuSign Protect & Sign (Client ID: DDE5E85D-4085-40B6-8785-DA3CCD16D81E)<br>Signaturaussteller: DocuSign Cloud Signing CA - SI1<br>Authentifizierung: Zugriffscode<br>**Vereinbarung bezüglich elektronischer Unterlagen und Signaturen:**<br>Nicht über Docusign angeboten	[redacted]<br>Signaturübernahme: Hochgeladenes Signaturbild<br>[redacted]<br>Standort des Signaturanbieters: https://ps-ws.dsf.docusign.net/ds-server/s/noauth/psm/tsp/sign	Gesendet: 12.06.2025 08:18:33<br>Eingesehen: 12.06.2025 08:18:39<br>Signiert: 12.06.2025 08:19:03
[redacted]<br>Sicherheitsstufe: E-Mail, Kontoauthentifizierung (keine), Digitales Zertifikat<br>**Signaturanbieterdetails:**<br>Signaturtyp: DocuSign Protect & Sign (Client ID: DDE5E85D-4085-40B6-8785-DA3CCD16D81E)<br>Signaturaussteller: DocuSign Cloud Signing CA - SI1<br>Authentifizierung: Zugriffscode<br>**Vereinbarung bezüglich elektronischer Unterlagen und Signaturen:**<br>Akzeptiert: 06.11.2023 13:51:24<br>ID: f06e3449-abad-405c-84cc-67c29b38baf2	[redacted]<br>Signaturübernahme: Vorgegebener Stil<br>[redacted]<br>Standort des Signaturanbieters: https://ps-ws.dsf.docusign.net/ds-server/s/noauth/psm/tsp/sign	Gesendet: 12.06.2025 08:18:34<br>Eingesehen: 12.06.2025 09:09:21<br>Signiert: 12.06.2025 09:10:36

Vor-Ort-Unterzeichner – Ereignisse	Signatur	Zeitstempel

Bearbeiterversandereignisse	Status	Zeitstempel

Beauftragtenzustellereignisse	Status	Zeitstempel

Vermittlerversandereignisse	Status	Zeitstempel

Zertifizierter Versand - Ereignisse	Status	Zeitstempel

Kopienereignisse	Status	Zeitstempel

Zeugen-Ereignisse	Signatur	Zeitstempel

Notarereignisse	Signatur	Zeitstempel

Umschlagereignisse – Überblick	Status	Zeitstempel
Umschlag gesendet	Hash-codiert/verschlüsselt	12.06.2025 08:18:34
Zertifiziert zugestellt	Sicherheitsprüfung ausgeführt	12.06.2025 09:09:21
Signiervorgang abgeschlossen	Sicherheitsprüfung ausgeführt	12.06.2025 09:10:36
Abgeschlossen	Sicherheitsprüfung ausgeführt	12.06.2025 09:10:37

Zahlungen	Status	Zeitstempel

Vereinbarung bezüglich elektronischer Unterlagen und Signaturen

ELECTRONIC RECORD AND SIGNATURE DISCLOSURE

From time to time, [redacted]we, us or Company) may be required by law to provide to you certain written notices or disclosures. Described below are the terms and conditions for providing to you such notices and disclosures electronically through the DocuSign system. Please read the information below carefully and thoroughly, and if you can access this information electronically to your satisfaction and agree to this Electronic Record and Signature Disclosure (ERSD), please confirm your agreement by selecting the check-box next to 'I agree to use electronic records and signatures' before clicking 'CONTINUE' within the DocuSign system.

Getting paper copies

At any time, you may request from us a paper copy of any record provided or made available electronically to you by us. You will have the ability to download and print documents we send to you through the DocuSign system during and immediately after the signing session and, if you elect to create a DocuSign account, you may access the documents for a limited period of time (usually 30 days) after such documents are first sent to you. After such time, if you wish for us to send you paper copies of any such documents from our office to you, you will be charged a $0.00 per-page fee. You may request delivery of such paper copies from us by following the procedure described below.

Withdrawing your consent

If you decide to receive notices and disclosures from us electronically, you may at any time change your mind and tell us that thereafter you want to receive required notices and disclosures only in paper format. How you must inform us of your decision to receive future notices and disclosure in paper format and withdraw your consent to receive notices and disclosures electronically is described below.

Consequences of changing your mind

If you elect to receive required notices and disclosures only in paper format, it will slow the speed at which we can complete certain steps in transactions with you and delivering services to you because we will need first to send the required notices or disclosures to you in paper format, and then wait until we receive back from you your acknowledgment of your receipt of such paper notices or disclosures. Further, you will no longer be able to use the DocuSign system to receive required notices and consents electronically from us or to sign electronically documents from us.

All notices and disclosures will be sent to you electronically

Unless you tell us otherwise in accordance with the procedures described herein, we will provide electronically to you through the DocuSign system all required notices, disclosures, authorizations, acknowledgements, and other documents that are required to be provided or made available to you during the course of our relationship with you. To reduce the chance of you inadvertently not receiving any notice or disclosure, we prefer to provide all of the required notices and disclosures to you by the same method and to the same address that you have given us. Thus, you can receive all the disclosures and notices electronically or in paper format through the paper mail delivery system. If you do not agree with this process, please let us know as described below. Please also see the paragraph immediately above that describes the consequences of your electing not to receive delivery of the notices and disclosures electronically from us.

How to contact Alcedis GmbH:

You may contact us to let us know of your changes as to how we may contact you electronically, to request paper copies of certain information from us, and to withdraw your prior consent to receive notices and disclosures electronically as follows:
To contact us by email send messages to: [redacted]

To advise Alcedis GmbH of your new email address

To let us know of a change in your email address where we should send notices and disclosures electronically to you, you must send an email message to us [redacted] and in the body of such request you must state: your previous email address, your new email address. We do not require any other information from you to change your email address.

If you created a DocuSign account, you may update it with your new email address through your account preferences.

To request paper copies from Alcedis GmbH

To request delivery from us of paper copies of the notices and disclosures previously provided by us to you electronically, you must send us an email t[redacted]e and in the body of such request you must state your email address, full name, mailing address, and telephone number. We will bill you for any fees at that time, if any.

To withdraw your consent with Alcedis GmbH

To inform us that you no longer wish to receive future notices and disclosures in electronic format you may:

i. decline to sign a document from within your signing session, and on the subsequent page, select the check-box indicating you wish to withdraw your consent, or you may;

ii. send us an email to [redacted] and in the body of such request you must state your email, full name, mailing address, and telephone number. We do not need any other information from you to withdraw consent.. The consequences of your withdrawing consent for online documents will be that transactions may take a longer time to process..

Required hardware and software

The minimum system requirements for using the DocuSign system may change over time. The current system requirements are found here: https://support.docusign.com/guides/signer-guide-signing-system-requirements.

Acknowledging your access and consent to receive and sign documents electronically

To confirm to us that you can access this information electronically, which will be similar to other electronic notices and disclosures that we will provide to you, please confirm that you have read this ERSD, and (i) that you are able to print on paper or electronically save this ERSD for your future reference and access; or (ii) that you are able to email this ERSD to an email address where you will be able to print on paper or save it for your future reference and access. Further, if you consent to receiving notices and disclosures exclusively in electronic format as described herein, then select the check-box next to 'I agree to use electronic records and signatures' before clicking 'CONTINUE' within the DocuSign system.

By selecting the check-box next to 'I agree to use electronic records and signatures', you confirm that:

- You can access and read this Electronic Record and Signature Disclosure; and
- You can print on paper this Electronic Record and Signature Disclosure, or save or send this Electronic Record and Disclosure to a location where you can print it, for future reference and access; and
- Until or unless you notify [redacted] as described above, you consent to receive exclusively through electronic means all notices, disclosures, authorizations, acknowledgements, and other documents that are required to be provided or made available to you by [redacted] during the course of your relationship with [redacted]

Supplementary Note 3. CONSORT 2025 checklist of information to include when reporting a randomised trial

Trial: Losartan and Prednisolone for Post-COVID Syndrome and Cardiac Inflammation (Myoflame-19). EudraCT 2022-001682-12; NCT05619653. Reported against the CONSORT 2025 statement (Hopewell et al., 2025), which supersedes CONSORT 2010. Page numbers refer to the main manuscript and should be confirmed against the final typeset version.

Section/Topic	Item No	Checklist item	Reported in (section / page)
Title and abstract			
Title and structured abstract	1a	Identification as a randomized trial	Title page (“A Randomized, Double-Blind, Placebo-Controlled Trial”)
	1b	Structured summary of the trial design, methods, results, and conclusions	Abstract
Open science			
Trial registration	2	Name of trial registry, identifying number (with URL) and date of registration	Title page (Clinical Trial Identifiers); Methods (EudraCT 2022-001682-12; NCT05619653; CTIS 2024-516463-84-00)
Protocol and statistical analysis plan	3	Where the trial protocol and statistical analysis plan can be accessed	Methods; Supplementary Note 1 (Trial Protocol) and Supplementary Note 2 (Statistical Analysis Plan); also publicly available on CTIS
Data sharing	4	Where and how the individual de-identified participant data (including data dictionary), statistical code and any other materials can be accessed	Data availability statement; Code availability statement
Funding and conflicts of interest	5a	Sources of funding and other support, and role of funders in the design, conduct, analysis and reporting of the trial	Funding; Role of the funding source
	5b	Financial and other conflicts of interest of the manuscript authors	Competing Interests
Introduction			
Background and rationale	6	Scientific background and rationale	Introduction
Objectives	7	Specific objectives related to benefits and harms	Introduction (final paragraph); Methods – Outcomes
Methods			
Patient and public involvement	8	Details of patient or public involvement in the design, conduct and reporting of the trial	Acknowledgments (engagement and advocacy of patient organizations)
Trial design	9	Description of trial design including type of trial, allocation ratio, and framework	Methods – Study design and participants; Randomization and masking (parallel-group, 1:1)
Changes to trial protocol	10	Important changes to the trial after it commenced including any outcomes or analyses that were not prespecified, with reason	Methods – Outcomes; protocol amendments described in Supplementary Note 1
Trial setting	11	Settings and locations where the trial was conducted	Methods – Study design and participants; Results – Baseline (four centres, five countries)
Eligibility criteria	12a	Eligibility criteria for participants	Methods – Participants (inclusion and exclusion criteria)
	12b	If applicable, eligibility criteria for sites and	Not applicable (oral drug

		for individuals delivering the interventions	intervention); standardized CMR procedures across centres are described in Methods – Trial procedures
Intervention and comparator	13	Intervention and comparator with sufficient details to allow replication	Methods – Randomization and masking (losartan plus prednisolone dosing; matching placebo)
Outcomes	14	Prespecified primary and secondary outcomes, including measurement variable, analysis metric, method of aggregation, and time point	Methods – Outcomes; Statistical analysis
Harms	15	How harms were defined and assessed	Methods – Safety and compliance; Outcomes
Sample size	16a	How sample size was determined, including all assumptions	Methods – Statistical analysis; Supplementary Information (Sample size); Supplementary Note 2
	16b	Explanation of any interim analyses and stopping guidelines	Not applicable — no interim analysis was planned or conducted, and no formal stopping rule was applied
Randomization: Sequence generation	17a	Who generated the random allocation sequence and the method used	Methods – Randomization and masking; Supplementary Notes 1–2
	17b	Type of randomization and details of any restriction (eg, stratification, blocking)	Methods – Randomization and masking (central 1:1; stratification by site removed by amendment); Supplementary Notes 1–2
Allocation concealment mechanism	18	Mechanism used to implement the random allocation sequence	Methods – Randomization and masking (central allocation; matching placebos)
Implementation	19	Whether personnel who enrolled and assigned participants had access to the random allocation sequence	Methods – Randomization and masking
Blinding	20a	Who was blinded after assignment to interventions	Methods – Randomization and masking (double-blind: participants and investigators); Trial procedures (blinded central CMR analysis)
	20b	If blinded, how blinding was achieved and description of the similarity of interventions	Methods – Randomization and masking (matching placebos)
Statistical methods	21a	Statistical methods used to compare groups for primary and secondary outcomes, including harms	Methods – Statistical analysis
	21b	Definition of who is included in each analysis, and in which group	Methods – Statistical analysis (modified intention-to-treat; per-protocol); Results
	21c	How missing data were handled in the analysis	Methods – Statistical analysis (complete-case analysis; no imputation)
	21d	Methods for any additional analyses, distinguishing prespecified from post hoc	Methods – Statistical analysis (baseline-adjusted ANCOVA; per-protocol analysis)
Results			
Participant flow, including flow diagram	22a	For each group, the numbers randomly assigned, receiving intended intervention, and analyzed for the primary outcome	Results – Baseline; Figure 1 (CONSORT flow diagram)

	22b	For each group, losses and exclusions after randomization, with reasons	Results – Baseline; Figure 1
Recruitment	23a	Dates defining the periods of recruitment and follow-up for outcomes of benefits and harms	Methods (first participant 14 December 2022; last 30 March 2025); Results – Baseline
	23b	If relevant, why the trial ended or was stopped	Results – Baseline (planned enrolment completed)
Intervention and comparator delivery	24a	Intervention and comparator as they were actually administered	Methods – Randomization and masking; Safety and compliance; Results (compliance and tolerability)
	24b	Concomitant care received during the trial for each group	Methods – Participants (guideline-directed cardiac therapy excluded); standardized lifestyle guidance (Supplementary – Lifestyle Guidance)
Baseline data	25	A table showing baseline demographic and clinical characteristics for each group	Table 1
Numbers analyzed, outcomes and estimation	26	For each primary and secondary outcome, by group: number analyzed; number with available data at the time point; result and estimated effect size with precision (eg, 95% CI); for binary outcomes, absolute and relative effect sizes	Results – Primary and secondary endpoints; Tables 2–3; Figure 2
Harms	27	All harms or unintended events in each group	Results – Safety, compliance and tolerability; Supplementary Tables
Ancillary analyses	28	Any other analyses performed, distinguishing pre-specified from post hoc	Methods – Statistical analysis; Results
Discussion			
Interpretation	29	Interpretation consistent with results, balancing benefits and harms, and considering other relevant evidence	Discussion
Limitations	30	Trial limitations, addressing sources of potential bias, imprecision, generalisability, and, if relevant, multiplicity of analyses	Discussion (limitations paragraph)

We strongly recommend reading this checklist in conjunction with the CONSORT 2025 Explanation and Elaboration for important clarifications on all the items. For up-to-date references relevant to this checklist, see www.consort-statement.org.

4 Supplementary References

1. Puntmann VO, Beitzke D, Kammerlander A, et al. Design and rationale of MYOFLAME-19 randomised controlled trial: MYOcardial protection to reduce post-COVID inFLAMmatory heart disease using cardiovascular magnetic resonance Endpoints. J Cardiovasc Magn Reson 2024;27(1):101121.

2. Goudsmit EM, Nijs J, Jason LA, Wallman KE. Pacing as a strategy to improve energy management in myalgic encephalomyelitis/chronic fatigue syndrome: a consensus document. Disabil Rehabilitation 2012;34(13):1140–7.

3. Sudre CH, Murray B, Varsavsky T, et al. Attributes and predictors of long COVID. Nat Med [Internet] 2021;27(4):626–31. Available from: https://www.nature.com/articles/s41591-021-01292-y

4. Puntmann VO, Martin S, Shchendrygina A, et al. Long-term cardiac pathology in individuals with mild initial COVID-19 illness. Nat Med 2022;28(10):2117–23.
