# Supplementary material for: Losartan and prednisolone for post-COVID syndrome and cardiac inflammation: a randomized, double-blind, placebo-controlled trial
Source: Nat Commun. 2026 Jul 30;17:7599. doi: 10.1038/s41467-026-75991-w (PMC13424660; doi:10.1038/s41467-026-75991-w)
Supplement: Supplementary file 3 — Transparent Peer Review File [file 41467_2026_75991_MOESM3_ESM.pdf]

nature portfolio

Peer Review File

Losartan and Prednisolone for Post-COVID Syndrome and Cardiac Inflammation: A Randomized, Double-Blind, Placebo-Controlled Trial

Corresponding Author: Dr Valentina Puntmann

This file contains all reviewer reports in order by version, followed by all author rebuttals in order by version.

Version 0:

Reviewer comments:

Reviewer #1

(Remarks to the Author)
The manuscript by Puntmann and co-workers titled "Cardioprotection and Immunomodulation with Losartan and Prednisolone for Post-COVID Inflammatory Cardiac Involvement" investigates the effects of Losartan and Prednisolone treatment in an investigator-initiated, multicenter, randomized, double-blind, placebo-controlled trial of losartan and prednisolone for 16 weeks in 279 individuals with CMR-defined inflammatory cardiac involvement.
The study showed a change in LVEF with a between-group difference of 0.75 percentage points (unpaired t-test, p = 0.10). A prespecified baseline-adjusted ANCOVA for baseline LVEF demonstrated a significant measurable between-group difference of 1.0 percentage points.

General comments:
The manuscript is well written, the introduction and aim are easy to follow, and the results are somewhat easy to follow. Notwithstanding, this reviewer is somewhat uneasy about the presentation of the changes in anatomical and functional measurements. It is not surprising that treatment with Losartan reduces systolic and diastolic blood pressure even in subjects with normal blood pressure. Thus, the reduction in blood pressure, in turn, reduces left ventricular afterload and should reduce left ventricular end-diastolic volume index, NT-pro-brain natriuretic peptide, and left atrial size. The data you present does show an increase in left ventricular ejection fraction, a reduction in NT-pro-brain natriuretic peptide, and a reduction in left atrial size. However, there seems to be no reduction in left ventricular end-diastolic volume index.
• Can the authors explain this result?
• Do the authors think that the expected reduction in left ventricular end-diastolic volume by Losartan is attenuated by the volume-loading caused by the Prednisolone treatment?
• Does it have to do with the presentation of least-squares mean changes?
Furthermore, Table 2 is difficult for this reviewer, and most likely also for most readers, to understand. The column titles show LS means, with the top title labeled "absolute change". However, parameters show "n,%". Also, the right-hand panel shows the percentage change by W16, but no percentage in the parameters. It is a bit confusing.
This reviewer strongly suggests that the authors present the changes in the measured variables more clearly.
Another point where this reviewer is uneasy is the lightness with which the authors suggest that the measurable increase in left ventricular ejection fraction supports the mechanistic hypothesis that attenuation of low-grade inflation can yield functional recovery in a post-COVID condition. There are two issues at play when treating with Losartan compared to a placebo.
1) Left ventricular ejection is afterload dependent; thus, systolic blood pressure should be taken into account when comparing left ventricular ejection fraction at different levels of systolic blood pressure (this may be why the ANCOVA analysis shows larger differences in left ventricular ejection fraction, however, this is difficult for the reader to understand because the statistics paragraph is lapidary at best and there are no reference to exactly how the data where treated during the statistical analysis and which adjustments were done). One way to circumvent this is to evaluate the stress-corrected left ventricular ejection fraction, i.e., left ventricular ejection fraction adjusted by left ventricular circumferential end-systolic stress.
2) Losartan impacts on left ventricular hypertrophy. However, one prerequisite when calculating left ventricular ejection fraction is that left ventricular wall thickness is minuscule. The data you present here suggest a very similar left ventricular mass index, so this reviewer thinks this latter point probably does not play a big role.

Notwithstanding, this reviewer is pretty sure that even treatment with Losartan in normal subjects would slightly increase left ventricular ejection fraction by reducing blood pressure. Thus, this reviewer is uneasy with the authors' suggestion that the increase in left ventricular ejection fraction is a mechanistic explanation for functional recovery. It is an association at best.
Minor comments:
The manuscript is well written; however, there are numerous abbreviations, and the reviewer stopped counting after 30. Please reduce to increase readability.

Reviewer #2

(Remarks to the Author)
This study is a multicentre randomised controlled trial comparing losartan plus predisolone vs placebo in 279 participants with long COVID 19 and CMR-defined inflammatory cardiac involvement between December 2022 and March 2025 across five European countries. The primary endpoint was the absolute change in LVEF from baseline to 16 weeks.

Overall, the manuscript is well structured, well written and data are well placed into a clinical context.

I have the following minor comments:

In the statistical analysis section (line 340). It stated that "missingness was minimal, and no imputation was required.". Please report the percentage of missing values, particularly for the primary outcome. In addition, please make the definition of changes clear: both absolute change and percentage change and explain the reasons that both changes were used.

In the results section (lines 107-108). "A total of 246 participants completed the week-16 CMR follow-up, and the per-protocol population included 244 individuals". Please indicate the two cases excluded.
Line 128. "… was not statistically significant.". Avoid using "statistically significant" as a result is either significant or not depending on the parameters set (See Sterne, J. A., & Smith, G. D. (2001). Sifting the evidence-what's wrong with significance tests? Physical therapy, 81(8), 1464-1469.). Please change this accordingly in the main text.
Line 131. Suggest adding "Table 2" after reporting the results.
Table 1. Noted that the number of participants listed in each group (n=124 for intervention group, n=122 for the other group) was not based on intention-to treat (ITT), not all randomised participants were included. Please clarify the definition of intention-to-treat population in the study. In addition, please add the unit of age, such as years. Also ">1cm, n,%" shown in the table was not clear.

Reviewer #3

(Remarks to the Author)
This manuscript reports a multicenter, randomized, double-blind, placebo-controlled trial testing combined losartan plus low-dose prednisolone for 16 weeks in adults with post-COVID cardiac symptoms and CMR-defined inflammatory cardiac involvement, excluding structural heart disease. The prespecified primary endpoint was absolute change in CMR-derived LVEF from baseline to week 16. The confirmatory analysis using an unpaired t-test showed a between-group difference of 0.74 percentage points (95% CI –0.14 to 1.62; p=0.10). In a prespecified supportive, baseline-adjusted ANCOVA, the estimated difference was 0.99 LVEF percentage points (95% CI 0.15–1.83; p=0.021).
The study addresses an important unmet need and the CMR-based endotype enrichment is a meaningful strategy to reduce heterogeneity in post-COVID cardiac syndromes. The work is potentially impactful as mechanistic/physiologic proof-of-concept using objective CMR endpoints and central blinded analysis. However, the clinical practice implications are tempered by the negative confirmatory primary analysis, the modest absolute effect size with uncertain clinical meaningfulness, and limited evidence on durability of a potential benefit. Overall, the findings support the rationale for larger confirmatory trials rather than definitive evidence of efficacy.
Comments:
1. The manuscript correctly notes that the prespecified confirmatory primary analysis did not reach statistical significance (p=0.10), but the abstract and discussion emphasize the supportive ANCOVA and use phrasing that may be read as confirmatory efficacy. I recommend tightening the framing throughout the article: this is a signal consistent with benefit supported by baseline-adjusted modeling, but the confirmatory primary test was not significant.
2. The abstract wording "ANCOVA for low-normal baseline LVEF" is ambiguous and reads like a subgroup-restricted analysis, whereas elsewhere the ANCOVA appears to be baseline adjustment. Please clarify explicitly whether ANCOVA was (i) performed in the full analysis set with baseline LVEF as covariate, or (ii) restricted to a "low-normal LVEF" subset. If subgroup-restricted, define the threshold, report sample size, confirm prespecification, and briefly address multiplicity.
3. The analysis population labeled "ITT" appears non-standard: 279 were randomized, but 246 were included in the ITT analysis (and completed week-16 CMR), with post-randomization exclusions such as treatment not initiated, refusal, lack of compliance, etc. This resembles a modified ITT / complete-case analysis and could bias estimates if exclusions relate to tolerability or outcome trajectory. Please define analysis populations precisely (safety, ITT vs modified ITT, PP), justify exclusions, report baseline characteristics of included vs excluded participants, and provide sensitivity analyses addressing missing week-16 CMR (e.g., imputation or conservative bounds).
4. Even when statistically significant, the absolute effect size on LVEF is modest (~1 percentage point). Please contextualize clinical meaningfulness, including expected multicenter CMR-LVEF variability (test–retest/inter-reader), and relate this to the lack of improvement in quality-of-life measures despite changes in some symptom scores.
5. Symptom improvements should be interpreted cautiously because structured pacing/hydration guidance was provided in both arms, which could contribute to improvement in both groups and dilute between-group differences. Please briefly

describe what the guidance entailed, how it was standardized across centers, whether adherence/fidelity was assessed, and keep symptom findings clearly framed as exploratory given multiple secondary endpoints without multiplicity adjustment.
6. How are the significant changes in cholesterol levels (in particluar in the placebo group) explained?

Version 1:

Reviewer comments:

Reviewer #2

(Remarks to the Author)
I thank the authors for their careful and thorough consideration of my previous remarks. I have no further comments.

Reviewer #3

(Remarks to the Author)
The authors have adequately addressed the raised concerns.

Open Access This Peer Review File is licensed under a Creative Commons Attribution 4.0 International License, which permits use, sharing, adaptation, distribution and reproduction in any medium or format, as long as you give appropriate credit to the original author(s) and the source, provide a link to the Creative Commons license, and indicate if changes were made.
In cases where reviewers are anonymous, credit should be given to 'Anonymous Referee' and the source.
The images or other third party material in this Peer Review File are included in the article's Creative Commons license, unless indicated otherwise in a credit line to the material. If material is not included in the article's Creative Commons license and your intended use is not permitted by statutory regulation or exceeds the permitted use, you will need to obtain permission directly from the copyright holder.
To view a copy of this license, visit https://creativecommons.org/licenses/by/4.0/

Responses to Reviewers' comments

We are very grateful for the thorough review and the reviewers' comments. We have revised the paper substantially in response. We believe that the manuscript has improved as a result.

Reviewer 1:

General comments:

The manuscript is well written, the introduction and aim are easy to follow, and the results are somewhat easy to follow.

Notwithstanding, this reviewer is somewhat uneasy about the presentation of the changes in anatomical and functional measurements. It is not surprising that treatment with Losartan reduces systolic and diastolic blood pressure even in subjects with normal blood pressure. Thus, the reduction in blood pressure, in turn, reduces left ventricular afterload and should reduce left ventricular end-diastolic volume index, NT-pro-brain natriuretic peptide, and left atrial size. The data you present does show an increase in left ventricular ejection fraction, a reduction in NT-pro-brain natriuretic peptide, and a reduction in left atrial size. However, there seems to be no reduction in left ventricular end-diastolic volume index.

• Can the authors explain this result?

• Do the authors think that the expected reduction in left ventricular end-diastolic volume by Losartan is attenuated by the volume-loading caused by the Prednisolone treatment?

• Does it have to do with the presentation of least-squares mean changes?

VP/EN: We thank the reviewer for this thoughtful physiological interpretation. The investigational regimen purposefully combined losartan with low-dose prednisolone, reflecting the dual therapeutic intent of hemodynamic modulation and attenuation of inflammatory myocardial involvement. While RAAS blockade alone may be expected to promote modest intravascular volume contraction and reductions in ventricular filling volumes, concomitant glucocorticoid therapy can exert countervailing effects through sodium and fluid retention. Interpretation of volumetric findings must therefore consider the intervention's combined pharmacologic profile rather than RAAS modulation in isolation. In this context, the absence of a measurable increase in LV end-diastolic volume index argues against clinically meaningful steroid-mediated volume loading. This is further supported by reductions in NT-proBNP and left atrial size, which suggest improved filling pressures rather than preload expansion. Presenting least-squares mean (LS-mean) changes does not create the absence of an LVEDVi change; it only affects the adjusted estimate of the change. The conclusion (little/no change in LVEDVi) is the same in raw and adjusted summaries.

Accordingly, the observed increase in LVEF is unlikely to be explained by loading-related augmentation of stroke volume. We have clarified this interpretation and tempered mechanistic language in the revised Discussion.

Furthermore, Table 2 is difficult for this reviewer, and most likely also for most readers, to understand. The column titles show LS means, with the top title labelled "absolute change". However, parameters show "n,%". Also, the right-hand panel shows the percentage change by W16, but no percentage in the parameters. It is a bit confusing.

This reviewer strongly suggests that the authors present the changes in the measured variables more clearly.

VP/EN: We thank the reviewer for highlighting the need to improve the clarity of Table 2. We have adjusted the presentation accordingly. In the revised manuscript, we have restructured Table 2 by separating continuous and categorical variables into distinct panels to enhance readability and align the parameter types with the statistical summaries.

Least-squares mean changes were derived from ANCOVA models that included treatment group as a fixed factor and the baseline value of the outcome parameter as a covariate. This approach adjusts for baseline variability in the specific parameter being analysed and provides an estimate of the adjusted between-group treatment effect.

Specifically, we have:

- **Clarified column headers to distinguish absolute from relative (%) change.**
- **Added explanatory footnotes defining least-squares mean estimates and model adjustments.**
- **Ensured that percentage change values are explicitly labelled and expressed relative to baseline.**

Another point where this reviewer is uneasy is the lightness with which the authors suggest that the measurable increase in left ventricular ejection fraction supports the mechanistic hypothesis that attenuation of low-grade inflation can yield functional recovery in a post-COVID condition. There are two issues at play when treating with Losartan compared to a placebo.

1) Left ventricular ejection is afterload dependent; thus, systolic blood pressure should be taken into account when comparing left ventricular ejection fraction at different levels of systolic blood pressure (this may be why the ANCOVA analysis shows larger differences in left ventricular ejection fraction, however, this is difficult for the reader to understand because the statistics paragraph is lapidary at best and there are no reference to exactly how the data where treated during the statistical analysis and which adjustments were done). One way to circumvent this is to evaluate the stress-corrected left ventricular ejection fraction, i.e., left ventricular ejection fraction adjusted by left ventricular circumferential end-systolic stress.

2) Losartan impacts on left ventricular hypertrophy. However, one prerequisite when calculating left ventricular ejection fraction is that left ventricular wall thickness is minuscule. The data you present here suggest a very similar left ventricular mass index, so this reviewer thinks this latter point probably does not play a big role.

Notwithstanding, this reviewer is pretty sure that even treatment with Losartan in normal subjects would slightly increase left ventricular ejection fraction by reducing blood pressure. Thus, this reviewer is uneasy with the authors' suggestion that the increase in left ventricular ejection fraction is a mechanistic explanation for functional recovery. It is an association at best.

VP/EN: We thank the reviewer for these mechanistically focused comments.

We agree that LVEF is load-dependent and may be influenced by afterload reduction. Functional findings are not interpreted in isolation but alongside structural and biomarker indices. LVEF improved without measurable LV end-diastolic volume expansion and with reductions in NT-proBNP and left atrial size, arguing against dominant preload effects. The contribution of afterload cannot be excluded or quantified in this study.

Thank you for indicating the need for clearer articulation of the role of baseline-adjusted ANCOVA analyses. The study was conducted in an emerging disease population with subtle functional impairment and limited prior therapeutic data. The confirmatory primary analysis used an unpaired t-test on absolute change in LVEF, providing a direct comparison of mean change between groups without baseline adjustment.

Baseline-adjusted ANCOVA incorporates baseline LVEF as a covariate, enabling comparison of follow-up values while accounting for starting differences. This reduces variance related to baseline dispersion and regression to the mean, and is particularly informative in cohorts with preserved baseline function and a narrow dynamic range. ANCOVA offers complementary insight into treatment-associated physiological signal detection without superseding the confirmatory analysis. Its interpretation remains supportive and hypothesis-generating. The combined analytical framework informs mechanistic understanding and the design of subsequent confirmatory trials.

We have revised the manuscript to clarify both the interpretive scope of LVEF findings and the role of the ANCOVA analyses.

Minor comments:

The manuscript is well written; however, there are numerous abbreviations, and the reviewer stopped counting after 30. Please reduce to increase readability.

VP/EN: Thank you very much. In response to the reviewer, we have reduced the number of abbreviations considerably.

Reviewer #2 (Remarks to the Author):

This study is a multicentre randomised controlled trial comparing losartan plus predisolone vs placebo in 279 participants with long COVID 19 and CMR-defined inflammatory cardiac involvement between December 2022 and March 2025 across five European countries. The primary endpoint was the absolute change in LVEF from baseline to 16 weeks.

Overall, the manuscript is well structured, well written and data are well placed into a clinical context.

I have the following minor comments:

In the statistical analysis section (line 340). It stated that "missingness was minimal, and no imputation was required." Please report the percentage of missing values, particularly for the primary outcome. In addition, please make the definition of changes clear: both absolute change and percentage change and explain the reasons that both changes were used.

VP/EN: We thank the reviewer for this request for clarification.

For the primary endpoint, inclusion in both the modified intention-to-treat and per-protocol populations required evaluable LVEF measurements at both baseline and week 16. Participants who did not complete the week-16 CMR assessment were therefore excluded from the primary efficacy analyses. Consequently, no missing primary endpoint values were present within the analysed datasets.

Participants who discontinued prior to week 16 remained included in safety analyses if they had taken at least one dose of IMP, with all available data up to the point of withdrawal contributing to safety evaluation, in accordance with standard trial reporting conventions.

We have revised the Methods and Results sections to clarify the definitions of the analysis population and the data inclusion principles.

Absolute change from baseline to week 16 was prespecified for the primary endpoint and represents the principal efficacy metric for LVEF. This measure reflects the direct physiological difference in ventricular function over time and aligns with established reporting conventions for interventional cardiac imaging trials.

Percentage change was additionally presented for selected secondary parameters in a descriptive capacity. The rationale for including percentage change is to facilitate interpretive comparison across variables with differing baseline scales and units of measurement. Expressing change relative to baseline can provide contextual insight into proportional shifts in biomarkers, volumetric indices, and tissue parameters that may not be readily comparable on an absolute scale.

We have revised the Methods section and Table legends to clarify this analytical distinction.

In the results section (lines 107-108). "A total of 246 participants completed the week-16 CMR follow-up, and the per-protocol population included 244 individuals". Please indicate the two cases excluded.

VP/EN: We thank the reviewer for this request for clarification. Two participants who completed the week-16 CMR follow-up. Both participants received ≥4 weeks of treatment and therefore met modified intention-to-treat criteria, but discontinued study medication before reaching the prespecified adherence threshold required for per-protocol inclusion (>75% of the trial period). The legend for the Consort has been revised to clarify this distinction.

Line 128. "… was not statistically significant.". Avoid using "statistically significant" as a result is either significant or not depending on the parameters set (See Sterne, J. A., & Smith, G. D. (2001). Sifting the evidence-what's wrong with significance tests? Physical therapy, 81(8), 1464-1469.). Please change this accordingly in the main text.

VP/EN: We thank the reviewer for this thoughtful suggestion. We have revised the manuscript to replace dichotomous phrasing such as "statistically significant" or "not statistically significant" with language referencing prespecified significance thresholds and reporting effect estimates where appropriate.

Line 131. Suggest adding "Table 2" after reporting the results.

VP/EN: Thank you very much, adjusted as requested.

Table 1. Noted that the number of participants listed in each group (n=124 for intervention group, n=122 for the other group) was not based on intention-to treat (ITT), not all randomised participants were included. Please clarify the definition of intention-to-treat population in the study. In addition, please add the unit of age, such as years. Also ">1cm, n,%" shown in the table was not clear.

VP/EN: We thank the reviewer for this helpful request for clarification.

In this study, the intention-to-treat population corresponds to a modified ITT (full analysis set) and included all randomized participants who had both baseline and week-16 CMR assessments and received at least 4 weeks of study treatment, in accordance with the prespecified statistical analysis plan. Participants who did not complete week-16 imaging were therefore not included in efficacy analyses but remained included in safety analyses. This approach aligns with full analysis set methodology for imaging-endpoint trials requiring paired measurements

We have revised the Methods section and the Table 1 legend to clarify this definition and population structure.

In addition, the unit for age has been added (years), and the variable previously labelled ">1 cm, n (%)" has been clarified to specify the anatomical measurement threshold of pathophysiological clinical relevance.

Reviewer #3 (Remarks to the Author):

This manuscript reports a multicenter, randomized, double-blind, placebo-controlled trial testing combined losartan plus low-dose prednisolone for 16 weeks in adults with post-COVID cardiac symptoms and CMR-defined inflammatory cardiac involvement, excluding structural heart disease. The prespecified primary endpoint was absolute change in CMR-derived LVEF from baseline to week 16. The confirmatory analysis using an unpaired t-test showed a between-group difference of 0.74 percentage points (95% CI –0.14 to 1.62; p=0.10). In a prespecified supportive, baseline-adjusted ANCOVA, the estimated difference was 0.99 LVEF percentage points (95% CI 0.15–1.83; p=0.021).

The study addresses an important unmet need and the CMR-based endotype enrichment is a meaningful strategy to reduce heterogeneity in post-COVID cardiac syndromes. The work is potentially impactful as mechanistic/physiologic proof-of-concept using objective CMR endpoints and central blinded analysis. However, the clinical practice implications are tempered by the negative confirmatory primary analysis, the modest absolute effect size with uncertain clinical meaningfulness, and limited evidence on durability of a potential benefit. Overall, the findings support the rationale for larger confirmatory trials rather than definitive evidence of efficacy.

Comments:

1. The manuscript correctly notes that the prespecified confirmatory primary analysis did not reach statistical significance (p=0.10), but the abstract and discussion emphasize the supportive ANCOVA and use phrasing that may be read as confirmatory efficacy. I recommend tightening the framing throughout the article: this is a signal consistent with benefit supported by baseline-adjusted modeling, but the confirmatory primary test was not significant.

VP/EN: We thank the reviewer for this important comment. We have revised the abstract and discussion accordingly to transparently reflect the prespecified confirmatory hierarchy. The unadjusted primary analysis is now presented as neutral, and the baseline-adjusted ANCOVA is used as a supportive model, providing complementary insight into treatment-associated functional change without altering confirmatory inference. Interpretive language implying efficacy has been removed throughout.

2. The abstract wording “ANCOVA for low-normal baseline LVEF” is ambiguous and reads like a subgroup-restricted analysis, whereas elsewhere the ANCOVA appears to be baseline adjustment. Please clarify explicitly whether ANCOVA was (i) performed in the full analysis set with baseline LVEF as covariate, or (ii) restricted to a “low-normal LVEF” subset. If subgroup-restricted, define the threshold, report sample size, confirm prespecification, and briefly address multiplicity.

VP/EN: We thank the reviewer for noting this ambiguity. The low-normal LVEF was intended to describe the prespecified population, not a subgroup. We removed this term to avoid confusion. We also expanded the limitation section to clarify that ANCOVA was conducted in the full analysis set with baseline LVEF included as a covariate, consistent with conventional baseline-adjusted modelling. The observed absolute change should be interpreted within the context of a physiologically

preserved cohort, which inherently constrains the dynamic range for measurable improvement over a short intervention period. We have revised the abstract and methods and limitations to clarify this point and removed wording that could imply subgroup restriction. We concur with the reviewer that the findings support the rationale for larger confirmatory trials.

3. The analysis population labeled “ITT” appears non-standard: 279 were randomized, but 246 were included in the ITT analysis (and completed week-16 CMR), with post-randomization exclusions such as treatment not initiated, refusal, lack of compliance, etc. This resembles a modified ITT / complete-case analysis and could bias estimates if exclusions relate to tolerability or outcome trajectory. Please define analysis populations precisely (safety, ITT vs modified ITT, PP), justify exclusions, report baseline characteristics of included vs excluded participants, and provide sensitivity analyses addressing missing week-16 CMR (e.g., imputation or conservative bounds).

We thank the reviewer for this important point. We agree that our efficacy population corresponds to a modified intention-to-treat set (full analysis set) rather than classic ITT, owing to the prespecified primary endpoint being the change in CMR-derived LVEF from baseline to week 16, therefore, the estimation requires paired CMR measurements. Only participants with both measurements were included in the efficacy analyses. We define the study populations more precisely, all randomised, safety, modified ITT/full analysis set, and per-protocol.

We describe post-randomisation exclusions in the Consort chart. There was no difference in attrition between the intervention and placebo groups. The attrition rate was modestly higher than expected (12% vs 8%). Nine participants did not proceed with therapeutic participation after eligibility confirmation. Included in the limitations.

We also clarified that participants without week-16 CMR, but who have taken at least 1 dose of IMP were retained for safety analyses. Table 1S now includes all randomised subjects.

4.Even when statistically significant, the absolute effect size on LVEF is modest (~1 percentage point). Please contextualize clinical meaningfulness, including expected multicenter CMR-LVEF variability (test–retest/inter-reader), and relate this to the lack of improvement in quality-of-life measures despite changes in some symptom scores.

VP/EN: We thank the reviewer for this important comment.

We thank the reviewer for this important comment. We agree that the absolute LVEF difference is modest, and not significant by the confirmatory unpaired t-test. Supportive ANCOVA provides an estimate of functional improvement with biological plausibility. We contextualise these results relative to baseline variability, in light of the cohort’s preserved baseline systolic function, which limited the dynamic range for improvement. These insights are valuable and will inform future studies. Added to the Methods and limitations.

Imaging was analysed in a centralised core laboratory with standardised acquisition and blinded reads, reducing measurement variability.

We also address the dissociation between imaging changes and quality-of-life outcomes, noting that QoL measures are complex and may be less sensitive to short-term physiological change and reflect broader systemic determinants.

Added to the discussion.

5. Symptom improvements should be interpreted cautiously because structured pacing/hydration guidance was provided in both arms, which could contribute to improvement in both groups and dilute between-group differences. Please briefly describe what the guidance entailed, how it was standardized across centers, whether adherence/fidelity was assessed, and keep symptom findings clearly framed as exploratory given multiple secondary endpoints without multiplicity adjustment.

VP/EN: We thank the reviewer for this important point. Structured pacing and hydration guidance was provided to participants in both treatment arms, as part of standardized supportive care. We have expanded the Supplementary Methods to describe the content, delivery, and cross-site standardisation of all procedures though initiation visit. Adherence to pharmacological therapy was monitored; however, fidelity to pacing and hydration recommendations was not formally quantified. Symptom outcomes are now explicitly framed as exploratory secondary endpoints without multiplicity adjustment and interpreted cautiously within this context.

6. How are the significant changes in cholesterol levels (in particular in the placebo group) explained?

We thank the reviewer for highlighting this observation. We believe that the apparent between-group difference in total cholesterol was partly driven by an increase in HDL in the intervention arm, while LDL remained largely unchanged. Although exploratory, this directionality is consistent with prior reports describing pleiotropic metabolic effects of angiotensin receptor blockade, including preservation or modest increases in HDL cholesterol (Olsen j et al J Hypertens. 2009 Mar;27(3):567-74. doi: 10.1097/HJH.0b013e32831daf96). However, given the exploratory nature of lipid analyses and absence of multiplicity adjustment, this finding should be interpreted cautiously and warrants confirmation in future studies.
